# Annotating very high-resolution satellite imagery: A whale case study

**DOI:** 10.1016/j.mex.2023.102040

**Published:** 2023-01-25

**Authors:** Hannah Charlotte Cubaynes, Penny Joanna Clarke, Kimberly Thea Goetz, Tyler Aldrich, Peter Thomas Fretwell, Kathleen Elise Leonard, Christin Brangwynne Khan

**Affiliations:** a British Antarctic Survey, High Cross, Madingley Road, Cambridge, CB3 0ET, United Kingdom; b School of Engineering, The University of Edinburgh, Sanderson Building, Robert Stevenson Road, The King's Buildings, Edinburgh, EH9 3FB, United Kingdom; c Marine Mammal Laboratory, Alaska Fisheries Science Center, National Marine Fisheries Service, NOAA, Seattle, Washington, United States; d Northeast Fisheries Science Center, National Marine Fisheries Service, NOAA, Woods Hole, MA, United States; e Protected Resources Division, Alaska Regional Office, National Marine Fisheries Service, NOAA, Anchorage, AK, United States

**Keywords:** VHR optical satellite image, Wildlife, Cetacean, Labeling, AI-ready data, Machine learning, Satellite image annotation to create point, bounding boxes and image datasets to train automated systems.

## Abstract

The use of very high-resolution (VHR) optical satellites is gaining momentum in the field of wildlife monitoring, particularly for whales, as this technology is showing potential for monitoring the less studied regions. However, surveying large areas using VHR optical satellite imagery requires the development of automated systems to detect targets. Machine learning approaches require large training datasets of annotated images. Here we propose a standardised workflow to annotate VHR optical satellite imagery using ESRI ArcMap 10.8, and ESRI ArcGIS Pro 2.5., using cetaceans as a case study, to develop AI-ready annotations.

• A step-by-step protocol to review VHR optical satellite images and annotate the features of interest.
• A step-by-step protocol to create bounding boxes encompassing the features of interest.
• A step-by-step guide to clip the satellite image using bounding boxes to create image chips.

## Graphical abstract

**Figure:**
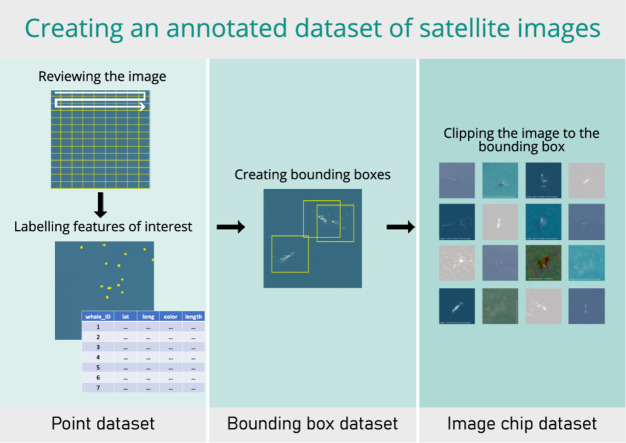

Specifications table

**Table:** 

Subject area:	Earth and Planetary Sciences
More specific subject area:	Earth observation
Name of your method:	Satellite image annotation to create point, bounding boxes and image datasets to train automated systems.
Name and reference of original method:	Cubaynes, H.C., Fretwell, P.T. (2022) Whales from space dataset, an annotated satellite image dataset of whales for training machine learning models. *Sci. Data* **9**, 245. https://doi.org/10.1038/s41597-022-01377-4
Resource availability:	Software: ESRI ArcGIS Pro 2.5, ESRI ArcMap 10.8

## Related research article

For a published article:

Cubaynes, H.C., Fretwell, P.T. (2022) Whales from space dataset, an annotated satellite image dataset of whales for training machine learning models. *Sci. Data* 9 (2022), 245. https://doi.org/10.1038/s41597–022–01377–4

## Background

The latest advancements of very high-resolution (VHR) optical satellite imagery (below 1 m spatial resolution) show tremendous potential for monitoring wildlife in recent trials [1], [2], [3], [4], [5], [6]. There are also a few VHR satellites with synthetic aperture radar (SAR) sensor, which can image in the dark and through clouds by returning an image of the surface roughness. However, SAR sensor applications to wildlife surveys is at an early stage [4]. Therefore, in this study we focus on VHR optical satellites, and refer to them as VHR satellites in the remainder of the text.

VHR satellite imagery is currently being assessed as a complementary approach to traditional survey methods for monitoring whales, and is particularly beneficial for less studied regions and over large areas [3,7]. Monitoring whales is crucial, particularly for estimating abundance and distribution, which is of broad interest to government agencies, academic, and commercial institutions around the globe. Some countries are legally required to monitor marine mammals inhabiting their national waters, such as the US with the Marine Mammal Protection Act 1972 [8], and Australia with the Environment Protection and Biodiversity Act 1999 [9]. Whale abundance and trends are monitored to assess their status and recovery from commercial whaling and other anthropogenic threats (*e.g.* ship strike, entanglement in fishing gear, noise pollution) [10], [11], [12].

Research using VHR satellite images to monitor cetaceans has increased since Abileah (2002) [13] and Fretwell et al. (2014) [14] pioneering studies, highlighting how VHR satellite imagery may help gather missing information about whales, and complement boat and aircraft surveys [3,[15], [16], [17], [18], [19], [20], [21], [22], [23]. There have been developments in using this technology in remote regions to estimate whale density [17], detect strandings [21,^24^,25], and count cetaceans [18]. Each study highlights the challenges that need addressing and the further work required but agree on the opportunity this technology offers for monitoring whales in remote regions.

Among the challenges to scale this technology to its full potential, is the need to analyze the imagery efficiently using automated systems, with machine learning approaches being presented as most suitable for wildlife [15,[26], [27], [28]. In machine learning, models are trained to recognize and classify visual objects through an iterative process, where many examples of the target object are fed into model training [29,30]. Machine learning models require a large annotated dataset of the target species and sometimes confounding features to train and test the algorithms. Initially, these datasets need to be created by humans manually annotating imagery, until automated or semi-automated systems can accurately identify the target feature. Such datasets, openly accessible, are few, with Cubaynes and Fretwell (2022)[31] dataset, which include point, and bounding box annotations, and image chips; and Charry et al. (2021)[18] dataset, which include point annotations. Ideally, the creation of such a dataset would be a collaborative innovative effort using similar protocols and data formats [31].

Our aim is to share a detailed step-by-step workflow for annotating VHR satellite images and for creating datasets of annotations as points, bounding boxes, and image chips in a png format, which will facilitate collaboration across research groups towards the development of an operational system for marine animal detection in VHR satellite imagery. Here we provide a general outline of the steps required to annotate satellite images, and create datasets, alongside detailed protocols for ESRI ArcMap 10.8 (Supplemental 1) [32] and ESRI ArcGIS Pro 2.5 (Supplemental 2) [33], as used by several studies detecting wildlife in VHR satellite imagery [3,^17^,^19^,^26^,31] but with more details to allow reproducibility and transferability. We use cetaceans as a case study to explain the steps, which are transferable to other objects that can be individually labelled in VHR optical satellite imagery. We also provide guidance on ways to differentiate species of cetaceans in VHR satellite image (Supplementary material 3), as well as assessing the certainty of the detection (Supplementary material 5).

## Method details

### Step 1: Image acquisition

**Fig. 1:**
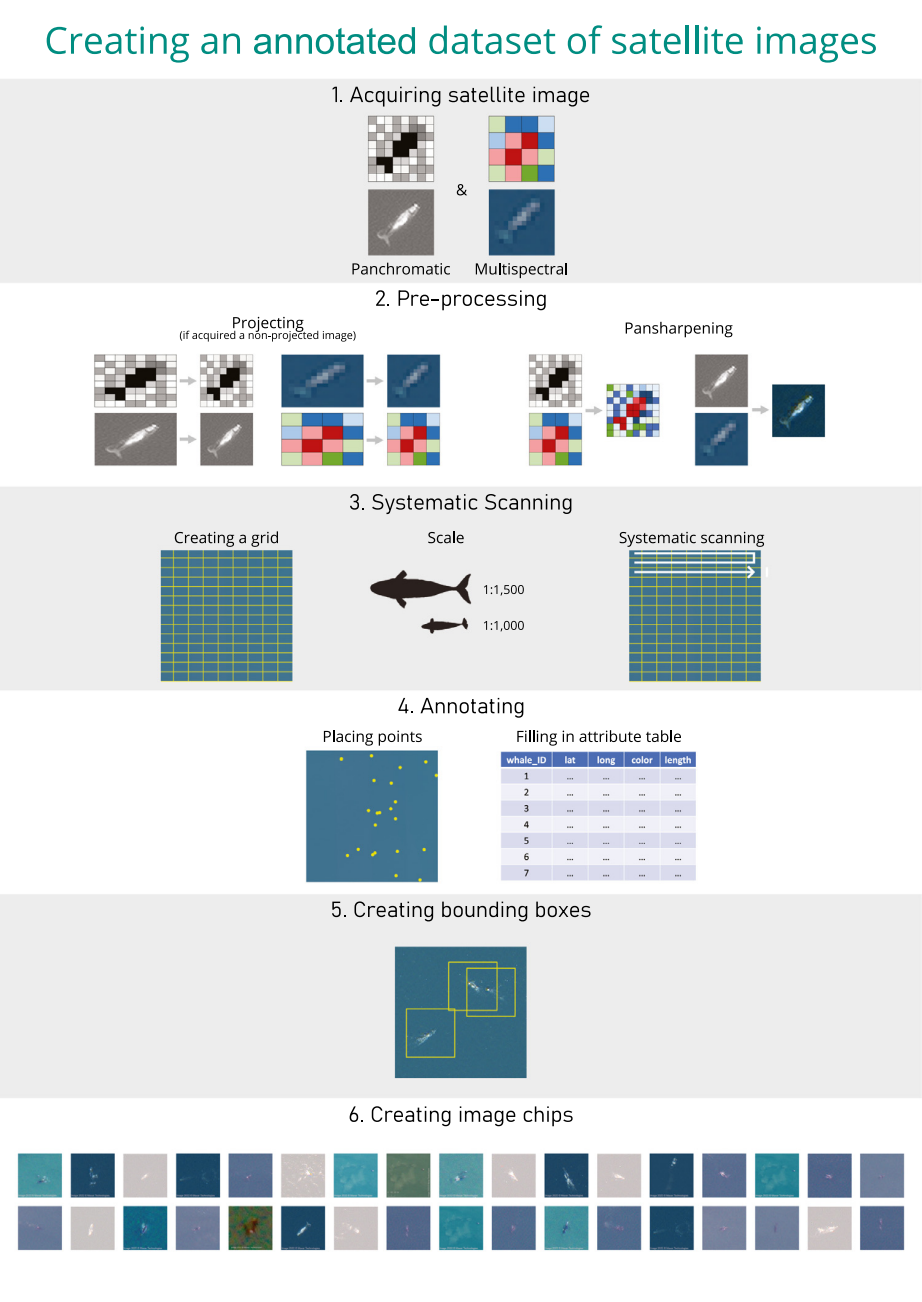
Workflow highlighting the main steps needed to build a dataset of annotated whales from satellite images.

The first step to detecting or counting whales in VHR satellite imagery is to acquire the image (step 1 of Fig. 1). Images can be delivered in different formats. Most VHR satellites capture a panchromatic image (one band, greyscale image, highest spatial resolution) and a multispectral image (multi bands, usually four or eight bands, colored image, lower spatial resolution than the panchromatic image), except for the WorldView-1 satellite, which only captures a panchromatic image.

The main operators of VHR satellites are Airbus, Maxar Technologies, and Planet. Table 1 shows the sensors in orbit for each of these operators, as well as the planned future missions. Due to the commercial nature, VHR satellite imagery is expensive, with discounts available for education and research. We recommend contacting the separate companies to get quotes.

**Table 1:** List of VHR satellites with the company operating them and the type of images available. The spatial resolution for each satellite refers to the panchromatic spatial resolution, which is higher than the multispectral image.

Satellite operator	Satellite	Spatial resolution	Type of image acquisition
Airbus	Pleaides (two satellites)	0.5 m	Archive and tasked
	Pleiades Neo (constellation of four satellites)	0.3 m	Archive and tasked
Maxar Technologies	GeoEye-1 (one satellite)	0.41 m	Archive and tasked
	Ikonos-2 (one satellite)	0.82 m	Archive
	Quickbird-2 (one satellite)	0.65	Archive
	Wolrdview-1 (one satellite)	0.50 m	Archive and tasked
	WorldView-2 (one satellite)	0.46 m	Archive and tasked
	WorldView-3 (one satellite)	0.31 m	Archive and tasked
	WorldView-4 (one satellite)	0.31 m	Archive
	WorldView-Legion (constellation of six satellites)	0.31 m	No images available yet, launch anticipated for 2023
Planet	Skysat (constellation of 21 satellites)	0.5 m	Archive and tasked
	Pelican (constellation of up to 32 satellites)	0.3 m	No images available yet, launch planned for 2023

VHR satellites do not continuously capture images; they attempt to collect imagery over target locations when tasked to do so. The success of tasking satellite image acquisition is influenced by the satellite schedule, cloud cover, and competing priorities. Once images have been acquired, the images then get added to the archive where they are available for anyone to purchase. Purchasing archival imagery is more affordable than requesting a custom tasking of image collection for a specific time and location.

### Step 2: Pre-processing

Before annotating an image, there are a few pre-processing steps that may be needed depending on the type of product acquired (step 2 of Fig. 1). The type of product varies between satellites, operators, but tend to be a variation on whether images are projected, or pansharpened (Table 2). Other pre-processing, such as correcting for the top of atmosphere may be needed depending on the survey goals.

**Table 2:** List of product type for the main VHR satellite imagery providers, Airbus [34], Planet [35] and Maxar Technologies [36].

Satellite operator	Product name	Mapping projection
Airbus	Primary	Coordinate Reference System: WGS84 Map projection: None
	Projected	Coordinate Reference System: WGS84 Map projection: UTM
	Ortho	Coordinate Reference System: WGS84 Map projection: UTM
Maxar Technologies	System-Ready (Basic) 1B System-Ready Stereo (Basic) 1B	Coordinate Reference System: WGS84 Map projection: None
	View-Ready (Standard) OR2A View-Ready Stereo (Standard) OR2A	Coordinate Reference System: WGS84 Map projection: UTM
	View-Ready (Standard) 2A	Coordinate Reference System: WGS84 Map projection: UTM
	Map-Ready (Ortho) 1:12,000	Coordinate Reference System: WGS84 Map projection: UTM
Planet	SkySat Basic Scene	Coordinate Reference System: WGS84 Map projection: None
	SkySat Ortho Scene	Coordinate Reference System: WGS84 Map projection: UTM
	SkySat Ortho Collect	Coordinate Reference System: WGS84 Map projection: UTM

#### Projection

**Fig. 2:**
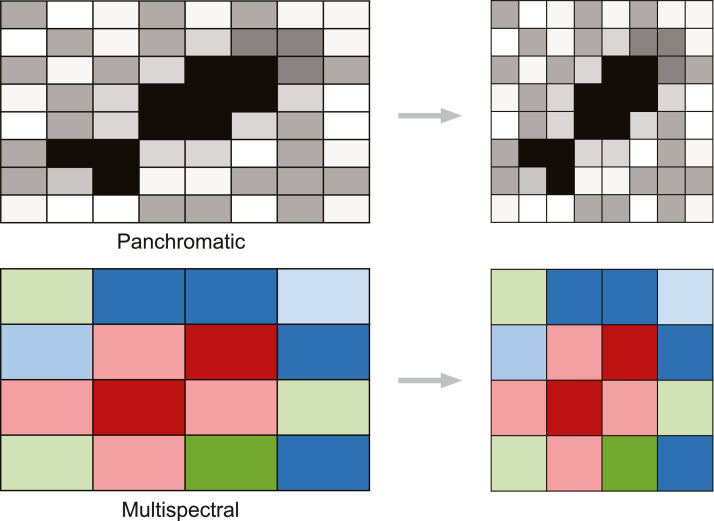
Projecting a panchromatic (top) and multispectral (bottom) satellite images.

Projection is the process of mathematically transforming the coordinate system from a sphere to a flat surface. Several coordinate systems exists with some better suited to represent data for different geographic locations. When a satellite captures an image of the Earth surface, it will show some distortions, as the image is a flat surface and the Earth a sphere. This distortion needs to be corrected by assigning the appropriate coordinate system to the image (Fig. 2). If the imagery acquired is not already projected in WGS 1984 with the relevant UTM zone, projecting the image is required before annotation.

#### Pansharpening

**Fig. 3:**
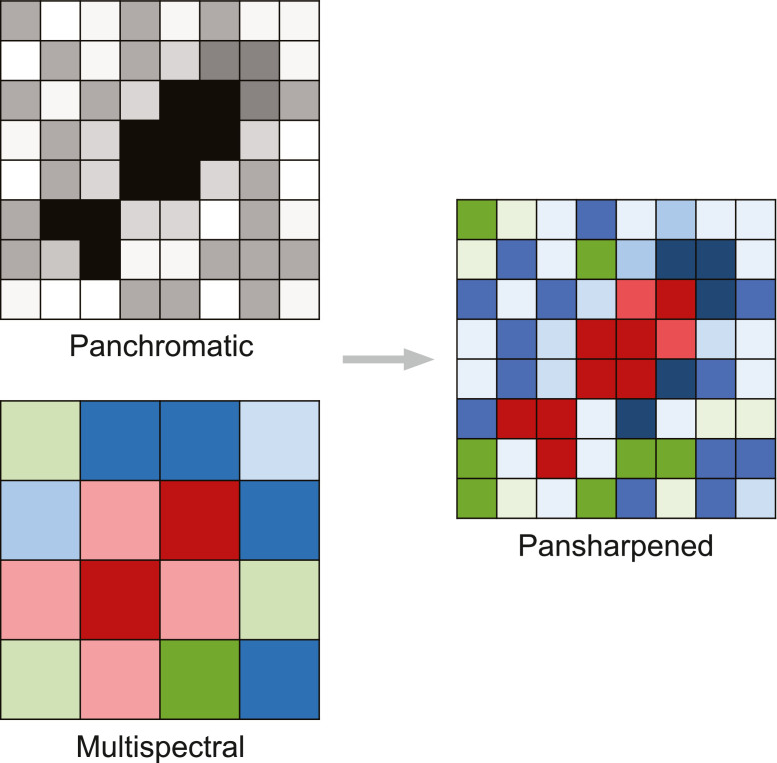
Process of pansharpening.

Pansharpening is the process by which the pixels of the panchromatic image are combined with the pixels of a multispectral image, to produce a new image with the high spatial resolution of the panchromatic image and with the additional color information from the multispectral image (Fig. 3). We highly recommend this step for manually annotating VHR satellite images, as it improves the ability to discriminate objects in the image. Using only the panchromatic image is possible but the color adds confidence in detection. Images that have already been pansharpened can be acquired from the imagery provider. Detailed pansharpening protocols are outlined in Supplementary material 1 for ESRI ArcMap 10.8 and Supplementary material 2 for ESRI ArcGIS Pro 2.5.

#### Atmospheric correction

If the aim of the project is to compare the spectral reflectance of whales between different images, then the images need to be corrected for atmospheric effects. Atmospheric correction removes atmospheric effects, such as scattering and absorption from gas and aerosols present in the atmosphere, this is dependent upon the composition of the atmosphere and the geometry of the collected parameters of the data. Two types of atmospheric corrections exist to obtain spectral reflectance, top-of-atmosphere, and bottom-of-atmosphere [37].

Top-of-atmosphere correction requires parameters based upon the mean solar spectral irradiance, solar zenith angle, and spectral radiance at the sensor's aperture. These are available from the imagery metadata and can almost always be applied to VHR satellite imagery [38], [39], [40]. The bottom-of-atmosphere (sometimes referred to as full atmospheric correction) will give the spectral reflectance of the feature as it would be if measured at the surface of the earth. It will allow true comparison of the spectral of pixels between different satellite images, taken at different times with different atmospheric conditions. However, this full atmospheric correction requires knowing the accurate composition in gas and aerosols of the atmosphere at a given time. This is difficult to estimate accurately, as it varies among regions, days and time of day, requiring *in situ* measurements, or use of atmospheric composition models accurate for the location studied [37]. These are rarely available at field sites. Therefore, when comparing whale spectra between images, at minimum the top-of-atmosphere correction should be applied. This can be done in ENVI, similar to Cubaynes et al. (2019) [3], or other available software. In ArcGIS Pro, the Apparent Reflectance function allows to correct the top-of-atmosphere for the following VHR satellites: IKONOS, QuickBird, GeoEye-1, RapidEye, DMCii, WorldView-1, WorldView-2, SPOT 6, and Pleiades [41].

### Step 3: Systematic scanning

To ensure that the whole image is reviewed for the presence of cetaceans, systematic scanning is necessary (step 3 of Fig. 1). A grid needs to be overlayed on top of the satellite image to review it in a systematic pattern from the top to the bottom of the image, scanning left to right, then right to left, etc. We recommend reviewing the image at a scale of 1:1500 for large cetaceans (animals between 9 and 20 m long) and zooming in as needed. For the larger whale species (above 25 m long) such as fin whales (*Balaenoptera physalus*) and blue whales (*Balaenoptera musculus*) a scale of 1:2000 is sufficient, and for smaller cetaceans (less than 9 m long) we recommend using a scale of 1:1250 [3,^17^,18]. As some images can cover a large area (more than 500km^2^), it could take days to review it fully; therefore, we recommend keeping track of the grid cells that have been reviewed by following the steps outlined in Supplementary material l 1 for ESRI ArcMap 10.8 and Supplementary material 2 for ESRI ArcGIS Pro 2.5.

### Step 4: Annotating

**Fig. 4:**
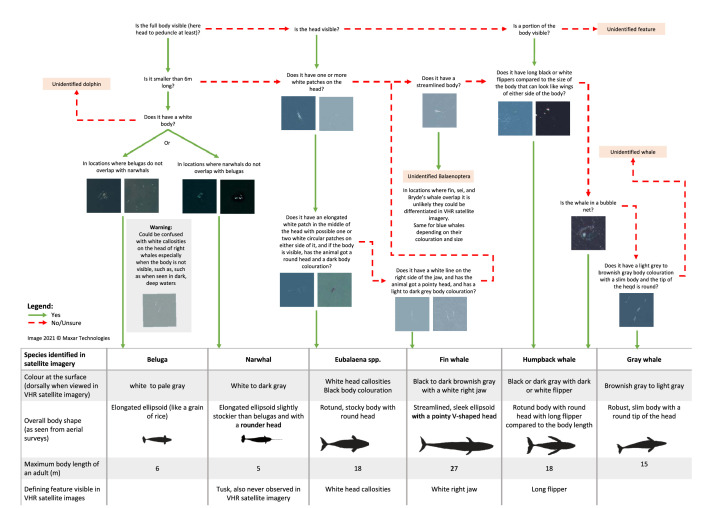
Species decision tree for cetaceans previously observed in VHR satellite imagery.

Annotating consists of labeling your imagery by placing points or bounding boxes on the object of interest, in this case whales (step 4 of Fig. 1) and filling in the relevant information needed for your machine learning model, such as the species name (Table 3). In ESRI ArcMap and ESRI ArcGIS Pro, points can be stored in a shapefile, which retains the coordinate information of the points, alongside any associated metadata. An important aspect of annotating is assessing the confidence in the detection of the target object. We have built a workflow to help assess species identification (Fig. 4.; see Supplementary material 3 for more details) and assign a certainty level (see Supplementary material 5). Detailed instructions to annotate VHR satellite images are outlined in Supplemental 1 for ESRI ArcMap 10.8 and Supplementary material 2 for ESRI ArcGIS Pro 2.5.

**Table 3:** List of fields recommended to include in the attribute table for annotating cetaceans in VHR satellite images, although these may vary with project goals.

**Field**	**Description**
Observer	Name of person reviewing the image.
location	Name of the location where the satellite image was captured.
Satellite	Name of the satellite that captured the image.
Ground sampling distance	The ground sampling distance (the distance between the center points of each pixel), which can be found in the metadata, by right clicking on the panchromatic file and selecting “Properties”, then “Source” and “Raster Information”.
Image id	Unique identification that the satellite imagery provider assigns to each image. With Maxar, this corresponds to the catalog ID.
Image date	Date the image was captured.
Image time	Time the image was captured.
Product type	The product type indicates the level of pre-processing an image has gone through when it was acquired from the satellite imagery provider, such as projection. See Table 1 for the various product type offered by the main VHR satellite imagery providers.
Sea state	Sea state adapted from Fig. 4 in Bamford et al. [17] **1= Good** (minimal swell, no white caps, no wavelets) **2 = Moderate** (minimal swell, sparse white caps, few wavelets) **3 = Average** (slight swell, intermittent wavelets, no or very few white caps) **4 = Sub-average** (medium swell, apparent waves, several white caps). **5 = Poor** (significant swell, directional surface wind, large wave, several white caps)
Cloud coverage	Cloud cover for the whole image, using the aviation system: 0 = SKC (sky clear) 1-2 = FEW (traces) 3-4 = SCT (scattered) 5-7 = BKN (Broken) 8 = OVC (Overcast)
Cloud thickness	Cloud thickness for the clouds present in the image 1 = Thin (can see fairly well through the cloud) 2 = Medium thin (can see through but no clear view of the sea) 3 = Thick (can't see through) 4 = mix of thin, medium, thick clouds
Glare	Proportion of glare in the whole image: 0 = None 1= Mild 2 = Moderate 3 = Severe
Turbidity	Qualitative estimations of the level of turbidity: 1 = Non-turbid 2 = Moderate 3 = Turbid 4 = mix of turbid and non-turbid waters
Other environment	Other environmental conditions that the observer thinks might limit the visibility of whales (*e.g.* dark image for polar regions from autumn to spring)
Latitude	Latitude of the whale detection
Longitude	Longitude of the whale detection
Geographical coordinate system	Geographical coordinate system, it can be found in the metadata
Projection	Projection applied to the image to remove distortion
Species code	Species code for the species or the next higher taxonomic level, see Supplementary material 3 to help you decide, and Supplementary material 4 for the code to use
Certainty	Certainty of the assignment of the species or the next higher taxonomic level. See Supplementary material 5 to help you decide. 1 = **Definite**: you are confident in your species determination (90-100% confidence) 2 = **Probable**: you think that your species determination is likely but you are not sure (60-90% confidence) 3 = **Possible**: you think that your species determination is possible but it is hard to tell (10-60% confidence)
Body color	Body color of the whale when at the surface (dorsally when viewed in VHR satellite imagery)
Body shape	Overall shape of the body excluding fluke and flippers
Body length	Maximum visible length between the tip of the head and the fluke with values ranging from calf size to maximum adult length
Body width	Body width, it is measured at the widest part of the body and perpendicular the body length
Flipper	Forelimb used to stabilize and turn 1 = Yes 2 = No 3 = Maybe
Long flipper	Species specific – Humpback whale have long flippers, which are one third of the body length 1 = Yes 2 = No 3 = Maybe
Fluke	Tail used to generate thrust 1 = Yes 2 = No 3 = Maybe
Head callosities	Species specific – white head callosities for the species of the genus Eubalaena. White patches on top of the head 1 = Yes 2 = No 3 = Maybe
White lower jaw	Species specific – white right lower jaw for fin whales 1 = Yes 2 = No 3 = Maybe
After breach	Large white area left after a whale breached, or lobtailed, flipper-slapped 1 = Yes 2 = No 3 = Maybe
Bubble net	Species specific – bubble net for humpback whales. One white spiral formed of several white circular patches, or several white spirals nested together 1 = Yes 2 = No 3 = Maybe
Contour	White line separating the part of the whale body that is above and below the sea surface (*e.g.*, when a whale is rolling its back or surfacing to breathe) 1 = Yes 2 = No 3 = Maybe
Flukeprint	White circle left after whale dove or while swimming [42] 1 = Yes 2 = No 3 = Maybe
Wake	V-shaped white trail behind the animal 1 = Yes 2 = No 3 = Maybe
Blow	Vaporous whitish patch next to a whale, like fog 1 = Yes 2 = No 3 = Maybe
Mudtrail	Plume/cloud of substrate behind a whale 1 = Yes 2 = No 3 = Maybe
Surface active group	Two or more whales rolling and touching at the surface 1 = Yes 2 = No 3 = Maybe
Travel group	Two or more cetaceans traveling together in the same direction and less than a few meters apart 1 = Yes 2 = No 3 = Maybe
Mother-calf pair	Mother-calf pair, observed when the calf is next to the mother. 1 = Yes 2 = No 3 = Maybe
Other group	Other type of group, if not socializing or traveling. 1 = Yes 2 = No 3 = Maybe
Defecation	1 = Yes 2 = No 3 = Maybe
Comment	Any other comment the observer would like to make about the specific detection

### Step 5: Creating bounding boxes

Although point shapefiles of annotated cetaceans may be useful to automate detection, particularly for approaches utilizing spectral signatures, bounding boxes are often desired for training machine learning models [15,26]. Similar to Cubaynes and Fretwell (2022)[31], these boxes can be created from the point shapefile incorporating the metadata from the attribute table, so each bounding box has a set of specific information attached to it, necessary for automation (step 5 of Fig. 1). We recommend making the bounding box at least twice the size of the known adult size for the species of interest.

### Step 6: Creating image chips

Image chips can be created by using the bounding boxes to clip the satellite image into several image chips that contain cetaceans (see details in Supplementary material 1 for ESRI ArcMap 10.8 and Supplementary material 2 for ESRI ArcGIS Pro 2.5; step 6 of Fig. 1). VHR satellite images have limited distribution due to licensing restrictions. Some licenses, such as the group license with Maxar Technologies permits the sharing of subsets of the images as a png or jpeg format (with reduced spectral resolution and lacking spatial reference, and reduced spectral resolution)[31]. Therefore, it is important to verify with the satellite imagery provider what can be shared (*e.g.* format, subset or whole image) and with whom (under certain licenses sharing the raw images with collaborators is feasible).

## Methods validation

The workflow for ESRI ArcMap 10.8 was developed and used by several studies [3,^17^,^19^,31] with updates for ArcMap 10.8. None of these studies offered a step-by-step guide. The workflow for ESRI ArcGIS Pro 2.5 was adapted from the ArcMap workflow.

## Ethics statements

This method does not involve work with human subjects, nor animal experiments, nor data collected from social media platforms.

## Funding

This work was supported by the 10.13039/100005199Marine Mammal Commission (project MMC21-043). This study represents a contribution of the Ecosystems component of the British Antarctic Survey, funded by the Natural Environment Research council (NERC). This work also represents a contribution of the Geospatial Artificial Intelligence for Animals (GAIA) project.

## CRediT authorship contribution statement

**Hannah Charlotte Cubaynes:** Conceptualization, Methodology, Writing – original draft, Visualization, Funding acquisition. **Penny Joanna Clarke:** Validation, Writing – review & editing, Resources. **Kimberly Thea Goetz:** Validation, Writing – review & editing, Resources, Funding acquisition. **Tyler Aldrich:** Validation, Writing – review & editing, Resources. **Peter Thomas Fretwell:** Validation, Writing – review & editing, Resources. **Kathleen Elise Leonard:** Validation, Writing – review & editing, Resources. **Christin Brangwynne Khan:** Validation, Writing – review & editing, Resources, Funding acquisition.

## Declaration of Competing Interest

The authors declare that they have no known competing financial interests or personal relationships that could have appeared to influence the work reported in this paper.

## Data Availability

No data was used for the research described in the article.

## Appendix Supplementary materials

Supplementary materiel 1 A detailed step-by-step workflow to annotate whales in VHR satellite images and create points, bounding boxes, and image chips data sets using ESRI ArcMap 10.8. Image, application 1

Supplementary materiel 2 A detailed step-by-step workflow to annotate whales in VHR satellite images and create points, bounding boxes, and image chips data sets using ESRI ArcGIS Pro 2.5. Image, application 2

Supplementary materiel 3 Species decision tree for cetaceans observed in VHR satellite imagery. Image, application 3

Supplementary materiel 4 Species code. Image, application 4

Supplementary materiel 5 Assigning a certainty level to whale observed in VHR satellite imagery. Image, application 5

Supplementary materiel 6 Shapefile containing the template attribute table to be used with Supplementary materials 1 and 2. Image, application 6
