# Supplementary material for: Annotating very high-resolution satellite imagery: A whale case study
Source: MethodsX. 2023 Jan 25;10:102040. doi: 10.1016/j.mex.2023.102040 (PMC9923222; doi:10.1016/j.mex.2023.102040)
Supplement: Supplementary materiel 1 — A detailed step-by-step workflow to annotate whales in VHR satellite images and create points, bounding boxes, and image chips data sets using ESRI ArcMap 10.8. [file mmc1.zip › Supplementary1_ArcMAP.docx]

Supplementary material 1: User Guide to Annotating Cetaceans in VHR Satellite Imagery

Guidance for ArcMap 10.8.1

Hannah Cubaynes^1^ (Editor)

**Contributing Authors:**

Penny Clarke^1,2^, Christin Khan^3^, Kim Goetz^4^, Kathleen Leonard^4^, Peter Fretwell^1^, Daniel Cholewiak^3^, Tyler Aldrich^3^

**Affiliations:**

^1^British Antarctic Survey, High Cross, Madingley Road, Cambridge, CB3 0ET, United Kingdom

^2^School of Engineering, The University of Edinburgh, Sanderson Building, Robert Stevenson Road, The King’s Buildings, Edinburgh, EH9 3FB, United Kingdom

^3^Northeast Fisheries Science Center, National Marine Fisheries Service, NOAA, Woods Hole, Massachusetts, United States

^4^Marine Mammal Laboratory, Alaska Fisheries Science Center, National Marine Fisheries Service, NOAA, Seattle, Washington, United States

Contents

[List of Tables iv](#_Toc118288792)

[Key Terminology v](#_Toc118288793)

[Summary 1](#_Toc118288794)

[Setting Up 2](#_Toc118288795)

[Pre-processing 5](#_Toc118288796)

[Projection 5](#_Toc118288797)

[Top of atmosphere correction 6](#_Toc118288798)

[Pansharpening 6](#_Toc118288799)

[Preparing the Workspace 13](#_Toc118288800)

[Creating a grid 13](#_Toc118288801)

[Creating a point shapefile 24](#_Toc118288802)

[Preparing the attribute table 29](#_Toc118288803)

[Reviewing the Image 38](#_Toc118288804)

[Systematic scanning 38](#_Toc118288805)

[Scale 39](#_Toc118288806)

[Keeping track 39](#_Toc118288807)

[Annotating 43](#_Toc118288808)

[Placing points on top of whales 43](#_Toc118288809)

[Filling in the attribute table 49](#_Toc118288810)

[Joining annotations from multiple observers 70](#_Toc118288811)

[Creating bounding box 70](#_Toc118288812)

[Centre the points in a middle of a pixel 70](#_Toc118288813)

[Create a buffer around the centered points 80](#_Toc118288814)

[Create a bounding box around the buffer 89](#_Toc118288815)

[Creating image chips (PNG) 93](#_Toc118288816)

[Clipping the satellite image to the outline of the bounding boxes 93](#_Toc118288817)

[Exporting the clipped tif file as a png file 99](#_Toc118288818)

[Create multiple image chips 102](#_Toc118288819)

[Acknowledgments 106](#_Toc118288820)

[References 107](#_Toc118288821)

[Appendix 1: Species decision tree for cetaceans observed in VHR satellite imagery 109](#_Toc118288822)

[Appendix 2: Species code 111](#_Toc118288823)

[Appendix 3: Assigning a certainty level 112](#_Toc118288824)

# List of Tables

[**Table 1.** List of product type for the main VHR satellite imagery provider, Airbus (Airbus, 2022), Planet (Planet, 2022) and Maxar Technologies (Maxar Technologies, 2022). 5](#_Toc113271607)

[**Table 2.** Information necessary to fill the “Add Field” window, each row represents a different Field. 34](#_Toc113271608)

[**Table 3.** Description of each Field contained in the attribute table with information on how to fill in the information for each Field. 55](#_Toc113271609)

[**Table 4.** Information necessary to fill the “Add Field” window, each row represents a different Field. 89](#_Toc113271610)

# Key Terminology

**Annotation:** the process of categorizing and labelling objects of interest (*e.g.* whales) within the satellite image either by placing points or bounding boxes along with associated metadata.

**Multispectral**: describe a colored image, where each pixels comprised of different values representing different color bands (*e.g.* red, green, and blue). Most very high-resolution (VHR) satellite images capture multispectral images with four (blur, green, red, near-infrared) or eight bands (coastal blue, blue, green, yellow, red-edge, red, near-infrared 1, near-infrared 2). For VHR satellites, the spatial resolution is lower for the multispectral image compared to the panchromatic image.

**Panchromatic**: describe a grayscale image, where each pixels comprised of one value. For very high-resolution satellites, the spatial resolution is higher for the panchromatic image compared to the multispectral image

**Pansharpened**: a pansharpened image is created by combining the high spectral resolution (color) of a multispectral image with the high spatial resolution of a panchromatic image, resulting in an image that is both high resolution and color.

**Pansharpening**: the process of combining the high spectral resolution (color) multispectral image with the high spatial resolution panchromatic image, to create a pansharpened image of both high resolution and color. Various algorithms exist to execute this transformation (*e.g.* Brovey, Gram-Schmidt).

# Summary

In this document, we share a step-by-step guide and our recommendations for annotating satellite images with the ultimate goal to build a dataset of examples of whales in satellite images using ArcMap 10.8.

#

# Setting Up

1. Open ArcMap.
2. If annotating a new satellite image: close the “ArcMap – Getting Started” window (then go to step 4).
3. If returning to continue annotating a satellite image, in the window “ArcMap – Getting Started” select the map you previously saved and already worked on, and pick up where you left off.
4. Save the map.
   1. In the “Untitled – ArcMap” window select “File”.
   2. Then select “Save As”.
   3. A new window opens, choose a file name, there is only one file type available (“ArcMap Document”), and select “Save”.
5. Load the multispectral and panchromatic .TIL files, or the pansharpened file if available.
   1. **Option 1**: from ArcCatalog.
      1. Open ArcCatalog, and navigate to where your satellite image is saved, and drag and drop into ArcMap.
   2. **Option 2**: from ArcMap
      1. Select the “Add Data” icon
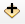
(circled in red in the image below).


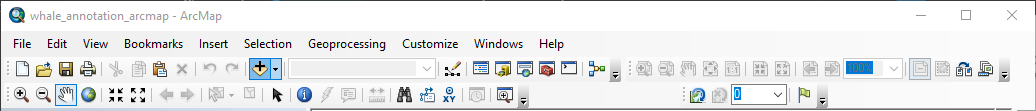


- - 1. The “Add Data” window opens, select the “Connect To Folder” icon
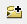
(circled in red in the image below).


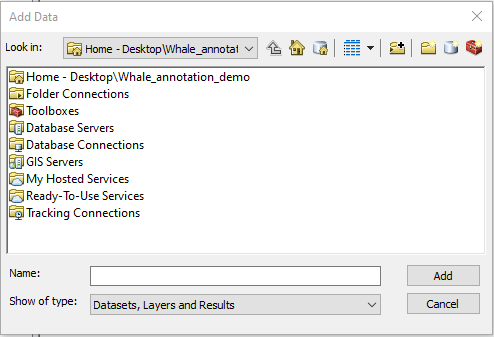


- - 1. The “Connect To Folder” window opens, select the folder you wish to connect to (it can be as general as “This PC” or as specific as the exact folder where you will save all the work related to this map).
    2. Once you select the folder you wish to connect to, it will bring you back to the “Add Data” window, under “Look in:” select “Folder Connections” (see red rectangle).


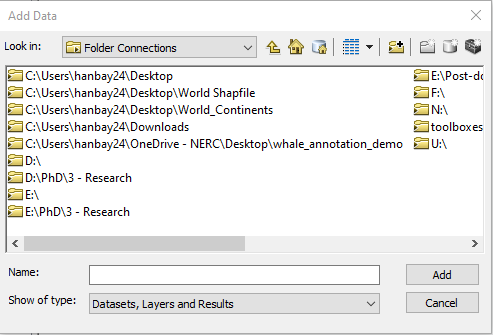


- - 1. Select the folder where your satellite images are saved.
       1. If adding the multispectral and panchromatic files, you will need to add them separately (you can start with either)
          1. Select the multispectral .TIL file (the .TIL file is a mosaic of all the .TIF files, which is easier to work with when using a large satellite images made of several .TIF files) and select “Add”. If you are asked to compute the pyramids, select “OK”.
          2. Select “Add Data” icon
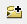
 in the toolbar and repeat the process for the panchromatic image (steps 5.2.4. to 5.2.5.1.1). If you are asked to compute the pyramids, select “OK”.
          3. Your ArcMap window should look similar to the image below. Here we used a satellite image of Witsand, South Africa, where there are several southern right whales.


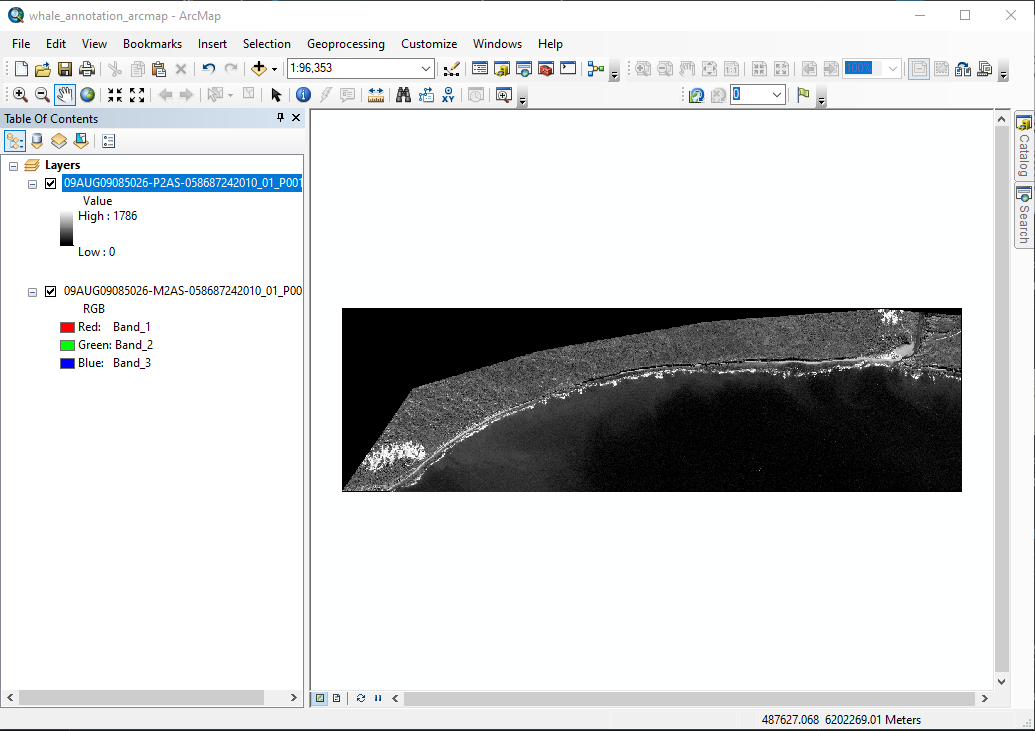


- - - 1. If adding the pansharpened file, select “Add Data” icon
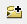
 in the toolbar and repeat steps 5.2.4. to 5.2.5.1.1 for the pansharpened image. If you are asked to compute the pyramids, select “OK”.

## Pre-processing

### Projection

Depending on the imagery provider and product type (Table1), you may need to project the image to WGS 1984 with the relevant UTM zone. To find the relevant UTM Zone to use, refer to this link (<https://www.dmap.co.uk/utmworld.htm>). The Witsand image used as an example here is already projected to WGS 1984 UTM Zone 34S. If the image you wish to annotate is not projected to WGS 1984 with the relevant UTM Zone, use the tool “Project Raster (Data Management)” to project your image, and refer to this link for additional guidance (<https://desktop.arcgis.com/en/arcmap/latest/tools/data-management-toolbox/project-raster.htm>).

**Table 1.** List of product type for the main VHR satellite imagery provider, Airbus (Airbus, 2022), Planet (Planet, 2022) and Maxar Technologies (Maxar Technologies, 2022).

|  | **Product name** | **Mapping projection** |
| --- | --- | --- |
| **Maxar** | System-Ready (Basic) 1B  System-Ready Stereo (Basic) 1B | Un-projected |
|  | View-Ready (Standard) OR2A  View-Ready Stereo (Standard) OR2A | Projected |
|  | View-Ready (Standard) 2A | Projected |
|  | Map-Ready (Ortho) 1:12,000 |  |
| **Airbus** | Primary | Coordinate Reference System: WGS84  Map projection: None |
|  | Projected | Coordinate Reference System: WGS84  Map projection: UTM |
|  | Ortho | Coordinate Reference System: WGS84  Map projection: None |
| **Planet** | SkySat Basic Scene | Coordinate Reference System: WGS84  Map projection: None |
|  | SkySat Ortho Scene | Coordinate Reference System: WGS84  Map projection: UTM |
|  | SkySat Ortho Collect | Coordinate Reference System: WGS84  Map projection: UTM |

### Top of atmosphere correction

If the aim of the project is to extract spectral data from the imagery, it is recommended to correct for the top of atmosphere. This can be done in ENVI similar to Cubaynes *et al.*, 2019, or other software.

### Pansharpening

1. If the image is not already pansharpened, (you are working with a multispectral and a panchromatic files), pansharpen using the following steps. If your image is already pansharpened go to step 6.7.
   1. Under the “Window” tab select “Image Analysis”.


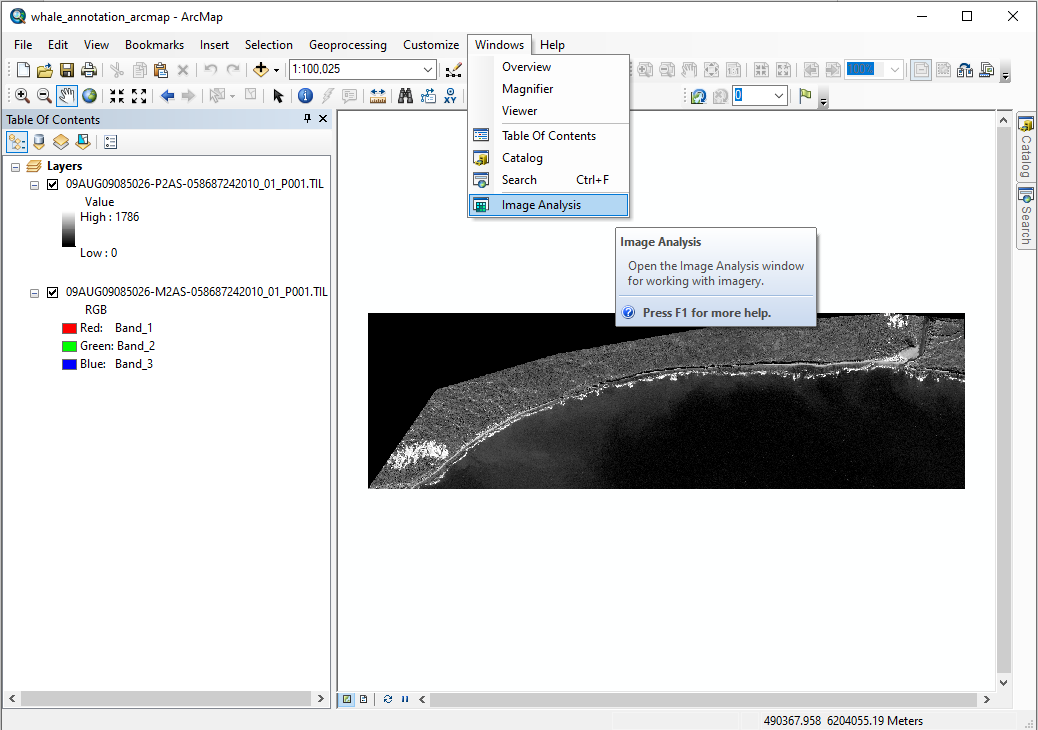


- 1. In the top left corner of the “Image Analysis” window, select the “Options” icon
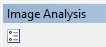
, this will open the “Image Analysis Options” window.
     1. Select the “Pan Sharpen” tab, and select the “Method”


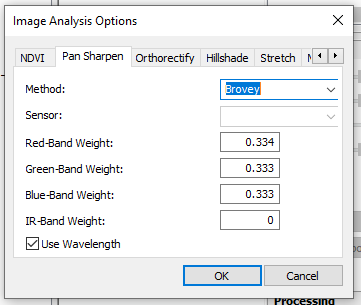


- - 1. We recommend using Brovey or Esri, as they yield the most crisp images but if they don’t work on your image try another method.
    2. Select “OK” to close this window
  1. Back in the “Image Analysis” window, highlight the multispectral and panchromatic files in the top pane.


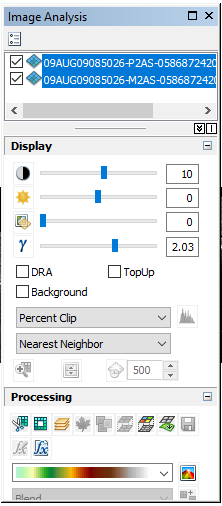


- 1. Under the “Processing” pane, select the pansharpening icon
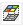
. It may take time to pansharpen.


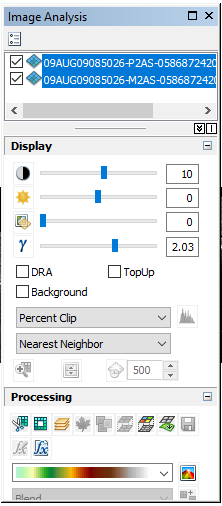


- 1. You will now see a new file under your “Table of Contents”,
  2. Close the “Image Analysis” window.
  3. Update the symbology settings for the newly created pansharpened file:
     1. Right click on the pansharpened file and select “Properties”


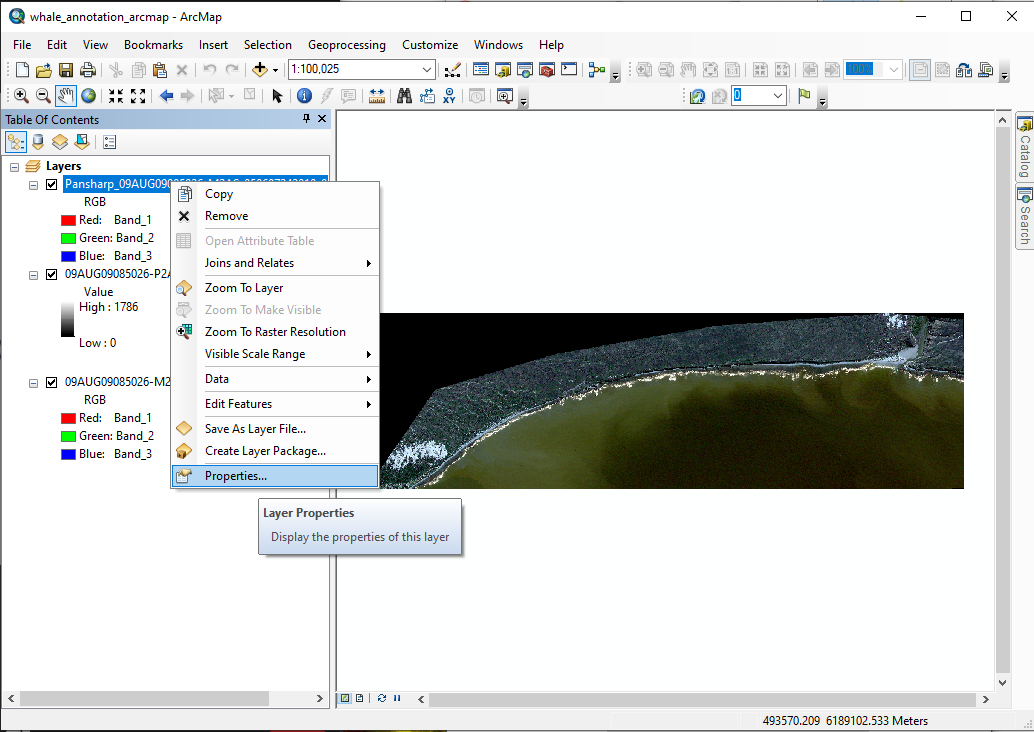


- - 1. A new window will open, called “Layer Properties”, select the “Symbology” tab.
       1. Change the band for the red, green and blue channel. For WorldView-2 to 4, GeoEye-1, band 1 is the blue channel, band 2 is the green channel, band 3 is the red channel and band 4 is the near infrared (NIR) channel. For these satellites, change the band for the red, green, blue channels to the following:


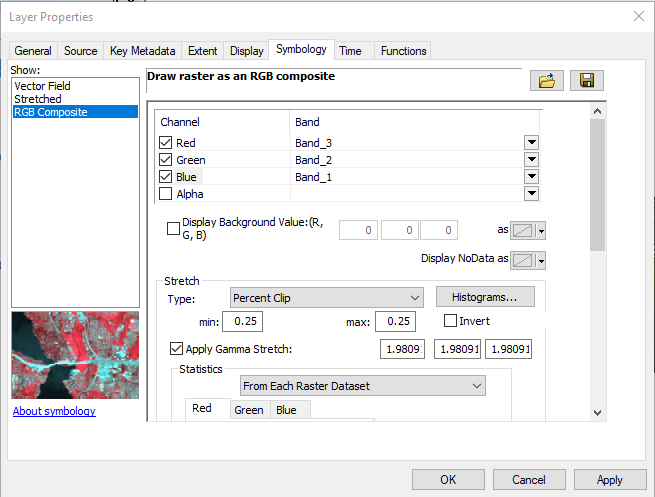


- - - 1. You can also update the stretch, standard deviation is usually a good one but it will vary between images and likely monitor/screen which is the better stretch.


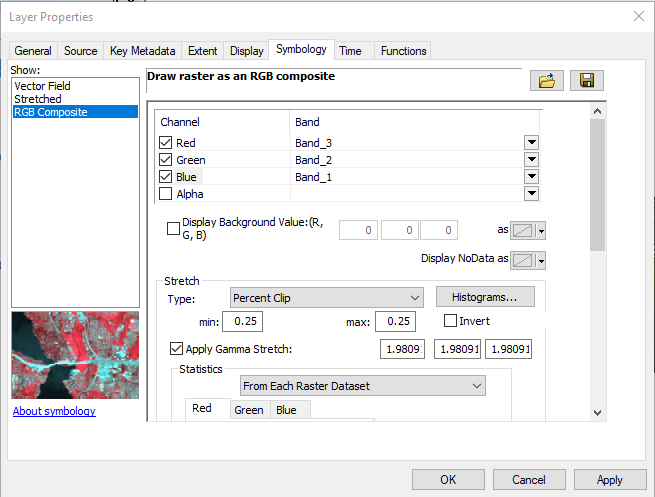


- - 1. Depending on the monitor/screen you are using you might need to update the contrast and brightness, which you can adjust under the “Display” window.


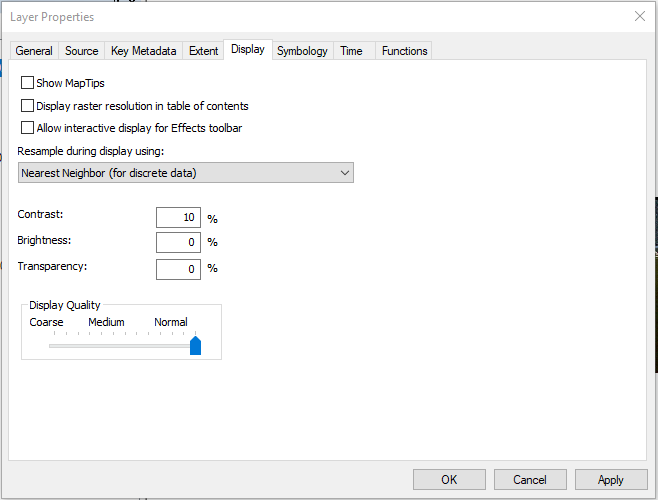


## Preparing the Workspace

### Creating a grid

1. Create a grid that will help review the image. Our recommendation is to review an image at a scale of 1:1,500 for large whales and zooming in when required (for smaller cetaceans, such as belugas use a scale of 1:1,250).
   1. First zoom in to 1:1,500 (or 1:1,250 if searching for smaller cetaceans or to your preferred scale).


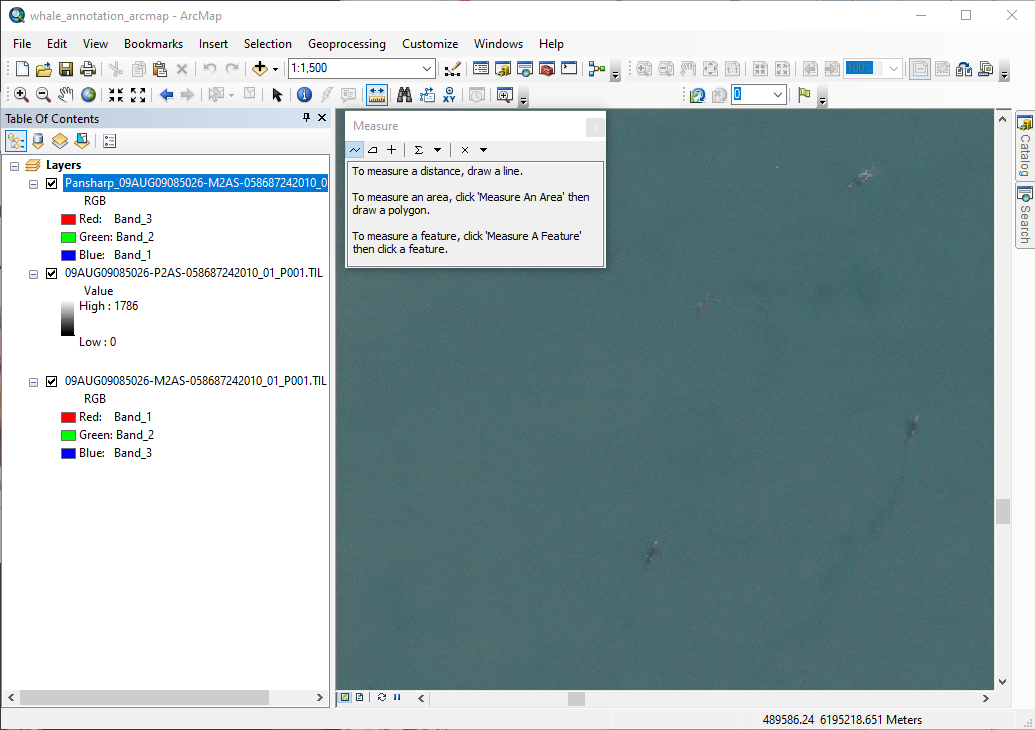


- 1. Then select the measuring tool
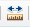
, it will open the “Measure” window.


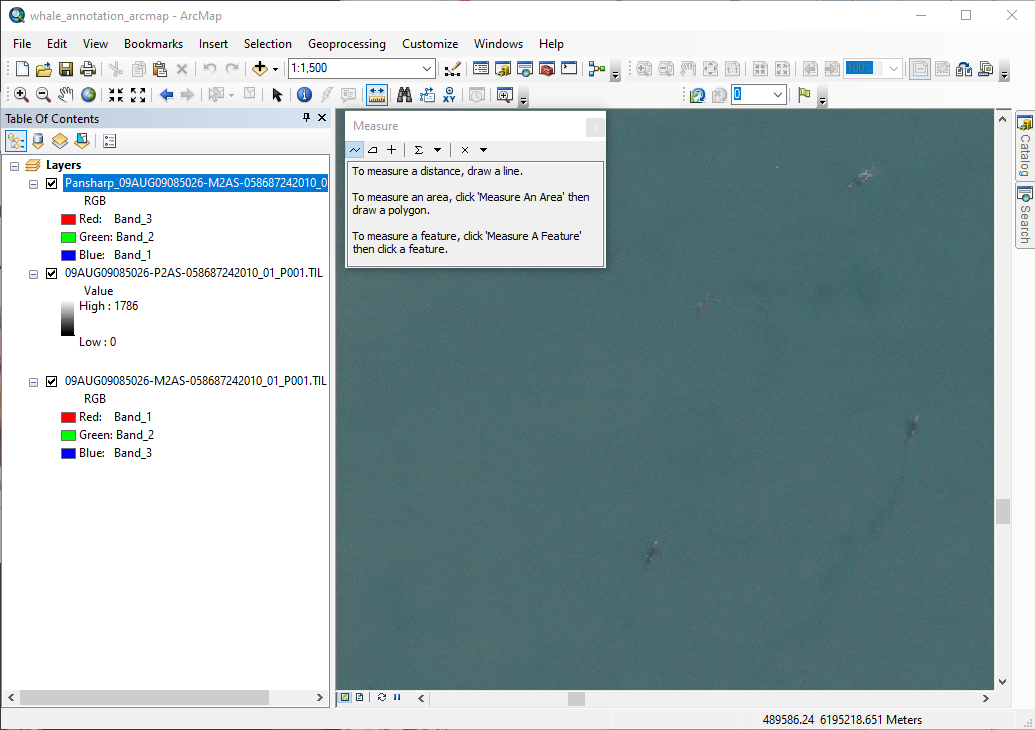


- 1. Ensure you are measuring in meters


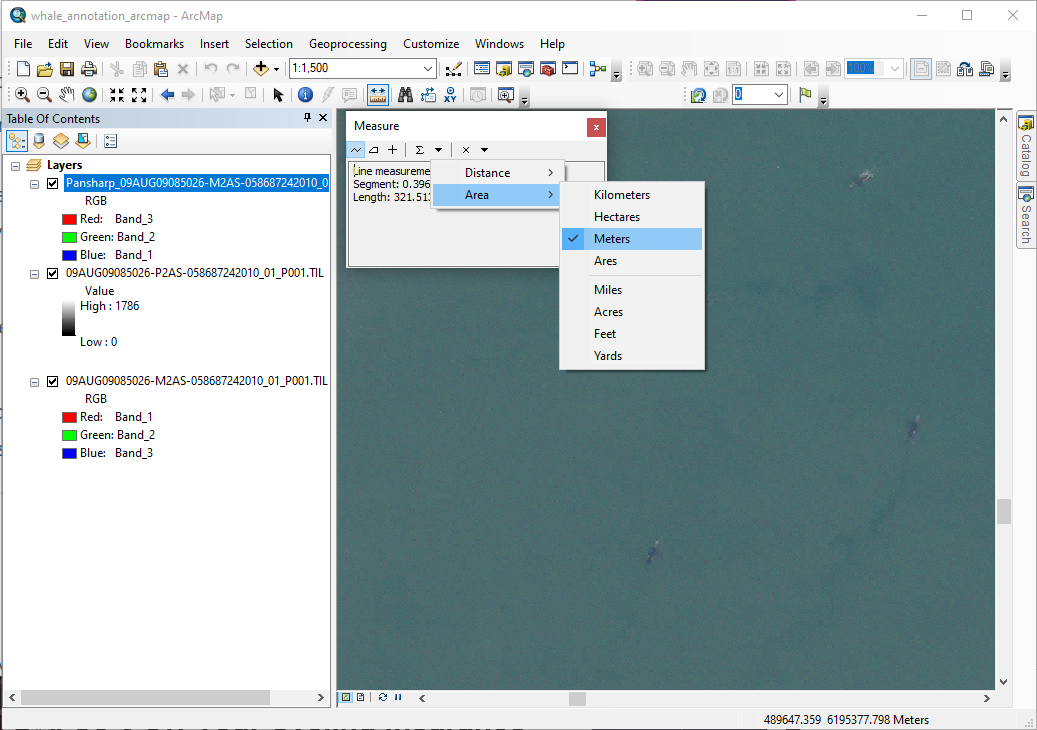


- 1. Measure the height and width of your screen in meters. Write this information on a piece of paper, as you will need it for the next step.


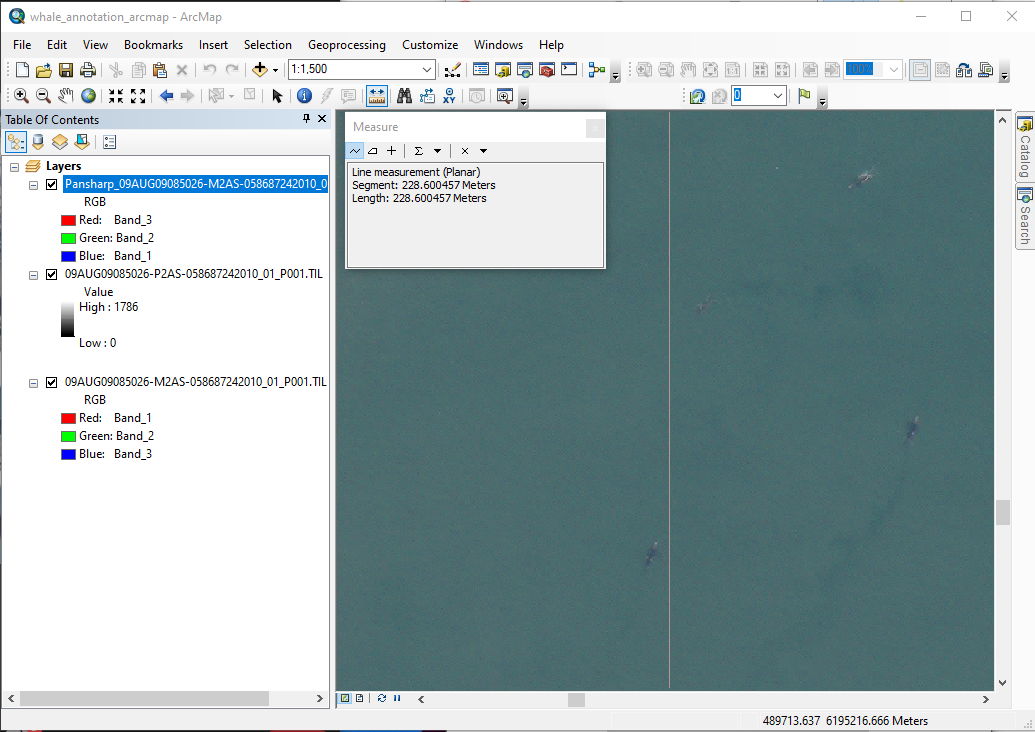


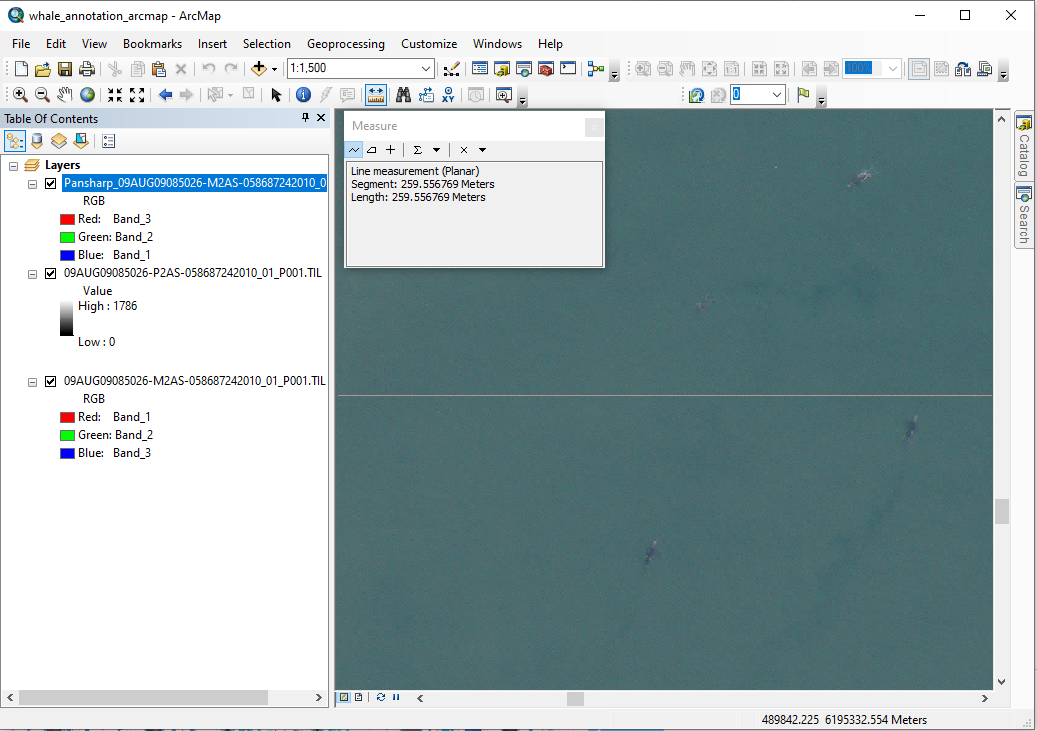


Here we would recommend creating a grid of height 225 m and width 250 m.

- 1. Open the ‘Create Fishnet’ tool either by:
     1. Using the search tool
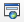
, and type “fishnet” in the search bar and press “Enter”. Select “Create Fishnet (Data Management)”.


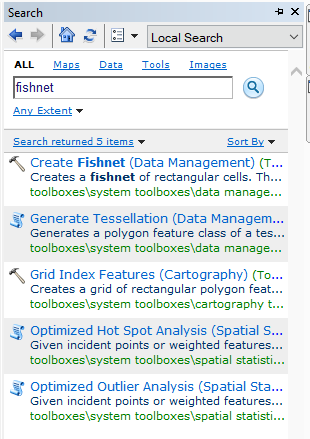


- - 1. Or using the ArcToolbox
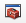
, under “Data Management Tools”, select “Sampling” and then “Create Fishnet”


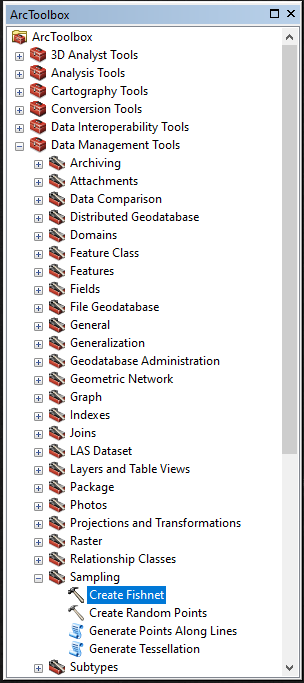


- - 1. The “Create Fishnet” window will open and fill in the following information:
- Output Feature Class: The name you wish to give to your grid, *e.g.* ‘grid_whale_annotation_arcmap’
- Template Extent (optional): select the pansharpened file
- Cell Size Width: the width you recorded at step 7.4.
- Cell Size Height: the height you recorded at step 7.4.
- Untick ‘Create Label points (optional)’
- Geometry Type: ‘Polygon’


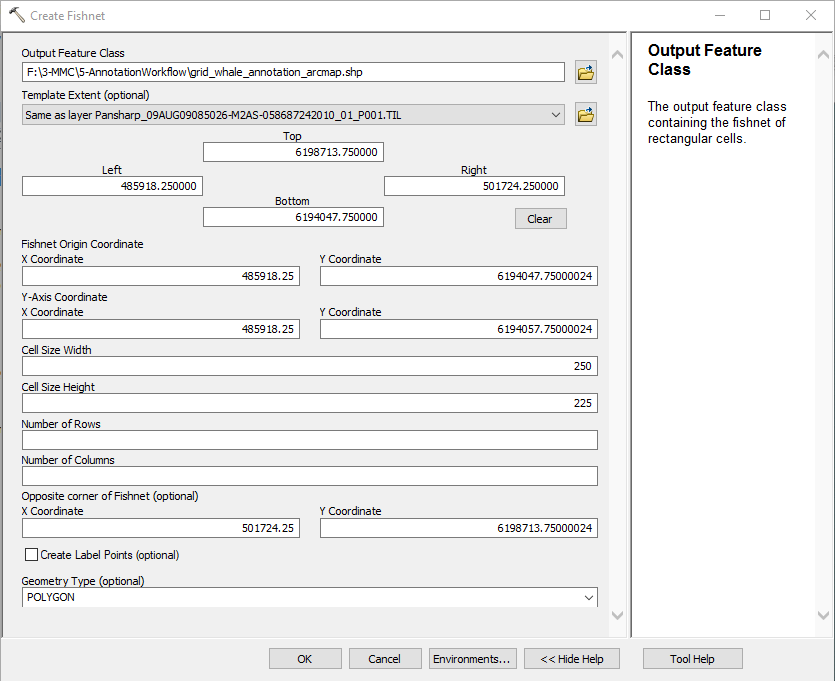


- - 1. After you select “OK”, the grid shapefile will then appear on the “Table of Contents” and in the “Data View”.


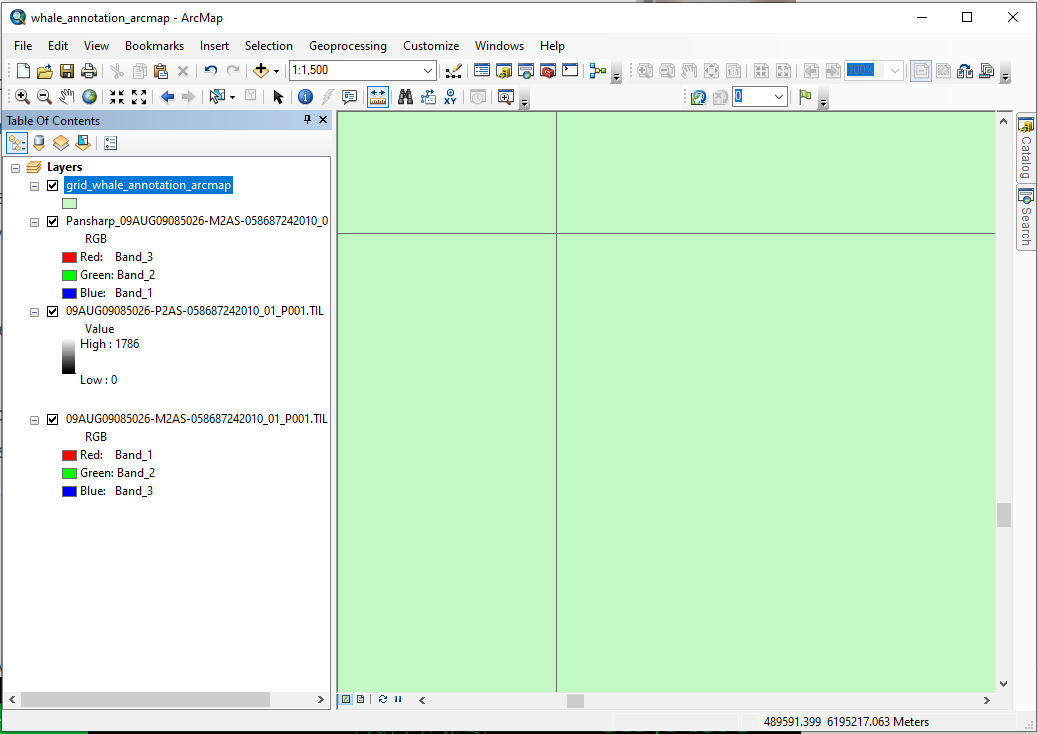


- - 1. Update the symbology to a no fill, thicken the outline width and change the outline color to a color that will contrast with your image. Double click on the colored rectangle below the grid shapefile (here it appears green).


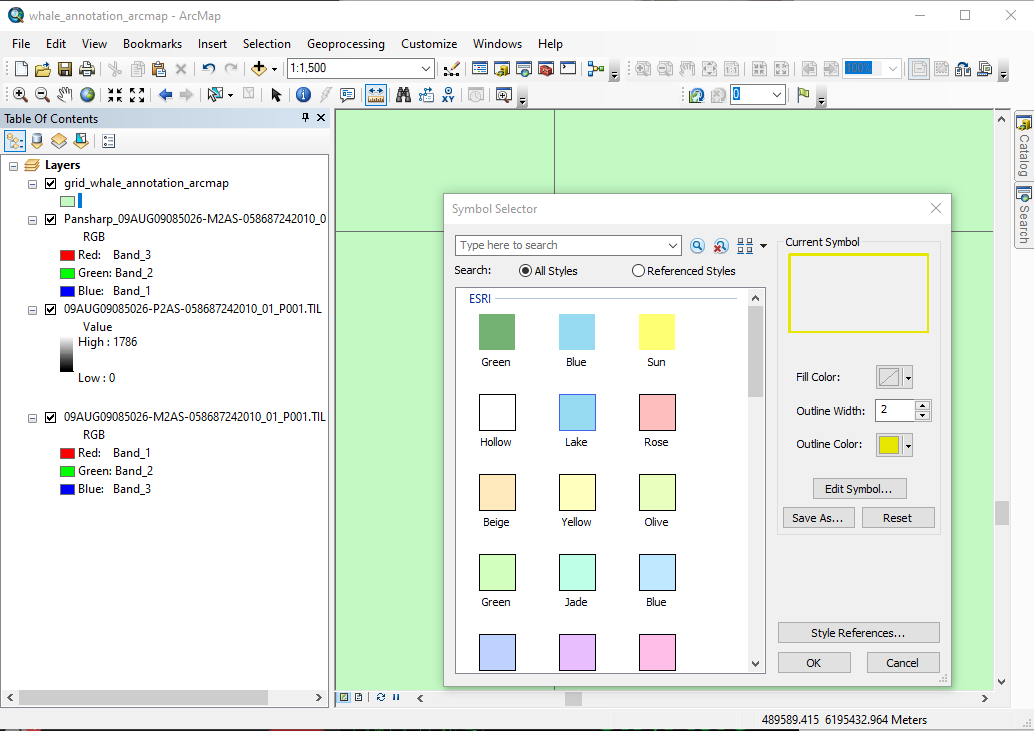


Your workspace should look similar to this.


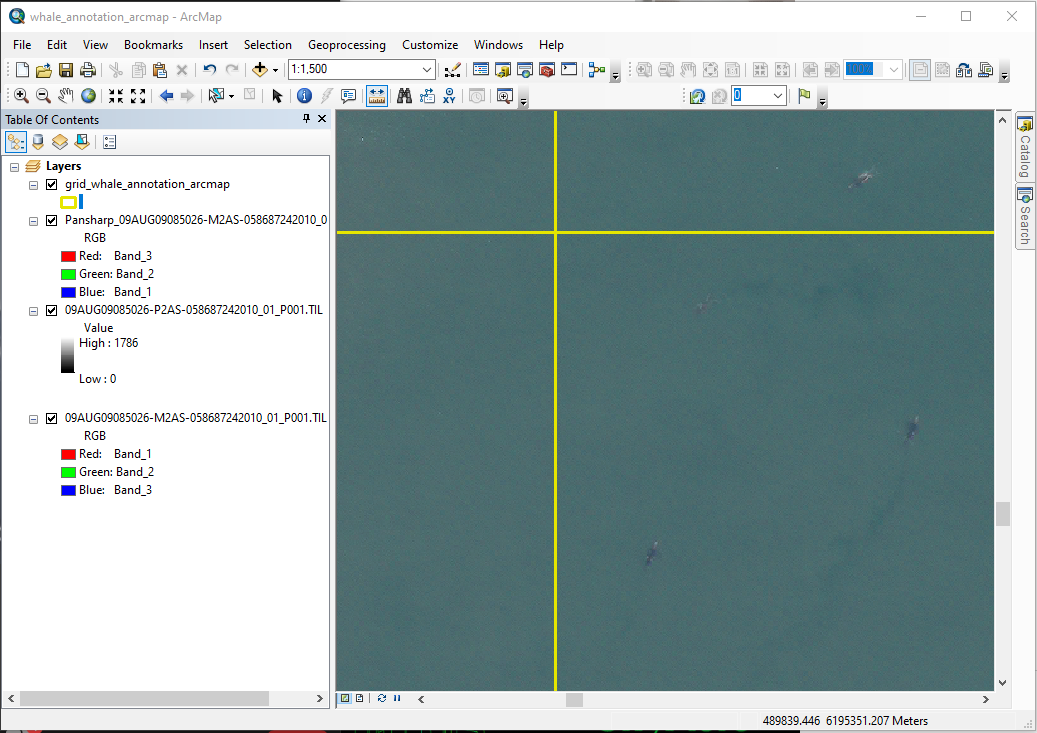


- - 1. To help you keep track of which cell of the grid have been reviewed, we recommend creating a new Field in the attribute table.
       1. To open the attribute table, right click on the grid shapefile, and select “Open attribute table”.


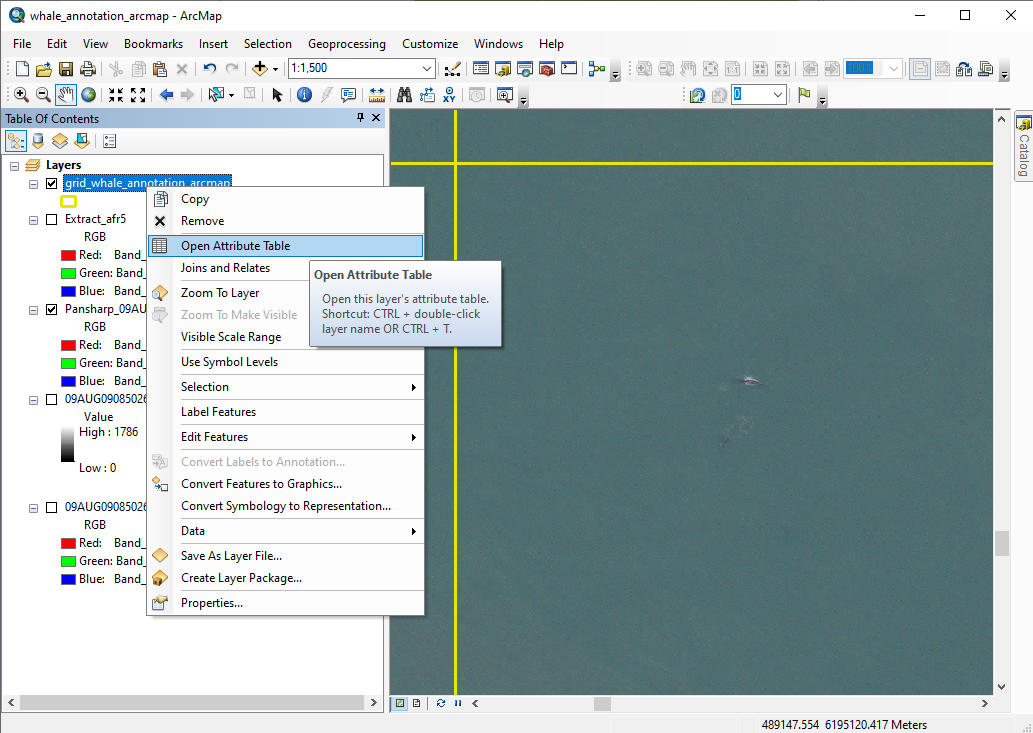


- - - 1. Under the “Table” window, select the black arrow point down next to the “Table Option” icon.


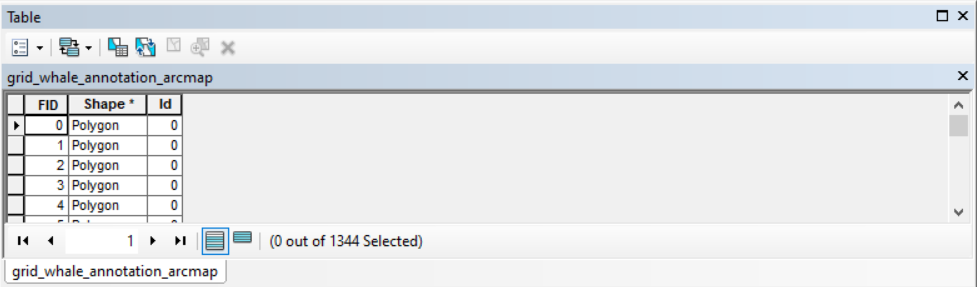


- - - 1. Select “Add Field”


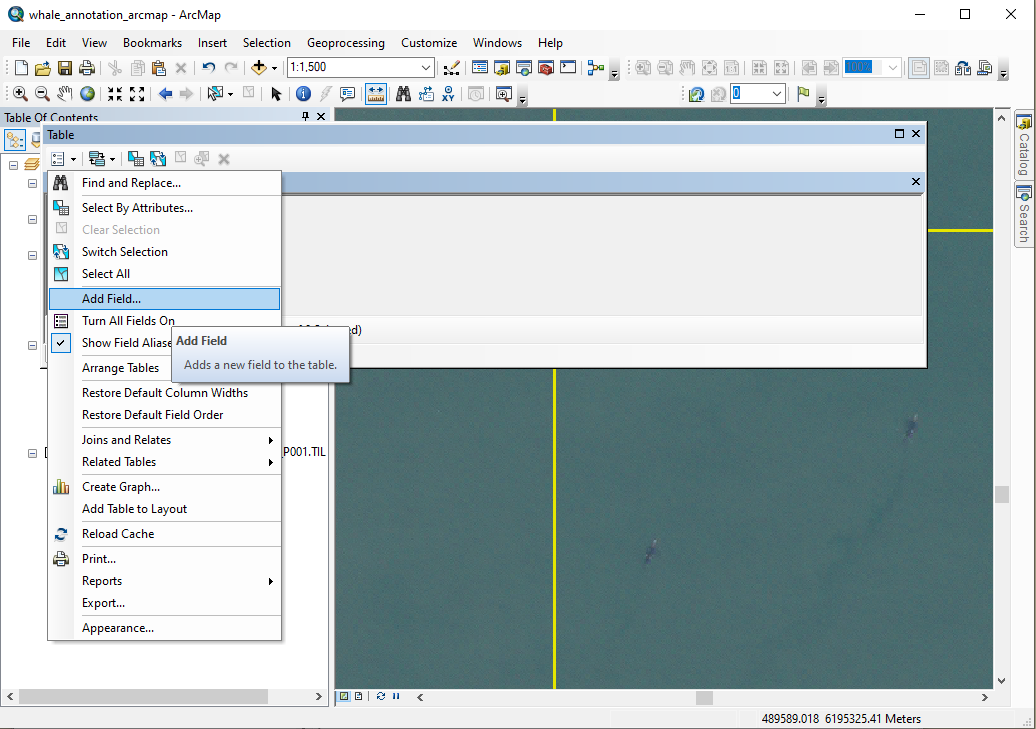


- - - 1. Fill in the “Add Field” window as follow:

Name: Done

Type: Text

Length: 5


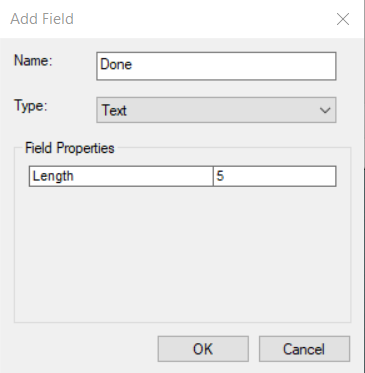


### Creating a point shapefile

1. Create a point shapefile that will be used to annotate the whales you detect in the imagery.
   1. Open ArcCatalog.
   2. In the ArcCatalog window, under the “Catalog Tree”, select “Folder Connections” and go to the folder you wish to save your files in. If you can’t find your folder, you need to create a connection with the following steps, otherwise go to step 8.3:
      1. Select the “Connect To Folder” icon
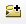
.


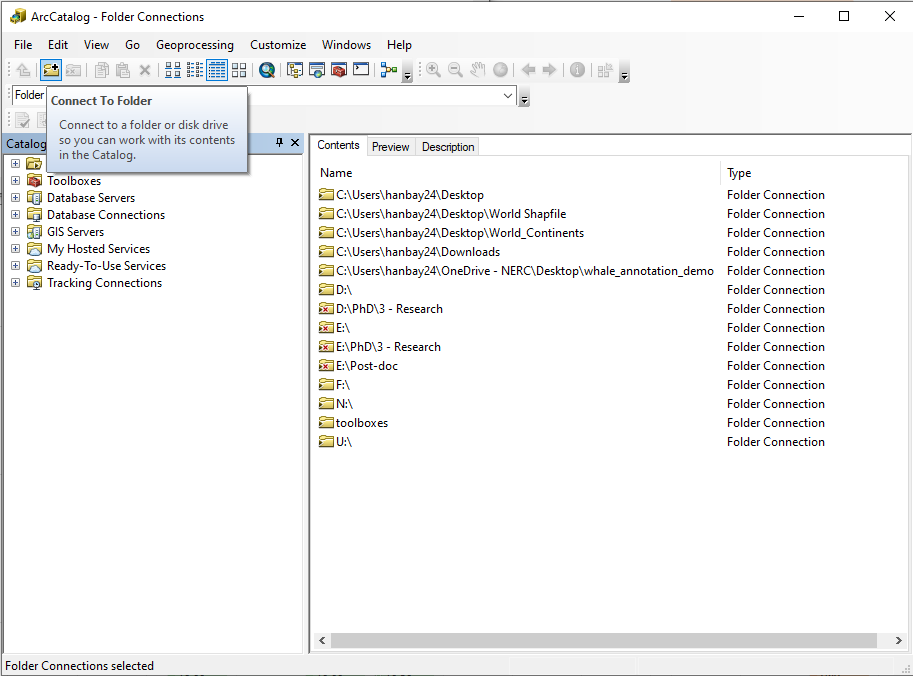


- - 1. In the “Connect To Folder” window, select the folder you wish to connect to save your files in, and select “OK”. Then go back to step 8.2.
  1. Right click in the blank area where there are no folders or files and select “New”, then “Shapefile”.


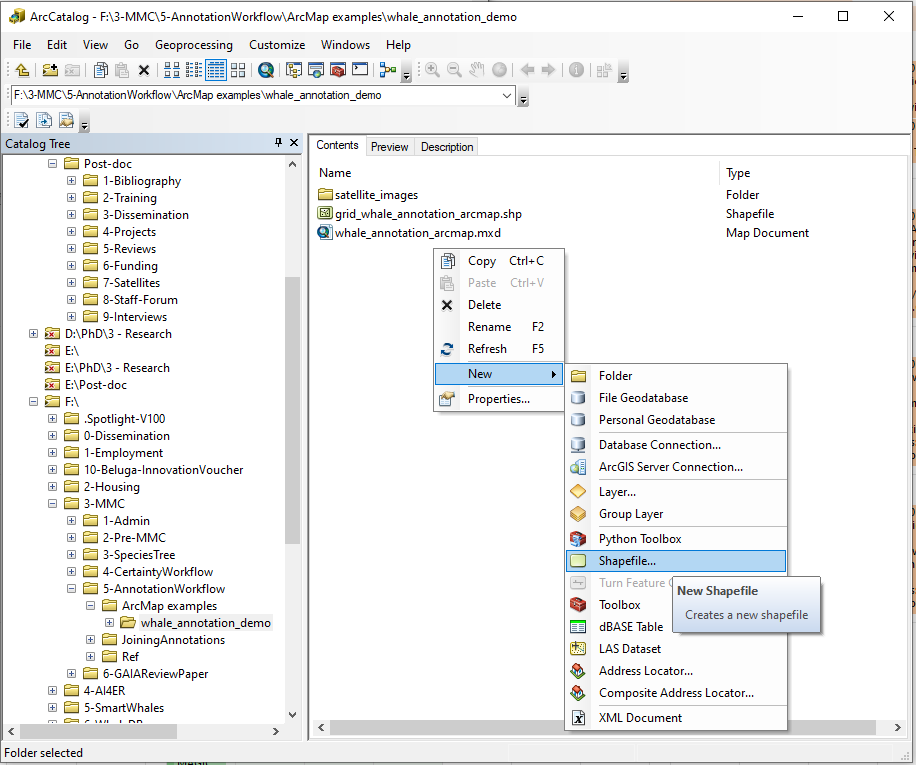


- 1. In the newly opened “Create New Shapefile” window, inform the following and select “OK”:
- Name: give a name to the shapefile (*e.g.* ‘Whales’)
- Feature Type: point
- Spatial reference: select the same spatial reference used of the pansharpened file


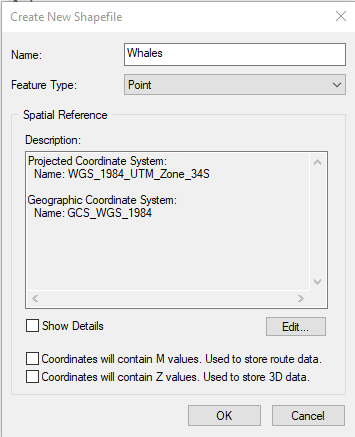


- - - 1. To find out the spatial reference of the pansharpened image, there are different ways, the quickest way is to go back to ArcMap.
         1. Under “Table of Contents”, Right click on the pansharpened file, and select “Properties”.


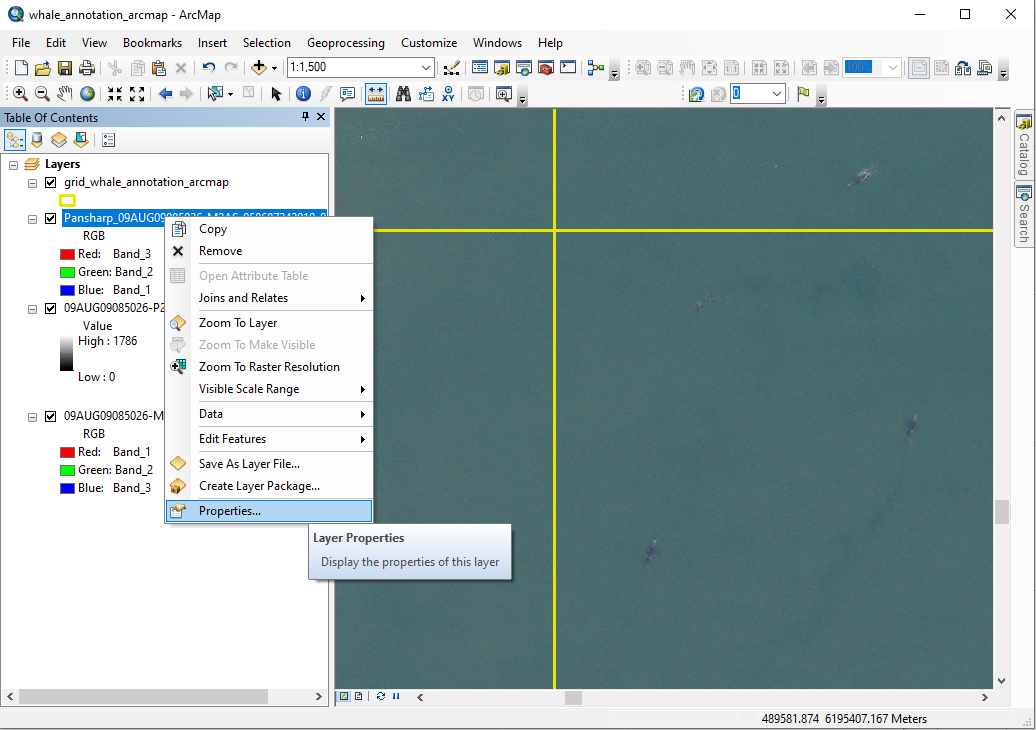


- - - - 1. Under the “Layer Properties” window, select the “Source” tab, and scroll to “Spatial Reference”


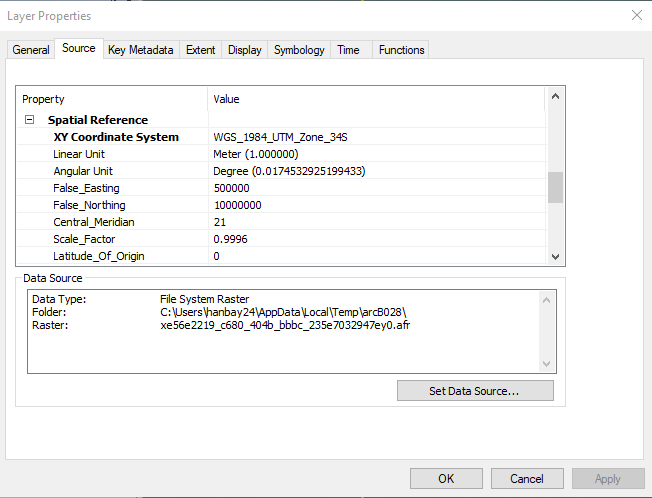


- - - - 1. The spatial reference you want to use for the point shapefile is the same as the value indicated opposite “XY Coordinate System”. Here it is WGS_1984_UTM_Zone_34S.


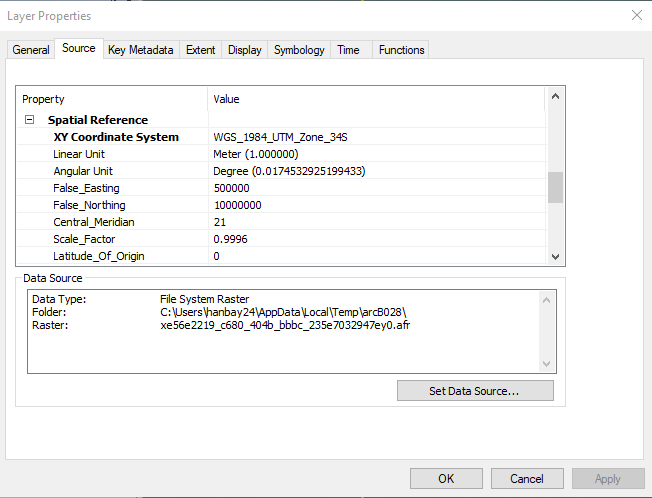


1. Optional: create a point shapefile for other features you would like to record, *e.g.* boats and planes. Repeat steps 8.3. to 8.4.

### Preparing the attribute table

1. Prepare the attribute table attached to the point shapefile you just created. You can either add the field yourself to the attribute table of the point shapefile (steps 10.1. to 10.6., and step 10.8) or you can add a list of pre-made fields (Table 2) to the attribute table to save time, by joining a template attribute table (steps 10.1 to 10.2., then steps 10.7 to 10.8).
   1. Import the point shapefile into ArcMap, by sliding it across from where you saved it in ArcCatalog to ArcMap, or by selecting the “Add Data” icon in ArcMap, and then selecting “Add Data…”.


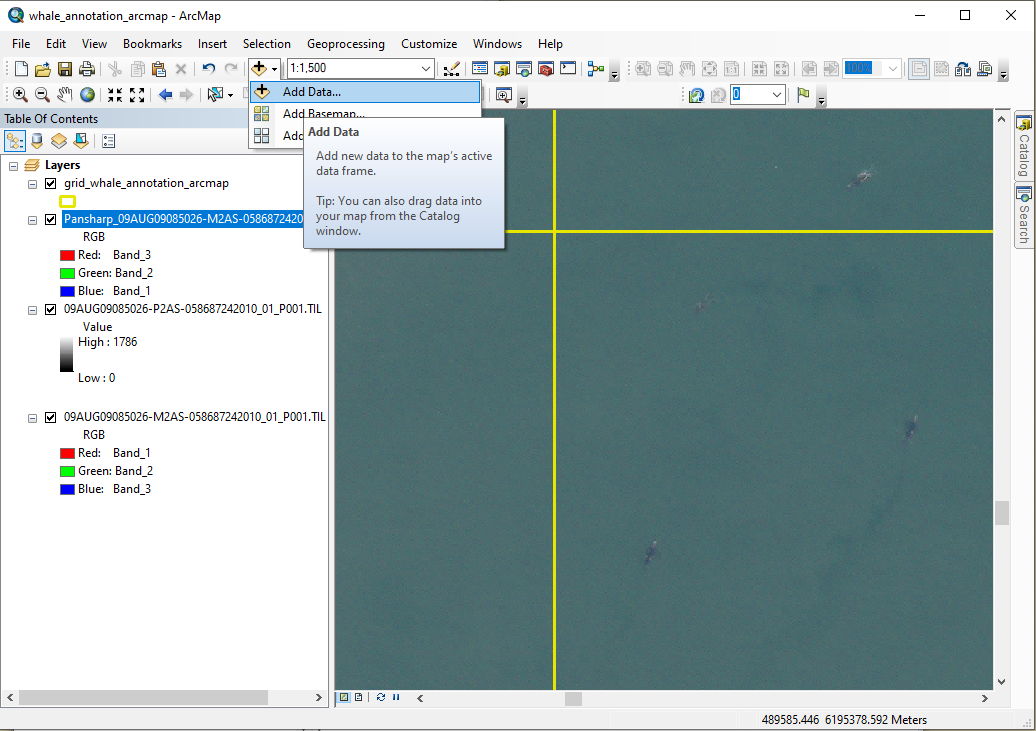


- 1. Under the “Add Data” window, navigate to the connected folder where you saved your point shapefile, select it and press “Add”.


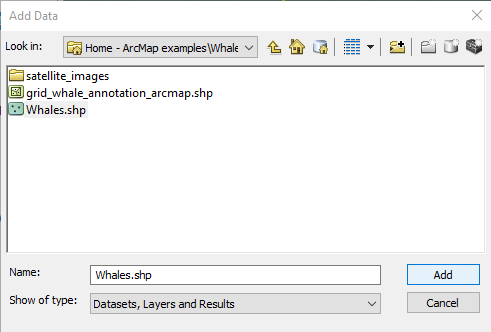


The shapefile will now appear under the “Table of Content”.


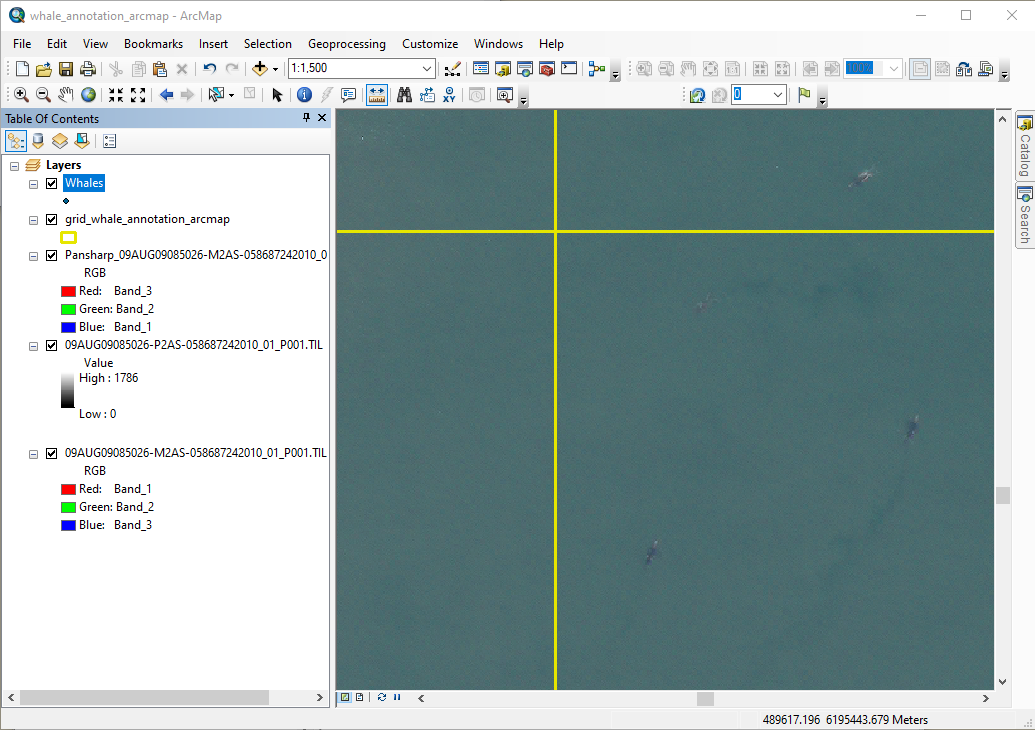


Feel free to change the symbology of the points to a different shape and color by double clicking on it.


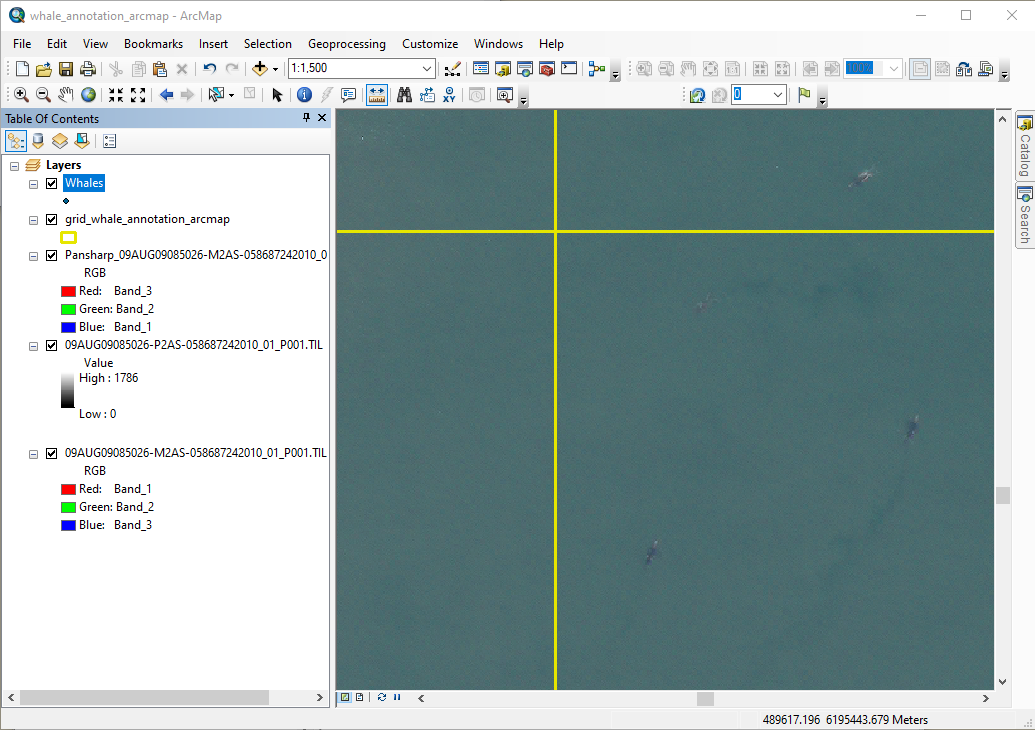


- 1. Open the attribute table. Right click on the point shapefile and select “Open Attribute Table”.


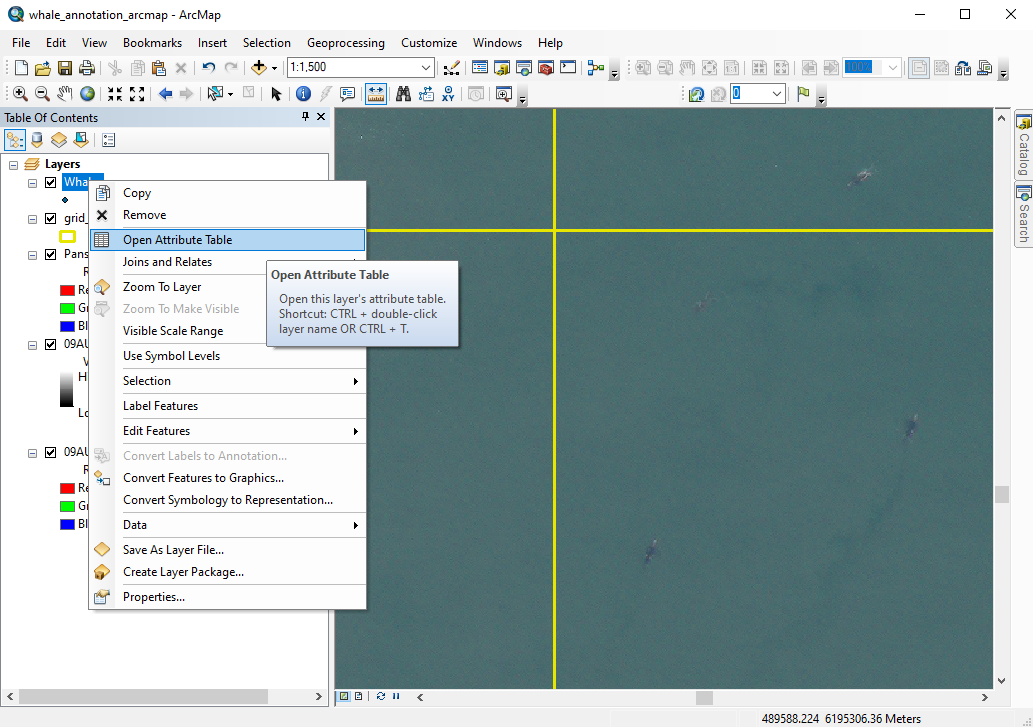


- 1. Under the “Table” window, select the black arrow point down next to the “Table Option” icon.


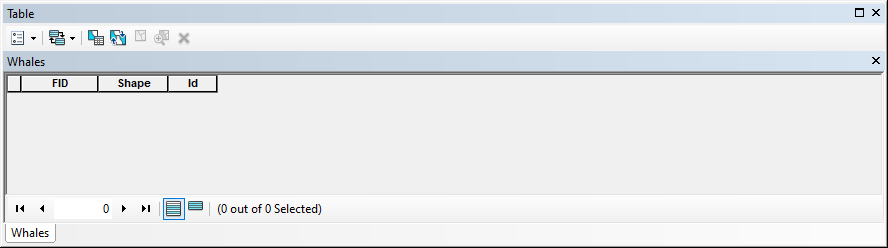


- 1. Select “Add Field”


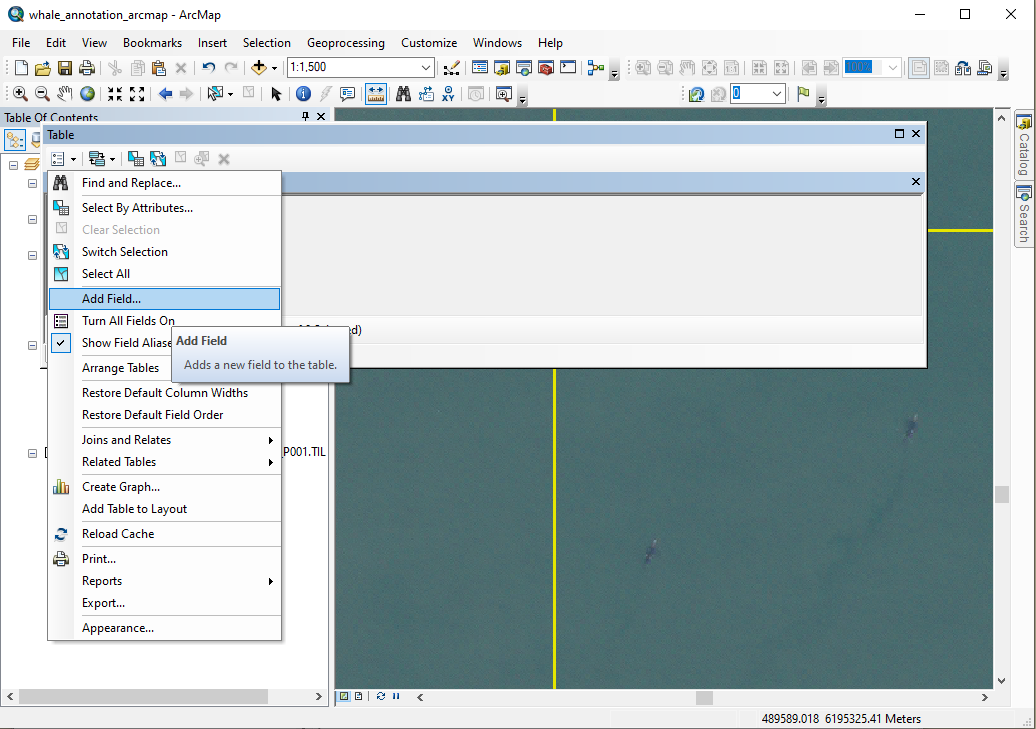


- 1. Fill in the “Add Field” window using the information provided in Table 2 (see Table 3 for a description of each field):

**Table 2.** Information necessary to fill the “Add Field” window, each row represents a different Field.

| Name | Type | Field properties |
| --- | --- | --- |
| observer | Text | Length: 50 |
| location | Text | Length: 25 |
| satellite | Text | Length: 20 |
| gsd_cm | Short Integer | Precision: 0 |
| img_cat_id | Text | Length: 50 |
| img_date | Date | NA |
| img_time | Text | Length: 5 |
| prod_type | Text | Length: 20 |
| sea_state | Short Integer | Precision: 0 |
| cloud_cov | Short Integer | Precision: 0 |
| cloud_th | Short Integer | Precision: 0 |
| glare | Short Integer | Precision: 0 |
| turbidity | Short Integer | Precision: 0 |
| otr_env | Text | Length: 100 |
| latitude | Double | Precision: 0  Scale: 0 |
| longitude | Double | Precision: 0  Scale:0 |
| gcs | Text | Length: 50 |
| projection | Text | Length: 50 |
| sp_code | Text | Length: 50 |
| certainty | Text | Length: 10 |
| body_color | Text | Length: 50 |
| body_shp | Text | Length: 50 |
| body_l | Short Integer | Precision: 0 |
| body_w | Short Integer | Precision: 0 |
| flipper | Short Integer | Precision: 0 |
| lg_flipper | Short Integer | Precision: 0 |
| fluke | Short Integer | Precision: 0 |
| head_callo | Short Integer | Precision: 0 |
| wh_lr_jaw | Short Integer | Precision: 0 |
| aft_breach | Short Integer | Precision: 0 |
| bubble_net | Short Integer | Precision: 0 |
| contour | Short Integer | Precision: 0 |
| flukeprint | Short Integer | Precision: 0 |
| wake | Short Integer | Precision: 0 |
| blow | Short Integer | Precision: 0 |
| mudtrail | Short Integer | Precision: 0 |
| sur_act_gr | Short Integer | Precision: 0 |
| travel_gr | Short Integer | Precision: 0 |
| mc_pair | Short Integer | Precision: 0 |
| otr_gr | Short Integer | Precision: 0 |
| defecation | Short Integer | Precision: 0 |
| comment | Text | Length: 200 |

- 1. If you wish to add the same attributes as in Table 2 faster, you can join the template attribute table (Supplementary material 6) to your point shapefile. Use the “Join Field” tool. You can either access the tool via the “ArcToolbox” icon
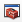
, or the “Search” icon
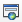
.
     1. You need to import the “Template_AT” shapefile (Supplementary material 6), similarly to step 10.1.
     2. If using ArcToolbox, select “Data Management Tool”, then select “Joins”, then “Join Field”.


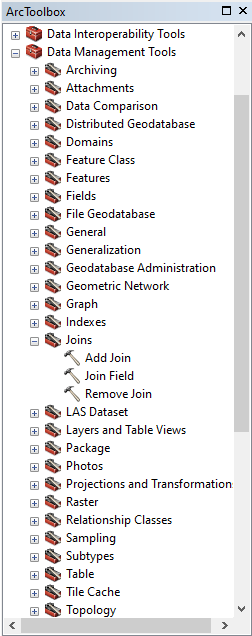


- - 1. If using the “Search” icon, type in “join” and select the “Join Field (Data Management)”.
    2. Under the “Join Field” window, fill in the following information, then select “OK”:

Input Table: the point shapefile you created at step 8

Input Join Field: FID

Join Table: Template_AT (Supplementary material 6)

Output Join Field: FID


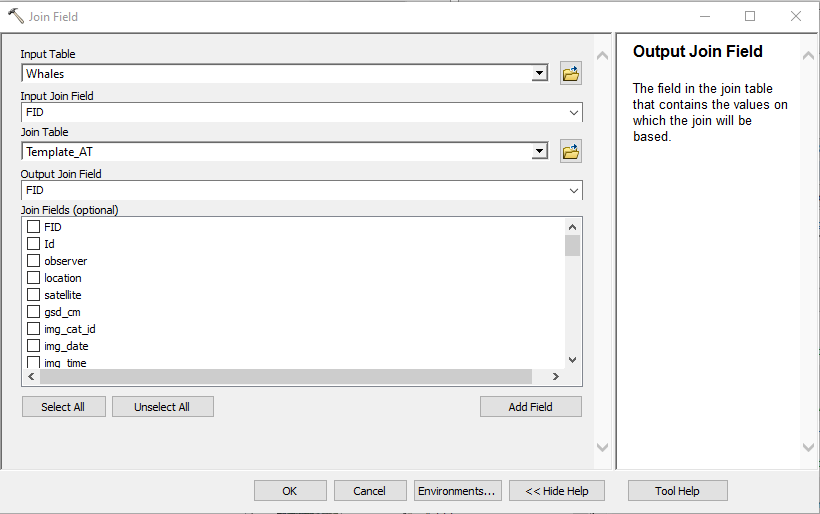


- 1. After having added all the fields listed in Table 2, or after having joined the template attribute table to your point shapefile, your “Table” window should look similar to this.


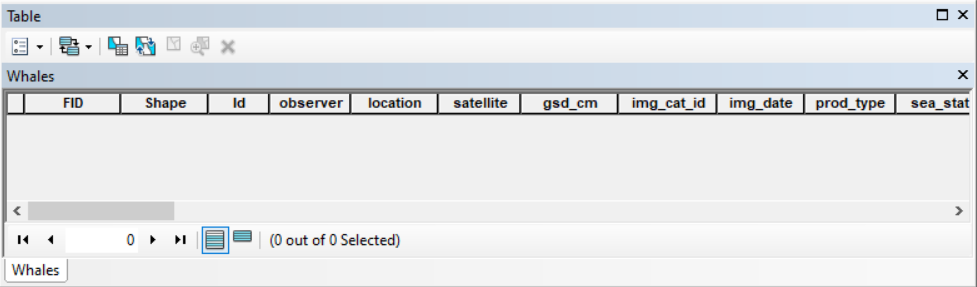


# Reviewing the Image

## Systematic scanning

Scanning the image for the presence of whales should be systematic; therefore, we recommend overlaying the grid created at step 7.5 on top of the pansharpened image, and reviewing one cell after another as shown below.


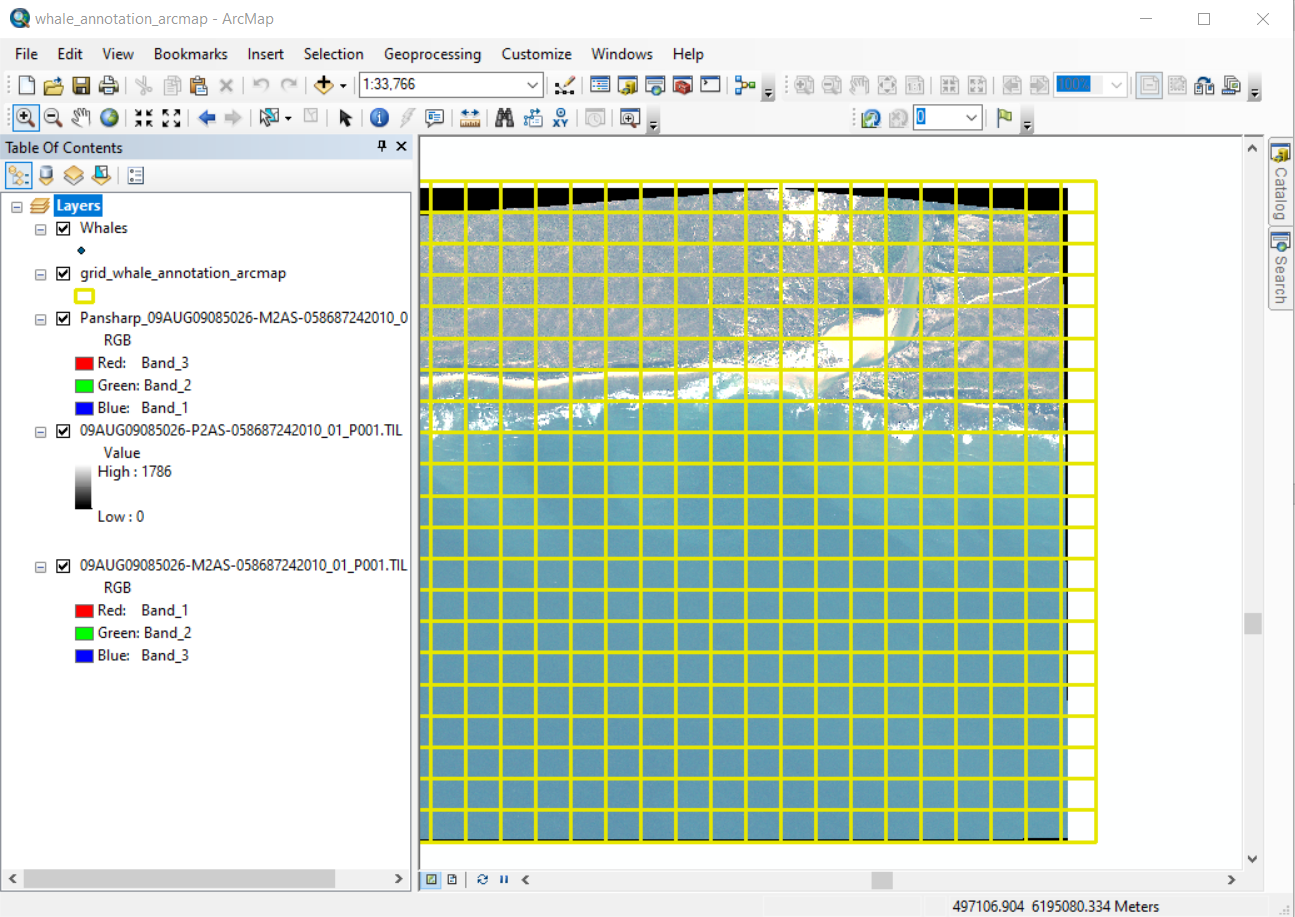


## Scale

We also recommend scanning the image at the scale at which the grid was created (*e.g.* 1:1,500 for large whales).

## Keeping track

To keep track of which cells were reviewed, we recommend filling the field “Done” in the attribute table after a couple rows or more or less frequently depending on the shape of the image that is being reviewed.

1. Allow editing of the grid shapefile
   1. In the “Table of Contents”, right click on the grid shapefile and select “Edit Features”, and then select “Start Editing”.


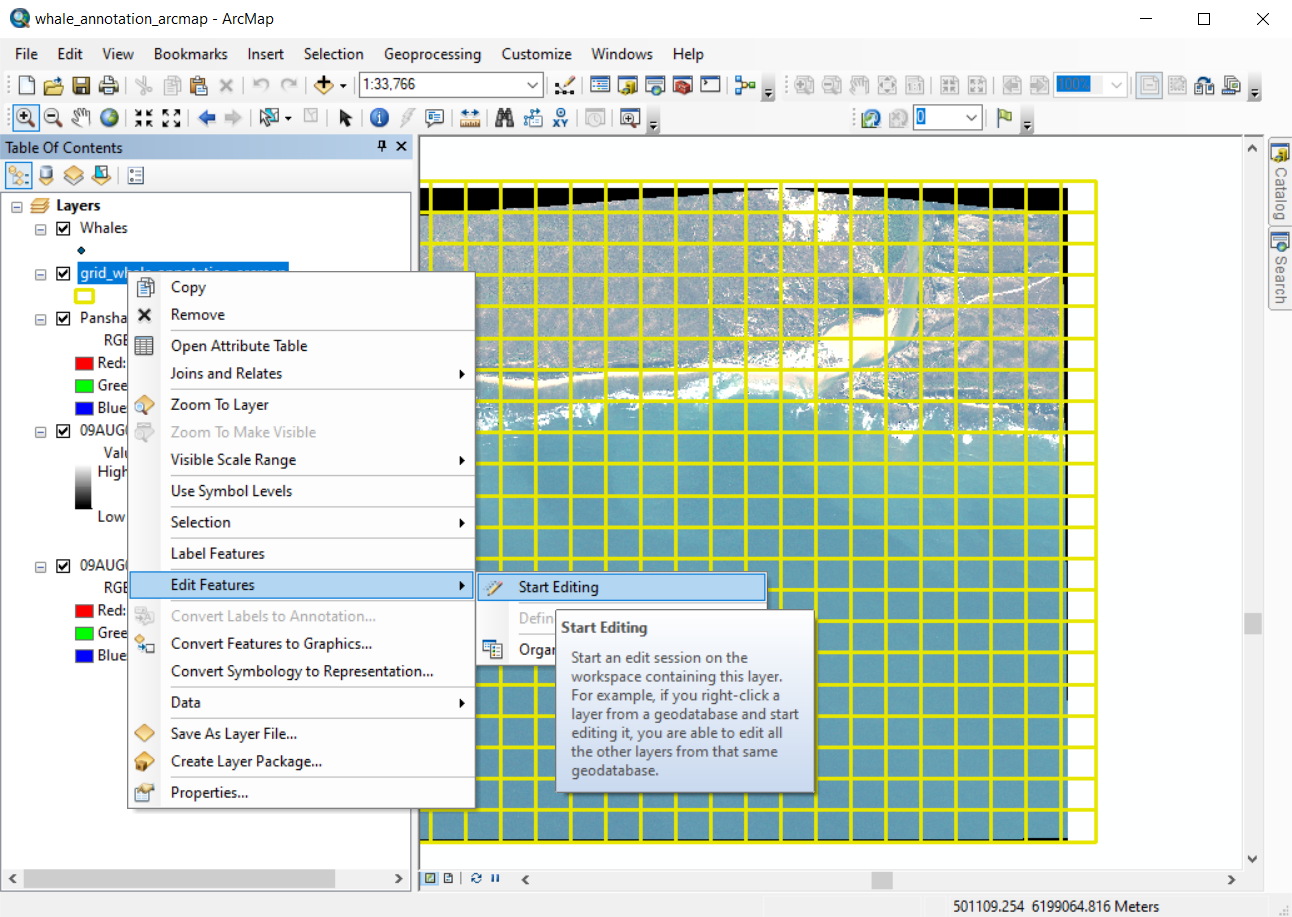


- 1. Open the attribute table, by right clicking on the grid shapefile similar to the step above.


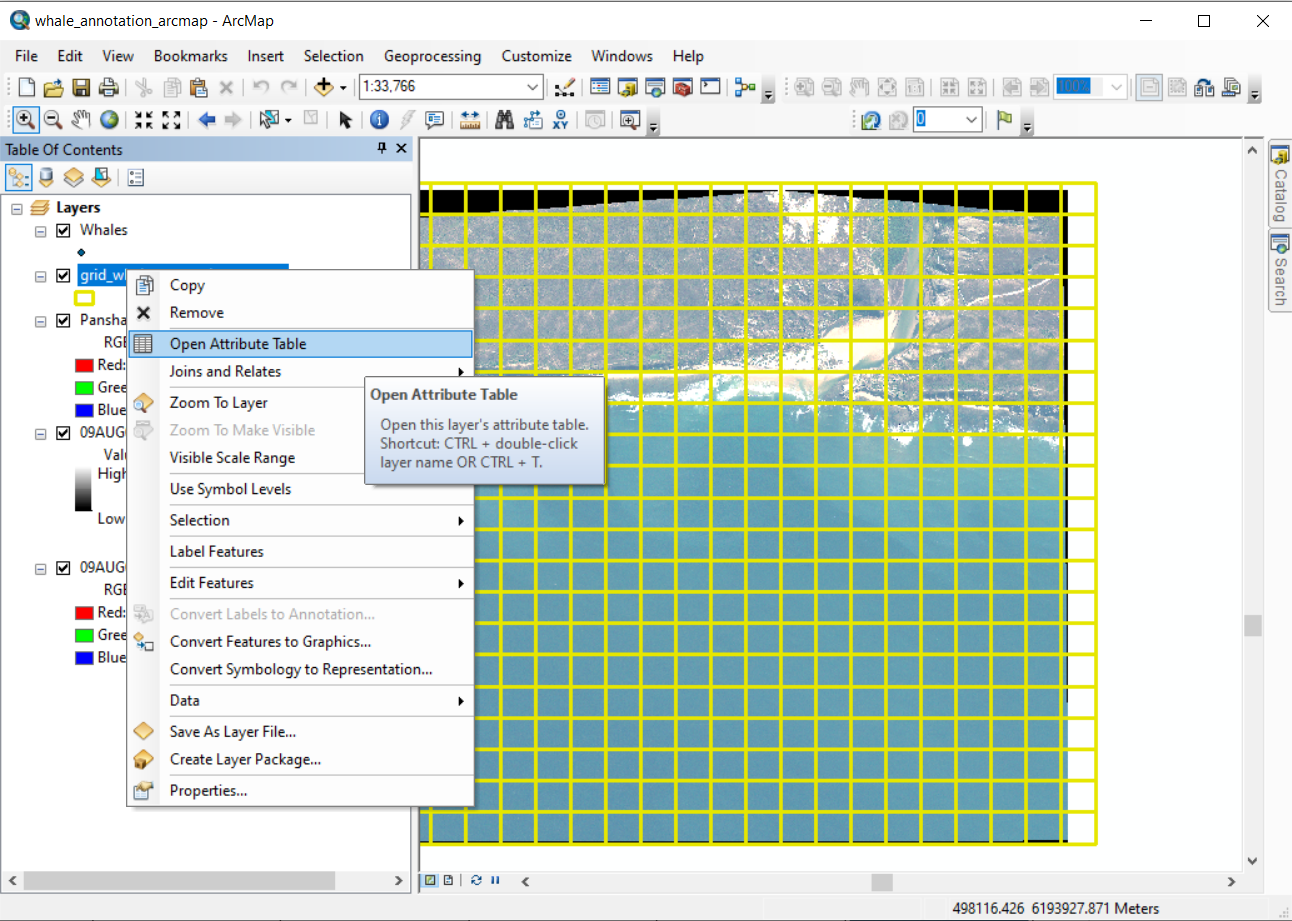


- 1. You can now write in each cell of the attribute table. We recommend typing “Yes” for the cell of the grid that have been reviewed.


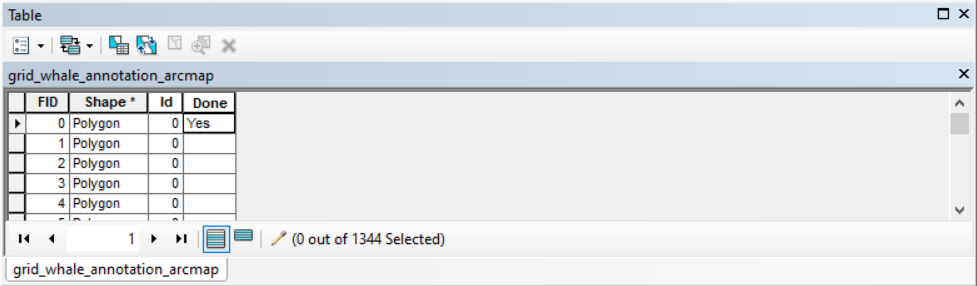


- 1. Make sure to save your edits. In the “Editor” toolbar, select the black arrow point down next to “Editor” and select “Save Edits”. When you log off, make sure to select “Stop Editing”.


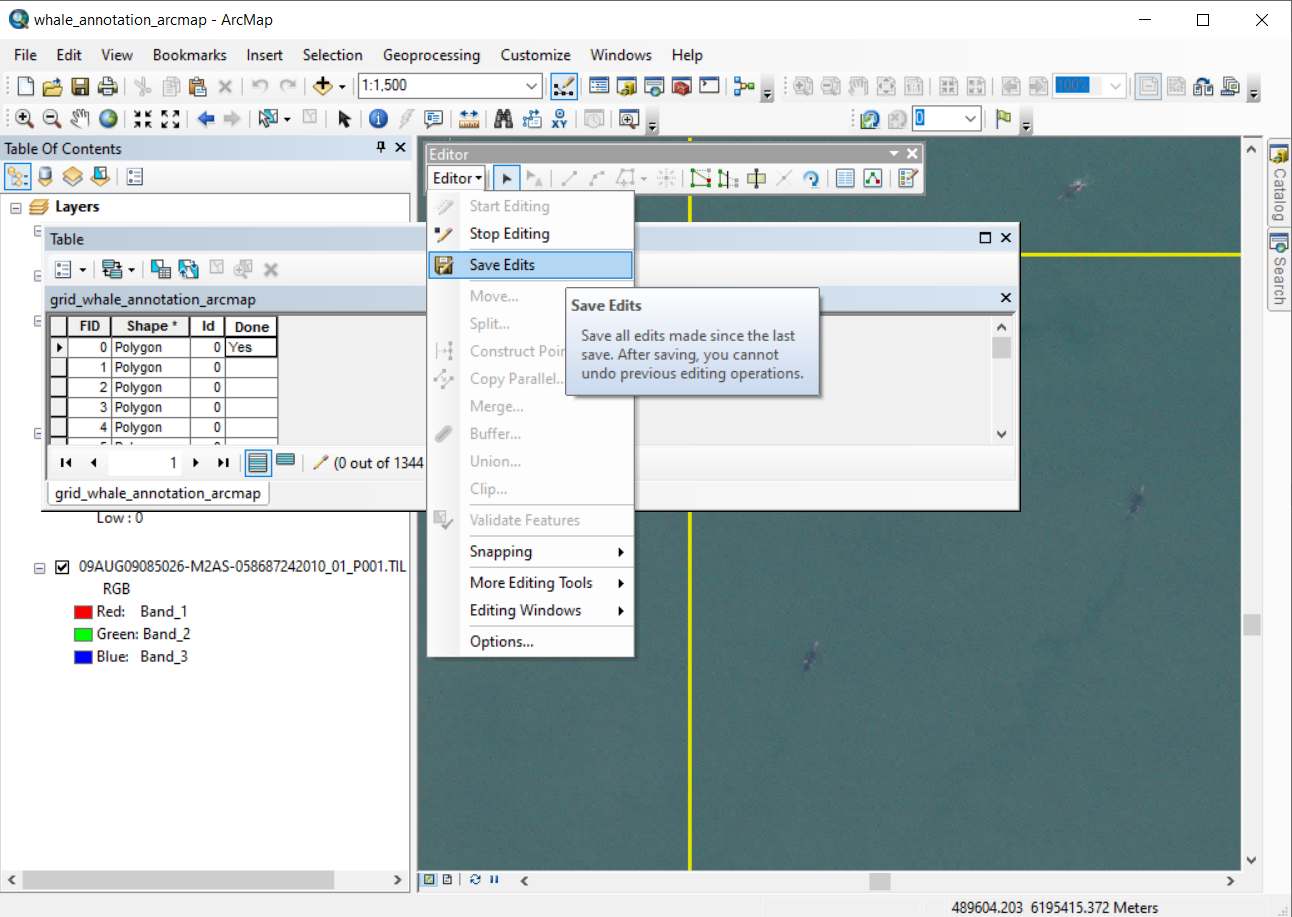


- 1. You can update the symbology of the grid shapefile to show the cells of the grid review in a different color than the cells that need reviewing.
     1. In the “Table of Contents” window, right click on the grid shapefile, then select “Properties”, then in the “Properties” window, select the “Symbology” tab, and update the following information:
- Categories: Unique Values
- Value Field: Done
- Select “Add values” then the “Yes” and empty value will appear.
- Update the symbology of the “Yes” and empty values by double clicking on the polygon, we recommend to use a no fill and colored outline for the empty value, and a transparency fill and colored outline for the “yes”.


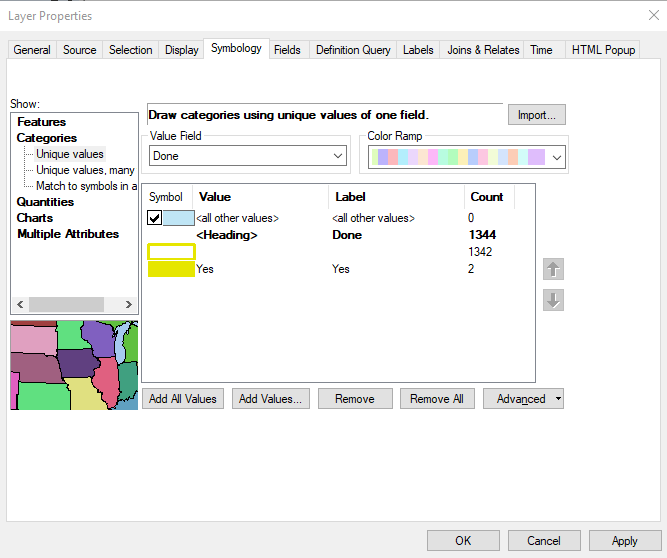


# Annotating

You are now all set up to start scanning the image and annotating when you find whales (or your feature of interest, such as confounding features).

## Placing points on top of whales

1. To be able to place a point on top of the whales you have found, you need to be in an editing mode. Right click the whale shapefile created step 8.4, select “Edit Features”, then select “Start Editing”.


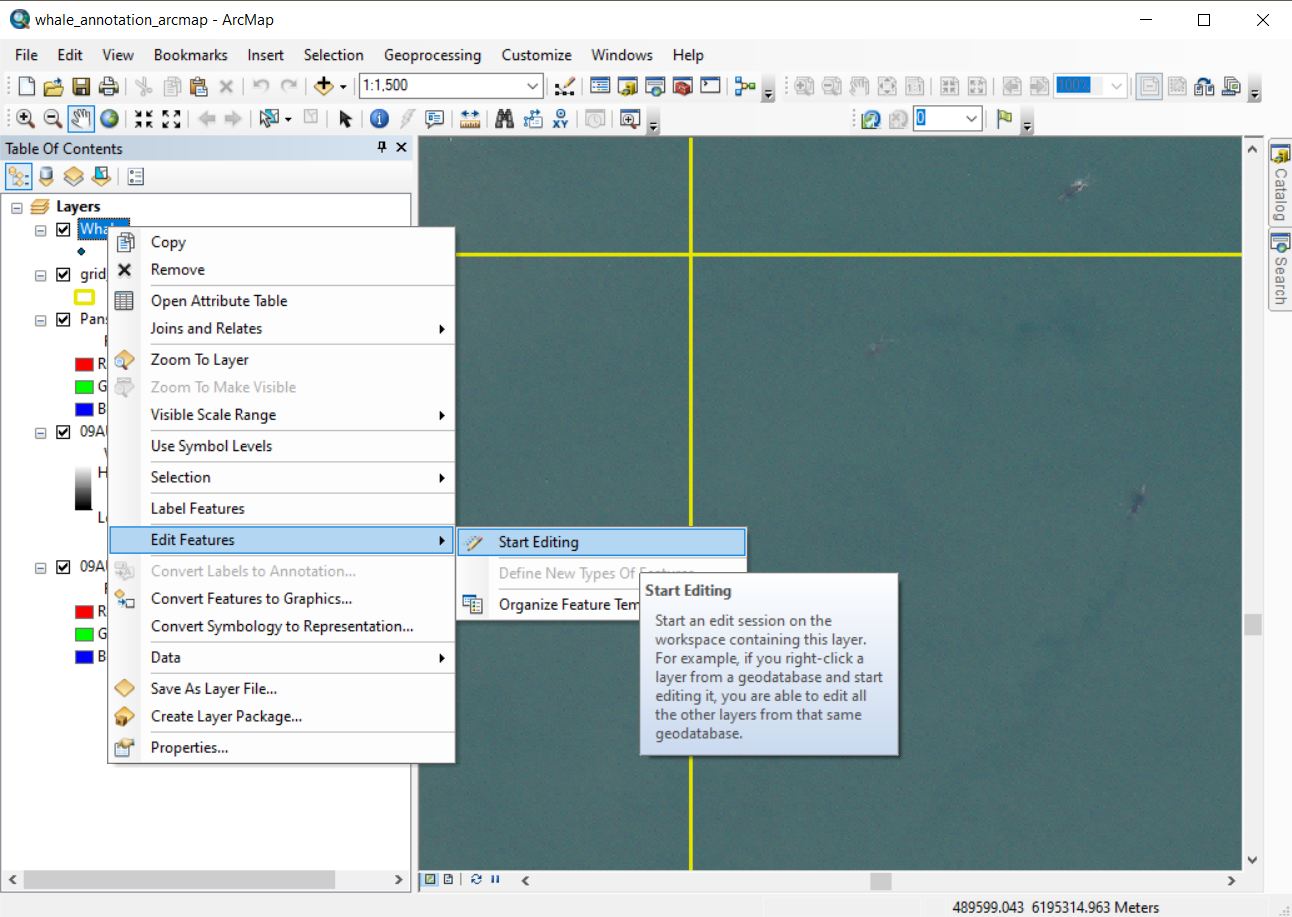


1. Under the “Editor” toolbar that just opened, select the black arrow pointing down next to “Editor”, and select ”Editing Windows”, then select “Create Features”.


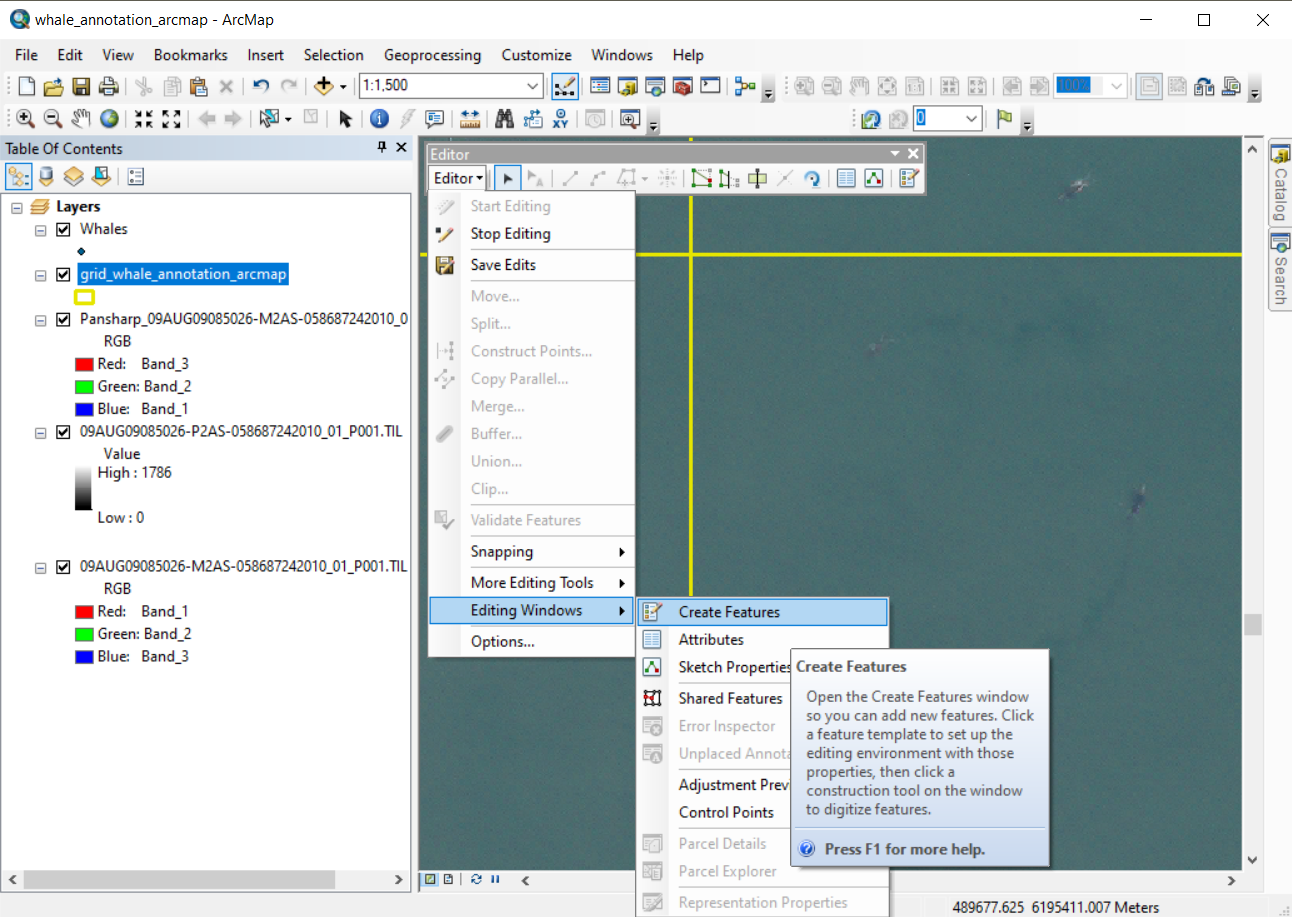


1. Under the new window that opened “Create Features”, select the whale shapefile created in step 8.4. Then under “Construction Tools” tab, select “Point”.


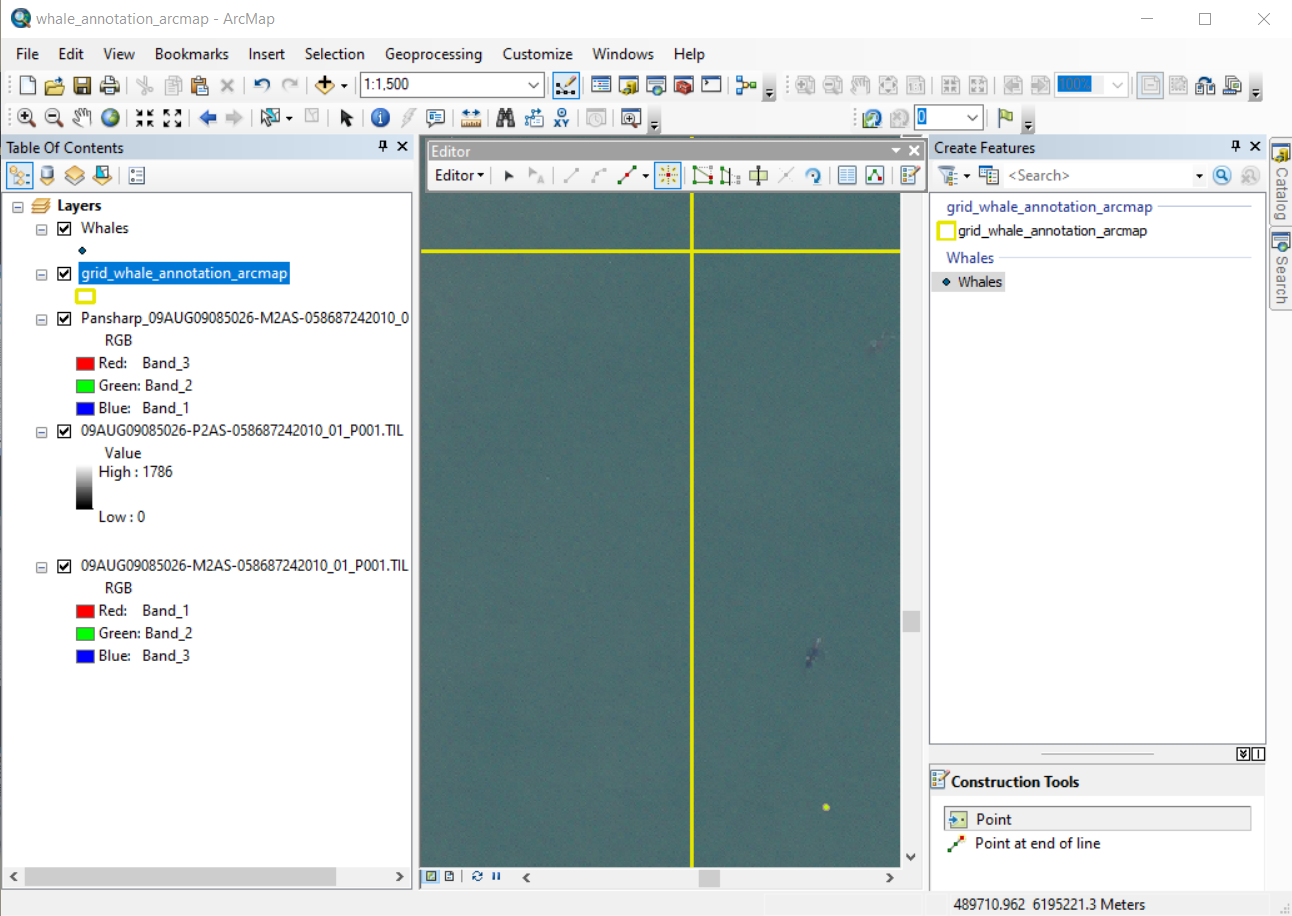


1. You are now ready to place a point on the image, by clicking in the middle of the whale.

**IMPORTANT:** it is crucial that the point is in the center of the visible part of the whale to facilitate joining the annotations of different observers.


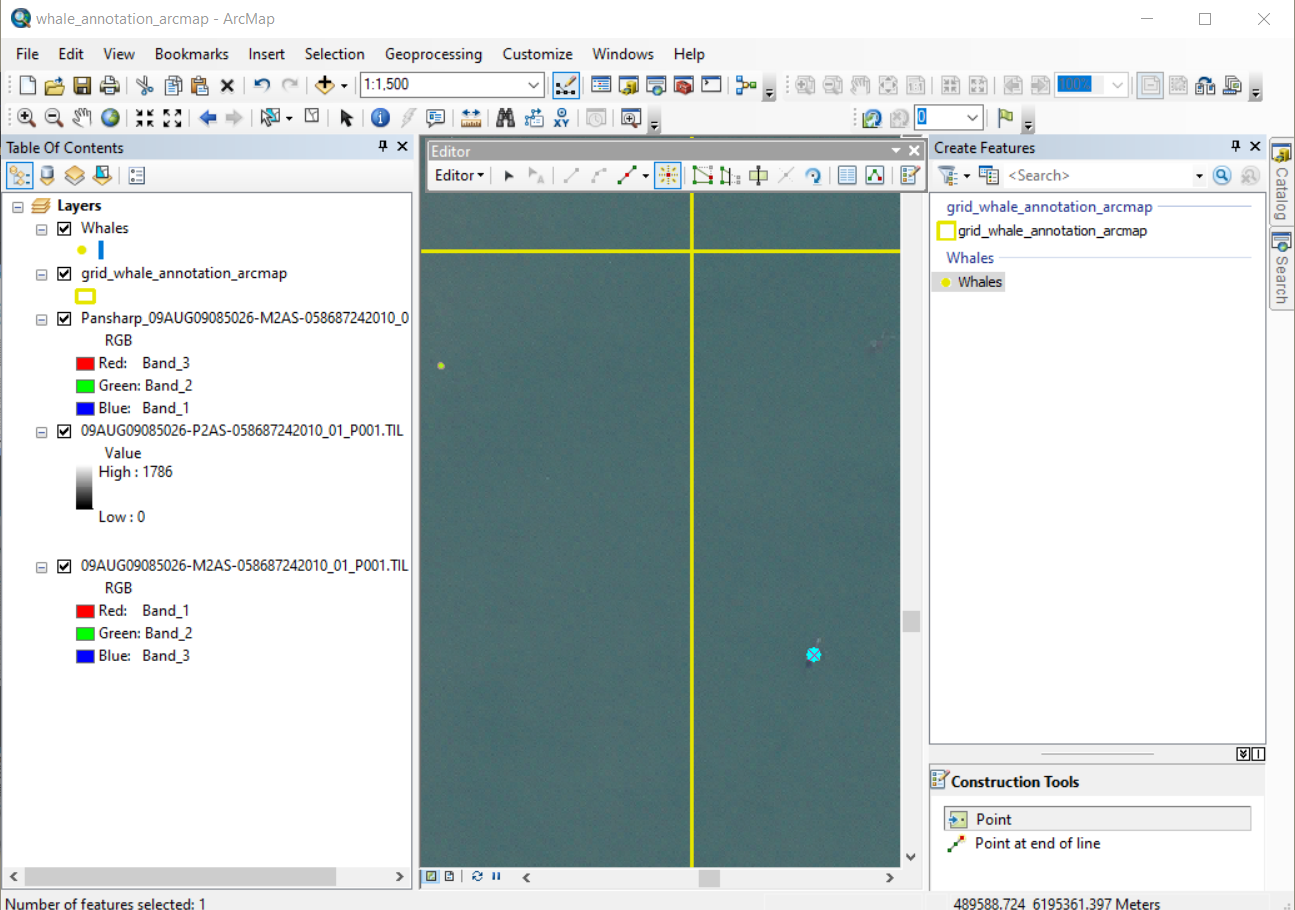


1. Make sure to save your edits, frequently, by selecting the black arrow pointing down next to “Editor”, and selecting “Save Edits”. As good practice we also recommend saving the whole map file regularly, by selecting the “Save” (floppy disk) icon in the toolbar.


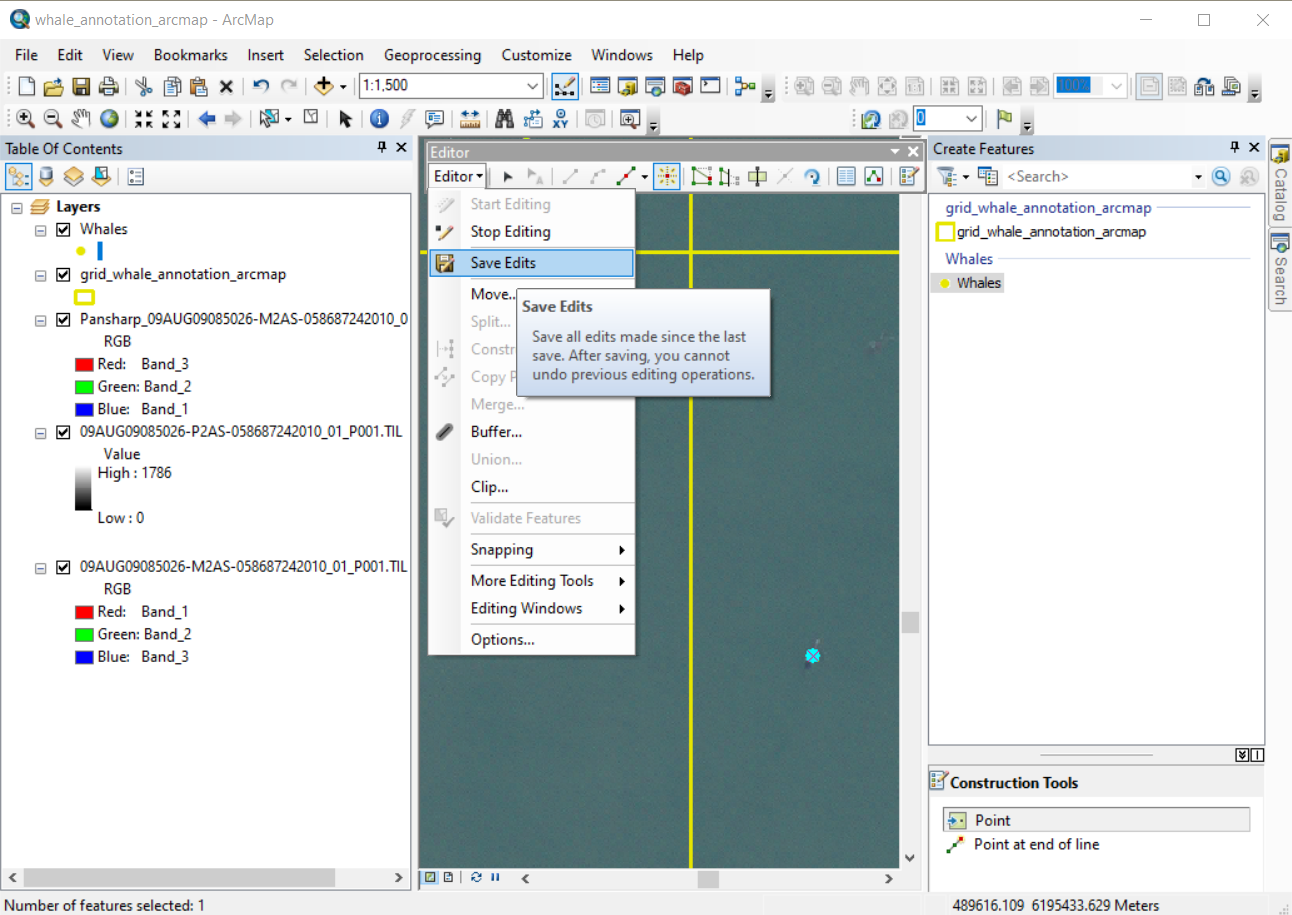


1. If you wish to leave this session, make sure to stop the edition session by selecting the black arrow pointing down next to “Editor”, and selecting “Stop Edits”.


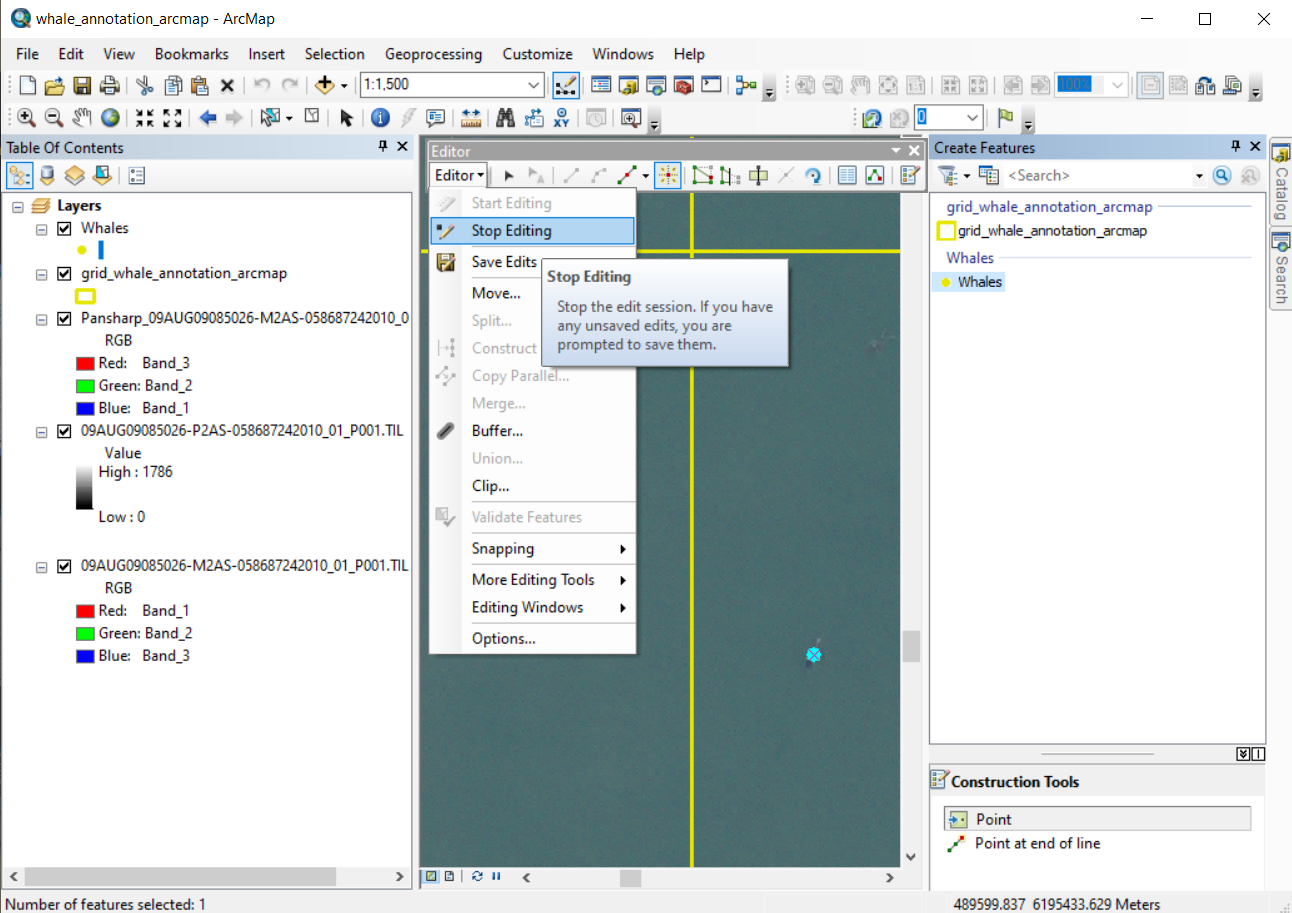


## Filling in the attribute table

Once you have fully scanned the image and placed points in the center of the detected whales, you can fill in the attribute table. Refer to Table 3 to know what information should go under each Field.

1. Open the attribute table, right click on the whale shapefile and select “Open Attribute Table”.


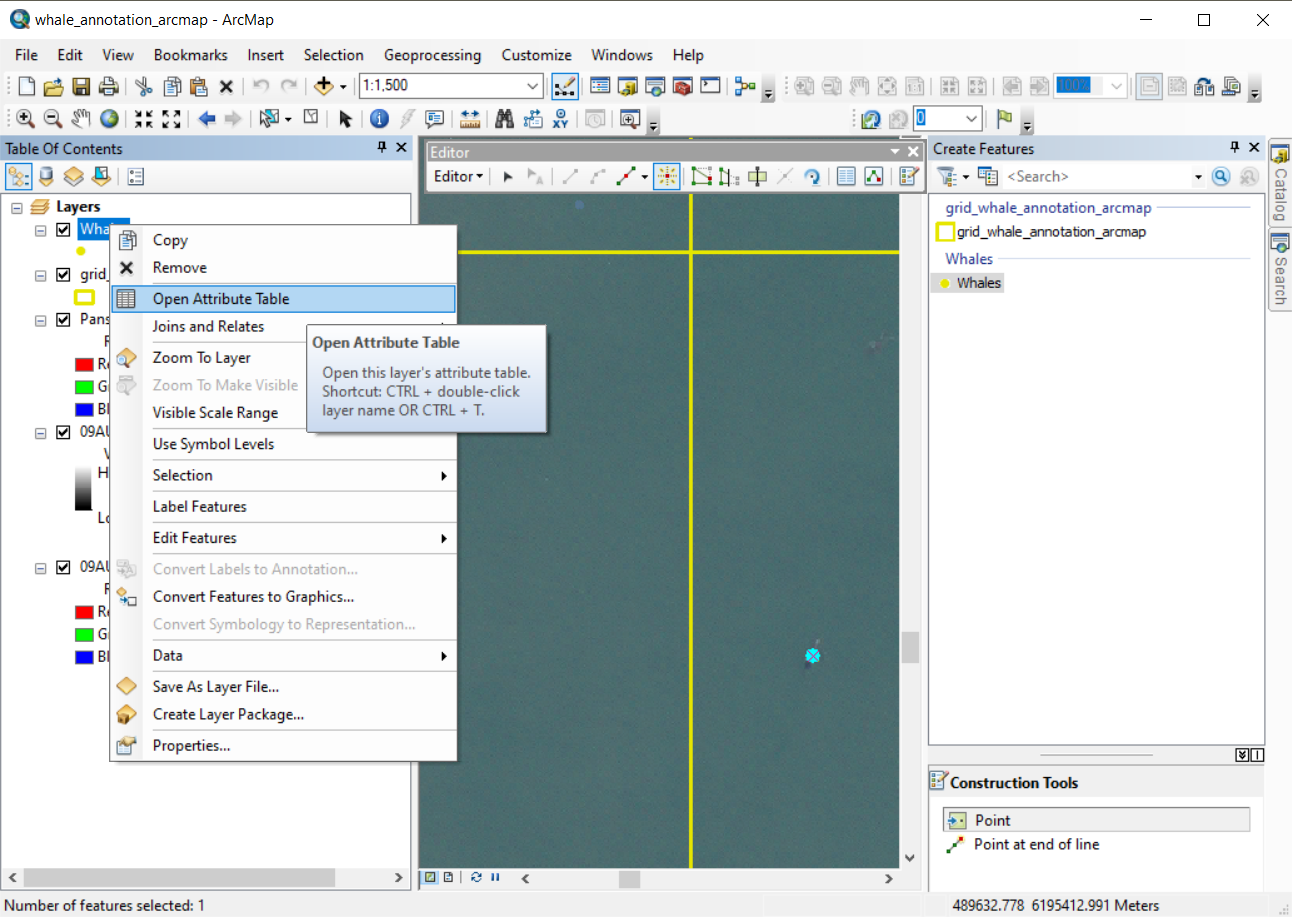


1. If you left the edition session open at step 12, reopen it following the instructions from step 12.
2. Fill in the different fields for each whale points.


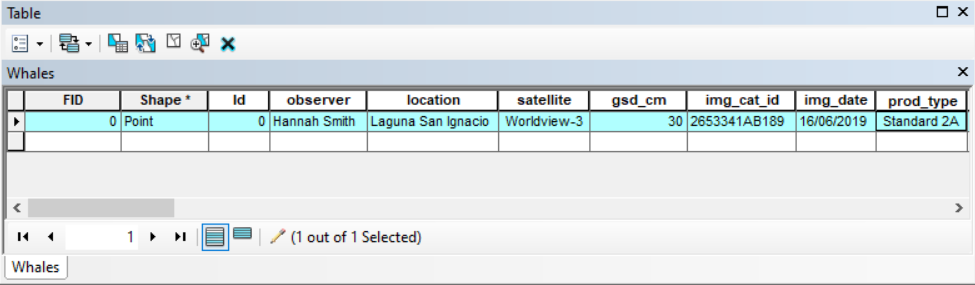


**TIPS:** to fill in the information quicker:

- you can use the “Field Calculator” tool for the following fields as they will contain the same information for all whale points: observer, location, satellite, gsd_cm, img_cat_id_img_date, prod_type, sea_state, cloud_cov, cloud_th, glare, turbidity, otr_env, gcs, projection. Right click on the name of the field, e.g. satellite as shown below and select “Field Calculator”.


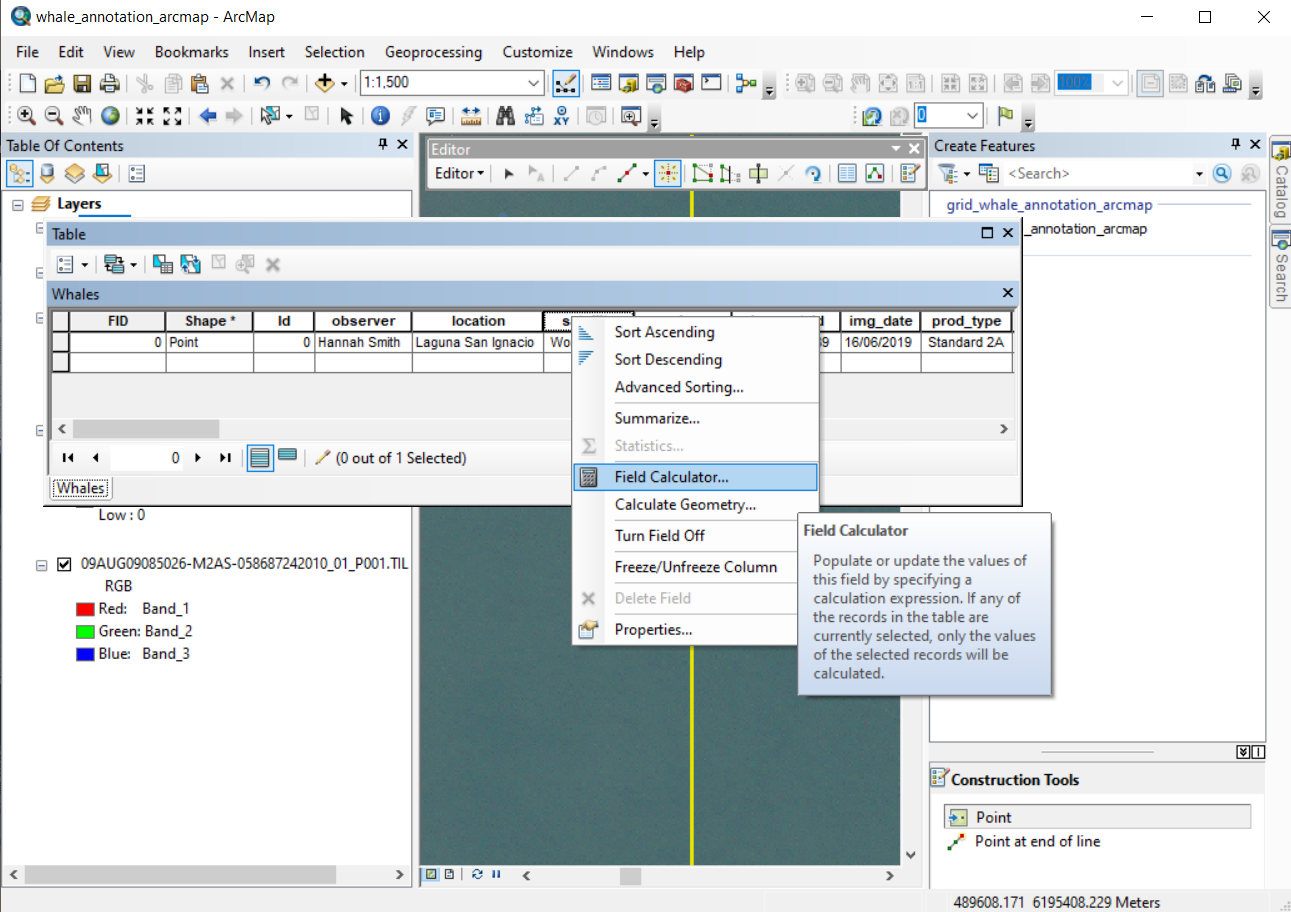


Under the “Field Calculator” window, you can write the value that will be the same through the whole field, e.g. for the Field “satellite”, you can type “WorldView-3” and select “OK”, as shown below and it will automatically populate every row in that column with the value “WorldView-3”.


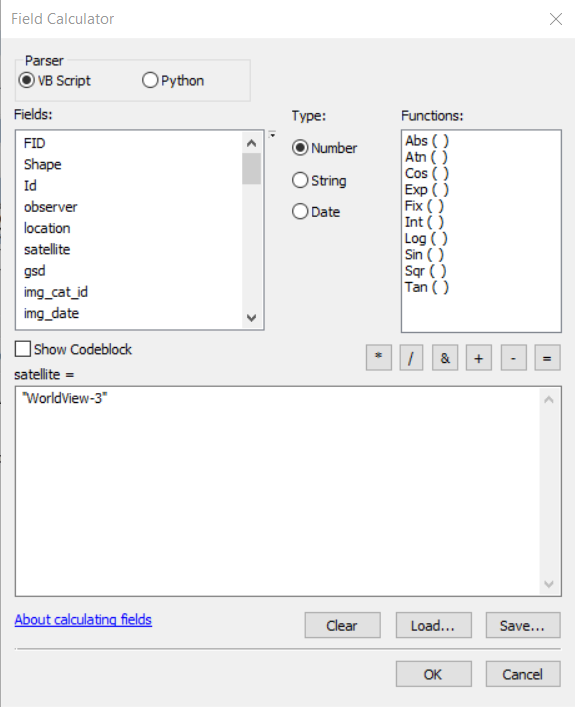


- you can use the “Calculate Geometry” tool, for the fields: latitude, longitude. Right click on the name of the field, e.g. latitude as shown below and select “Calculate Geometry”.


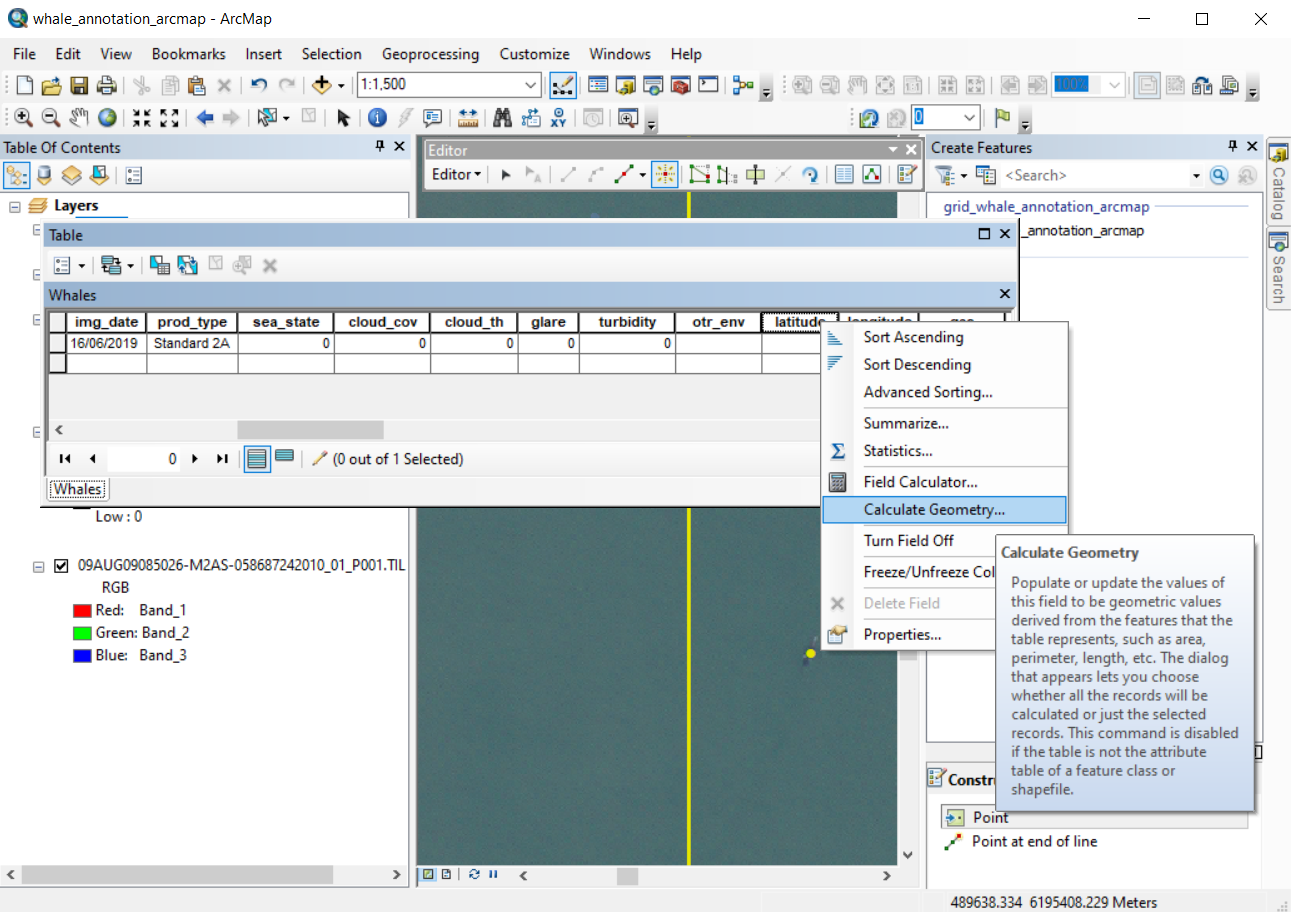


Under the “Calculate Geometry” window, enter the following information for latitude:

Property: Y Coordinate of Point

Use coordinate system of the data source

Units: Decimal Degrees


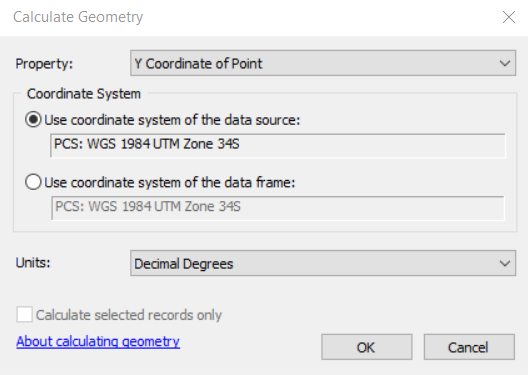


And the following information for longitude:

Property: X Coordinate of Point

Use coordinate system of the data source

Units: Decimal Degrees


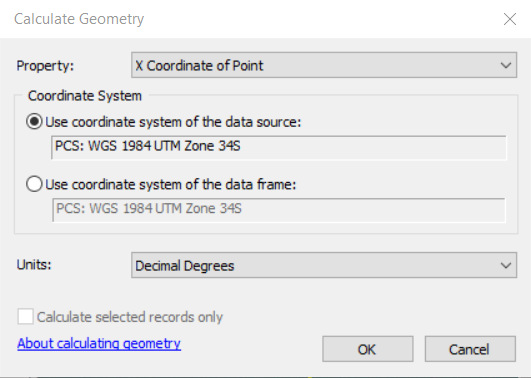


1. Make sure to save your edits, frequently, by selecting the black arrow pointing down next to “Editor”, and selecting “Save Edits”.


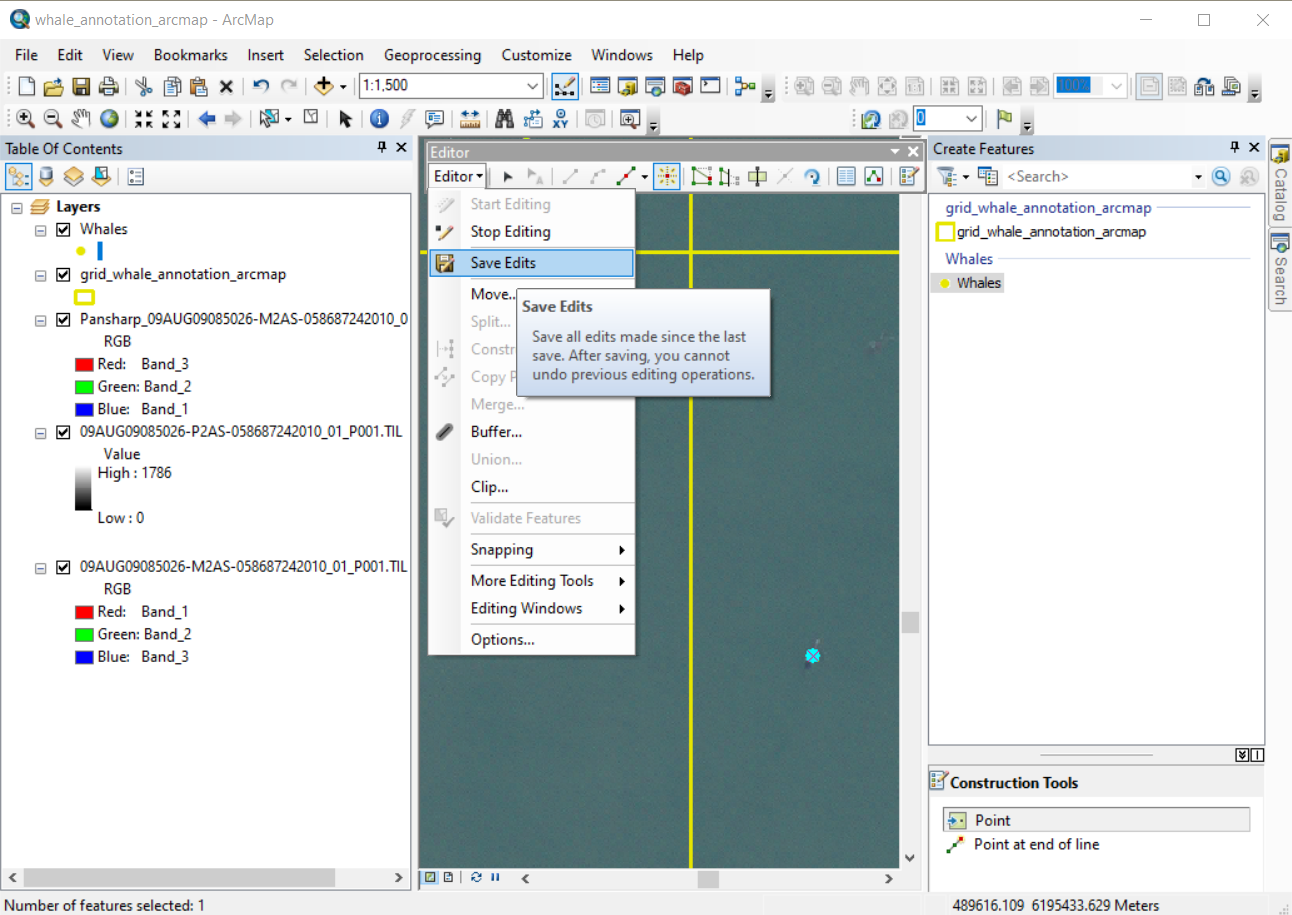


1. If you wish to leave this session, make sure to stop the edition session by selecting the black arrow pointing down next to “Editor”, and selecting “Stop Edits”.


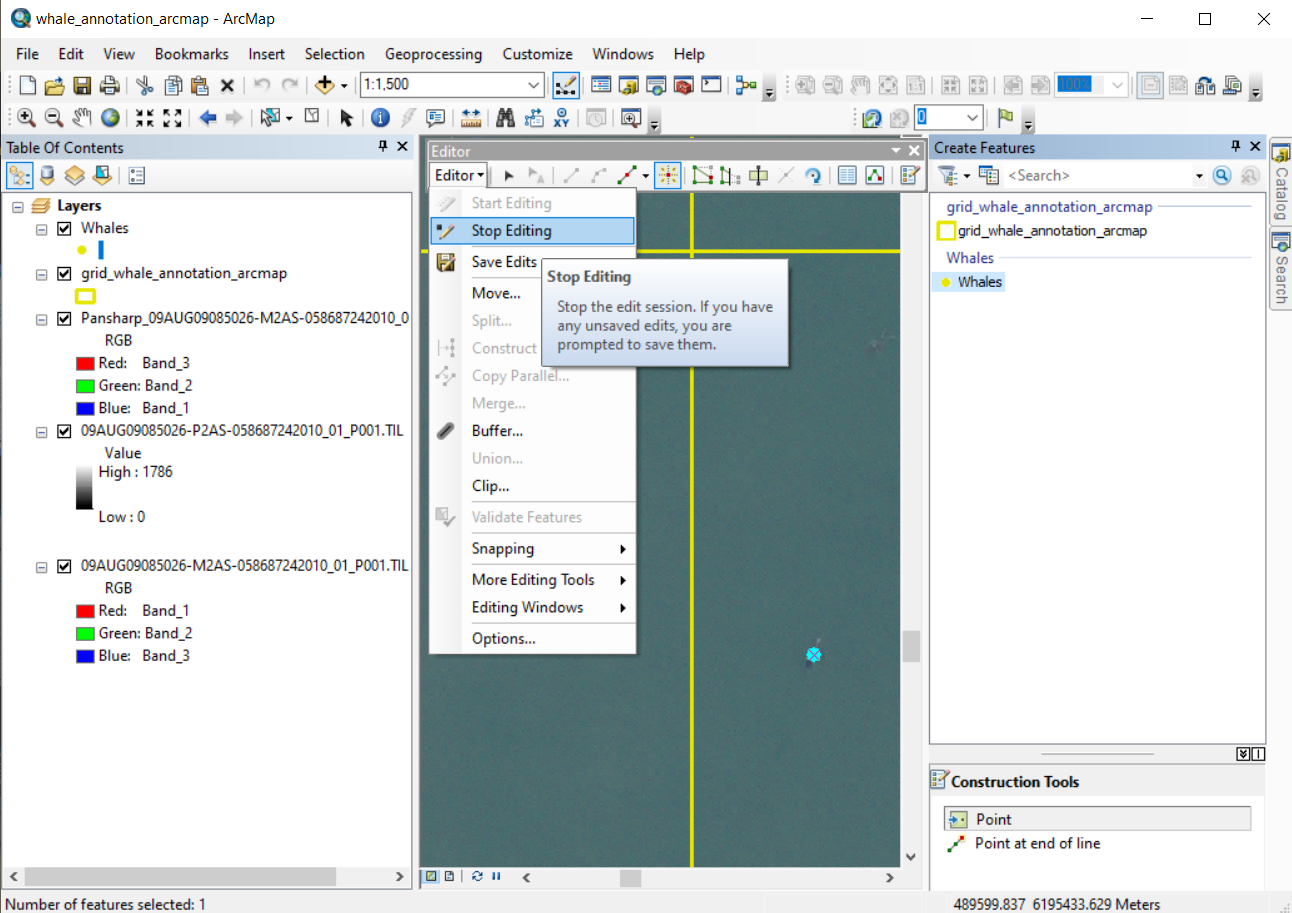


**Table 3.** Description of each Field contained in the attribute table with information on how to fill in the information for each Field.

| Field | Description | Unit | Value | Format |
| --- | --- | --- | --- | --- |
| observer | Name of person reviewing the image. | None | Letters | *e.g.* Hannah Smith |
| location | Name of the location where the satellite image was captured. | None | Letters | *e.g.* Laguna San Ignacio  *e.g.* Cape Cod |
| satellite | Name of the satellite that captured the image. | None | Letters and numbers | *e.g.* Worldview-3 |
| gsd_cm | The ground sampling distance (the distance between the center points of each pixel), which can be found in the metadata, by right clicking on the panchromatic file and selecting “Properties” then the “Source” tab. | Centimeter | Numbers | *e.g.* 3 |
| img_id | Unique identification that the satellite imagery provider assigns to each image. With Maxar, this corresponds to the catalog ID. | None | Letters and numbers | *e.g.* 10400ED2959 |
| img_date | Date the image was captured. | Day, month, year | Numbers | dd/mm/yyyy |
| Img_time | Time the image was captured. | Hours and minutes | Numbers | hh:mm |
| prod_type | The product type indicates the level of pre-processing an image has gone through when it was acquired from the satellite imagery provider, such as projection. See Table 1 for the various product type offered by the main VHR satellite imagery providers. | None | Letters and numbers | *e.g.* Standard 2A |
| sea_state | Sea state adapted from Figure 4 in Bamford et al (2020).  **1= Good** (minimal swell, no white caps, no wavelets)  **2 = Moderate** (minimal swell, sparse white caps, few wavelets)  **3 = Average** (slight swell, intermittent wavelets, no or very few white caps)  **4 = Sub-average** (medium swell, apparent waves, several white caps).  **5 = Poor** (significant swell, directional surface wind, large wave, several white caps) | None | 1 to 5 | *e.g.* 1 |
| cloud_cov | Cloud cover for the whole image, using the aviation system:  0 = SKC (sky clear)  1-2 = FEW (traces)  3-4 = SCT (scattered)  5-7 = BKN (BrOKen)  8 = OVC (Overcast) | OKta | 0 to 8 | *e.g.* 0 |
| cloud_th | Cloud thickness for the clouds present in the image  1 = Thin (can see fairly well through the cloud)  2 = Medium thin (can see through but no clear view of the sea)  3 = Thick (can’t see through)  4 = mix of thin, medium, thick clouds | Number | 1 to 4 | *e.g.* 1 |
| glare | Proportion of glare in the whole image:  0 = None  1 = Mild  2 = Moderate  3 = Severe | Number | 1 to 3 | *e.g.* 1 |
| turbidity | Qualitative estimations of the level of turbidity:  1 = Non-turbid  2 = Moderate  3 = Turbid  4 = mix of turbid and non-turbid waters | Number | 1 to 4 | *e.g.* 1 |
| otr_env | Other environmental conditions that the observer think might limit the visibility of whales (*e.g.* dark image for polar regions from autumn to spring) | None | Letters | *e.g.* dark image |
| latitude | Latitude of the whale detection | Decimal degree | Numbers | *e.g.* 67.50 |
| longitude | Longitude of the whale detection | Decimal degree | Numbers | *e.g.* 12.70 |
| gcs | Geographical coordinate system, it can be found in the metadata. | None | Letters and numbers | *e.g.* WGS 1984 |
| projection | Projection applied to the image to remove distortion | None | Letters and numbers | *e.g.* WGS 1984 UTM S12 |
| sp_code | Species code for the species or the next higher taxonomic level, see Appendix 1 (Supplementary material 3) to help you decide, and Appendix 2 (Supplementary material 4) for the code to use. | None | See Appendix 2 (or Supplementary material 4 | *e.g.* *Eubalaena glacialis* |
| certainty | Certainty of the assignment of the species or the next higher taxonomic level. See Appendix 3 (Supplementary material 5) to help you decide.  1 = **Definite**: you are confident in your species determination (90-100% confidence)  2 = **Probable**: you think that your species determination is likely but you are not sure (60-90% confidence)  3 = **Possible**: you think that your species determination is possible but it is hard to tell (10-60% confidence) | None | 1 to 3 | *e.g.* 1 |
| body_color | Body color of the whale when at the surface (dorsally when viewed in VHR satellite imagery). | None | Letters | *e.g.* brownish gray |
| body_shp | Overall shape of the body excluding fluke and flippers. | None | Letters | *e.g.* streamlined, sleek ellipsoid |
| body_l | Maximum visible length between the tip of the head and the fluke with values ranging from calf size to maximum adult length. | Meters | Numbers | *e.g.* 12.4 |
| body_w | Body width, it is measured at the widest part of the body and perpendicular the body length. | Meters | Numbers | *e.g.* 2.0 |
| flipper | Forelimb used to stabilise and turn.  1 = Yes  2 = No  3 = Maybe | None | 1 to 3 | *e.g.* 1 |
| lg_flipper | Species specific – Humpback whale have long flippers, which are one third of the body length.  1 = Yes  2 = No  3 = Maybe | None | 1 to 3 | *e.g.* 1 |
| fluke | Tail used to generate thrust.  1 = Yes  2 = No  3 = Maybe | None | 1 to 3 | *e.g.* 1 |
| head_callo | Species specific – white head callosities for the species of the genus Eubalaena. White patches on top of the head.  1 = Yes  2 = No  3 = Maybe | None | 1 to 3 | *e.g.* 1 |
| wh_lr_jaw | Species specific – white right lower jaw for fin whales.  1 = Yes  2 = No  3 = Maybe | None | 1 to 3 | *e.g.* 1 |
| aft_breach | After breach, large white area left after a whale breached, or lobtailed, flipper-slapped.  1 = Yes  2 = No  3 = Maybe | None | 1 to 3 | *e.g.* 1 |
| bubble_net | Species specific – bubble net for humpback whales. One white spiral formed of several white circular patches, or several white spirals nested together.  1 = Yes  2 = No  3 = Maybe | None | 1 to 3 | *e.g.* 1 |
| contour | White line separating the part of the whale body that is above and below the sea surface (*e.g.*, when a whale is rolling its back or surfacing to breathe).  1 = Yes  2 = No  3 = Maybe | None | 1 to 3 | *e.g.* 1 |
| flukeprint | White circle left after whale dove or while swimming (Levy *et al.*, 2011).  1 = Yes  2 = No  3 = Maybe | None | 1 to 3 | *e.g.* 1 |
| wake | V-shaped white trail behind the animal.  1 = Yes  2 = No  3 = Maybe | None | 1 to 3 | *e.g.* 1 |
| blow | Vaporous whitish patch next to a whale, like fog.  1 = Yes  2 = No  3 = Maybe | None | 1 to 3 | *e.g.* 1 |
| mudtrail | Plume/cloud of substrate behind a whale.  1 = Yes  2 = No  3 = Maybe | None | 1 to 3 | *e.g.* 1 |
| sur_act_gr | Two or more whales rolling and touching at the surface.  1 = Yes  2 = No  3 = Maybe | None | 1 to 3 | *e.g.* 1 |
| travel_gr | Two or more cetaceans traveling together in the same direction and less than a few meters apart.  1 = Yes  2 = No  3 = Maybe | None | 1 to 3 | *e.g.* 1 |
| mc_pair | Mother-calf pair, observed when the calf next to the mother.  1 = Yes  2 = No  3 = Maybe | None | 1 to 3 | *e.g.* 1 |
| otr_gr | Other type of group, if not socialising or traveling.  1 = Yes  2 = No  3 = Maybe | None | 1 to 3 | *e.g.* 1 |
| defecation | 1 = Yes  2 = No  3 = Maybe | None | 1 to 3 | *e.g.* 1 |
| comment | Any other comment the observer would like to make about the specific detection | None | Letters | *e.g.* mother of the mother-calf pair |

# Joining annotations from multiple observers

We recommend that at least two observers should review the same satellite image(s) independently. Once each observer has annotated the images, the point shapefiles can be used to join the annotations.

# Creating bounding box

Here we are following the method from Cubaynes and Fretwell, 2022 in more detail.

## Centre the points in a middle of a pixel

1. Use “Extract by Mask” tool to select the pixel the point is on. You can either access the tool via the “ArcToolbox” icon
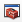
, or the “Search” icon
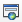
. Before using this tool, make sure the “Spatial Analyst” extension is enabled by selecting “Customize”, then “Extensions” and ensuring that the box next to “Spatial Analyst” is ticked.


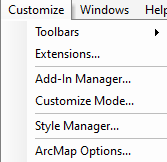


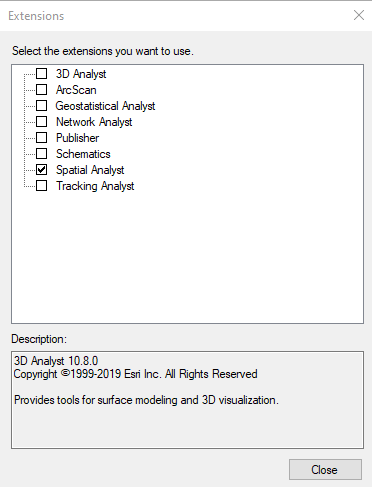


- 1. If using ArcToolbox, select “Spatial Analyst”, then select “Extraction”, then “Extract by Mask”.


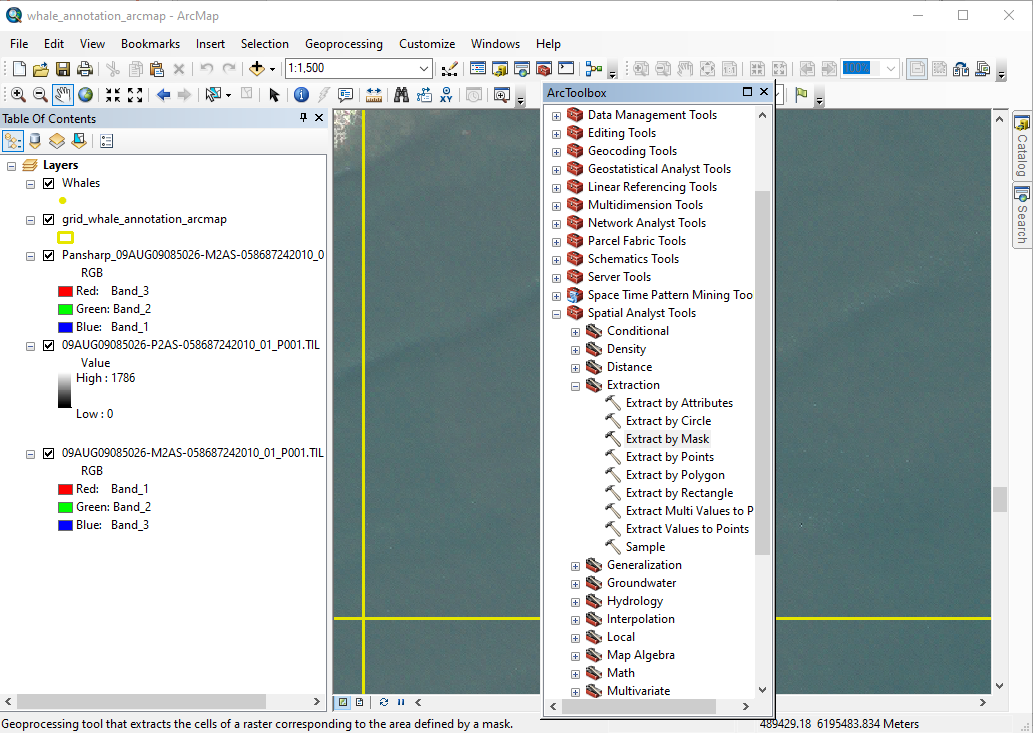


- 1. If using the “Search” icon, type in “extract by mask” and select the top option “Extract by Mask (Spatial Analyst)”.


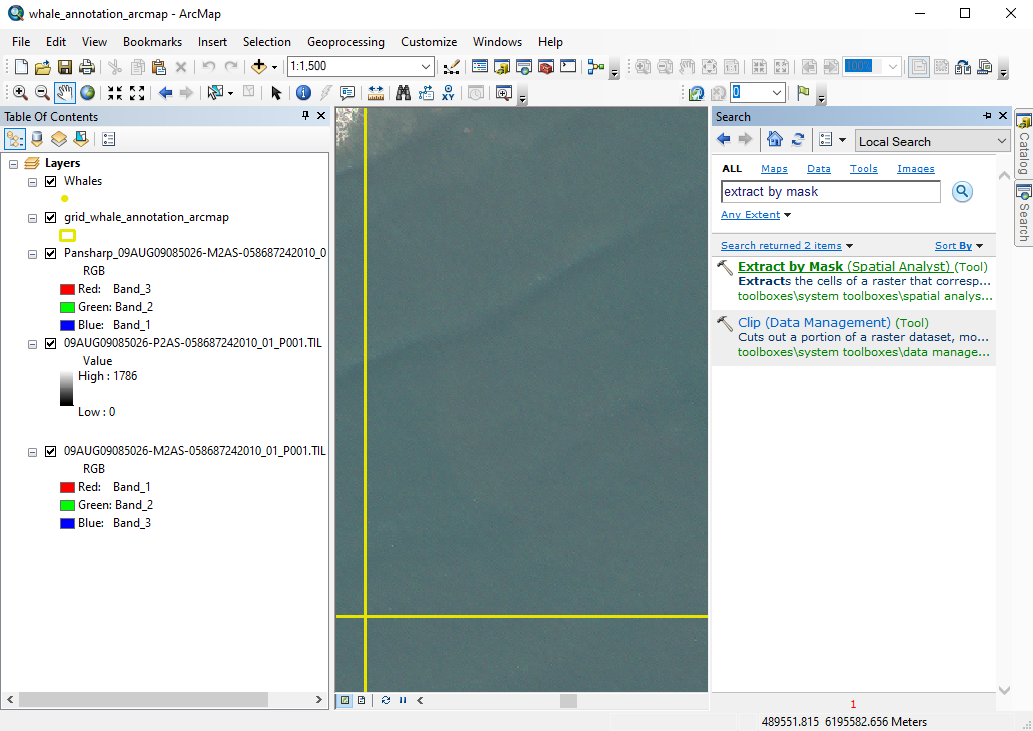


- 1. In the “Extract by Mask” window, fill in the following information, then select “OK”:

Input raster: the pansharpened file

Input raster or feature mask data: the point shapefiles with the joined whale annotations

Output raster: leave as it is, *i.e.* going to your default ArcGIS folder (if changing the destination of the output, the tool may fail).


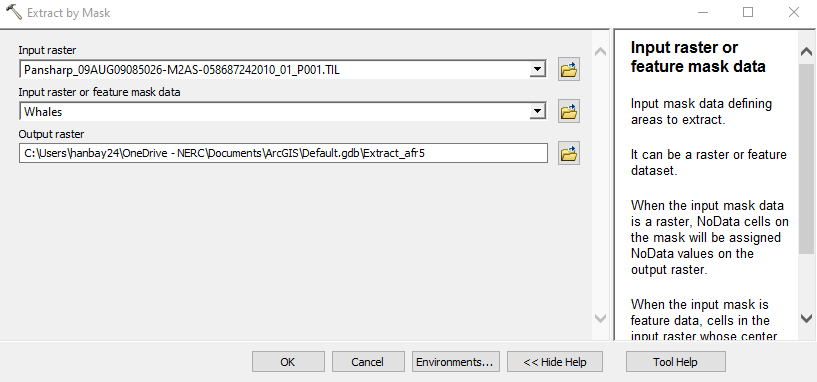


- 1. A new file will appear under the “Table of Contents”.


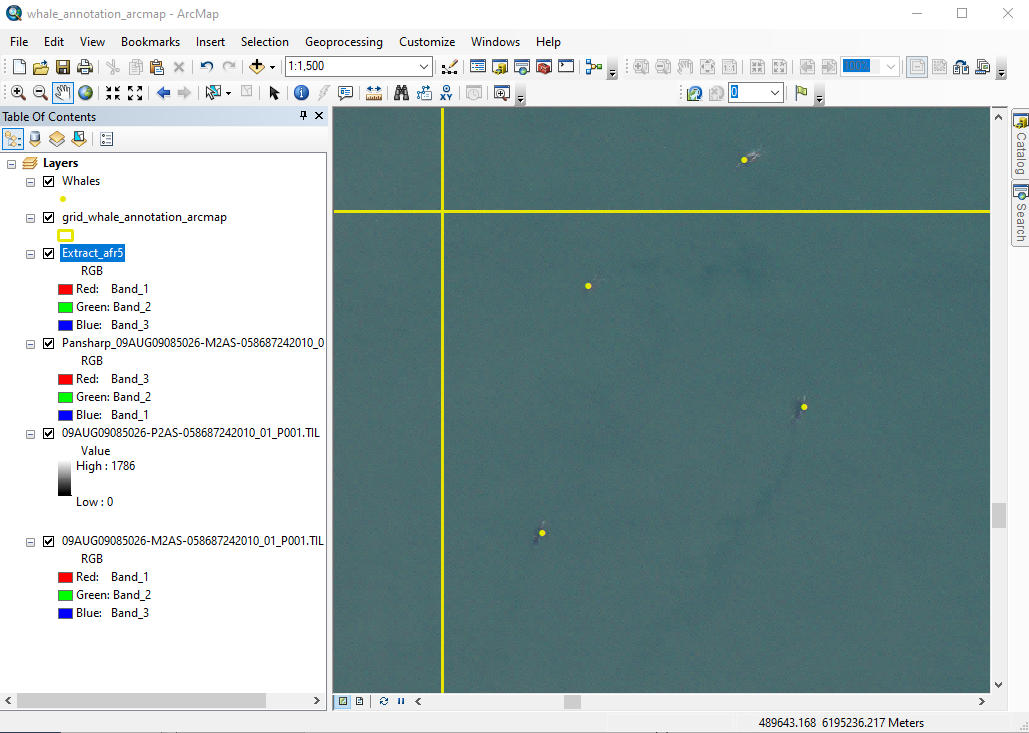


1. Use the “Raster to Point” tool to create a point centered on the pixel we have just extracted. You can either access the tool via the “ArcToolbox” icon
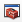
, or the “Search” icon
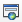
.
   1. If using ArcToolbox, select “Conversion”, then select “From Raster”, then “Raster to Point”.


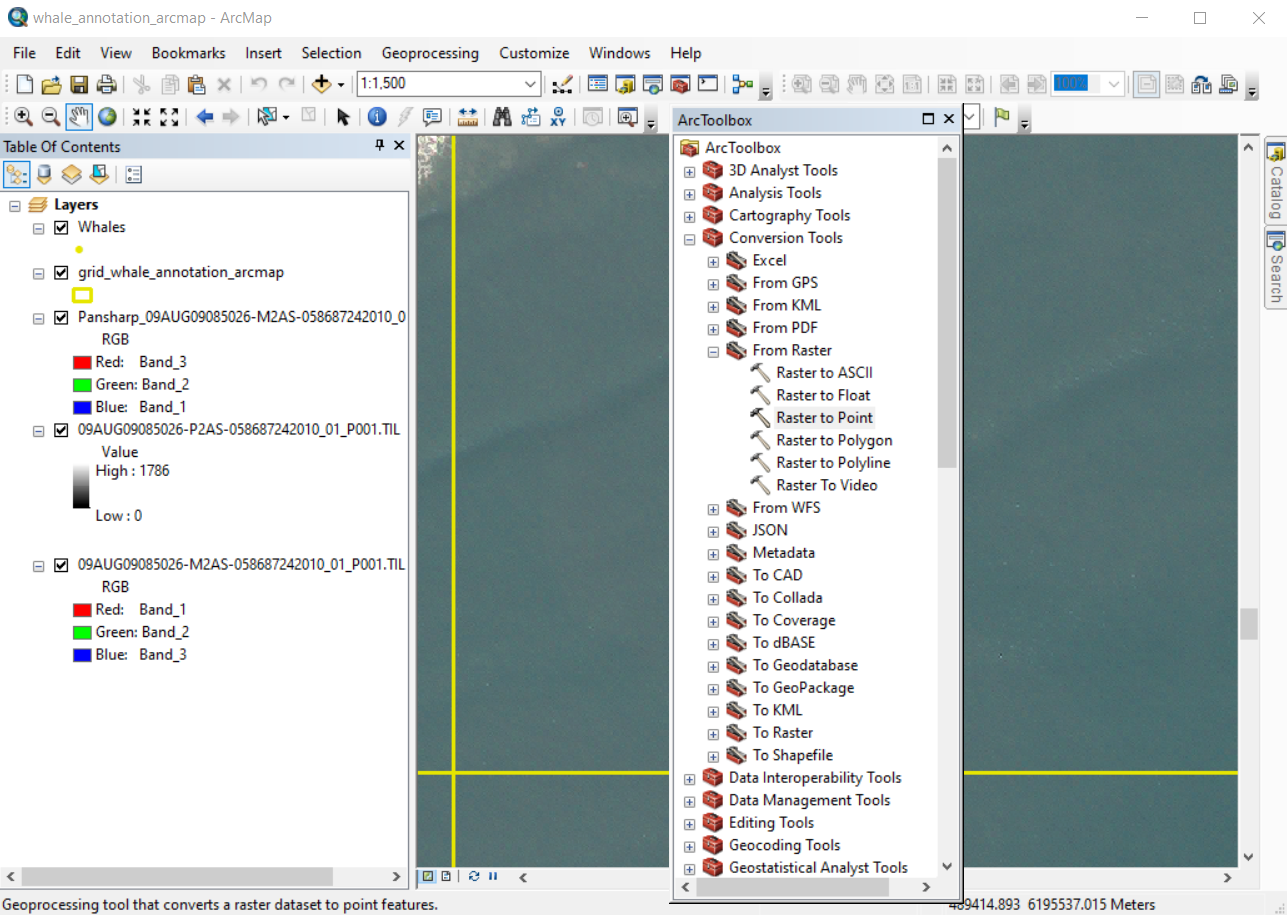


- 1. If using the “Search” icon, type in “raster to point” and select the “Raster to Point (Spatial Analyst)” tool.


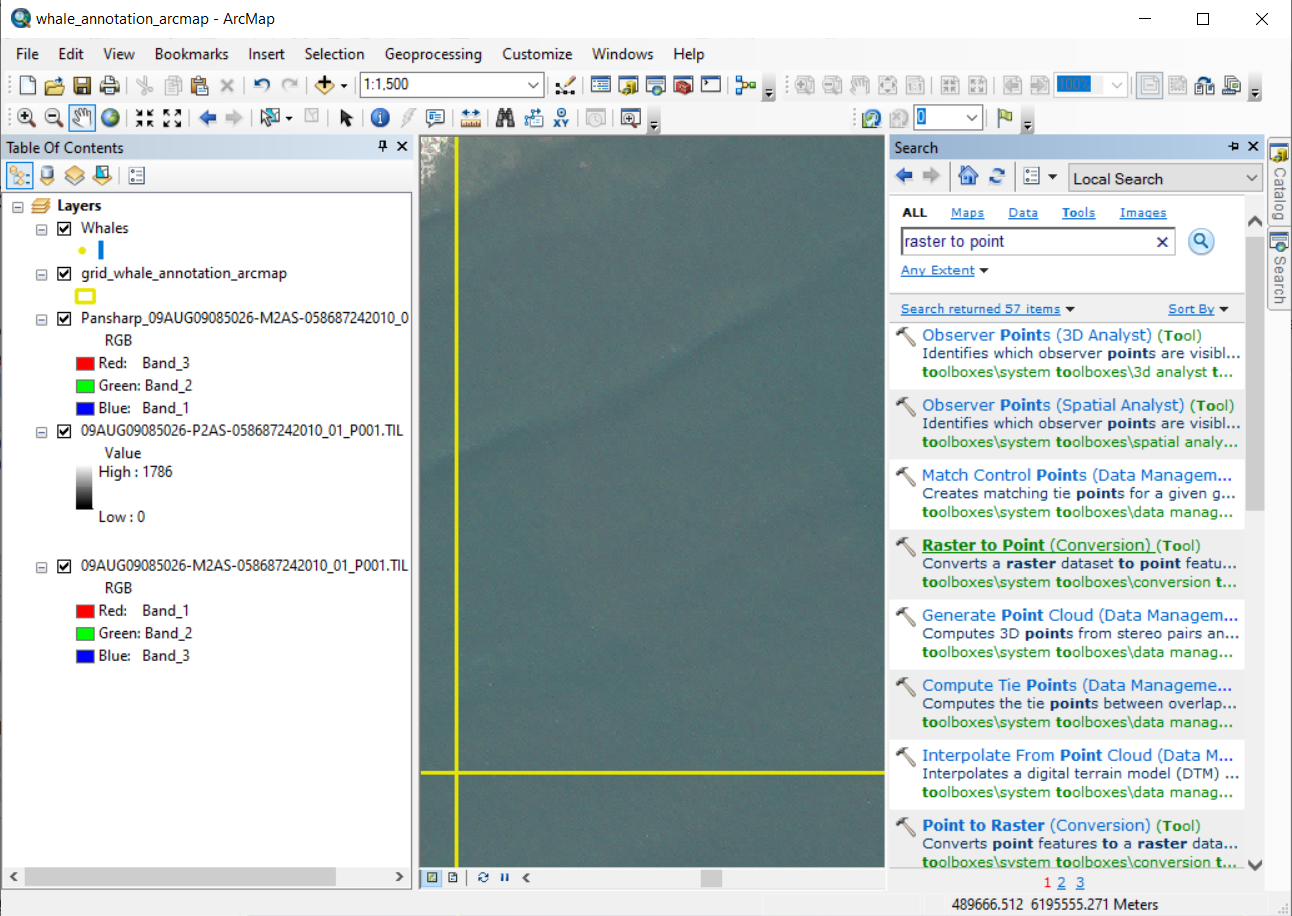


- 1. Under the “Raster to Point”, fill in the following information, then select “OK”:

Input raster: the file created in step 23.3

Field: Value

Output: leave as it is


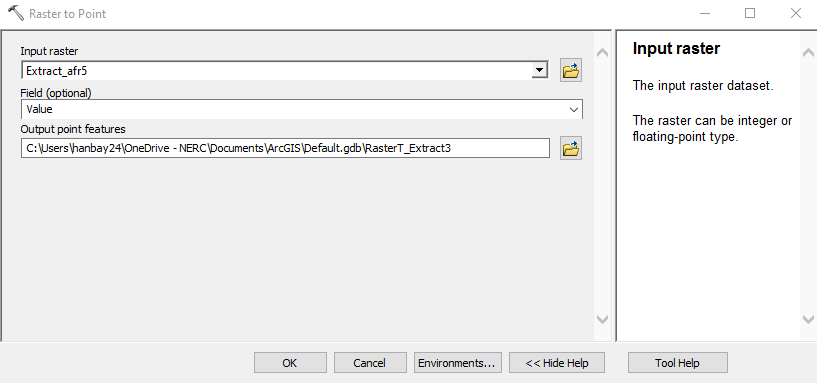


- 1. The newly created file will appear under the “Table of Contents”.


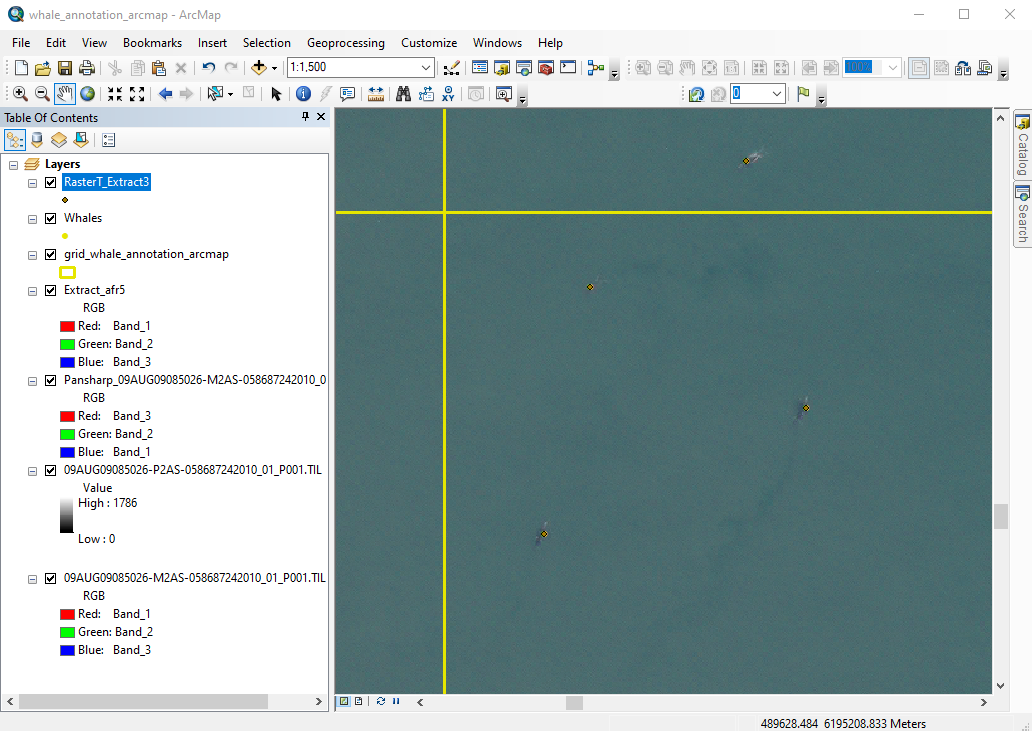


1. Use the “Spatial Join” tool, to attach the attribute table of the original point shapefile with the whale annotation to the newly created shapefile with centered points. You can either access the tool via the “ArcToolbox” icon
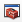
, or the “Search” icon
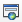
.
   1. If using ArcToolbox, select “Analysis Tool”, then select “Overlay”, then “Spatial Join”.


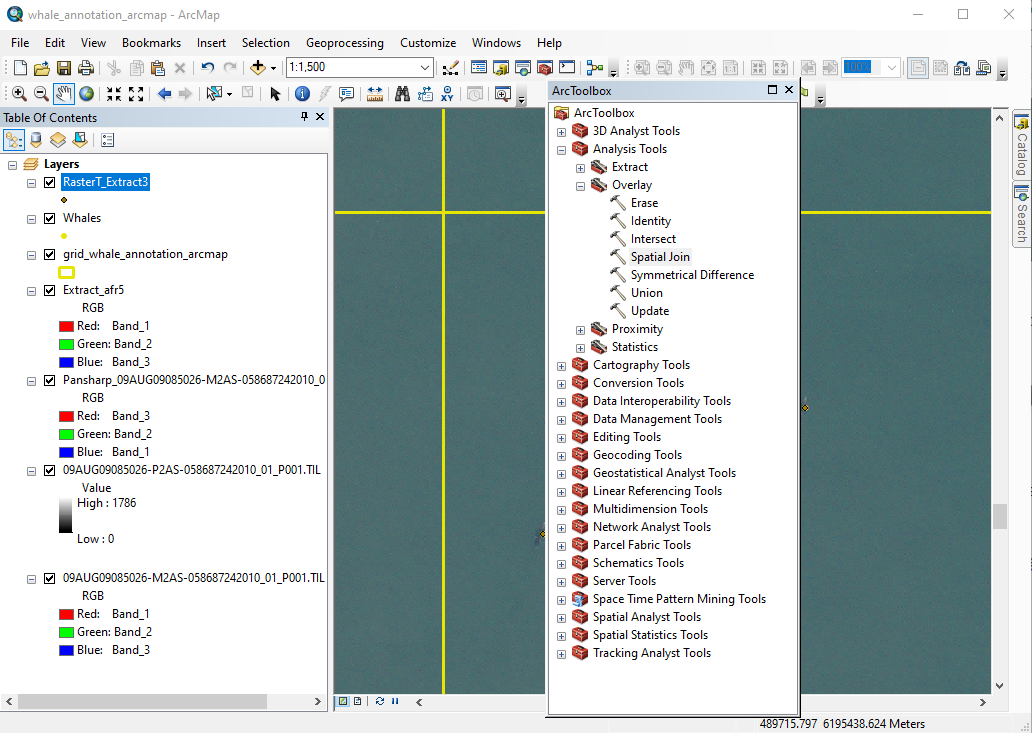


- 1. If using the “Search” icon, type in “spatial join” and select the “Spatial Join (Analysis)”.


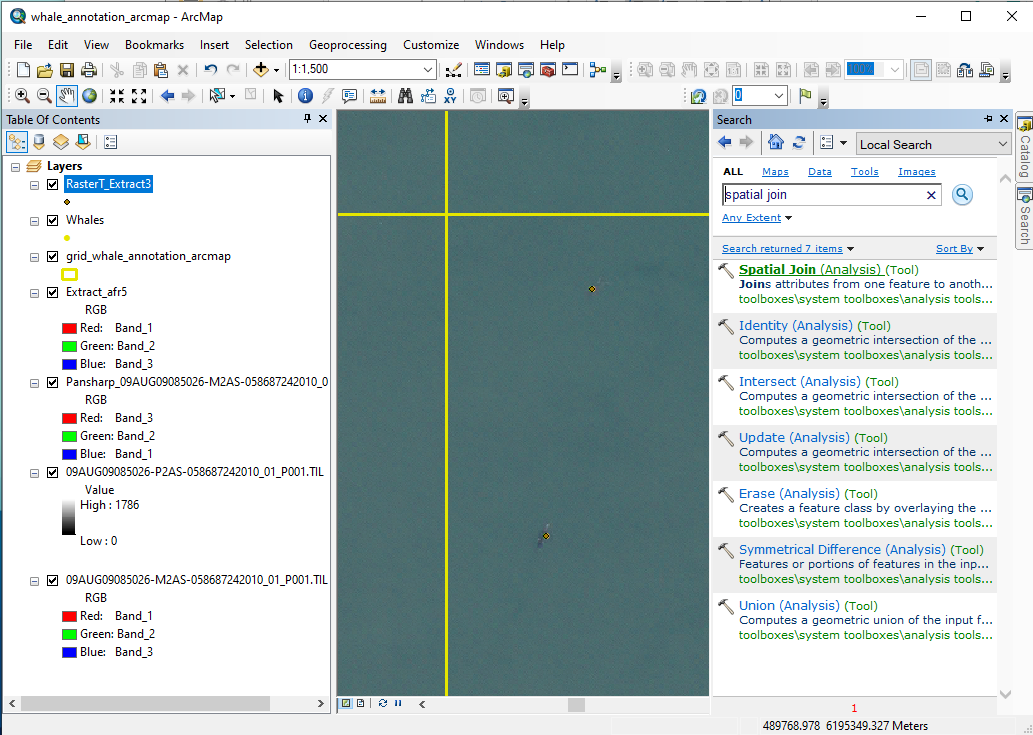


- 1. Under the “Spatial Join” window, fill in the following information, then select “OK”:

Target Features: the centered point shapefile created step 24.3.

Join Features: the point shapefile with the original whale annotation (uncentered).

Output Features Class: choose where to save it and name the file to be created.

Join Operation: JOIN_ONE_TO_MANY

Keep All Target Features: tick

Match Option: Closest

Search Radius: 0.5 Meters (here we are using a GeoEye-1 image which has a spatial resolution of 0.5m.


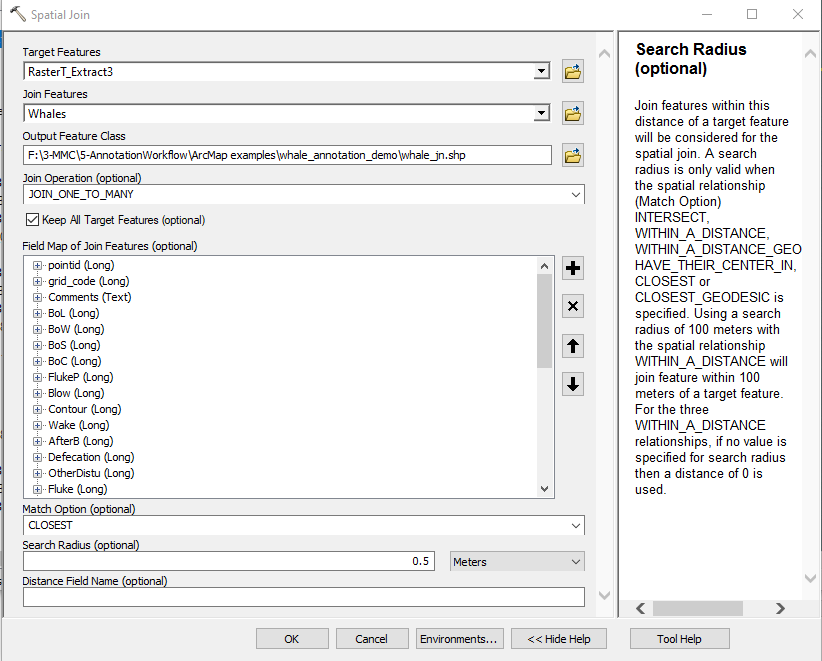


- 1. The newly created shapefile will appear in the “Table of Contents”.


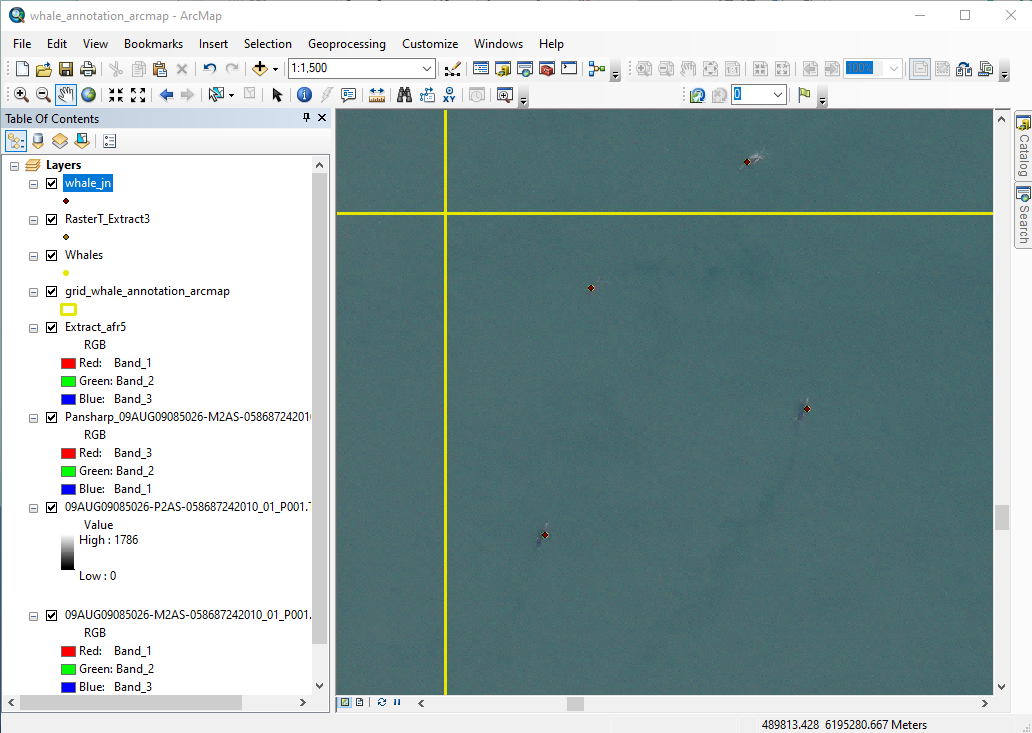


## Create a buffer around the centered points

1. Create a buffer around the centered points, by using the “Buffer” tool. You can either access the tool via the “ArcToolbox” icon
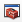
, or the “Search” icon
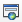
.
   1. If using ArcToolbox, select “Analysis Tool”, then select “Proximity”, then “Buffer”.


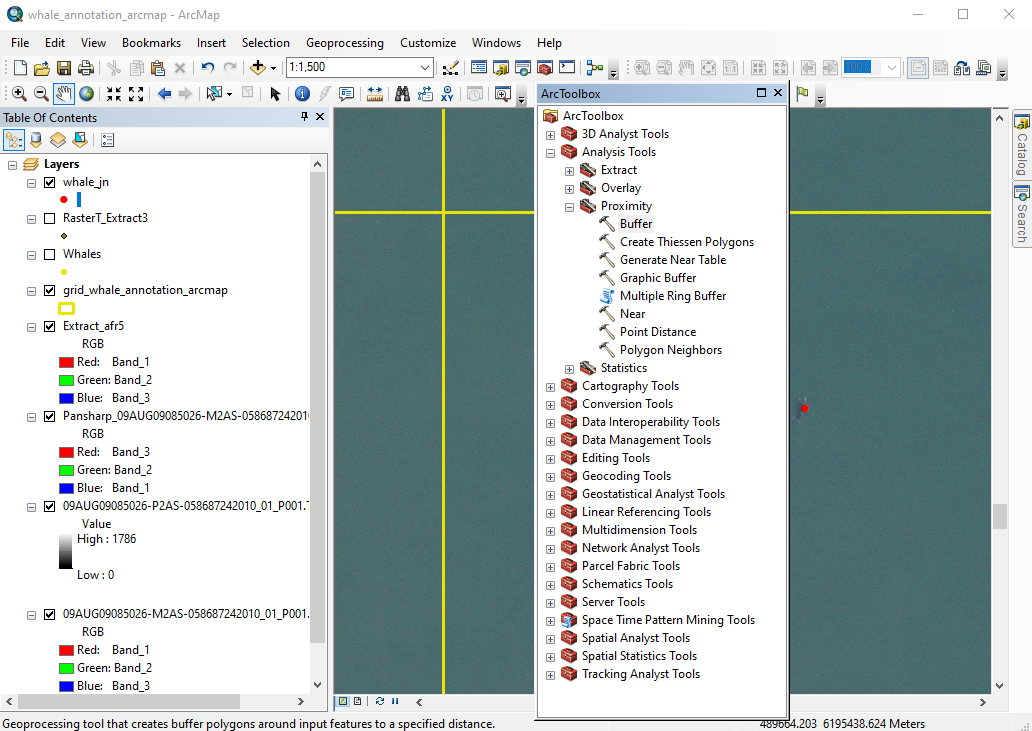


- 1. If using the “Search” icon, type in “buffer” and select the “Buffer (Analysis)”.


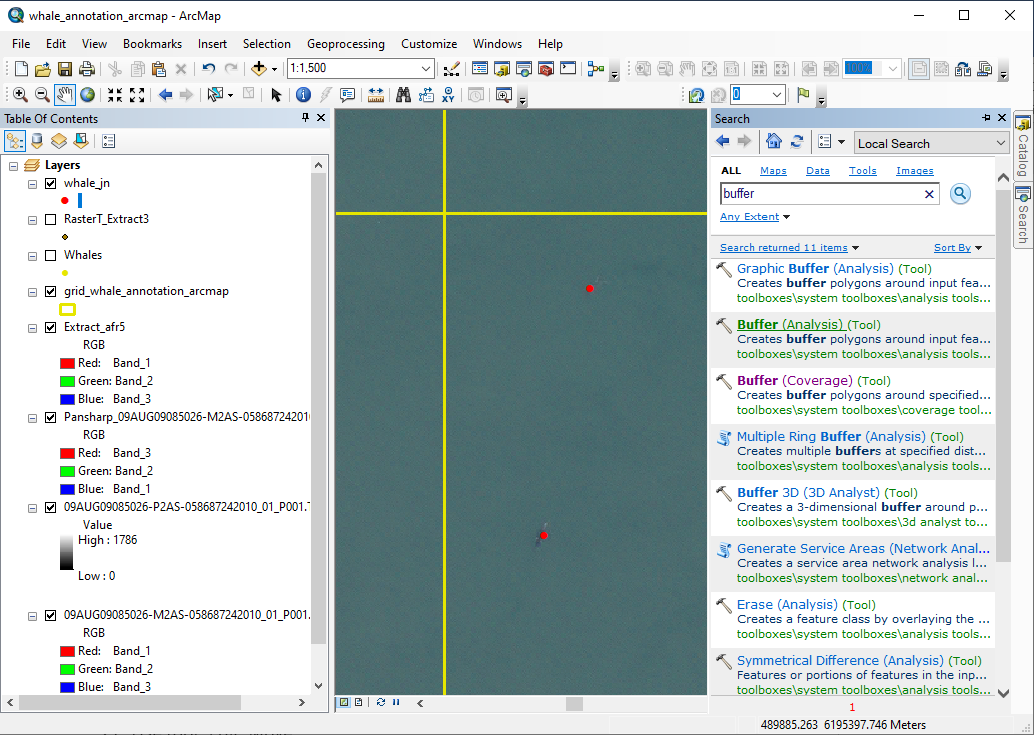


- 1. Under the “Buffer” window, fill the following information, then select “OK”:

Input Features: the point shapefile with the centered points and adjoining attribute table created in step 25.3

Output feature class: choose the location to save, and rename the file that is being created at this step.

Distance: tick linear unit and inform “32” Meters for a GeoEye-1 image, you can reduce or increase this size. A 32 buffer means that the whale point should be fully included within the buffer. For the bounding boxes to be useful to train machine learning systems, it is necessary that these boxes cover an even number of pixels and preferably a power of two, so we chose for our boxes to be 128 by 128 pixels. The width (or height of the bounding boxes, here 128 pixels) divided by the spatial resolution (50cm here), gives a diameter for the buffer of 64 m and a radius of 32 m.

Leave the rest as default.

- 1. The buffer shapefile will appear in the “Table of Contents” and you can visualize the buffers in the “Data View” window.

1. The buffers need to be shifted so they align with the border of the pixels at the top, bottom, left and right.
   1. Right click on the newly created buffer file, and select “Edit Features”, then select “Start Editing”.

- 1. Select all the buffers by zooming out to see the whole image and using the “Select Features” tool . Then click on the top left corner of the satellite image and drag to encompass all buffers.

- 1. Under the “Editor” toolbar, select the black arrow pointing down next to “Editor”, and select “Move”.

- 1. A new small window open, named “Delta X, Y”, fill in the following value for a GeoEye-1 Image if centering on the pansharpened image: 0.25 (i.e. the spatial resolution divided by 2). Then select “Enter”.

- 1. Remember to first save your edits and then stop the editing session. Select the black arrow pointing down next to “Editor” and select “Save Edits”, to save your edits; and select “Stop Editing”, to stop the editing session.

## Create a bounding box around the buffer

1. To create a box encompassing the buffer (bounding box), use the “Feature Envelope to Polygon” tool. You can either access the tool via the “ArcToolbox” icon , or the “Search” icon .
   1. If using ArcToolbox, select “Data Management”, then select “Proximity”, then “Feature Envelope to Polygon”.

- 1. If using the “Search” icon, type in “envelope” and select the “Feature Envelope to Polygon (Data Management)”.

- 1. Under the “Feature to Envelope” window, fill in the following information, then select “OK”:

Input Features: the buffer file

Output Feature Class: choose where to save the file to be created and name the file

Create multipart features: tick

- 1. The bounding box will now appear in the “Table of Contents” and be visible in the “Data View” window.

- 1. We recommend adding the following fields to the attribute table of the bounding box shapefile. Follow steps 10.3 to 10.5 to create new fields and add the fields listed in Table 4.

**Table 4.** Information necessary to fill the “Add Field” window, each row represents a different Field.

| **Name** | **Type** | **Field Properties** | **Description** |
| --- | --- | --- | --- |
| num_whale | Short integer | Precision: 0 | The number of whales within the bounding box. Sometimes two whales swimming nearby will be in each other’s bounding box. Even if only a portion of the whale is inside the box, it counts as one whale. |
| box_id | Text | Length: 50 | A unique ID for each bounding box. |
| box_size | Text | Length: 15 | The size of the bounding box, e.g. 128x128pixels |

- 1. Fill in the attribute table following the steps from steps 18 to 22. To fill in some information quicker, remember you can use the “Filed Calculator” tool (see step 20) using the information below.

TIP: to fill in the information of box_id quicker, especially if you have more than 10 boxes, you can enter the following in the “Field Calculator”: To get SRW_GoeEye-1_20170221_B1: write [sp_code]&"_"& [satellite]&"_"&"_"& [img_date]&"_"&"B"& [FID]

WARNING: ensure there is no space, otherwise it will not work.

# Creating image chips (PNG)

Due to licensing restrictions, the image chips cannot currently be shared in a .tif format; therefore, below we show how to export them in a .png format. If you wish to share your image chips, similar to Cubaynes and Fretwell, 2022, check the conditions with the satellite imagery provider, and the end user license agreement.

## Clipping the satellite image to the outline of the bounding boxes

1. Update the symbology of the pansharpened image (the image we wish to clip into several image chips).
   1. Right click on the image, and select “Properties”.

- 1. Under the “Properties” window, select the “Symbology” tab, and change the stretch to “None”, and ensure R, G, B is equal to 3, 2, 1.

1. Select all the bounding boxes by zooming out to see the whole image and using the “Select Features” tool. Then click on the top left corner of the satellite image and drag to encompass all boxes.

1. Open the “Image Analysis” window (under the main tab “Windows”) and select the pansharpened image.

1. In the “Image Analysis” window, under the “Processing” tab, select the “Clip” icon .

WARNING: make sure that the full extent of the satellite image is visible, as the clip tool only applies to what is visible in the “Data View” window.

1. The newly created clip file will automatically appear in the “Table of Contents”. Change the symbology to R, G, B, = 3, 2, 1 and ensure “Stretch” is equal to “None”.

## Exporting the clipped tif file as a png file

1. In the “Image Analysis” window, select the newly created clipped image, then under the “Processing” tab select the “Export” icon .

1. Fill in the newly opened “Export” window as follow:

Leave the “Extent” and “spatial Reference” as they are

Select “Use Renderer, and ensure “Force RGB” is selected too

Format: PNG

Name: choose the name for the png file to be created

No Data: 0 (as otherwise the output of the next step, using the split tool will be black image chips)

1. Under the “Table of Contents”, right click on the newly created image, select “Properties”. Under the tab “Symbology”, ensure the “Stretch” is “None”, and R, G, B, equals 1, 2, 3.

## Create multiple image chips

1. Use the “Split Raster” tool to split the png image into several image chips. You can either access the tool via the “ArcToolbox” icon , or the “Search” icon .
   1. If using ArcToolbox, select “Data Management”, then select “Raster”, then select “Raster Processing”, finally “Split Raster”.

- 1. If using the Search tool, type “split raster”, then select “Split Raster (Data Management)”.

- 1. Under the “Split Raster” window, fill in the following information:

Input Raster: the png image chips created in steps 34 to 35.

Output folder: choose the location where you wish to save the several image chips.

Output Basename: we recommend using the same as you entered in the box_id (minus the FID number).

Split Method: POLYGON_FEATURES

Output Format: PNG

Resampling method: NEAREST

Split Polygon Feature Class: select the bounding box shapefile you created in step 28.

Other Options> Overlap: 1000

Other Options> Units of Output Raster Size and Overlap: PIXELS

# Acknowledgments

This research was possible thanks to the Marine Mammal Commission for their financial support of the project MMC21-043. This study represents a contribution of the Ecosystems component of the British Antarctic Survey, funded by the Natural Environment Research council (NERC). The aerial images of cetaceans were collecting under the following permits: MMPA 17355, MMPA 21371, MMPA 775-1875, MMPA 775-1600.

# References

Airbus (2022) ‘Pleiades imagery user guide’, p. 114. Available at: https://www.intelligence-airbusds.com/en/8718-user-guides.

Cubaynes, H. C. *et al.* (2019) ‘Whales from space: Four mysticete species described using new VHR satellite imagery’, *Marine Mammal Science*, 35(2), pp. 466–491. doi: 10.1111/mms.12544.

Cubaynes, H. C. and Fretwell, P. T. (2022) ‘Whales from space dataset, an annotated satellite image dataset of whales for training machine learning models’, *Scientific Data*, 9, p. 245. doi: 10.1038/s41597-022-01377-4.

Jefferson, T. A., Webber, M. A. and Pitman, R. L. (2008) ‘Marine Mammals of the World: A Comprehensive Guide to their Identification’, *Aquatic Mammals*, 35(3), pp. 414–415. doi: 10.1578/AM.35.3.2009.414.

Larrat, S. and Lair, S. (2022) ‘Body condition index in beluga whale (Delphinapterus leucas) carcasses derived from morphometric measurements’, *Marine Mammal Science*, 38, pp. 274–287. doi: 10.1111/mms.12855.

Levy, R. *et al.* (2011) ‘A theory for the hydrodynamic origin of whale flukeprints’, *International Journal of Non-Linear Mechanics*, 46(4), pp. 616–626. doi: 10.1016/j.ijnonlinmec.2010.12.009.

Maxar Technologies (2022) *Core imagery product information*. Available at: https://securewatchdocs.maxar.com/en-us/Orders/Orders_ProductInfo.htm#CoreImageryProductInformation.

Planet (2022) ‘Planet imagery product specifications’, p. 101. Available at: https://assets.planet.com/docs/Planet_Combined_Imagery_Product_Specs_letter_screen.pdf.

Williams, T. M., Noren, S. R. and Glenn, M. (2011) ‘Extreme physiological adaptations as predictors of climate-change sensitivity in the narwhal, Monodon monoceros’, *Marine Mammal Science*, 27(2), pp. 334–349. doi: 10.1111/j.1748-7692.2010.00408.x.

Woodward, B. L., Winn, J. P. and Fish, F. E. (2006) ‘Morphological specializations of baleen whales associated with hydrodynamic performance and ecological niche’, *Journal of Morphology*, 267, pp. 1284–1294. doi: 10.1002/jmor.10474.

# Appendix 1: Species decision tree for cetaceans observed in VHR satellite imagery

**Important considerations when using the “Species decision tree for cetaceans observed in VHR satellite imagery”:**

- Figure S1.1 only includes the species that have confidently been observed in satellite imagery, which on 16^th^ June 2022 includes: narwhal (*Monodon monoceros*), beluga (*Delphinapterus leucas*), Eubalaena spp., fin whale (*Balaenoptera physalus*), humpback whale (*Megaptera novaeangliae*), and gray whale (*Eschrichtius robustus*).
- Biogeography: the location of the image will play an important role in determining the species, particularly for Eubalaena spp. Therefore, the “Species decision tree for cetaceans previously observed in VHR satellite imagery” needs to be used alongside known distribution map. For example narwhals are only found in the Arctic.
  - Suggested references: IUCN Red List (www.iucnredlist.org) and the Encyclopedia of Marine Mammals (https://www.sciencedirect.com/boOK/9780128043271/encyclopedia-of-marine-mammals)
- Full body here refers to seeing the head to at least the peduncle of the potential whale-object

**Figure S1.1.** Species decision tree for cetaceans observed in VHR satellite imagery.

# Appendix 2: Species code

Table S2.1. List of species codes for cetaceans that can be identified in very high-resolution satellite images.

| **sp_code** | **Scientific Name** | **Common Name** |
| --- | --- | --- |
| BELU | *Delphinapterus leucas* | Beluga |
| NAR | *Monoceros monoceros* | Narwhal |
| RIWH | *Eubalaena spp.* | Right whale |
| FIWH | *Balaenoptera physalus* | Fin whale |
| HUWH | *Megaptera novaeangliae* | Humpback whale |
| GRWH | *Eschrichtius robustus* | Gray whale |
| UNBA | NA | Unidentified Balaenoptera |
| UNWH | NA | Unidentified whale |
| UNDO | NA | Unidentified dolphin |
| UNFE | NA | Unidentified feature |

# Appendix 3: Assigning a certainty level

**Steps to Classify Whale Species in VHR Satellite Imagery and Assign Certainty Levels:**

1. **Background Research**

Before reviewing a very high-resolution (VHR) satellite image for the presence of whales, examine the images in Tables S3.1-4 to familiarize yourself with what the different species look like. Where VHR satellite imagery is not available for a particular species, we have provided examples from higher resolution aerial imagery captured with DSLR camera, but bear in mind that satellite images will appear more blurry or grainy. The species list is not exhaustive, so if there are other large animals in your study area, familiarise yourself with examples of these species in aerial images.

1. **Species Determination**

Once you have reviewed the available material and detected a potential whale in a satellite image, use the Species Decision Tree (Appendix 1 or Supplementary material 3) to assign a species or the next higher taxonomic level.

1. **Certainty**

Assign a certainty level based on the list of cues in Tables S3.1, S3.2, and S3.3, and examples of various species at various spatial resolution (Table S3.4):

- - **Definite**: you are confident in your species determination (90-100%)
  - **Probable**: you think that your species determination is likely but you are not sure (60-90%)
  - **Possible**: you think that your species determination is possible but it is hard to tell (10-60%)

**Table S3.1.** Individual characteristics to help confirm species identification based on (Woodward, Winn and Fish, 2006; Jefferson, Webber and Pitman, 2008; Williams, Noren and Glenn, 2011; Larrat and Lair, 2022). Examples in very high-resolution satellite imagery and aerial images are given. Grayed-out cells indicate no imagery was available

| **Cue** | **Description** | **Narwhal** | **Beluga** | **Gray whale** | **Fin whale** | **Humpback whale** | **Eubalaena spp.** |
| --- | --- | --- | --- | --- | --- | --- | --- |
| Body coloration | Color at the surface (dorsally when viewed in VHR satellite imagery) | White to dark gray | White to dark gray | Brownish gray to light gray | Black to dark brownish gray with a white right jaw | Black or dark gray with dark or white flipper | Black body with white head callosities |
| Body shape | Overall shape of the body excluding fluke and flippers | Elongated ellipsoid slightly stockier than belugas and with a rounder head | Elongated ellipsoid (like a grain of rice) | If full body visible: Robust, slim ellipsoid with a round tip of the head; if full body not visible: ellipsoid; if only head: circular (*e.g.*, when spy-hopping) or triangular with rounded angle | If full body visible: streamlined, sleek ellipsoid with a pointy V-shaped head; if full body not visible: ellipsoid; if only head: triangular with rounded angle | If full body visible: Rotund ellipsoid with round head; if full body not visible: ellipsoid; if only head: circular (*e.g.*, when spy-hopping) or triangular with rounded angle | If full body visible: Rotund, stocky ellipsoid with round head; if full body not visible: ellipsoid; if only head: circular (*e.g.*, when spy-hopping) or triangular with rounded angle |
| Body length | Maximum visible length between the tip of the head and the fluke with values ranging from calf size to maximum adult length | 1.6 – 4.2 m | 1.6 – 5.5 m | 4.6 m - 15 m | 6 m - 27 m | 4 m - 18 m | 4 m - 18 m |
| Body width | It is measured at the widest part of the body and perpendicular the body length. | ≤0.82 m | ≤0.75 m | ≤ 2.2 m | ≤ 3.9 m | ≤ 3.2 m | ≤ 3.3 m |
| Flipper – VHR | Forelimb used to stabilise and turn. |  |  |  |  | *See below “Long flipper”* |  |
| Flipper - Aerial |  |  |  |  |  |  |  |
| Long Flipper - VHR | Species specific – Humpback whale flippers are one third of the body length. | *NA* | *NA* | *NA* | *NA* |  | *NA* |
| Long flipper - Aerial |  |  |  |  |  |  |  |
| Fluke - VHR | Tail used to generate thrust. |  |  |  |  |  |  |
| Fluke - Aerial |  |  |  |  |  |  |  |
| White head callosities - VHR | Species specific – only reported for the species of the genus Eubalaena.  White patches on top of the head. | *NA* | *NA* | *NA* | *NA* | *NA* |  |
| White head callosities - Aerial |  |  |  |  |  |  |  |
| White right lower jaw - VHR | Species specific – only reported for fin whales.  White coloration of the lower right jaw. | *NA* | *NA* | *NA* |  | *NA* | *NA* |
| White right lower jaw - Aerial |  |  |  |  |  |  |  |

**Table S3.2.** Behavioural cues indicating the presence of whales related to sea surface disturbance. Examples in very high-resolution satellite imagery and aerial images are given. Grayed-out cells indicate no imagery was available

| **Cue** | **Description** | **Narwhal** | **Beluga** | **Gray whale** | **Fin whale** | **Humpback whale** | **Eubalaena spp.** |
| --- | --- | --- | --- | --- | --- | --- | --- |
| After-breach - VHR | Large white area left after a whale breached, or lobtailed, flipper-slapped. |  |  |  |  |  |  |
| After-breach - Aerial |  |  |  |  |  |  |  |
| Bubble net – VHR | Species specific - only reported for Humpback whales  One white spiral formed of several white circular patches, or several white spirals nested together. | *NA* | *NA* | *NA* | *NA* |  | *NA* |
| Bubble net – Aerial |  |  |  |  |  |  |  |
| Contour – VHR | White line separating the part of the whale body that is above and below the sea surface (*e.g.*, when a whale is rolling its back or surfacing to breathe). |  |  |  |  |  |  |
| Contour - Aerial |  |  |  |  |  |  |  |
| Flukeprint – VHR | White circle left after whale dove or while swimming (Levy *et al.*, 2011). |  |  |  |  |  |  |
| Flukeprint – Aerial |  |  |  |  |  |  |  |
| Wake – VHR | V-shaped white trail behind the animal. |  |  |  |  |  |  |
| Wake - Aerial |  |  |  |  |  |  |  |

**Table S3.3.** Other cues indicating the presence of whales (not related to sea surface disturbance or cetaceans’ body). Examples in very high-resolution satellite imagery and aerial images are given. Grayed-out cells indicate no imagery was available

| **Cue** | **Description** | **Narwhal** | **Beluga** | **Gray whale** | **Fin whale** | **Humpback whale** | **Eubalaena spp.** |
| --- | --- | --- | --- | --- | --- | --- | --- |
| Blow – VHR | Vaporous whitish patch next to a whale, similar looking to fog. |  |  |  |  |  |  |
| Blow – Aerial |  |  |  |  |  |  |  |
| Mudtrail – VHR | Plume/cloud of substrate behind a whale. |  |  |  |  |  |  |
| Mudtrail - Aerial |  |  |  |  |  |  |  |
| Surface active group - VHR | Two or more whales rolling and touching at the surface. |  |  |  |  |  |  |
| Surface active group – Aerial |  |  |  |  |  |  |  |
| Travelling group - VHR | Two or more cetaceans travelling together in the same direction and less than a few meters apart. |  |  |  |  |  |  |
| Travelling group - Aerial |  |  |  |  |  |  |  |
| Mother-calf pair - Aerial | When a calf is in the close proximity of an adult whale. |  |  |  |  |  |  |
| Mother-calf pair - VHR |  |  |  |  |  |  |  |
| Defecation – VHR | Trail of colored clouds behind animal. |  |  |  |  |  |  |

**Table S3.4.** Examples of narwhal, beluga, gray whale, fin whale, humpback whale and Eubalaena spp. in 30 cm and 50 cm very high-resolution (VHR) satellite imagery, and in aerial images. Grayed-out cells indicate no imagery was available.

|  | **Narwhal** | **Beluga** | **Gray whale** | **Fin whale** | **Humpback whale** | **Eubalaena spp.** |
| --- | --- | --- | --- | --- | --- | --- |
| 30cm VHR satellite imagery |  |  |  |  |  |  |
| 50 cm VHR satellite imagery |  |  |  |  |  |  |
| Aerial images |  |  |  |  |  |  |
