# Supplementary material for: Annotating very high-resolution satellite imagery: A whale case study
Source: MethodsX. 2023 Jan 25;10:102040. doi: 10.1016/j.mex.2023.102040 (PMC9923222; doi:10.1016/j.mex.2023.102040)
Supplement: Supplementary materiel 2 — A detailed step-by-step workflow to annotate whales in VHR satellite images and create points, bounding boxes, and image chips data sets using ESRI ArcGIS Pro 2.5. [file mmc2.zip › Supplementary2_ArcGIS_Pro.docx]

Supplementary material 2: User Guide to Annotating Cetaceans in VHR Satellite Imagery

Guidance for ArcGIS Pro 2.5

Hannah Cubaynes^1^ (Editor)

**Contributing Authors:**

Penny Clarke^1,2^, Christin Khan^3^, Kim Goetz^4^, Peter Fretwell^1^, Daniel Cholewiak^3,^ Kathleen Leonard^4^, Tyler Aldrich^3^

**Affiliations:**

^1^British Antarctic Survey, High Cross, Madingley Road, Cambridge, CB3 0ET, United Kingdom

^2^School of Engineering, The University of Edinburgh, Sanderson Building, Robert Stevenson Road, The King’s Buildings, Edinburgh, EH9 3FB, United Kingdom

^3^Northeast Fisheries Science Center, National Marine Fisheries Service, NOAA, Woods Hole, Massachusetts, United States

^4^Marine Mammal Laboratory, Alaska Fisheries Science Center, National Marine Fisheries Service, NOAA, Seattle, Washington, United States

Contents

[List of Tables iv](#_Toc118288857)

[Key Terminology v](#_Toc118288858)

[Summary 1](#_Toc118288859)

[Setting Up 2](#_Toc118288860)

[Pre-processing 11](#_Toc118288861)

[Projection 11](#_Toc118288862)

[Top of atmosphere correction 13](#_Toc118288863)

[Pansharpening 13](#_Toc118288864)

[Preparing the Workspace 19](#_Toc118288865)

[Creating a grid 19](#_Toc118288866)

[Creating a point shapefile 32](#_Toc118288867)

[Preparing the attribute table 36](#_Toc118288868)

[Reviewing the Image 44](#_Toc118288869)

[Systematic scanning 44](#_Toc118288870)

[Scale 45](#_Toc118288871)

[Keeping track 45](#_Toc118288872)

[Annotating 48](#_Toc118288873)

[Placing points on top of whales 48](#_Toc118288874)

[Filling in the attribute table 50](#_Toc118288875)

[Joining annotations from multiple observers 70](#_Toc118288876)

[Creating bounding box 70](#_Toc118288877)

[Centre the points in a middle of a pixel 70](#_Toc118288878)

[Create a buffer around the centered points 81](#_Toc118288879)

[Create a bounding box around the buffer 89](#_Toc118288880)

[Creating image chips (PNG) 93](#_Toc118288881)

[Clipping the satellite image to the outline of the bounding boxes 93](#_Toc118288882)

[Exporting the clipped tif file as a png file 96](#_Toc118288883)

[Create multiple image chips 99](#_Toc118288884)

[Acknowledgments 102](#_Toc118288885)

[References 103](#_Toc118288886)

[Appendix 1: Species decision tree for cetaceans observed in VHR satellite imagery 105](#_Toc118288887)

[Appendix 2: Species code 107](#_Toc118288888)

[Appendix 3: Assigning a certainty level 108](#_Toc118288889)

# List of Tables

[**Table 1.** List of product type for the main VHR satellite imagery provider, Airbus (Airbus, 2022), Planet (Planet, 2022) and Maxar Technologies (Maxar Technologies, 2022). 12](#_Toc113271513)

[**Table 2.** Information necessary to fill the “Add Field” window, each row represents a different Field. 38](#_Toc113271514)

[**Table 3.** Description of each Field contained in the attribute table with information on how to fill in the information for each Field. 55](#_Toc113271515)

[**Table 4.** Information necessary to fill the “Fields” window, each row represents a different Field. 90](#_Toc113271516)

# Key Terminology

**Annotation:** the process of categorizing and labelling objects of interest (*e.g.* whales) within the satellite image either by placing points or bounding boxes along with associated metadata.

**Multispectral**: describe a colored image, where each pixels comprised of different values representing different color bands (*e.g.* red, green, and blue). Most very high-resolution (VHR) satellite images capture multispectral images with four (blur, green, red, near-infrared) or eight bands (coastal blue, blue, green, yellow, red-edge, red, near-infrared 1, near-infrared 2). For VHR satellites, the spatial resolution is lower for the multispectral image compared to the panchromatic image.

**Panchromatic**: describe a grayscale image, where each pixels comprised of one value. For very high-resolution satellites, the spatial resolution is higher for the panchromatic image compared to the multispectral image

**Pansharpened**: a pansharpened image is created by combining the high spectral resolution (color) of a multispectral image with the high spatial resolution of a panchromatic image, resulting in an image that is both high resolution and color.

**Pansharpening**: the process of combining the high spectral resolution (color) multispectral image with the high spatial resolution panchromatic image, to create a pansharpened image of both high resolution and color. Various algorithms exist to execute this transformation (*e.g.* Brovey, Gram-Schmidt).

# Summary

In this document, we share a step-by-step guide and our recommendations for annotating satellite images with the ultimate goal to build a dataset of examples of whales in satellite images using ArcGIS Pro 2.5.

# Setting Up

1. Open ArcGIS Pro.
2. Sign in using your credentials.
3. If annotating a new satellite image, select “Start without a templat
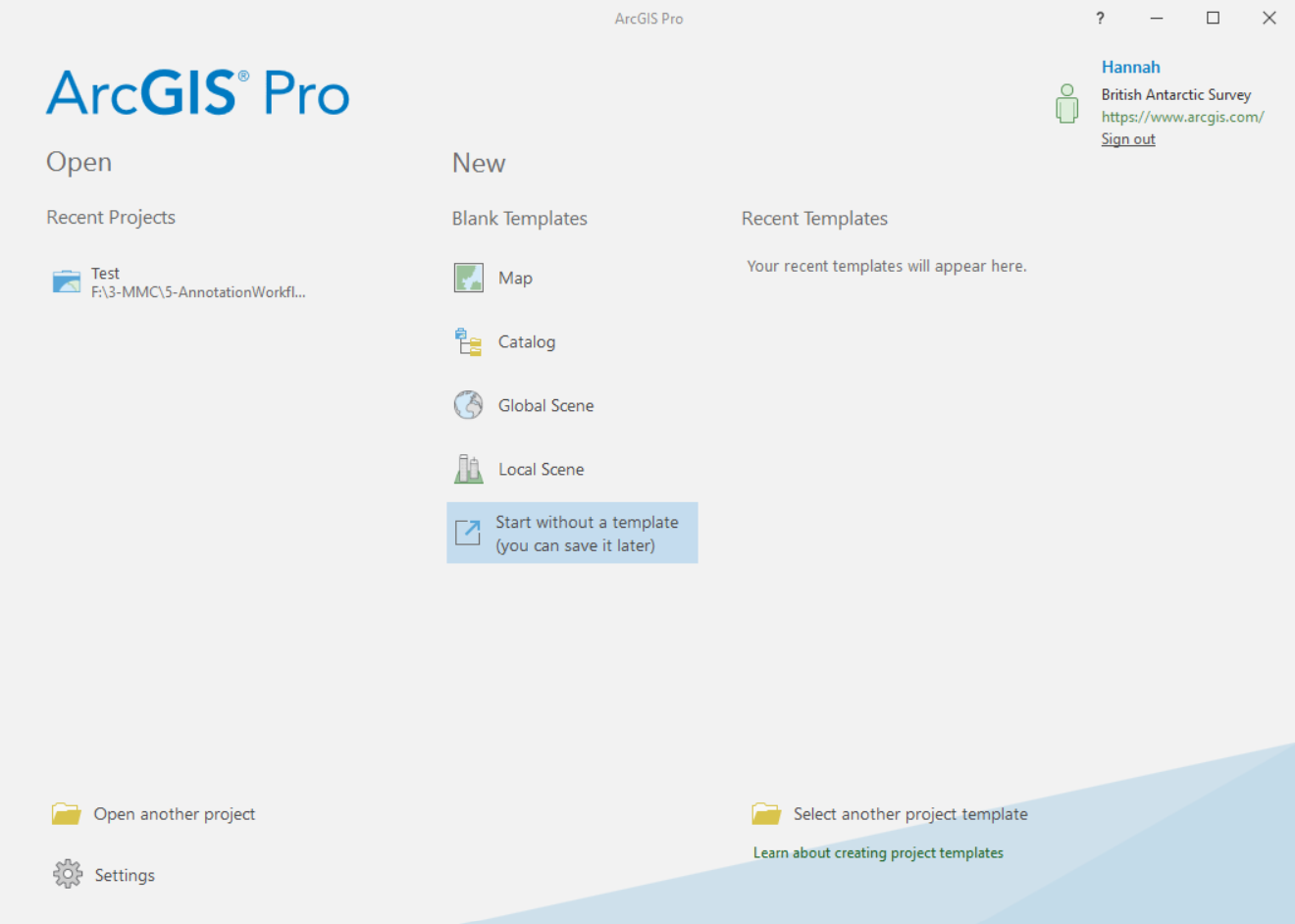

4. If returning to continue annotating a satellite image, under “recent projects” select the map you previously saved and already worked on, and pick up where you left off.


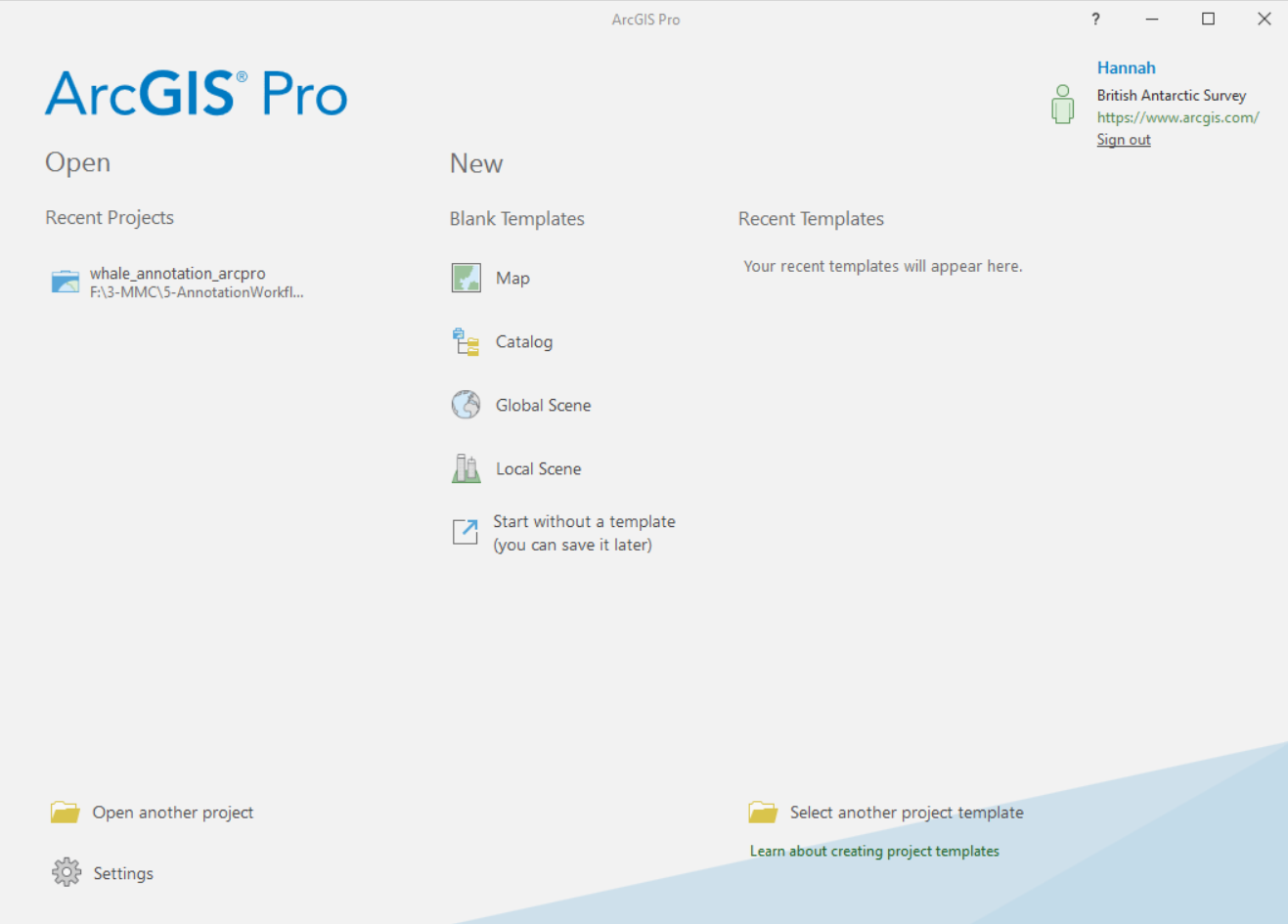


1. Save the map.
   1. In the “Untitled – ArcGIS Pro” window select “Project”.


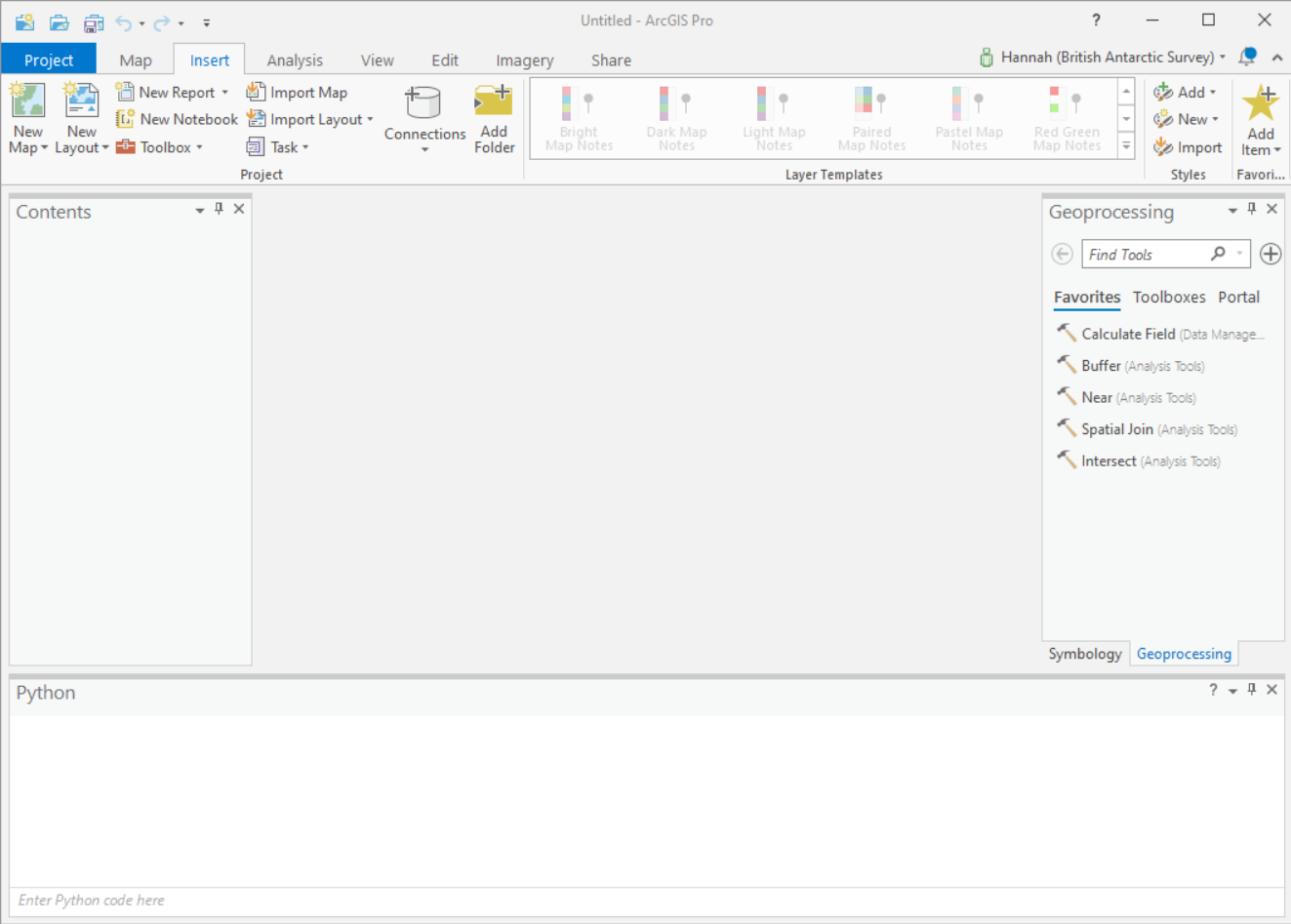


- 1. Then select “Save As”.
  2. A new window opens, choose a file name, there is only one file type available “Projects (APRX)”, and select “Save”.
  3. If they are open, you may close the “Python”, “Geoprocessing”, “Symbology” windows.


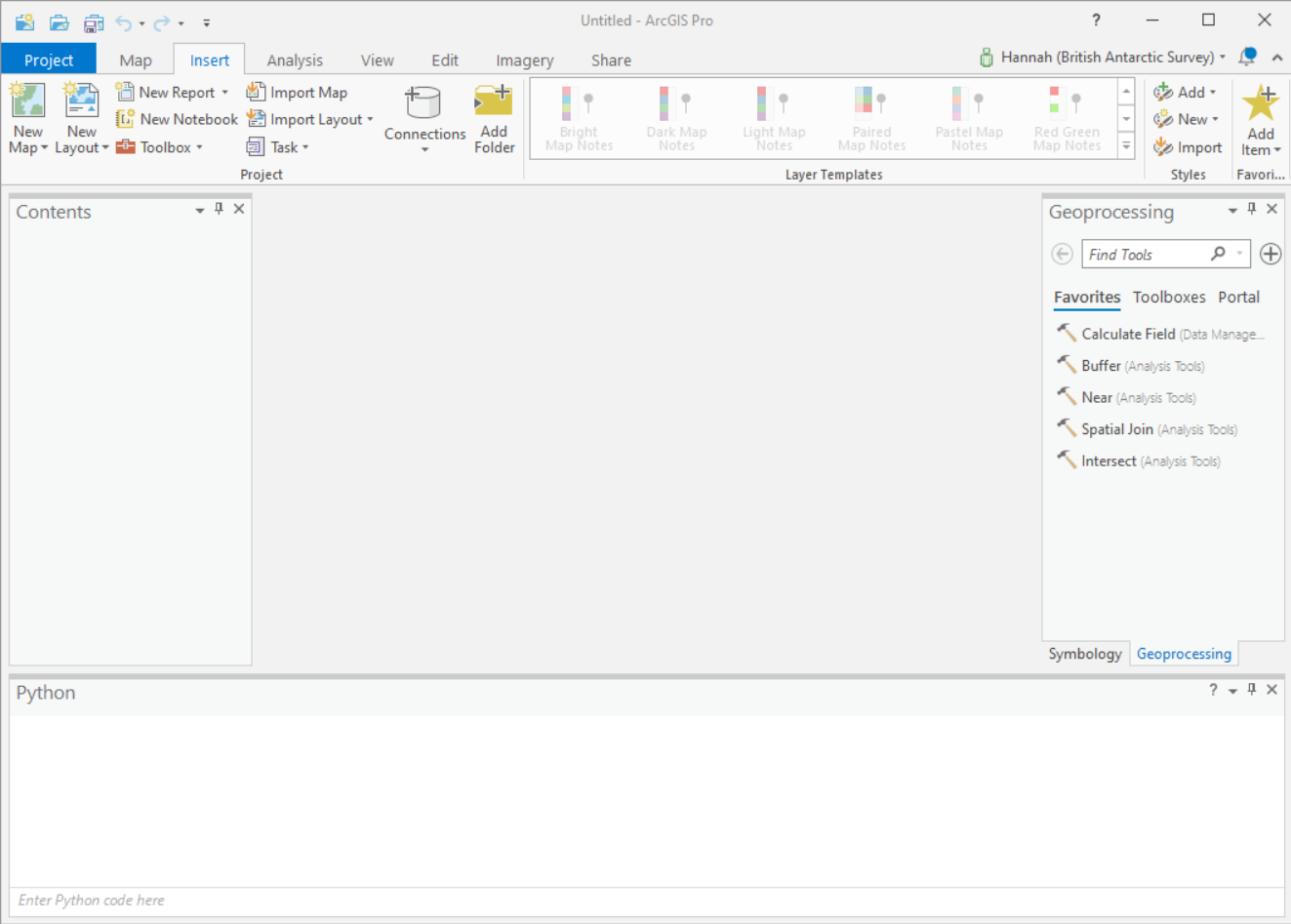


1. Under the “Insert” tab, select “New Map”.


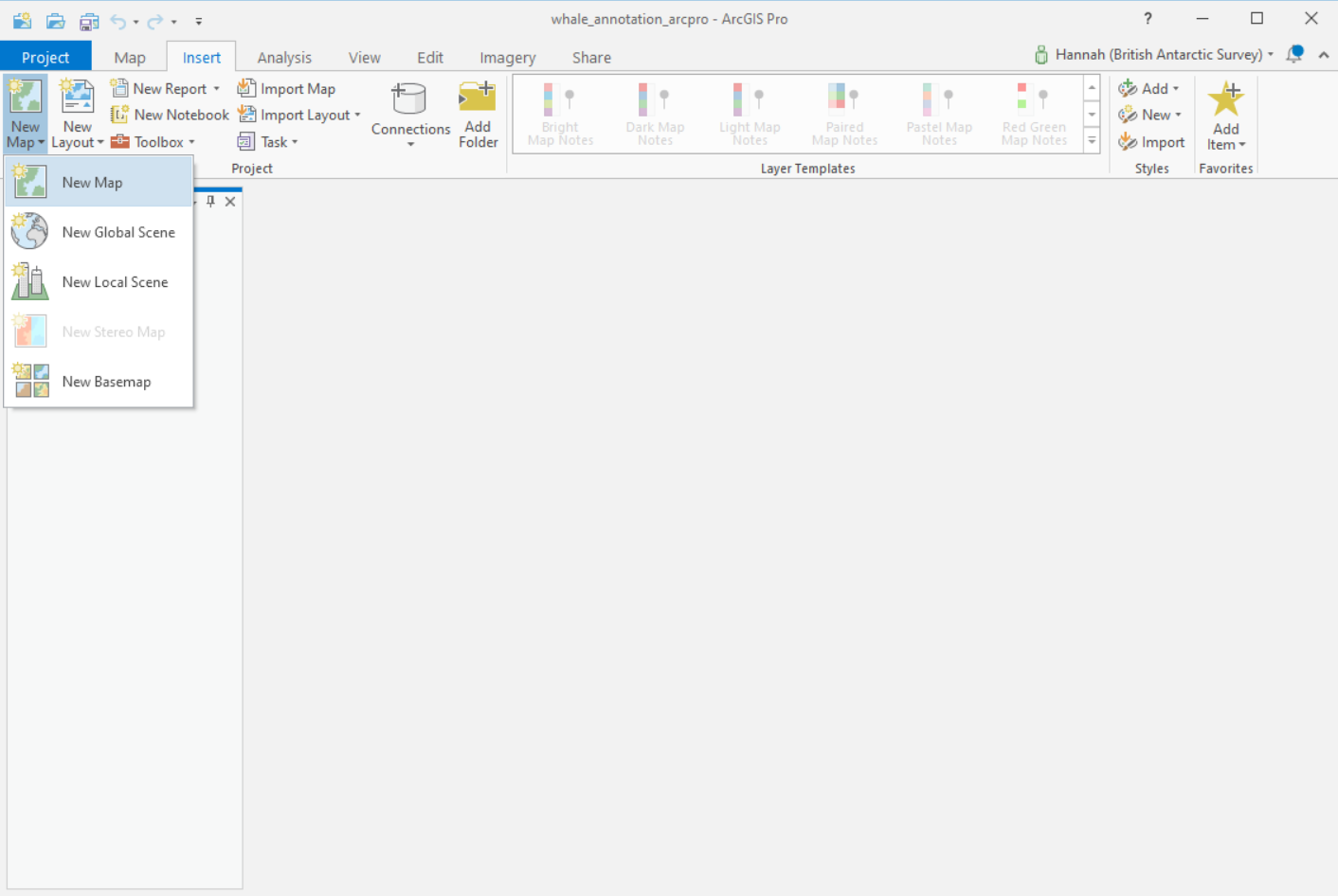


1. Remove the basemap that automatically loads with the new map, by right clicking on the layer and selecting “Remove”. Repeat for all other layers.


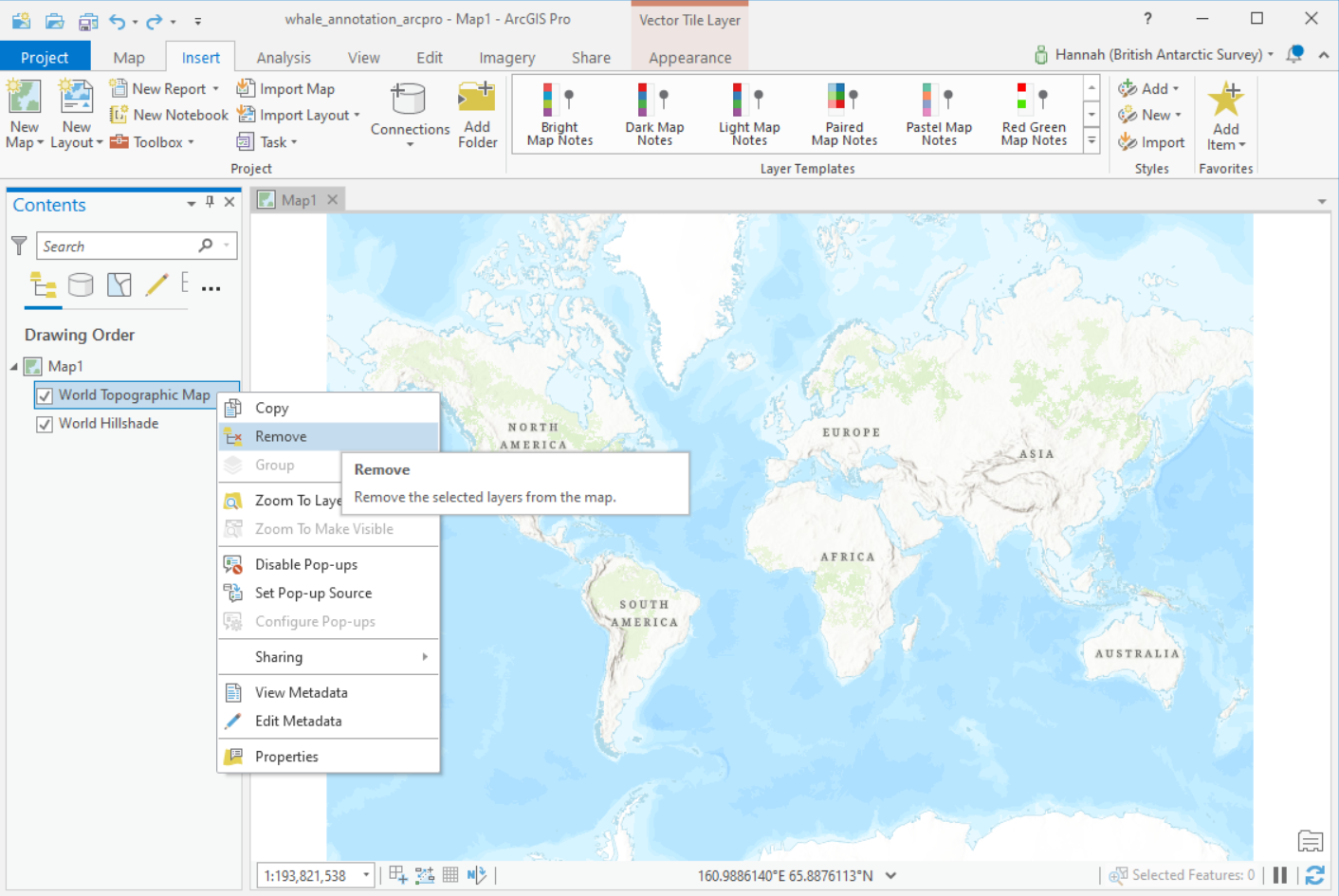


1. Load the multispectral and panchromatic .TIL files, or the pansharpened file if available. First, you need to link the folder where the satellite images are saved.
   1. Under the “Insert” tab, select, “Add Folder”.


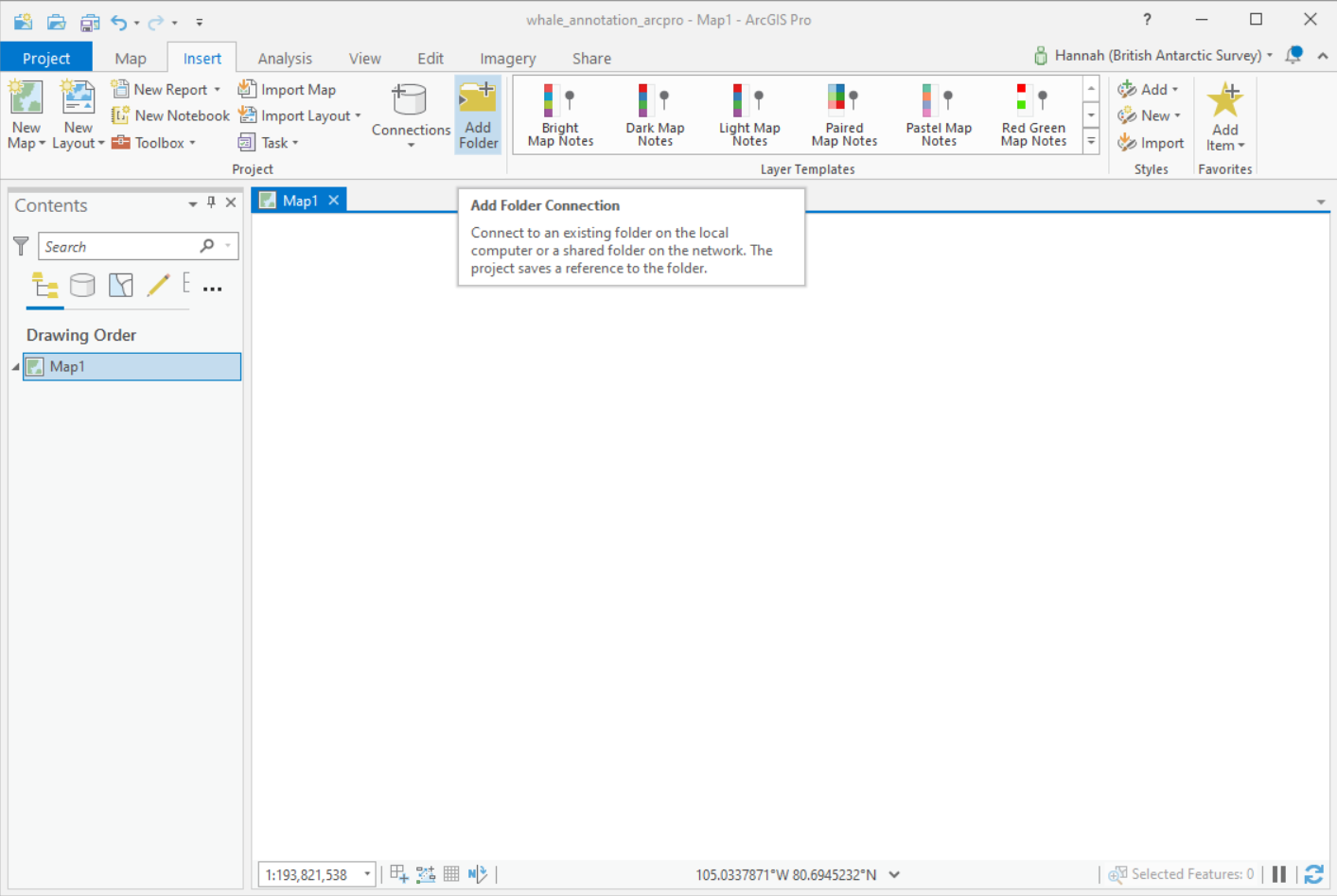


- 1. A new window opens, called “Add Folder Connection”. Navigate to the folder where your satellite images are, select it and click “OK”.


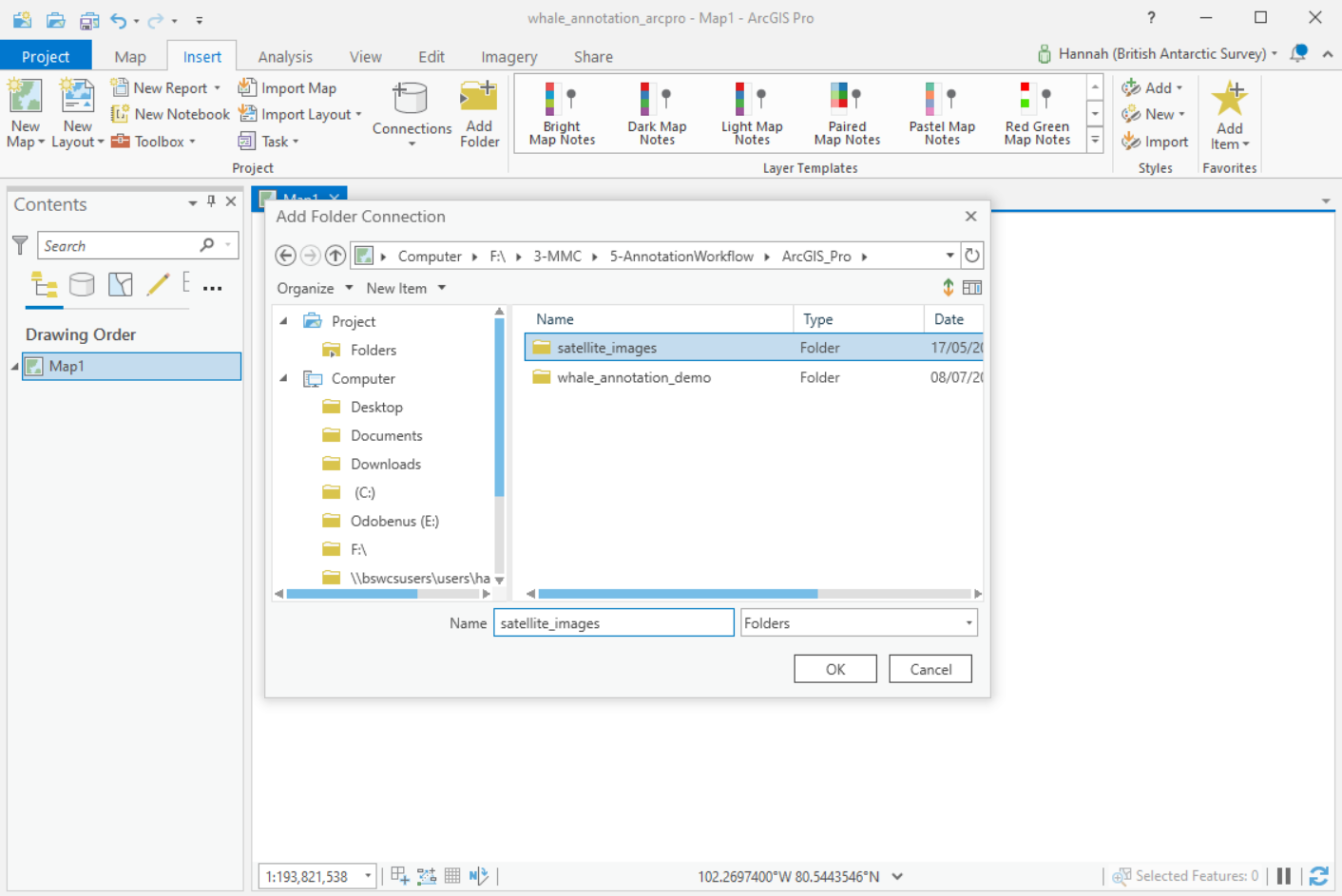


- 1. You can now add the satellite images.
     1. Under the “View” tab, select “Catalog Pane”.


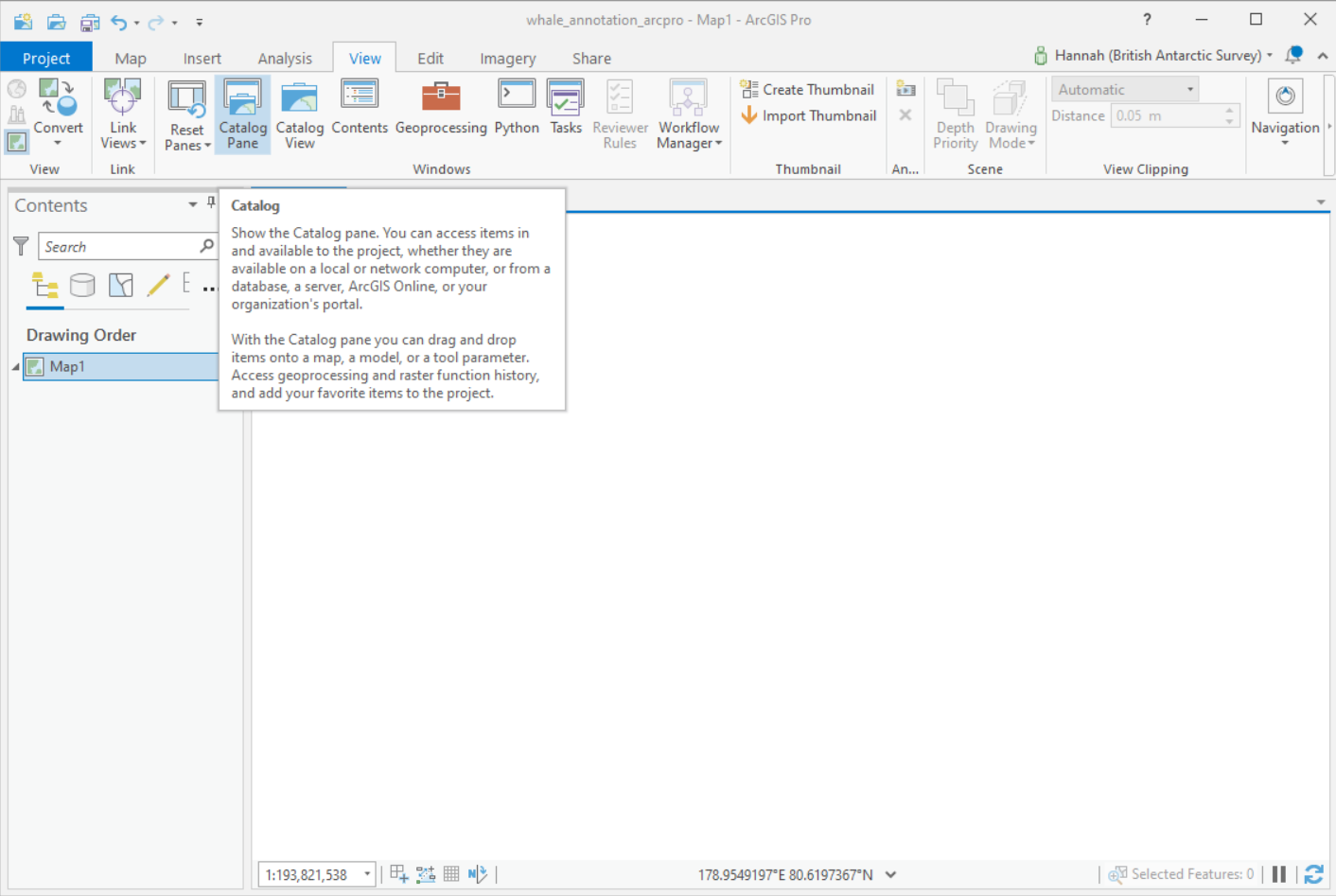


- - 1. A new window will open, called “Catalog”.


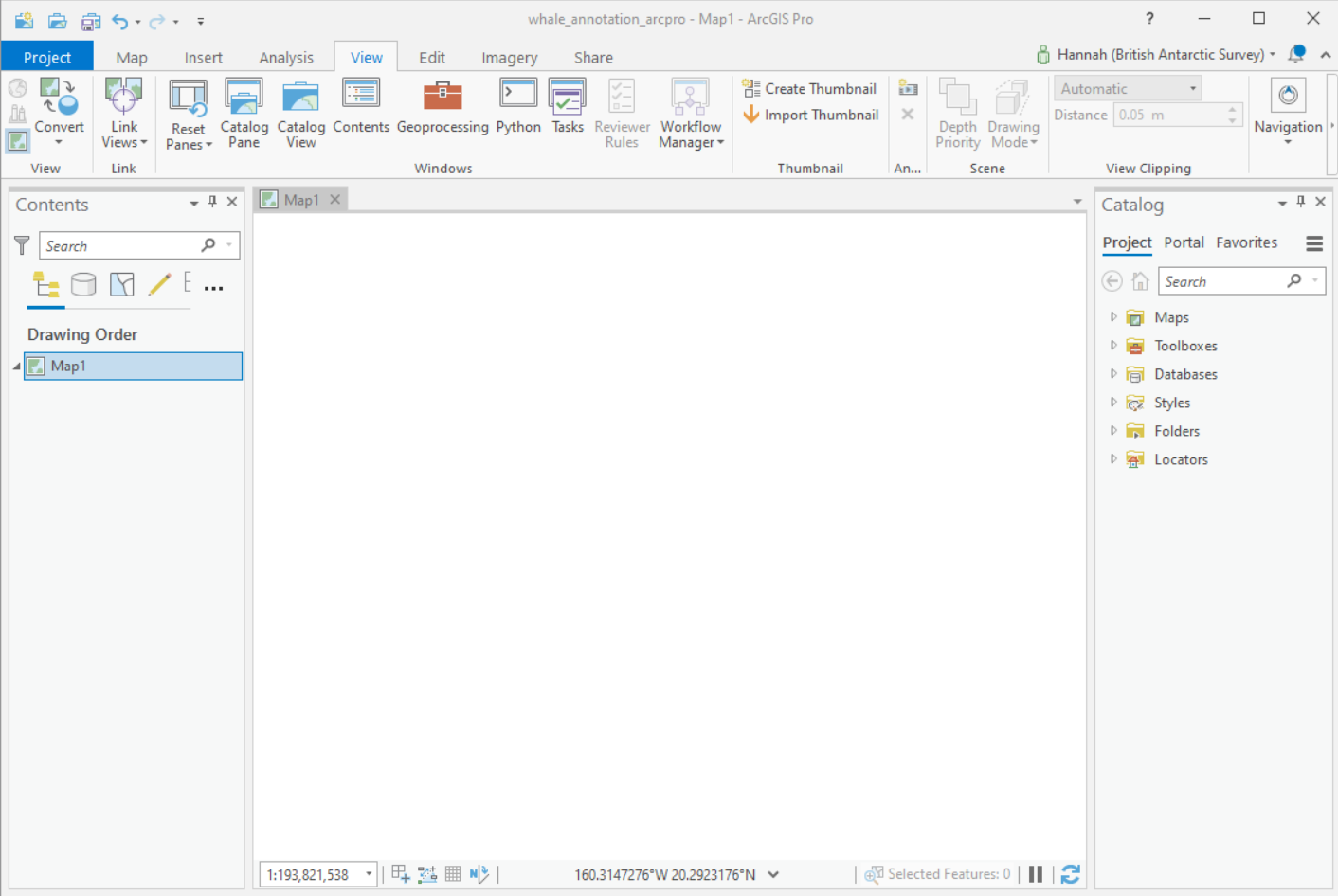


- - 1. If adding the multispectral and panchromatic files, you will need to add them separately (you can start with either). Under the window “Catalog”, select “Folders”, navigate to the folder connected at steps 8.1-8.2, and select and drag the multispectral file to the “Content” window. You will now be able to visualize the image. Select the multispectral .TIL file (the .TIL file is a mosaic of all the .TIF files, which is easier to work with when using a large satellite images made of several .TIF files) and select “Add”. If you are asked to compute the pyramids, select “OK”.


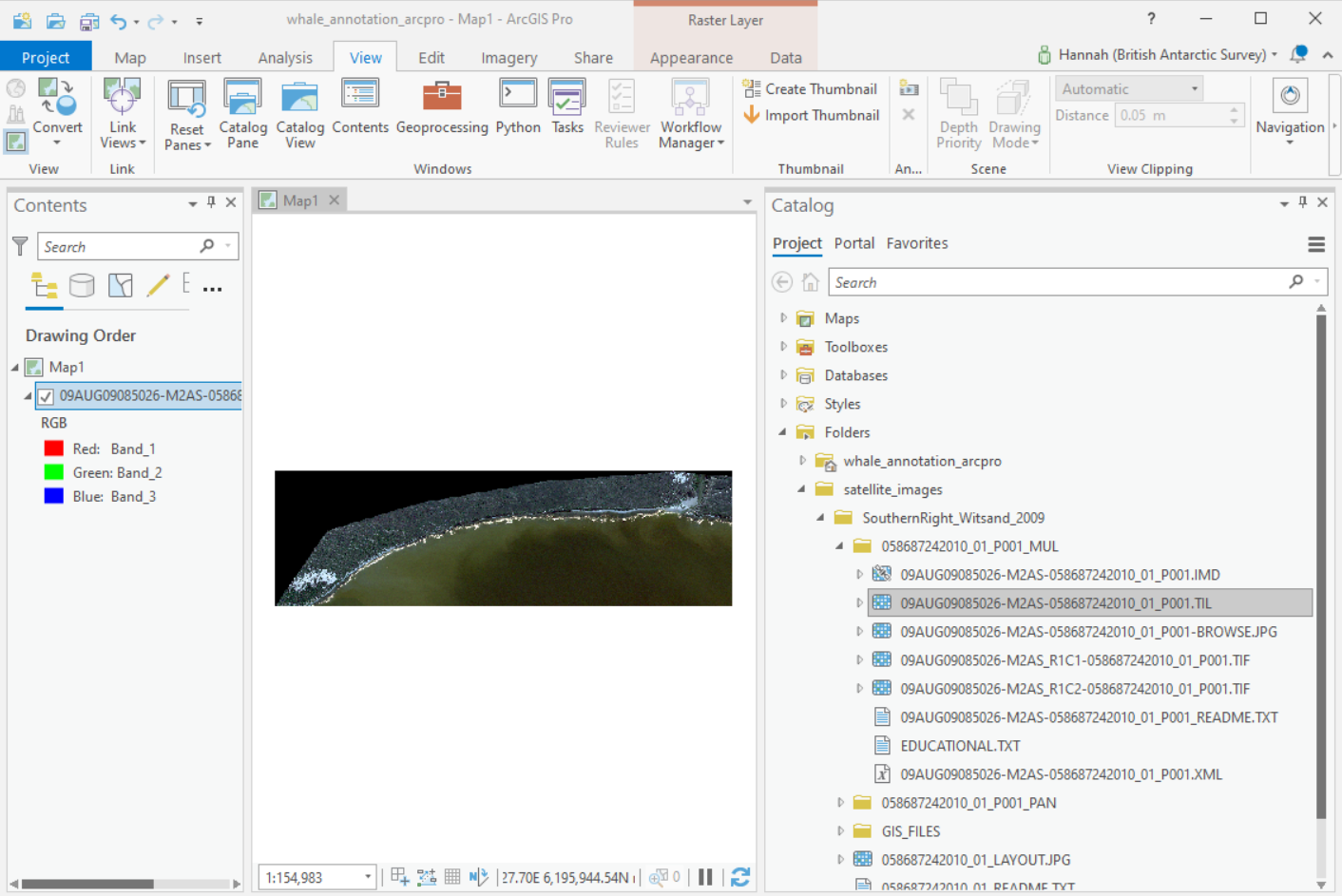


- - 1. Repeat the step above (8.3.3.) for the panchromatic file. Your ArcGIS Pro window should look similar to the image below. Here we used a satellite image of Witsand, South Africa, where there are several southern right whales.


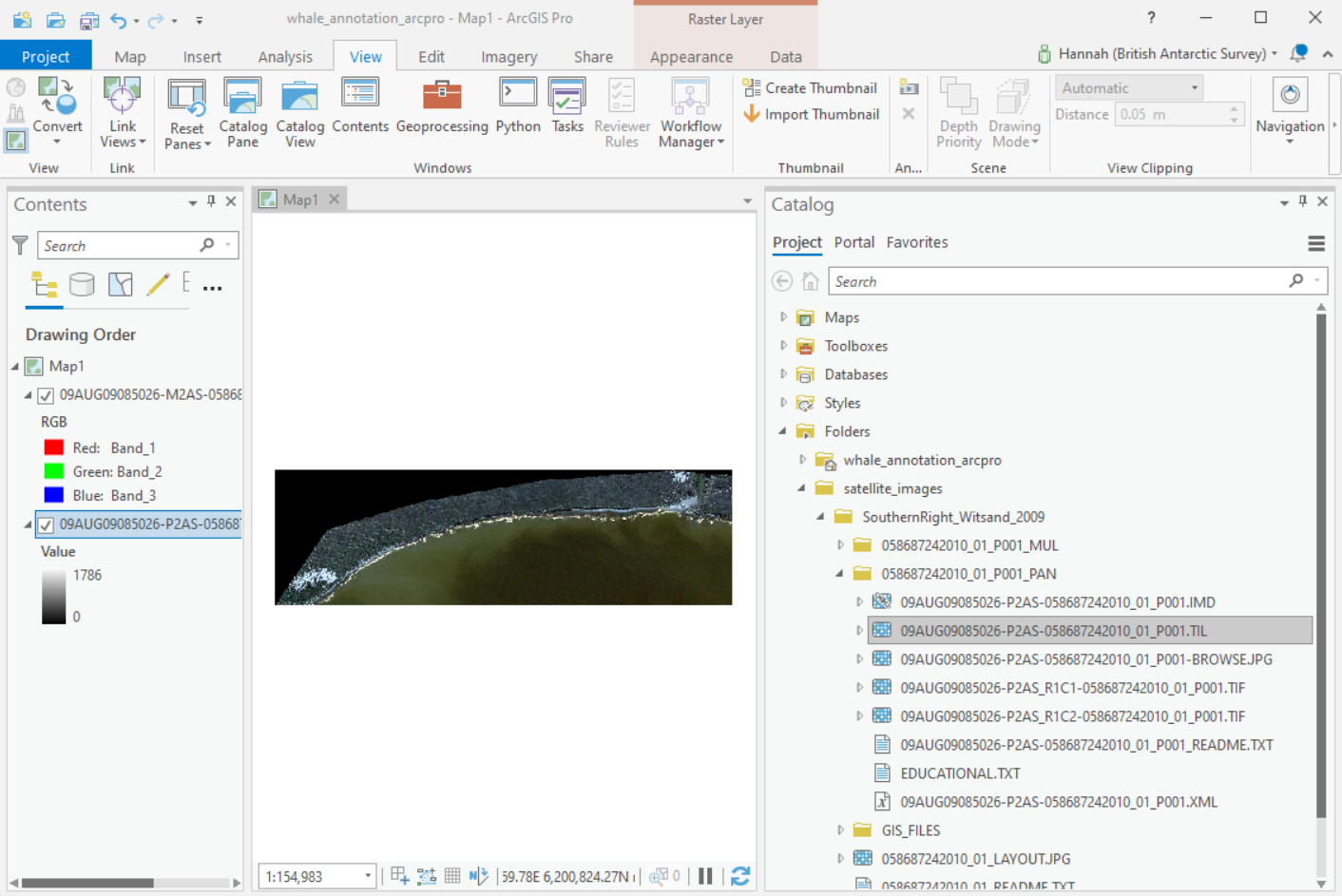


## Pre-processing

### Projection

Depending on the imagery provider and product type (Table1), you may need to project the image to WGS 1984 with the relevant UTM zone. To find the relevant UTM Zone to use, refer to this link (<https://www.dmap.co.uk/utmworld.htm>).

The Witsand image used as an example here is already projected to WGS 1984 UTM Zone 34S. If the image you wish to annotate is not projected to WGS 1984 with the relevant UTM Zone, use the tool “Project Raster (Data Management Tools)” to project your image, and refer to this link for additional guidance for versions 2.8 and above (<https://pro.arcgis.com/en/pro-app/2.8/tool-reference/data-management/project-raster.htm>), and this link for earlier versions (<https://pro.arcgis.com/en/pro-app/latest/get-started/archived-arcgis-pro-help.htm>).

**Table 1.** List of product type for the main VHR satellite imagery provider, Airbus (Airbus, 2022), Planet (Planet, 2022) and Maxar Technologies (Maxar Technologies, 2022).

|  | **Product name** | **Mapping projection** |
| --- | --- | --- |
| **Maxar** | System-Ready (Basic) 1B  System-Ready Stereo (Basic) 1B | Un-projected |
|  | View-Ready (Standard) OR2A  View-Ready Stereo (Standard) OR2A | Projected |
|  | View-Ready (Standard) 2A | Projected |
|  | Map-Ready (Ortho) 1:12,000 | Projected |
| **Airbus** | Primary | Coordinate Reference System: WGS84  Map projection: None |
|  | Projected | Coordinate Reference System: WGS84  Map projection: UTM |
|  | Ortho | Coordinate Reference System: WGS84  Map projection: None |
| **Planet** | SkySat Basic Scene | Coordinate Reference System: WGS84  Map projection: None |
|  | SkySat Ortho Scene | Coordinate Reference System: WGS84  Map projection: UTM |
|  | SkySat Ortho Collect | Coordinate Reference System: WGS84  Map projection: UTM |

### Top of atmosphere correction

If the aim of the project is to extract spectral data from the imagery, it is recommended to correct for the top of atmosphere. This can be done in ENVI similar to Cubaynes *et al.*, 2019, or other software.

### Pansharpening

1. If the image is not already pansharpened, (you are working with a multispectral and a panchromatic files), pansharpen using the following steps. If your image is already pansharpened go to step 9.6.
   1. Under the “View” tab, select “Geoprocessing”.


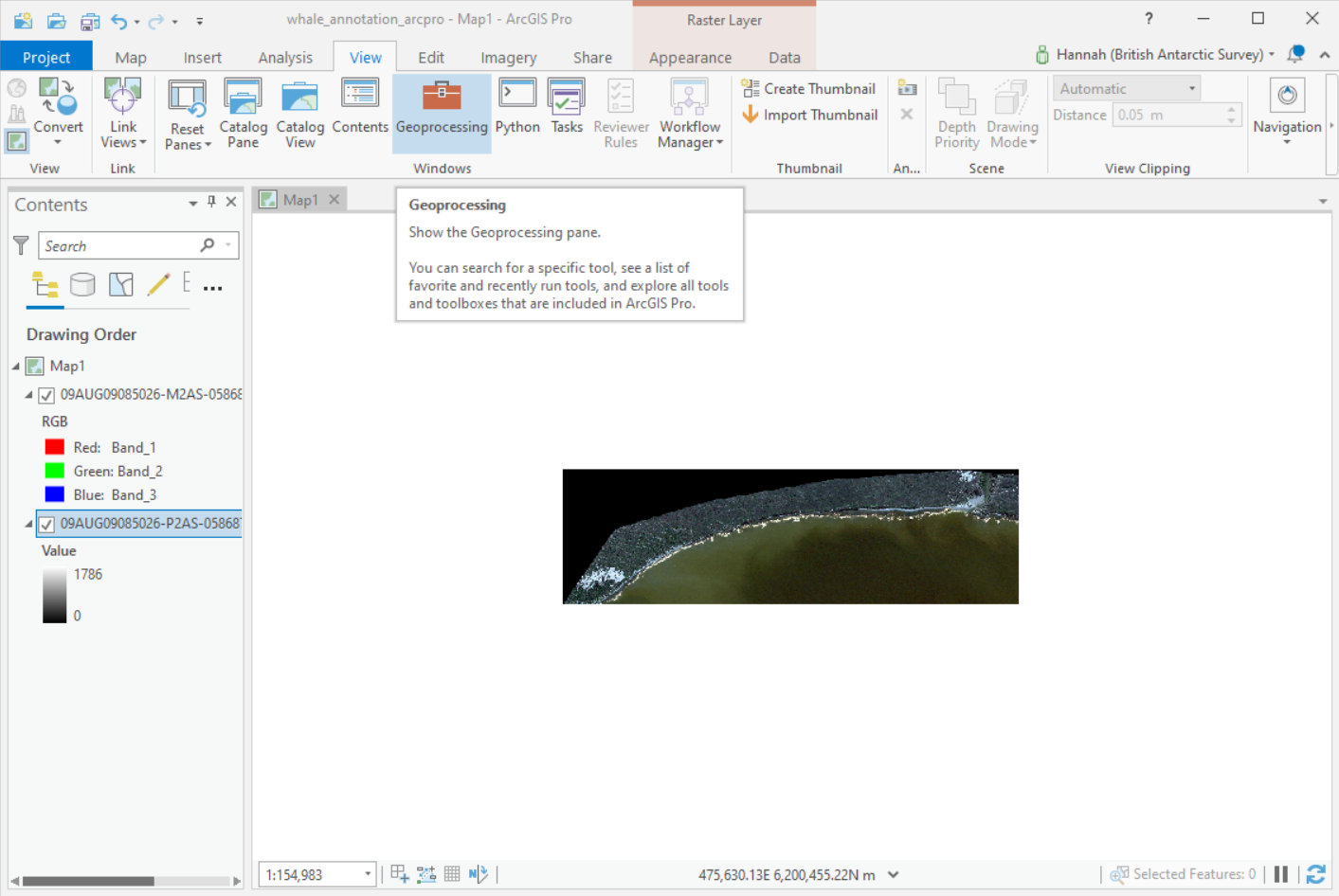


- 1. Under the new window that opened called “Geoprocessing”, search for “pansharpen”, and select “Create Pansharpened Raster Dataset”.


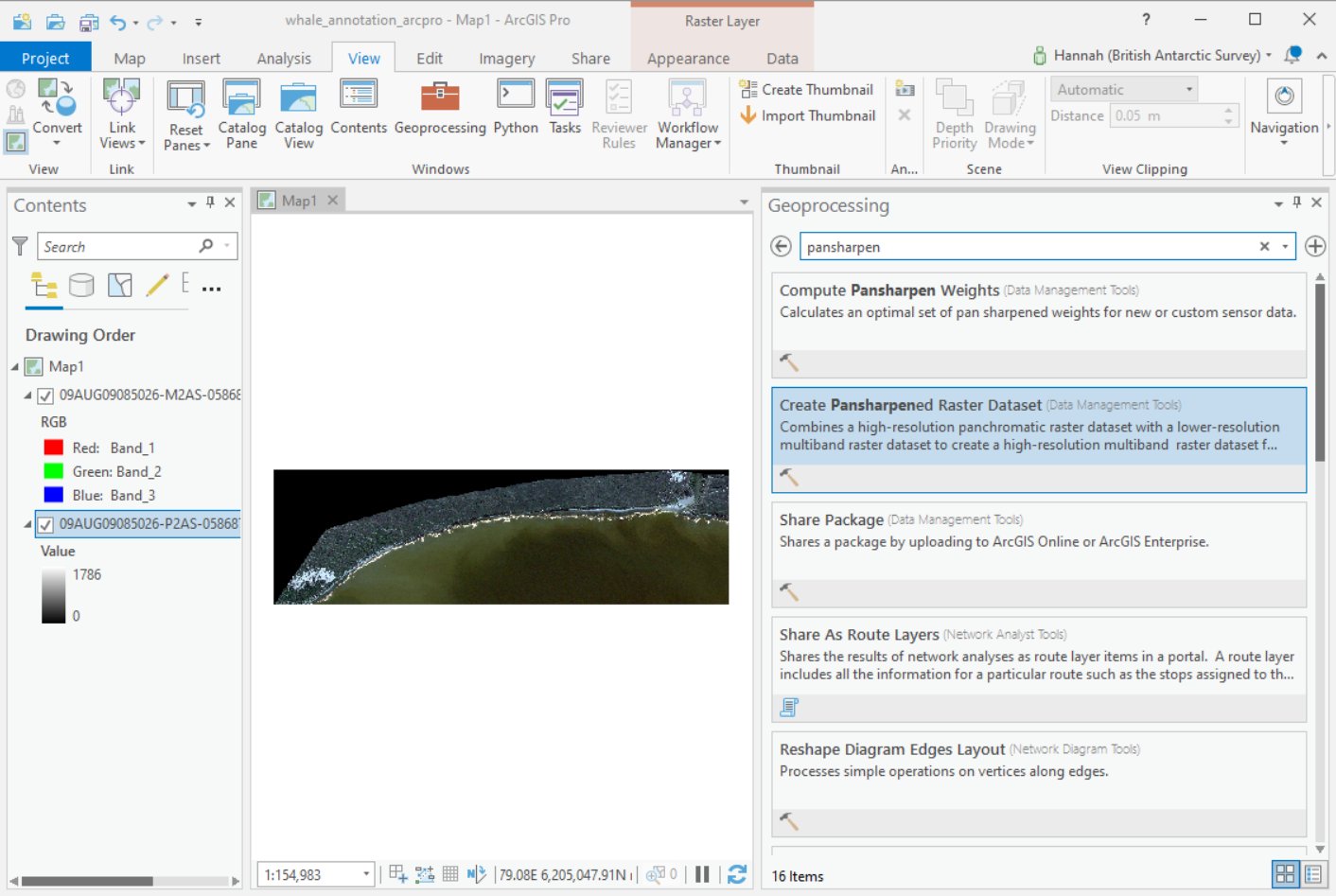


- 1. Fill in the following information, and select “Run”, this step may take time:
- Input Raster: from the drop down menu, select the multispectral image.
- Output Raster Dataset: Choose the location, and name for the pansharpened file that will be created.
- Panchromatic Image: from the drop down menu select the panchromatic image.
- Pan-sharpening Type: we would suggest using Brovey.


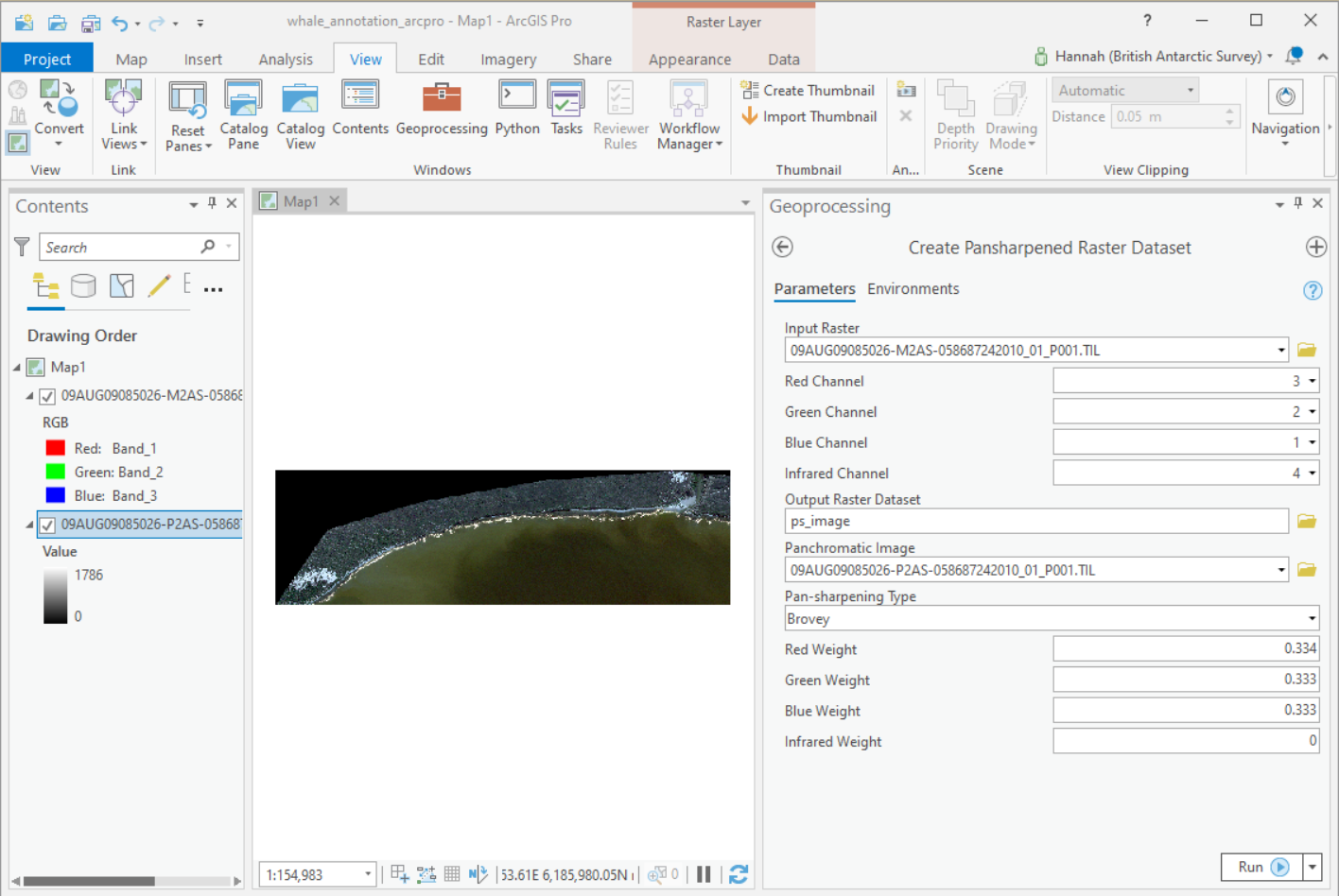


- 1. You will now see a new file under your “Table of Contents”.
  2. Close the “Geoprocessing” window.
  3. Update the symbology settings for the newly created pansharpened file:
     1. Right click on the pansharpened file and select “Symbology”


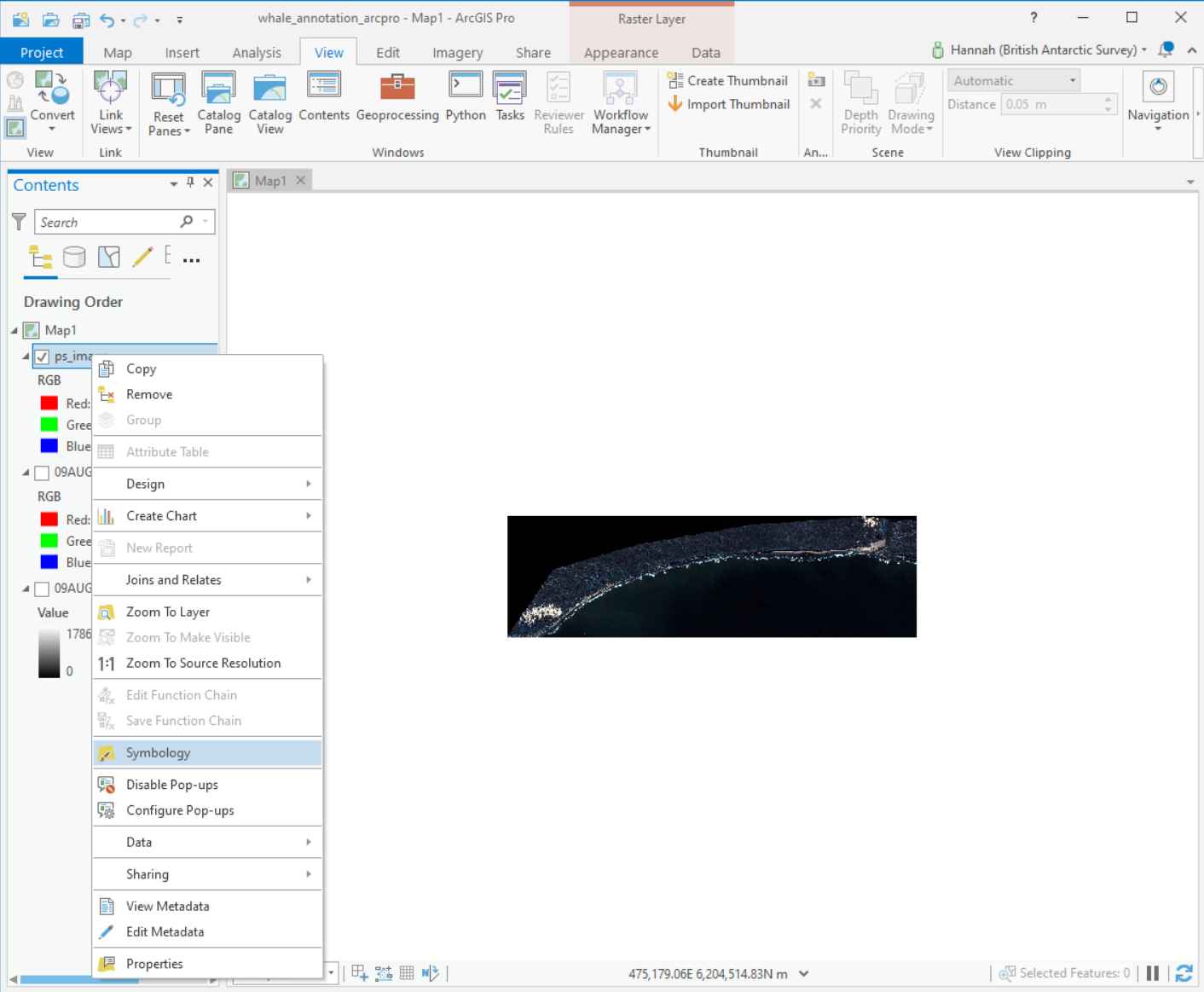


- - 1. A new window will open, called “Symbology”. You will have to change the band for the red, green and blue channel. For WorldView-2 to 4, and GeoEye-1, band 1 is the blue channel, band 2 is the green channel, band 3 is the red channel and band 4 is the near infrared (NIR) channel. For these satellites, change the band for the red, green, blue channels to the following:


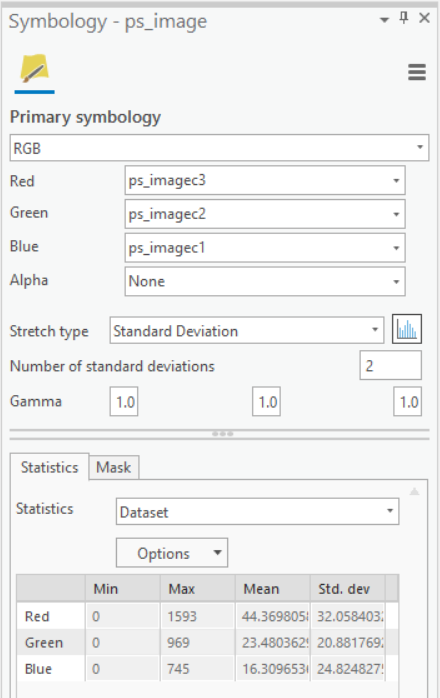


Feel free to play with the combination of bands as another combination might make it easier to review the image.

- - - 1. You can also update the stretch, standard deviation is usually a good one but it will vary between images and likely monitor/screen.


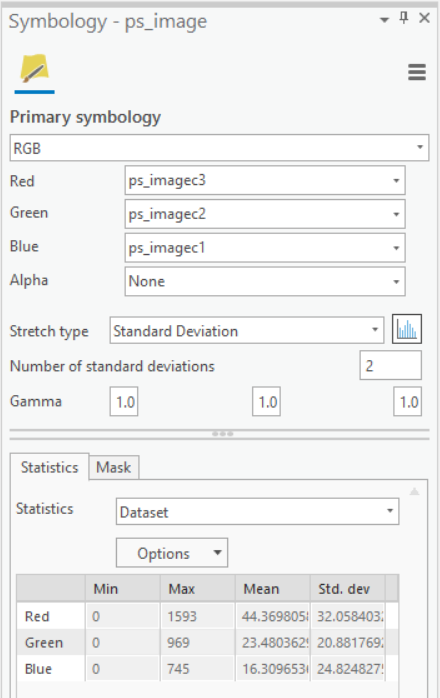


- - 1. Depending on the monitor/screen you are using you might need to update the contrast and brightness, which you can adjust using the “Enhancement” tool, under the “Appearance” tab.


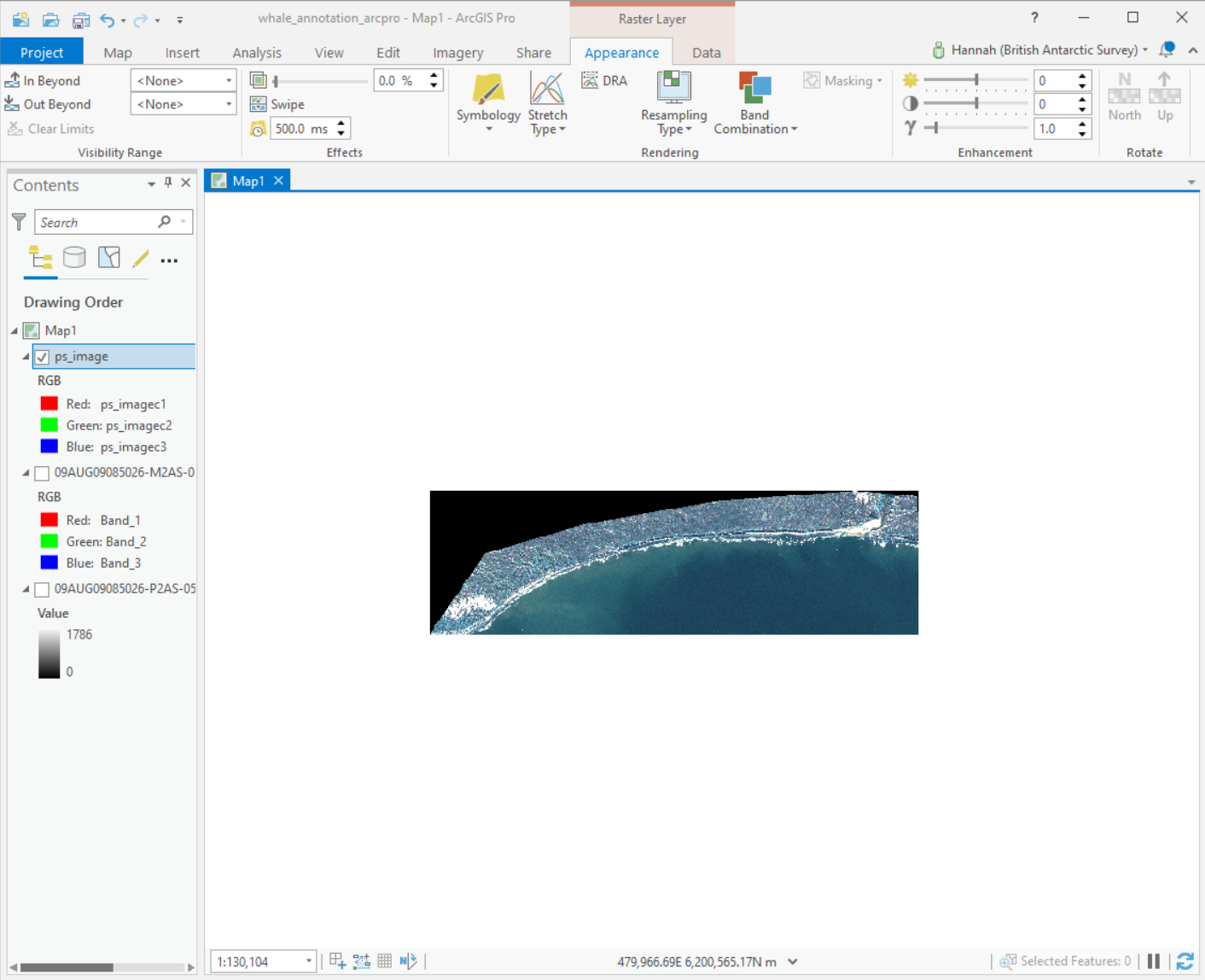


Alternatively, you can change the brightness and contrast, by selecting the “Image” tab, then “Raster Functions”, then “Appearance”.

## Preparing the Workspace

### Creating a grid

1. Create a grid that will help review the image. Our recommendation is to review an image at a scale of 1:1,500 for large whales and zooming in when required (for smaller cetaceans, such as belugas use a scale of 1:1,250).
   1. First, zoom in to 1:1,500 (or 1:1,250 if searching for smaller cetaceans or to your preferred scale).


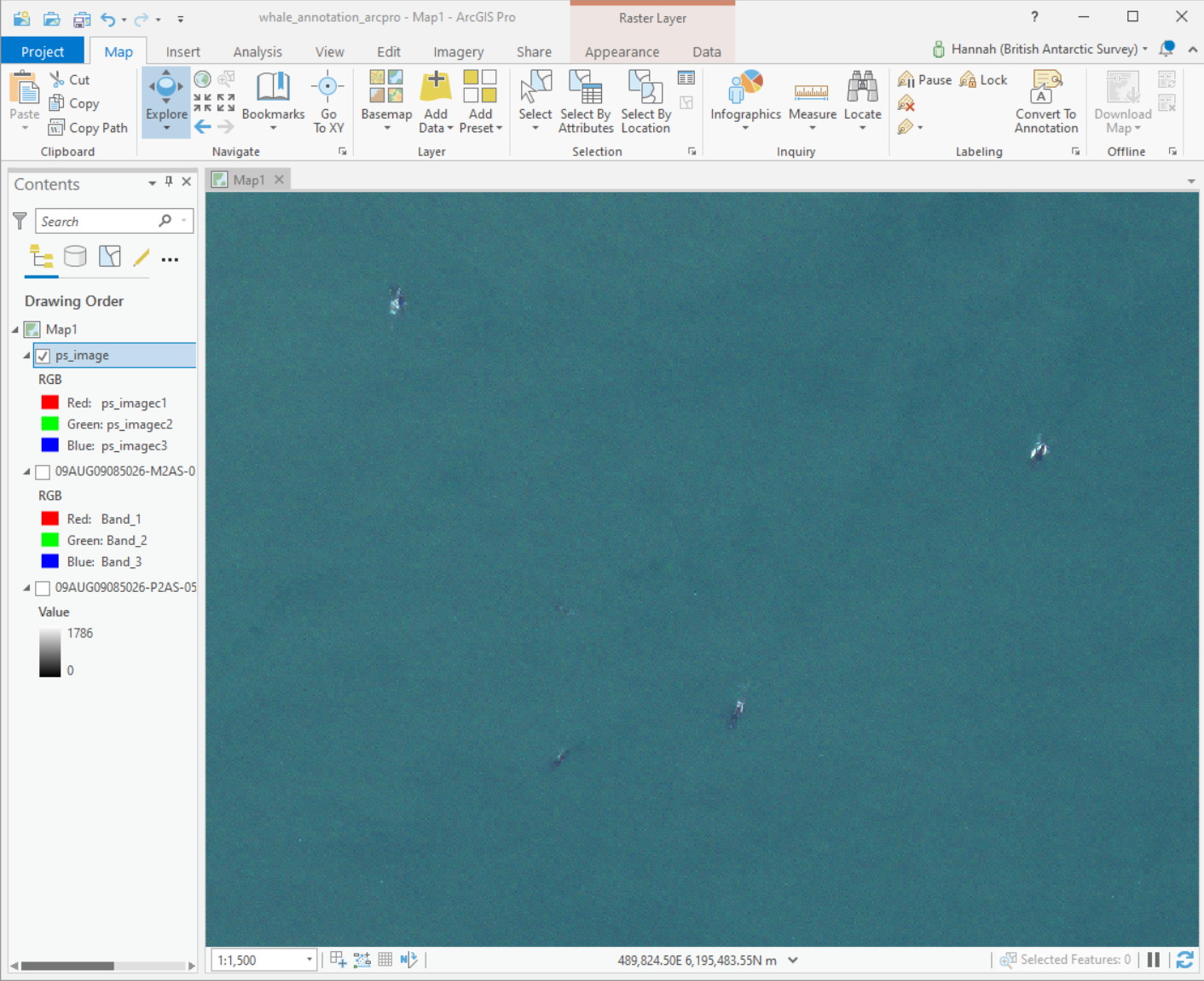


- 1. Under the “Map” tab select, “Measure”, then “Measure Distance”. It will open the “Measure Distance” window.


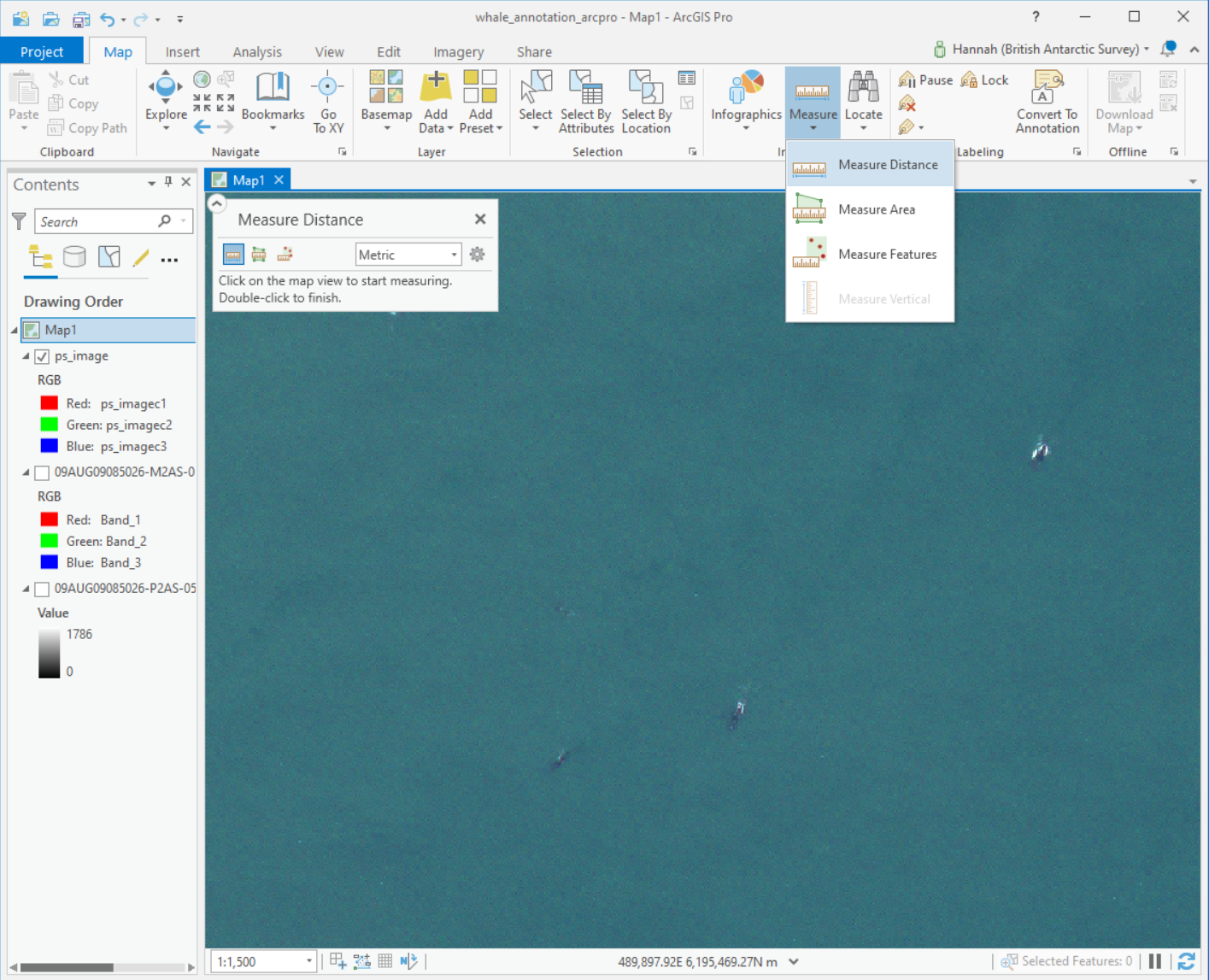


- 1. Ensure you are measuring in meters


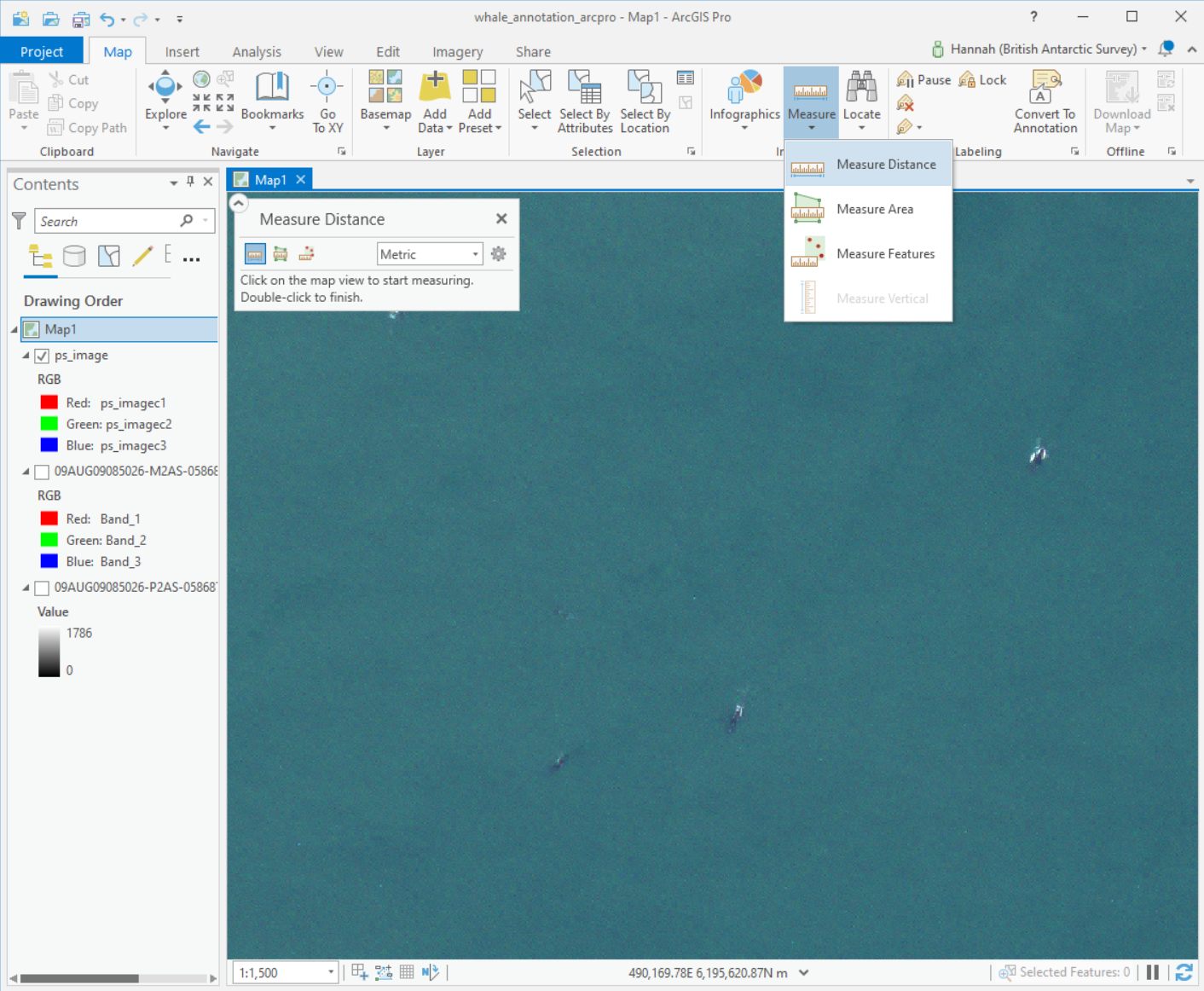


- 1. Measure the height and width of your screen in meters, write this information on a piece of paper, as you will need it for the next step.


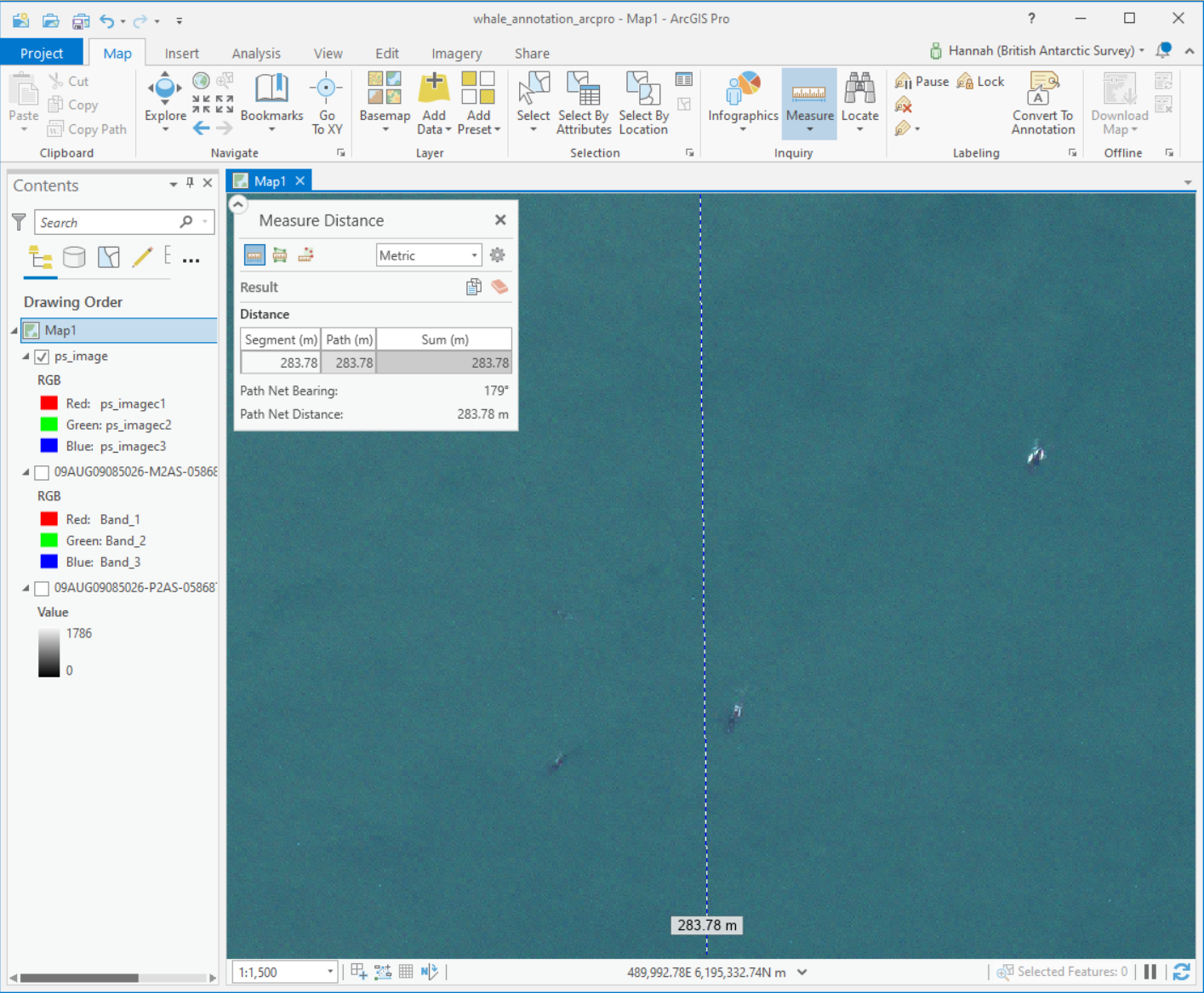


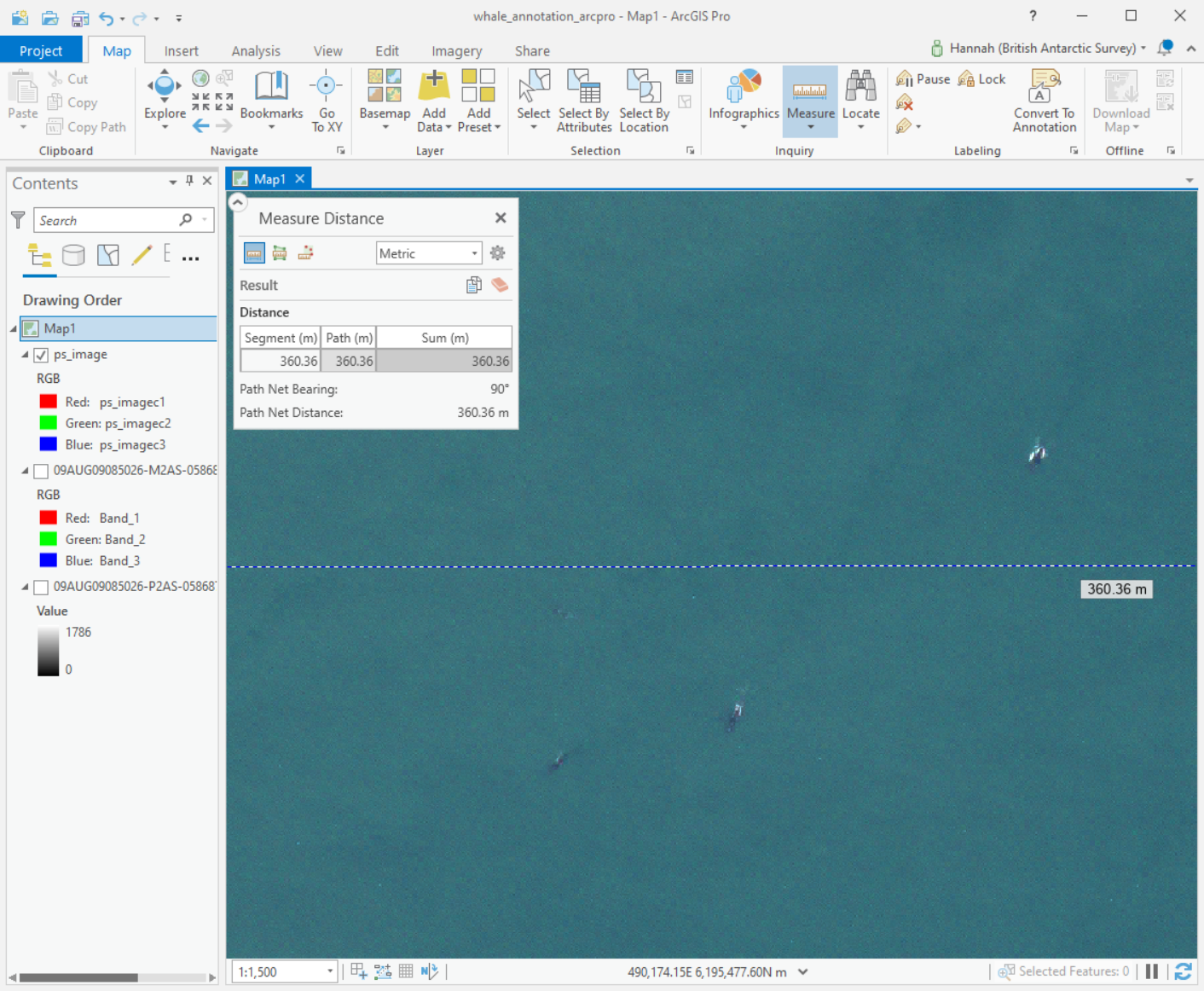


Here we would recommend creating a grid of height 280 m and width 360 m.

- 1. Open the ‘Create Fishnet’ tool, by selecting “Geoprocessing” under the “View” tab, and searching for “fishnet”. Then select “Create Fishnet”.


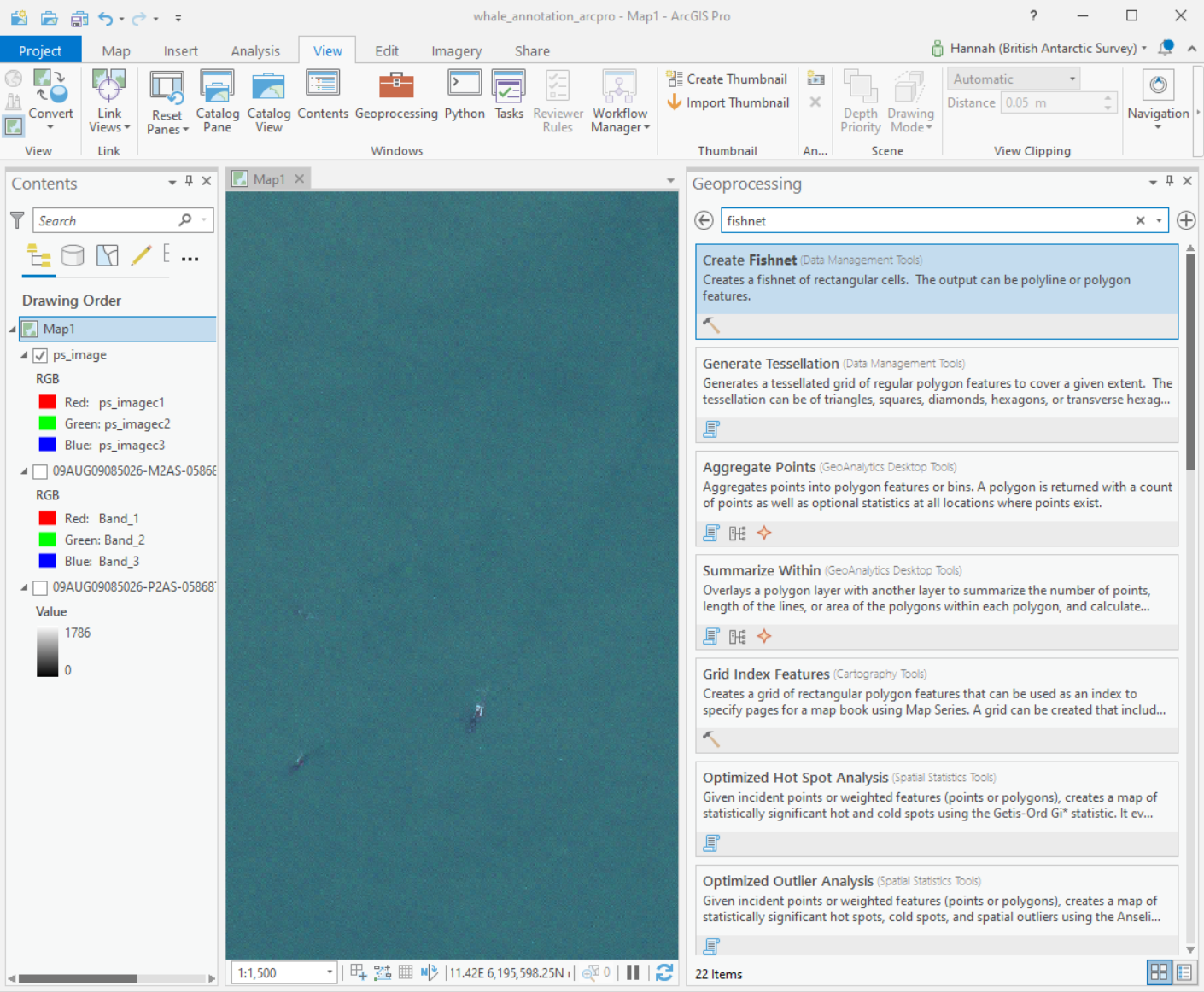


- - 1. The “Create Fishnet” window will open and fill in the following information:
- Output Feature Class: The name you wish to give to your grid, *e.g.* ‘grid’
- Template Extent (optional): select the pansharpened file
- Cell Size Width: the width you recorded at step 10.4.
- Cell Size Height: the height you recorded at step 10.4.
- Untick ‘Create Label points (optional)’
- Geometry Type: ‘Polygon’


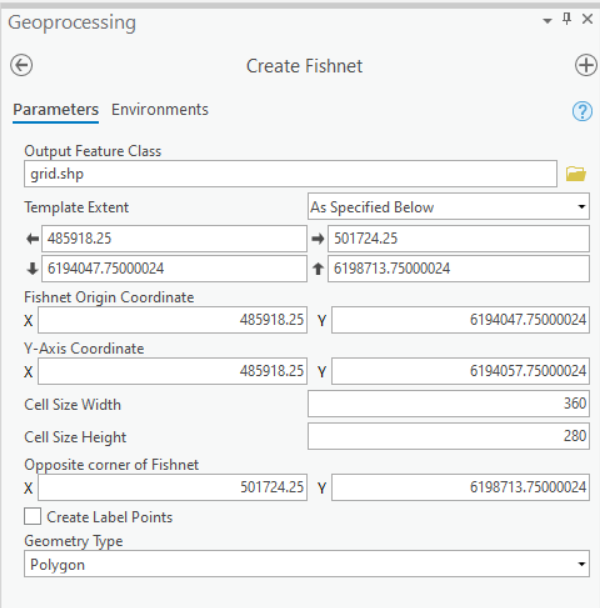


- - 1. After you select “Run”, the grid shapefile will then appear under the window “Contents”.


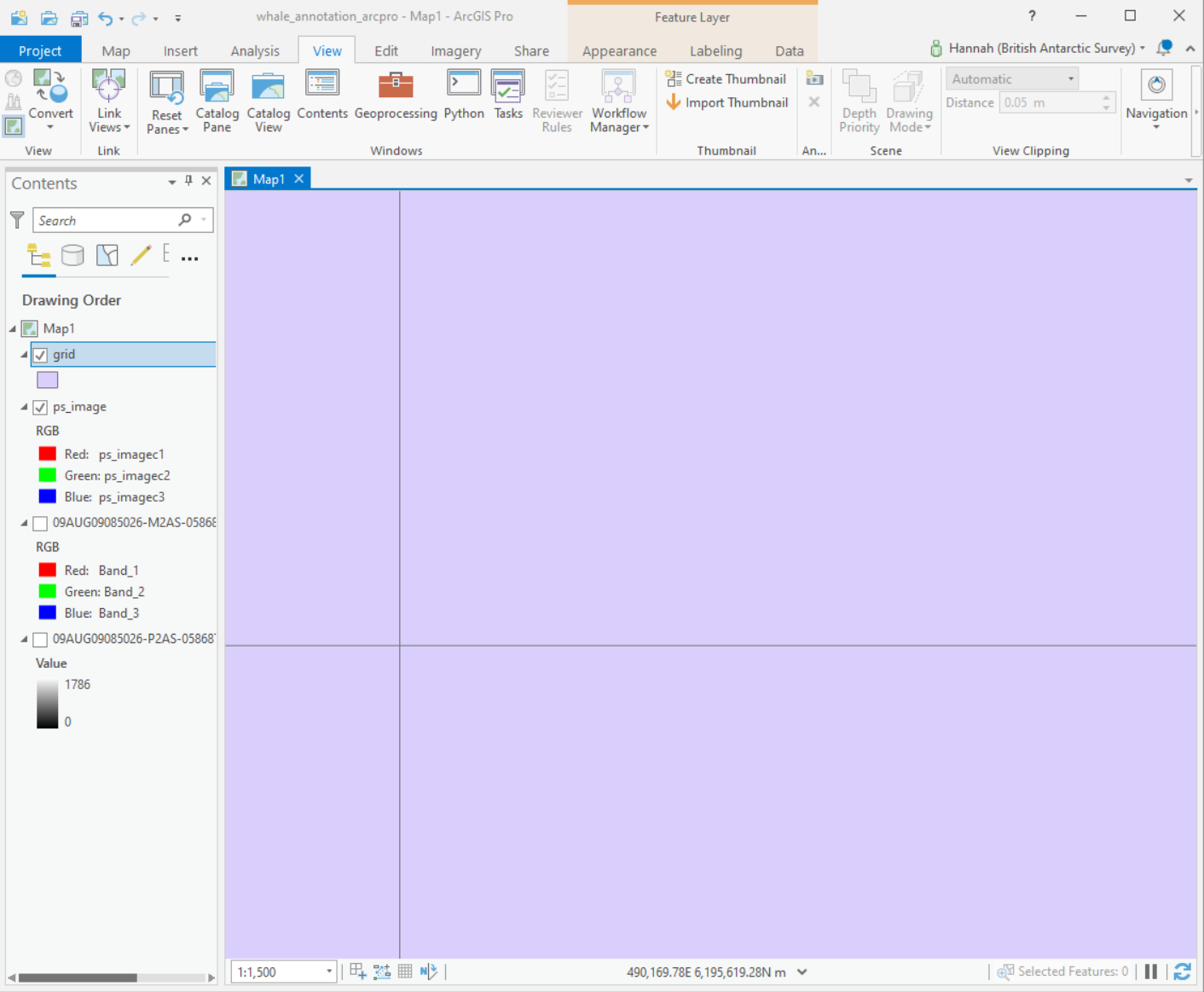


- - 1. Update the symbology to a no fill, thicken the outline width and change the outline color to a color that will contrast with your image. Double click on the rectangle below the grid shapefile. Then under the “Symbology” window, select the “Properties” tab.


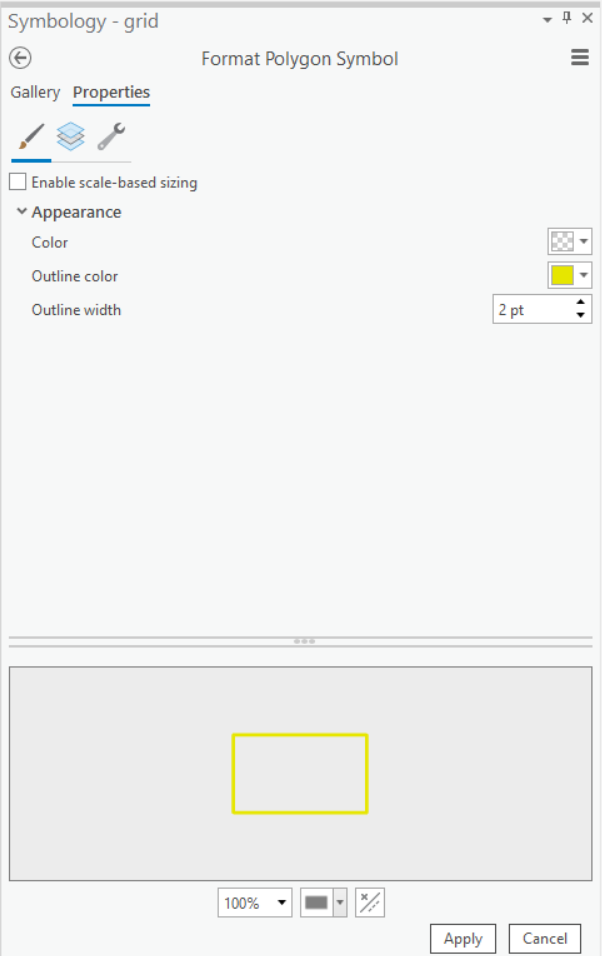


Your workspace should look similar to this.


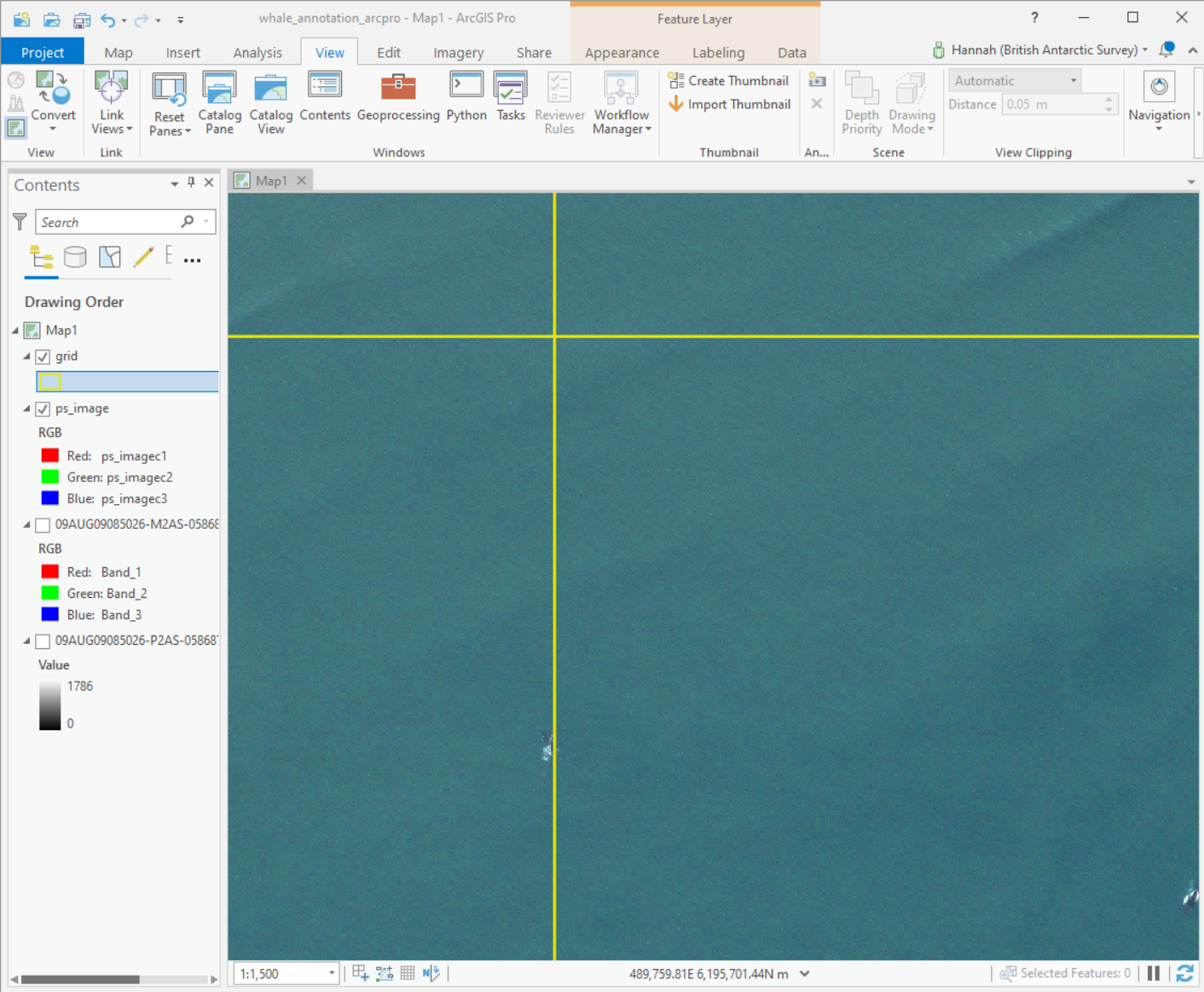


- - 1. To help you keep track of which cell of the grid have been reviewed, we recommend creating a new Field in the attribute table.
       1. Select your grid shapefile, in the “Contents” window, then under the “Data” tab select “Attribute Table”.


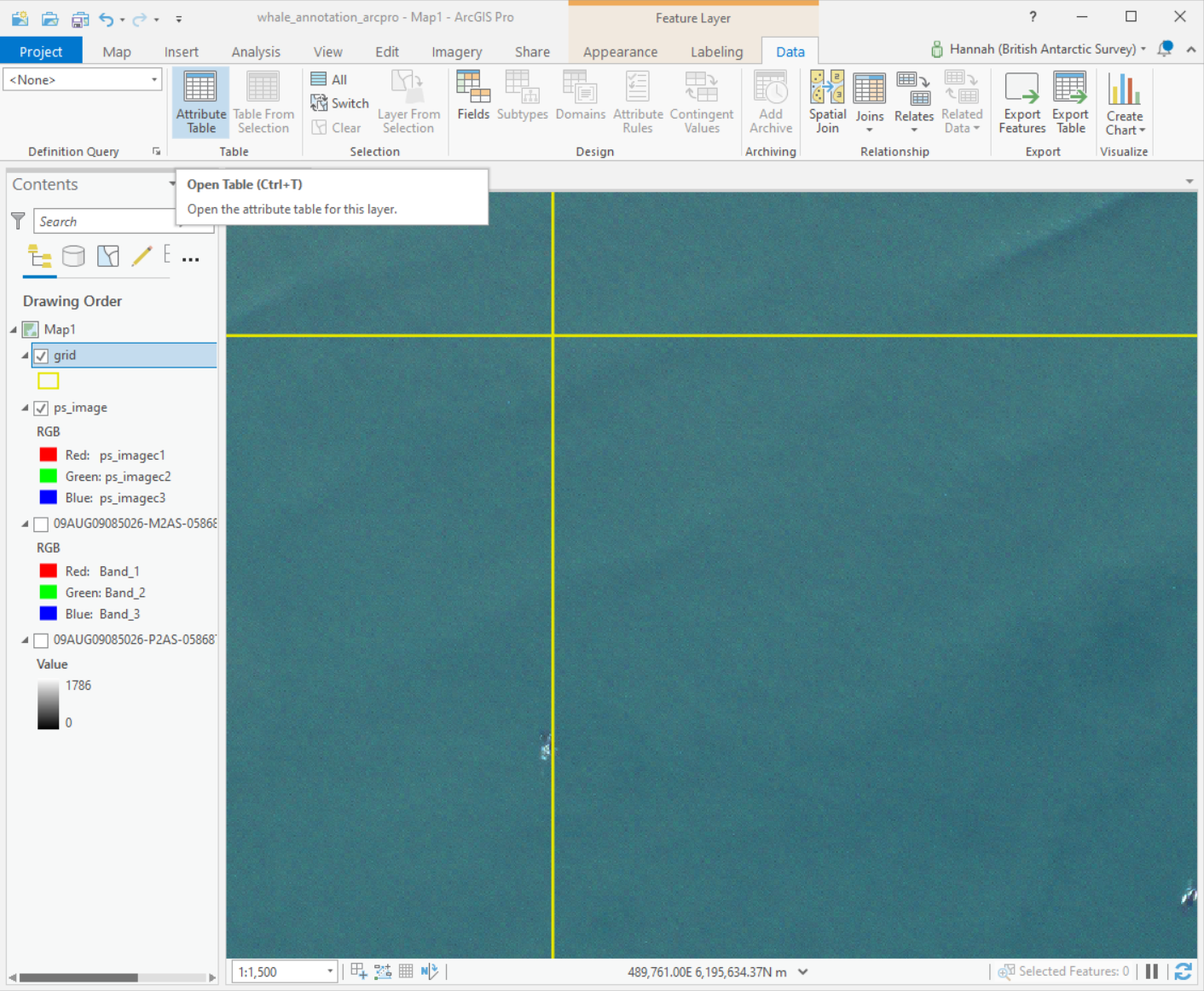


- - - 1. Under the “Table” window, select the “Add Field” icon.


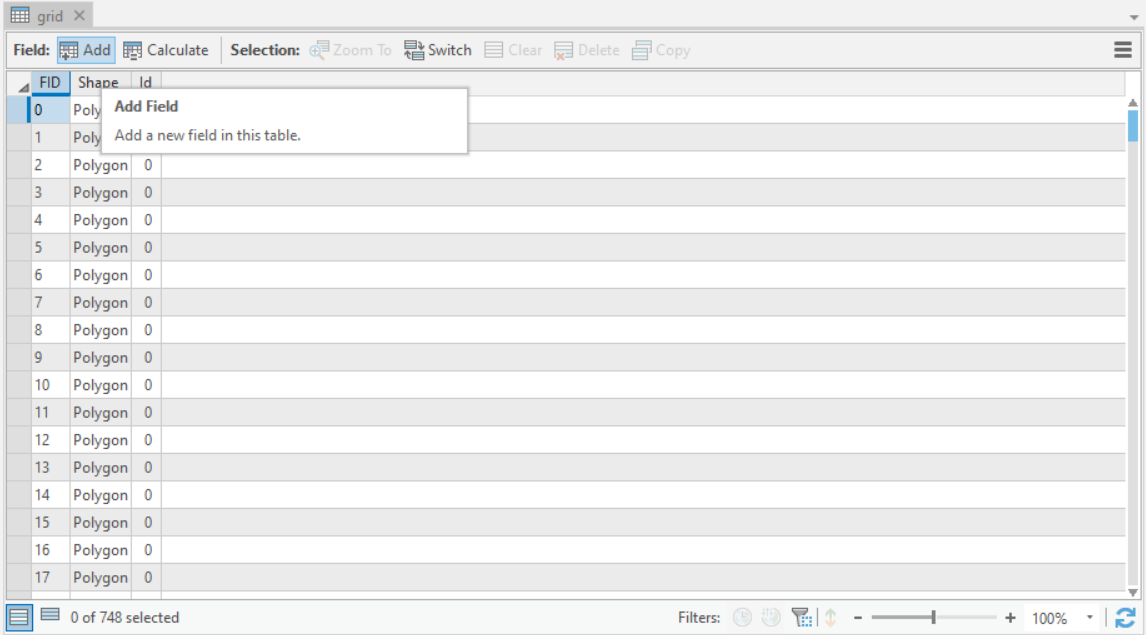


- - - 1. Fill in the “Add Field” window as follow:

Name: Done

Data Type: Text

Length: 5


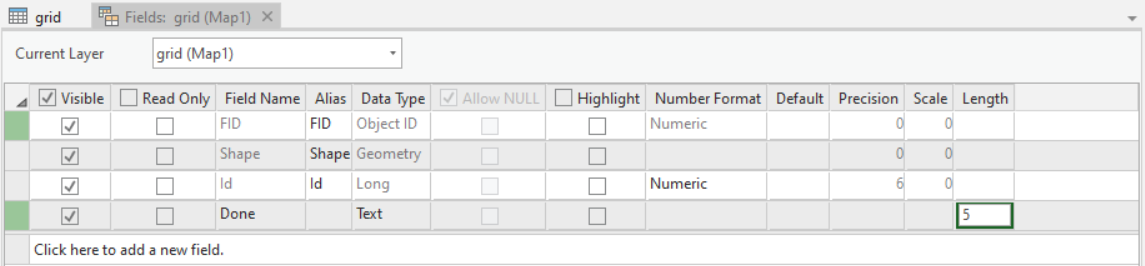


- - - 1. To save your changes, go the “Fields” tab, and select “Save”.


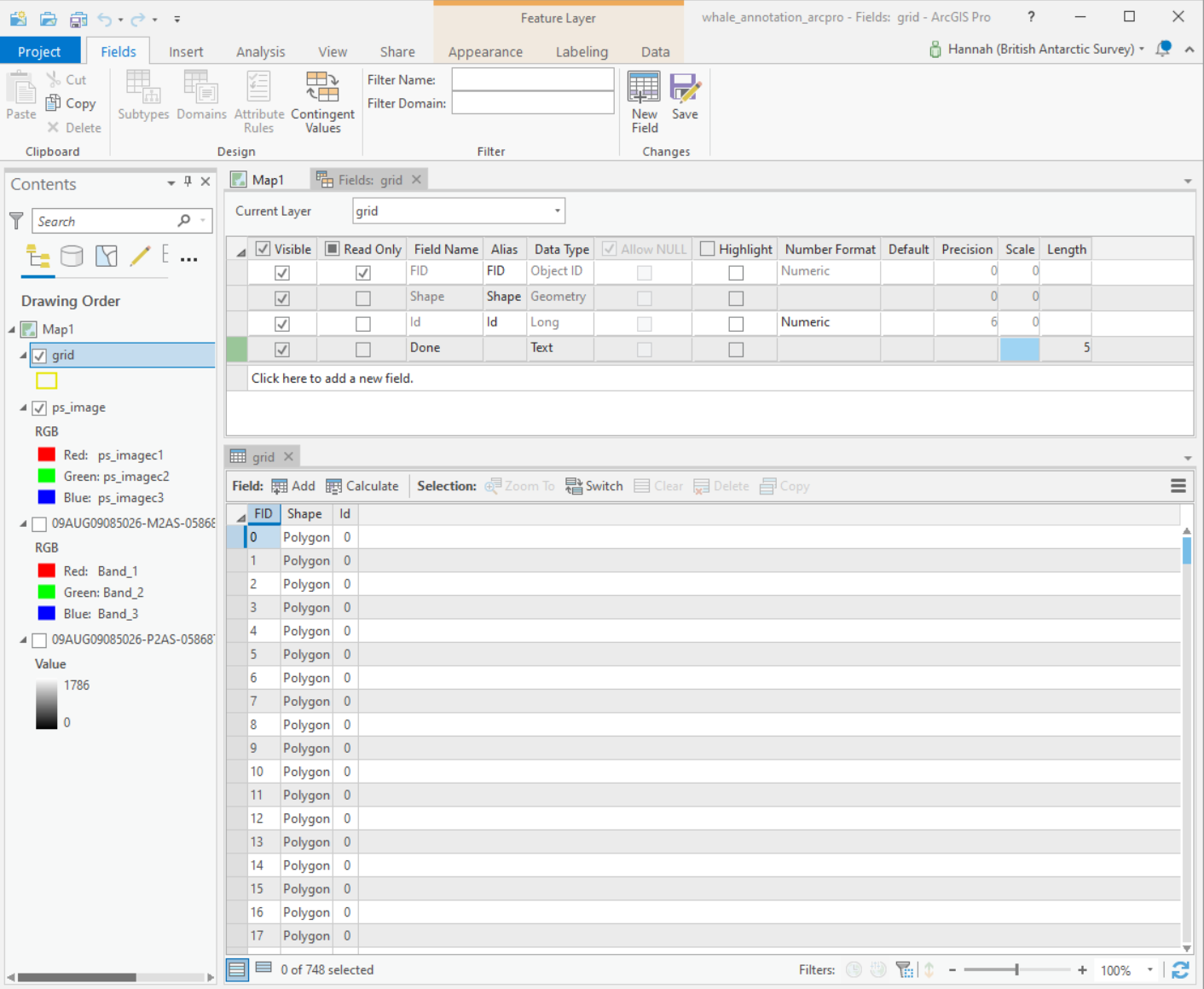


### Creating a point shapefile

1. Create a point shapefile that will be used to annotate the whales you detect in the imagery.
   1. Under the “View” tab, select the “Catalog Pane.


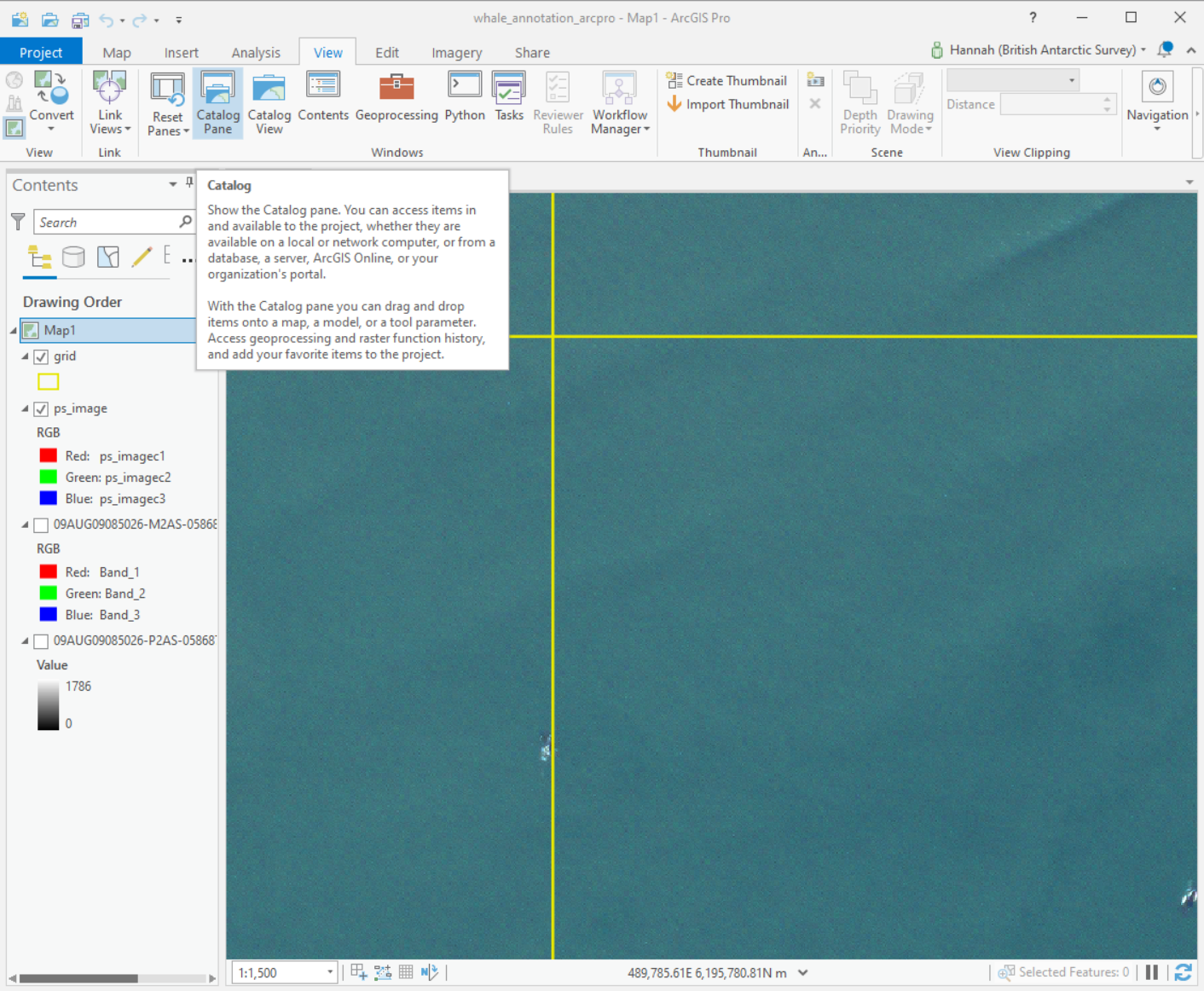


- 1. In the Catalog window, under the “Catalog Tree”, go to the folder you wish to save your file in. If you can’t find your folder, you need to create a connection with the following steps, otherwise go to step 11.3:
     1. Right click on “Folders”, and select “Add Folder Connection”.


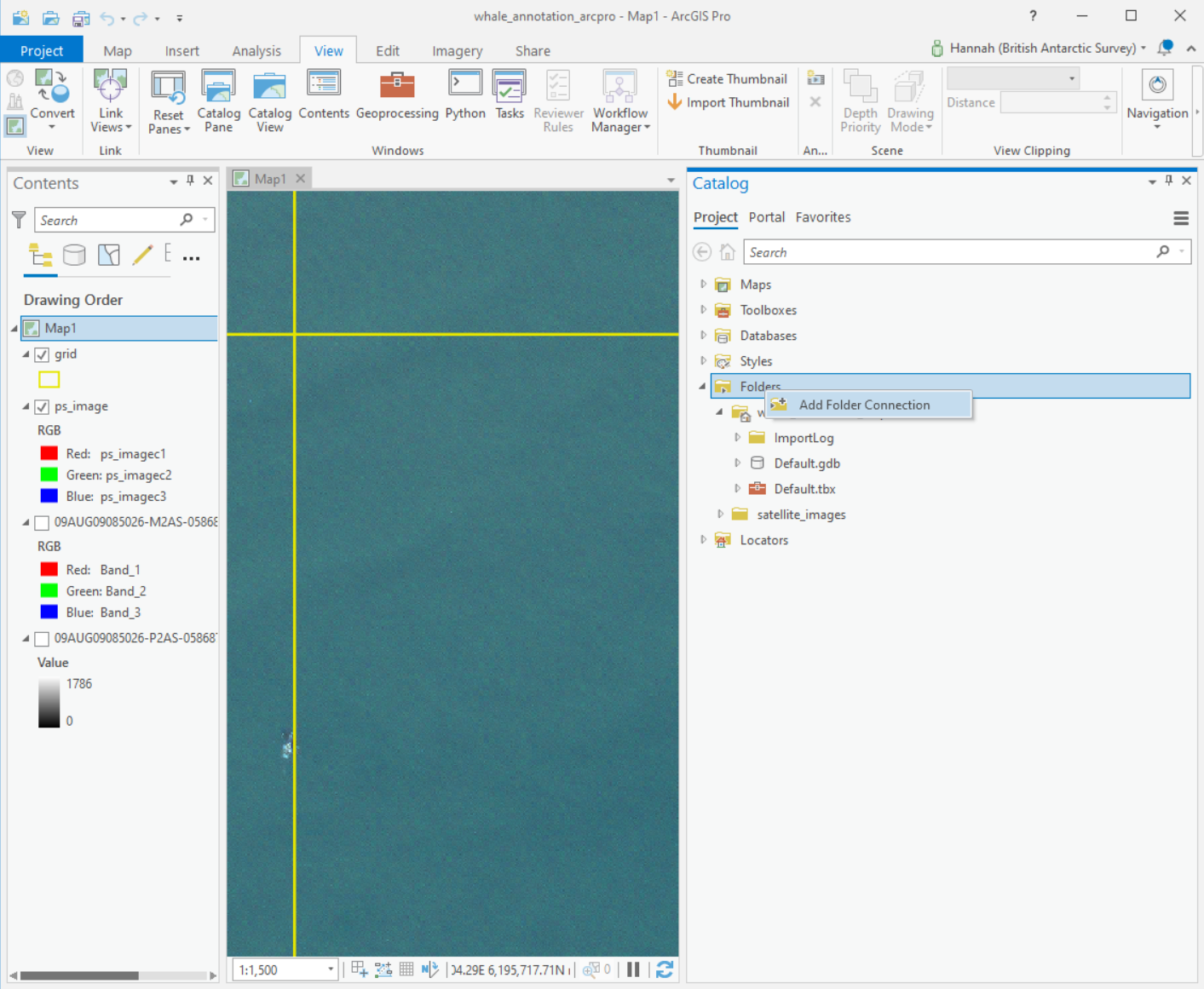


- - 1. In the “Add Folder Connection” window, select the folder you wish to connect to save your files in, and select “OK”. Then go back to step 11.2.
  1. Right click on the folder where you wish to save your file, select “New”, then “Shapefile”.


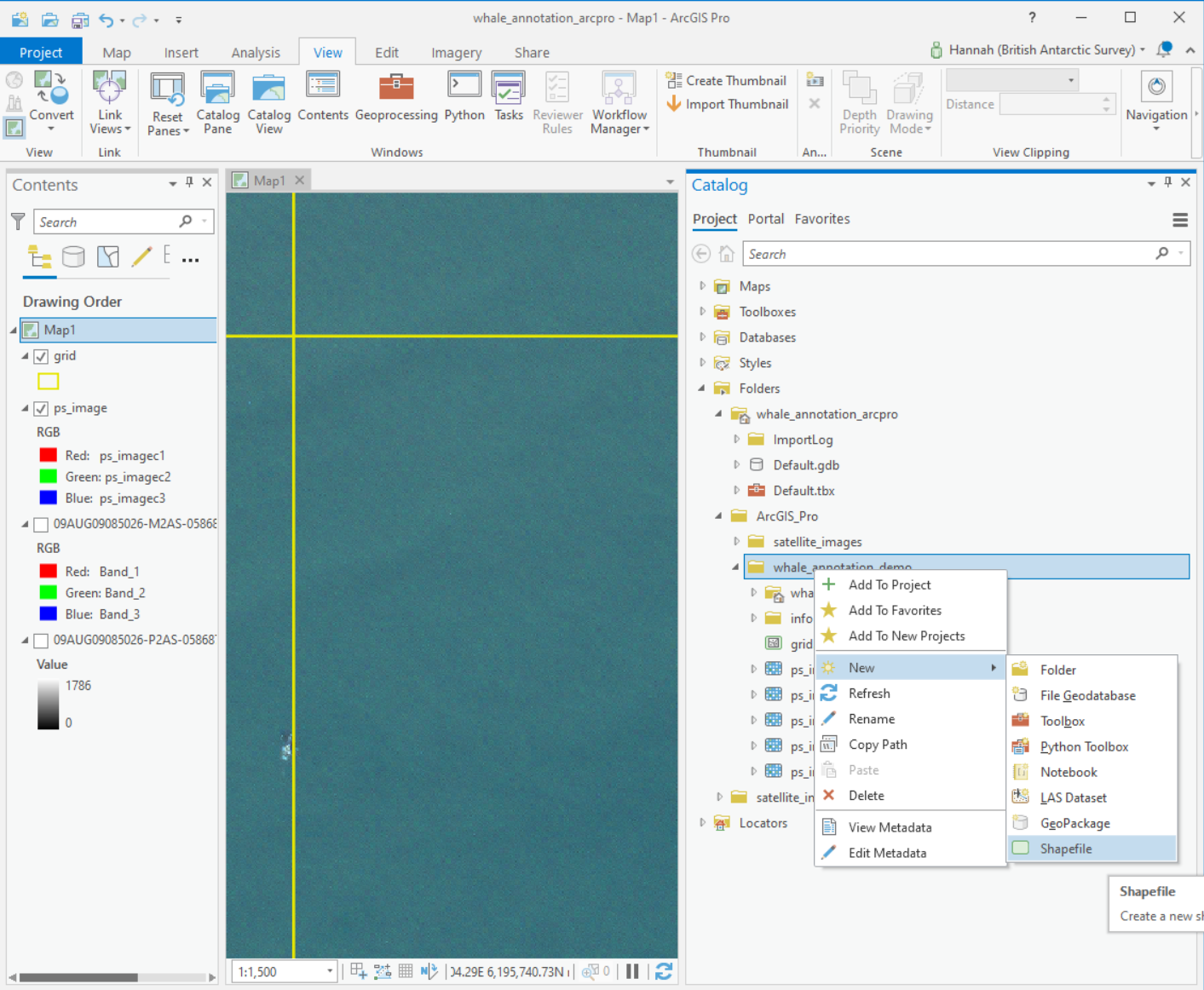


- 1. In the newly opened “Create Feature Class” window, inform the following and select “Run”:
- Feature Class Name: give a name to the shapefile (*e.g.* ‘Whales’)
- Geometry Type: Point
- Coordinate System: Select the same spatial reference used for the pansharpen file (the pansharpen file will be proposed alongside all other files uploaded on the map)


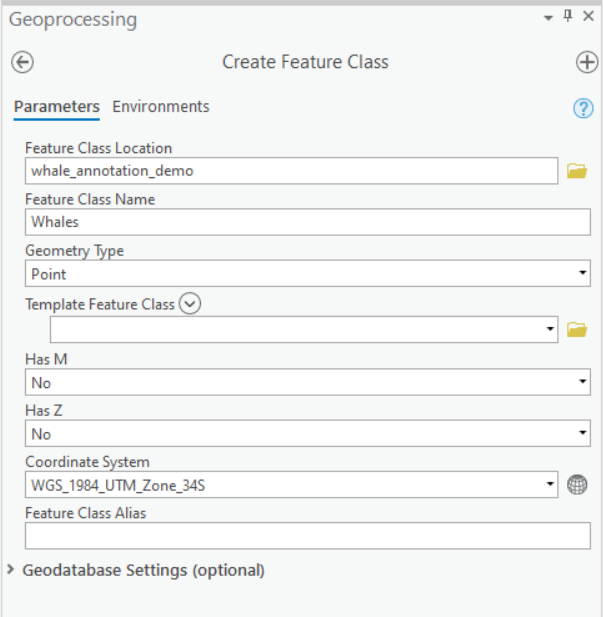


- - 1. Feel free to change the symbology of the points to a different shape and color by double clicking on it.


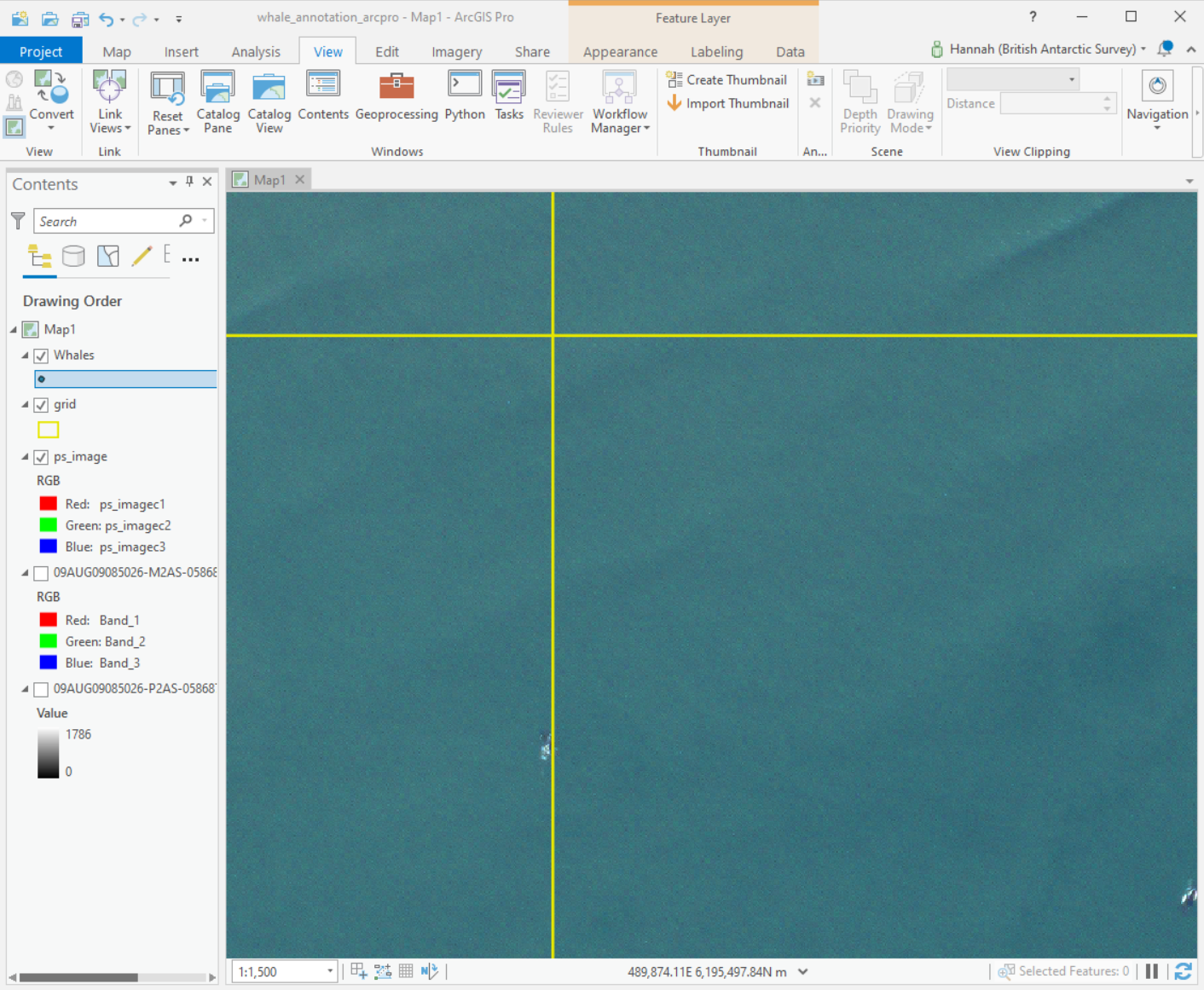


1. Optional: create a point shapefile for other features you would like to record, *e.g.* boats and planes. Repeat steps 11.3. to 11.4.

### Preparing the attribute table

1. Prepare the attribute table attached to the point shapefile you just created. You can either add the field yourself to the attribute table of the point shapefile (steps 13.1. to 13.5.) or you can add a list of pre-made fields (Table 2) to the attribute table to save time, by joining a template attribute table (steps 13.6.).
   1. Open the attribute table. In the “Contents” window, right click on the point shapefile and select “Attribute Table”.


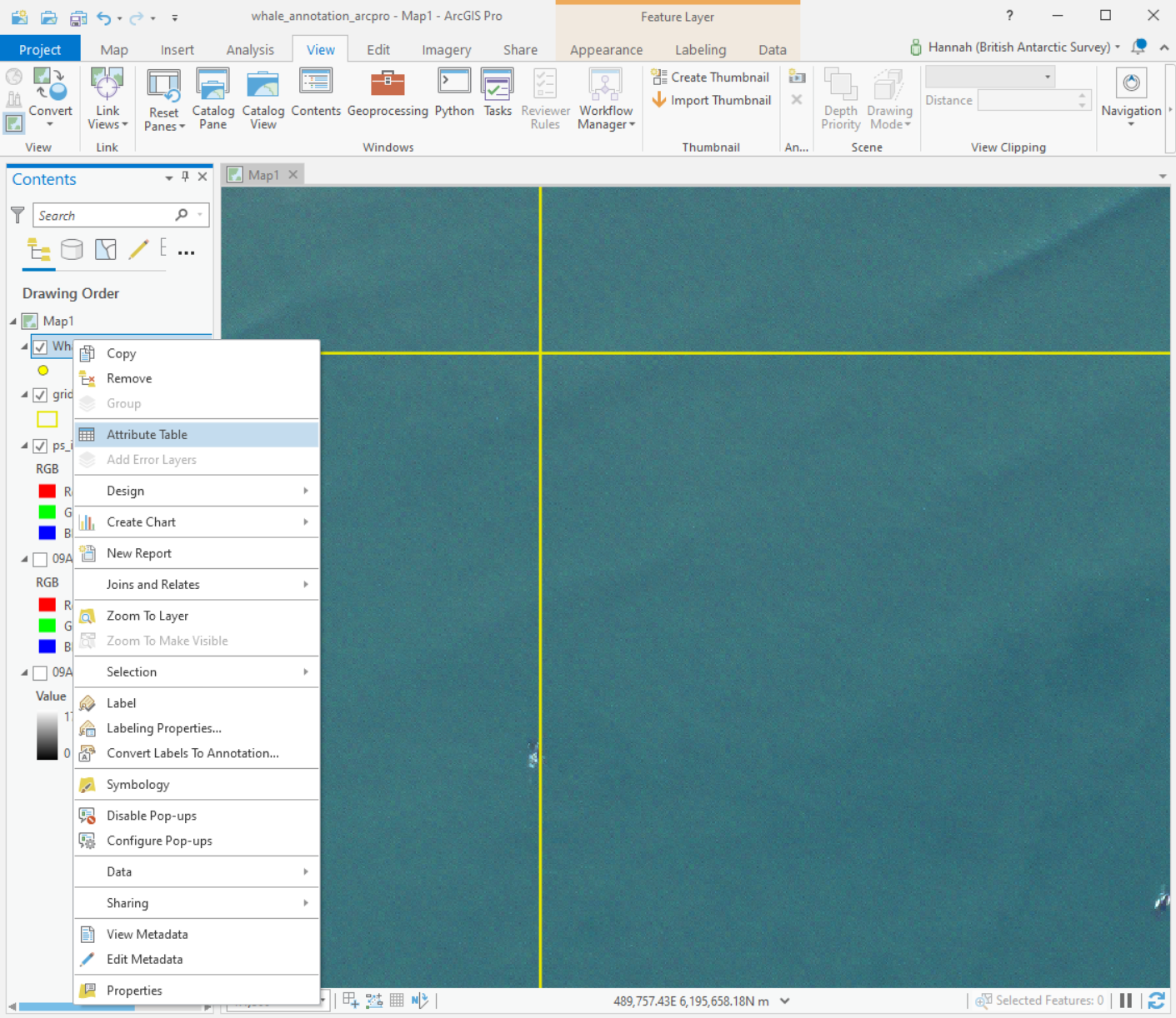


- 1. Under the “Table” window, select the “Add Field” icon.


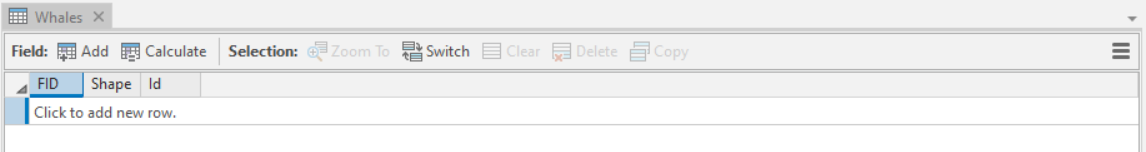


- 1. Fill in the “Fields” window using the information provided in Table 2 (see Table 3 for a description of each field). To keep adding new fields, select “Click here to add a new field.” below the table of fields. At the end your table should look something similar to this:


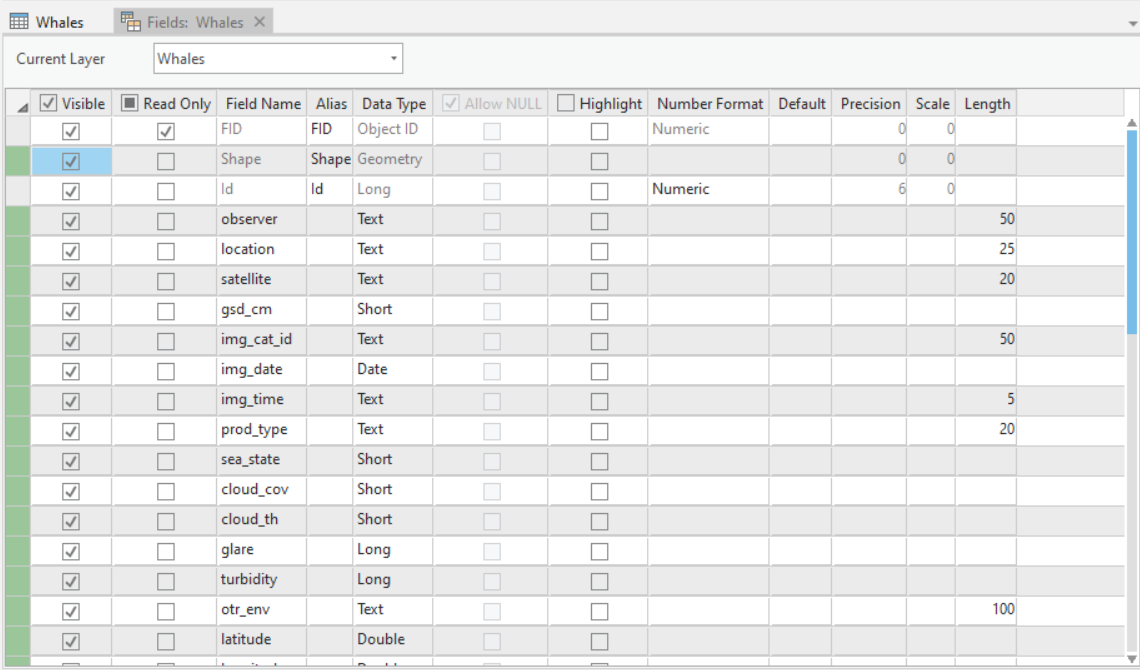


**Table 2.** Information necessary to fill the “Add Field” window, each row represents a different Field.

| Name | Type | Field properties |
| --- | --- | --- |
| observer | Text | Length: 50 |
| location | Text | Length: 25 |
| satellite | Text | Length: 20 |
| gsd_cm | Short (short integer) | NA |
| img_cat_id | Text | Length: 50 |
| img_date | Date | NA |
| img_time | Text | Length: 5 |
| prod_type | Text | Length: 20 |
| sea_state | Short (short integer) | NA |
| cloud_cov | Short (short integer) | NA |
| cloud_th | Short (short integer) | NA |
| glare | Short (short integer) | NA |
| turbidity | Short (short integer) | NA |
| otr_env | Text | Length: 100 |
| latitude | Double | NA |
| longitude | Double | NA |
| gcs | Text | Length: 50 |
| projection | Text | Length: 50 |
| sp_code | Text | Length: 50 |
| certainty | Text | Length: 10 |
| body_color | Text | Length: 50 |
| body_shp | Text | Length: 50 |
| body_l | Short (short integer) | Precision: 0 |
| body_w | Short (short integer) | Precision: 0 |
| flipper | Short (short integer) | Precision: 0 |
| lg_flipper | Short (short integer) | Precision: 0 |
| fluke | Short (short integer) | Precision: 0 |
| head_callo | Short (short integer) | Precision: 0 |
| wh_lr_jaw | Short (short integer) | Precision: 0 |
| aft_breach | Short (short integer) | Precision: 0 |
| bubble_net | Short (short integer) | Precision: 0 |
| contour | Short (short integer) | Precision: 0 |
| flukeprint | Short (short integer) | Precision: 0 |
| wake | Short (short integer) | Precision: 0 |
| blow | Short (short integer) | Precision: 0 |
| mudtrail | Short (short integer) | Precision: 0 |
| sur_act_gr | Short (short integer) | Precision: 0 |
| travel_gr | Short (short integer) | Precision: 0 |
| mc_pair | Short (short integer) | Precision: 0 |
| otr_gr | Short (short integer) | Precision: 0 |
| defecation | Short (short integer) | Precision: 0 |
| comment | Text | Length: 200 |

- 1. After having added all the fields listed in Table 2, go to the “Fields” tab and select “Save”. Then you can close the “Fields” and attribute table windows.


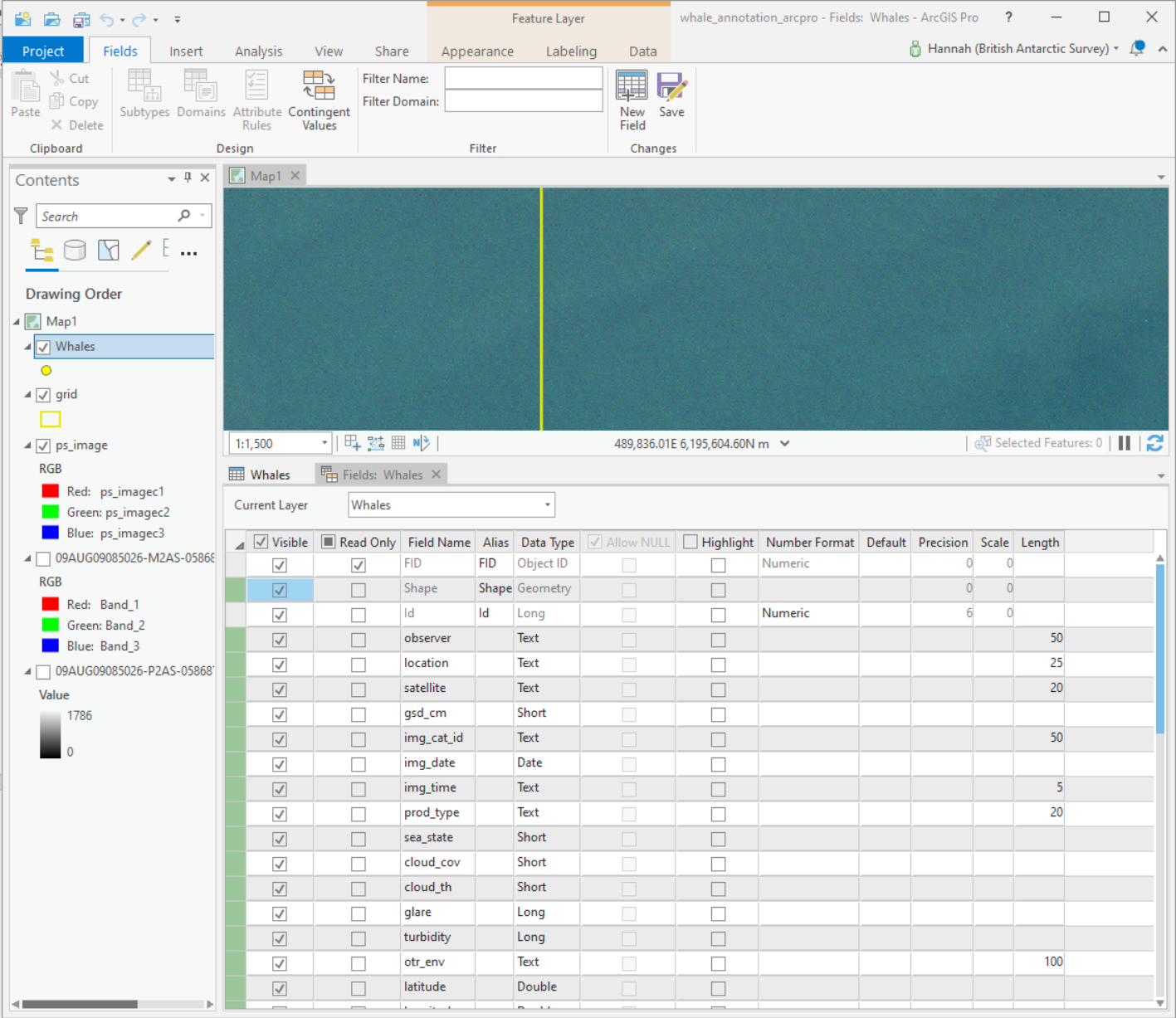


- 1. If you wish to export your attribute table, have your attribute table open, then:
     1. Select the “Table” tab, and select “Export Table”.


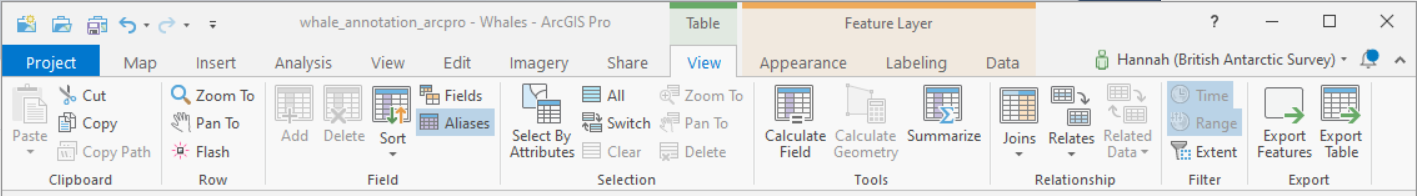


- - 1. Or, in the attribute table window, select the “Option” icon and select “Export”.


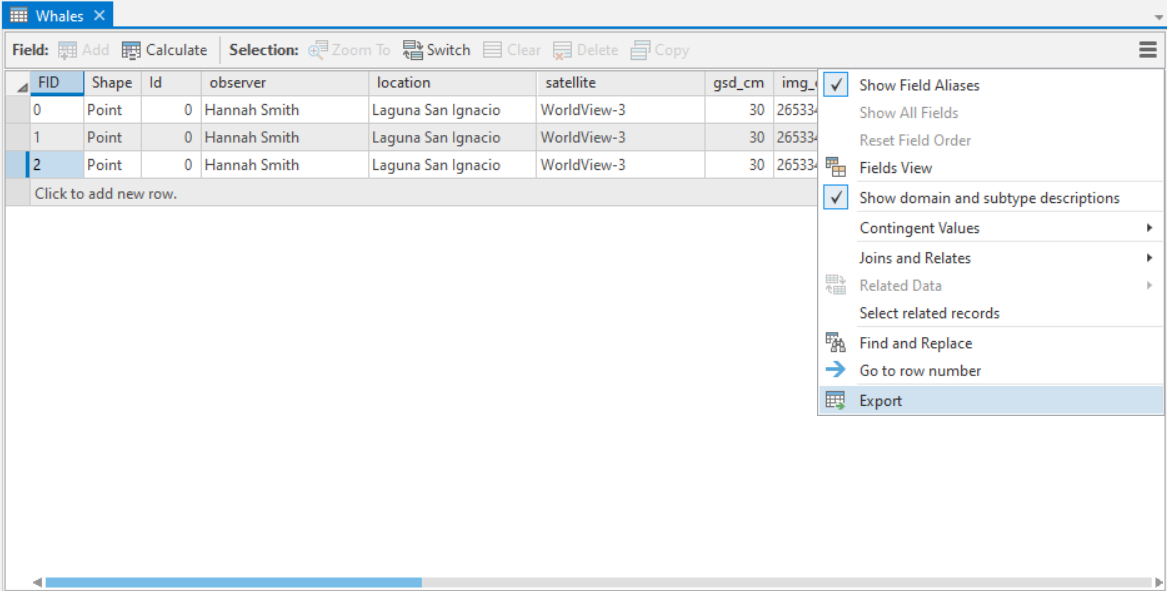


- - 1. Under the “Copy Rows” window, fill in the following information, then select “Run”:
- Input Rows: select the shapefile with the attribute table you wish to export
- Output Table: select the location you wish to export it to and type in the name followed by “.csv” to save as an csv file


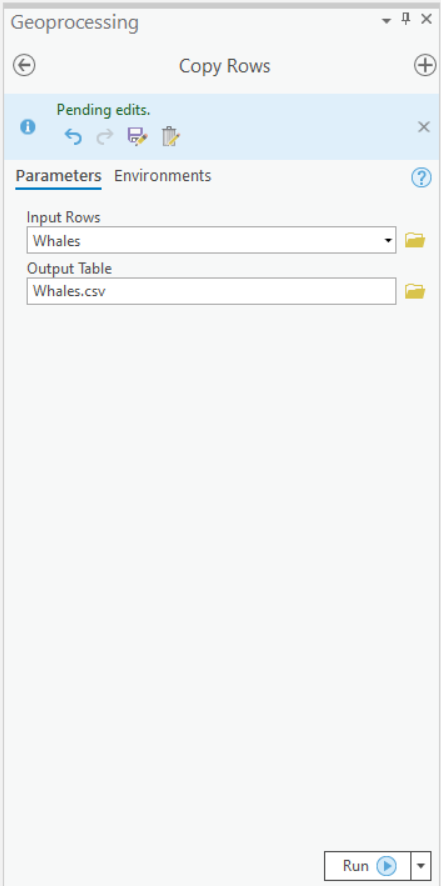


- 1. If you wish to add the same attributes as in Table 2 faster, you can join the template attribute table (Supplementary material 6) to your point shapefile.
     1. Import the “Template_AT” shapefile into your map.
     2. Use “Join Field” tool, which you can access by selecting “Tools”, under the “Analysis” tab.
        1. Under the “Geoprocessing” window, type “join” and select the top option “Join Field (Data Management)”.


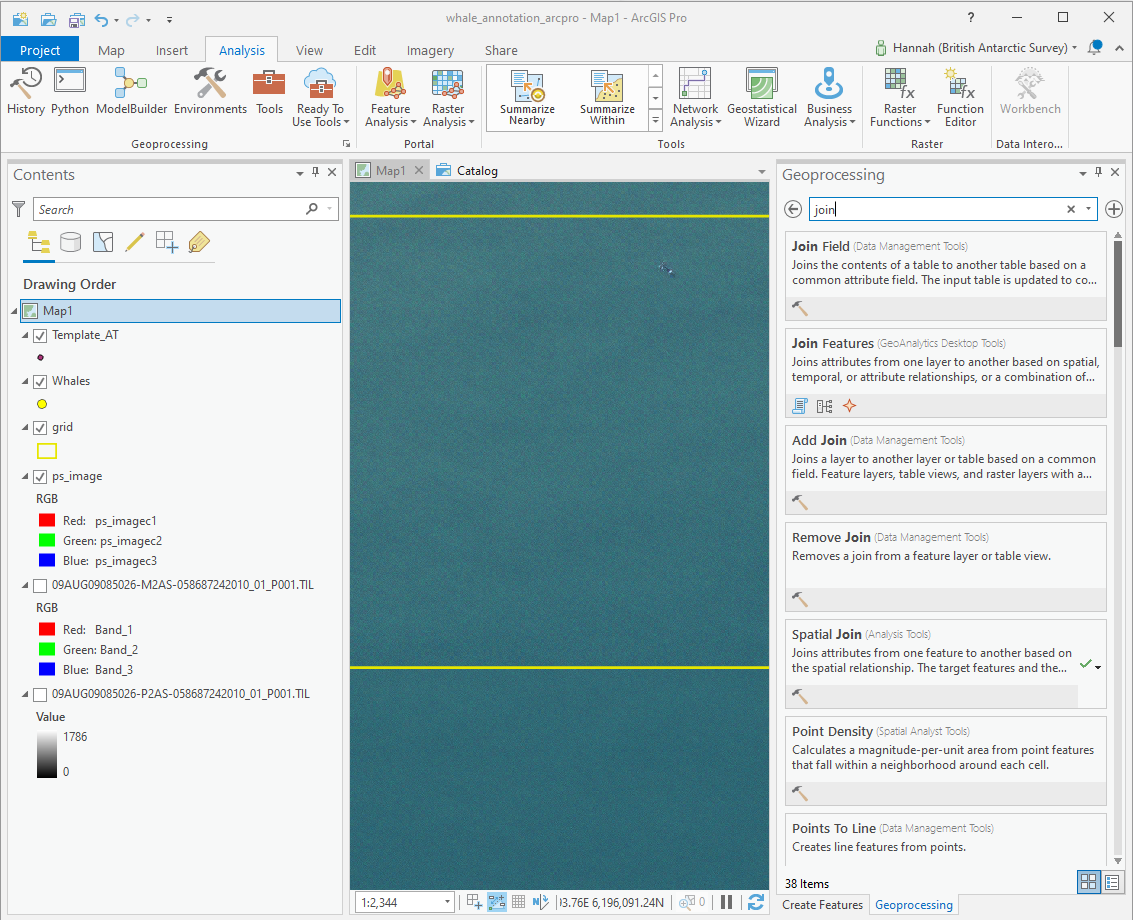


- - - 1. In the “Join Field” window, fill in the following information, then select “Run”:

Input Table: the shapefile you created at step 11

Input Join Field: FID

Join Table: “Template_AT” shapefile (Supplementary material 6)

Output Join Field: FID


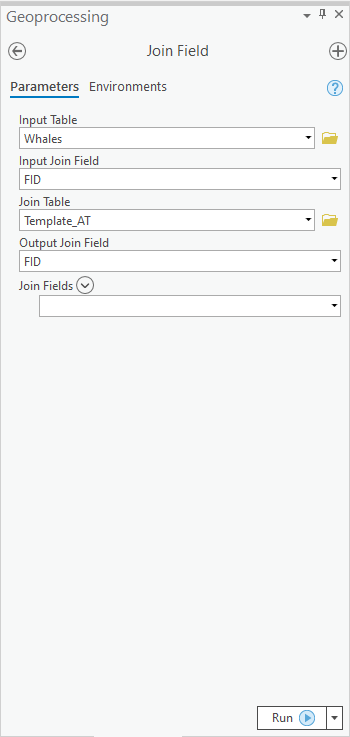


# Reviewing the Image

## Systematic scanning

Scanning the image for the presence of whales should be systematic; therefore, we recommend overlaying the grid created at step 10.5 on top of the pansharpened image, and reviewing one cell after another as shown below.


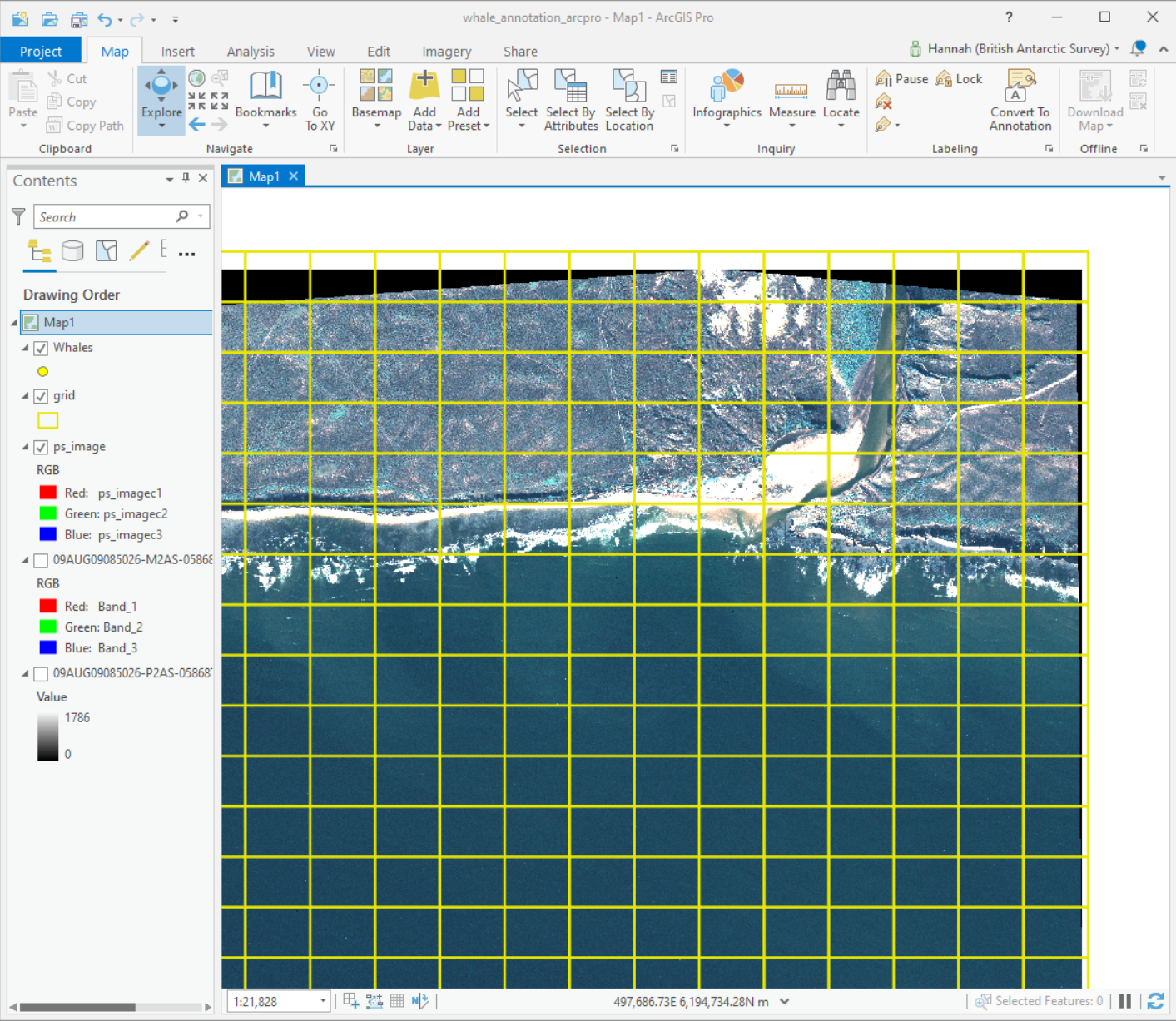


## Scale

We also recommend scanning the image at the scale at which the grid was created (*e.g.* 1:1,500 for large whales).

## Keeping track

To keep track of which cells were reviewed, we recommend filling the field “Done” in the attribute table after a couple rows or more or less frequently depending on the shape of the image that is being reviewed.

1. Open the attribute table of the grid shapefile by right clicking on the file and selecting “Attribute Table”
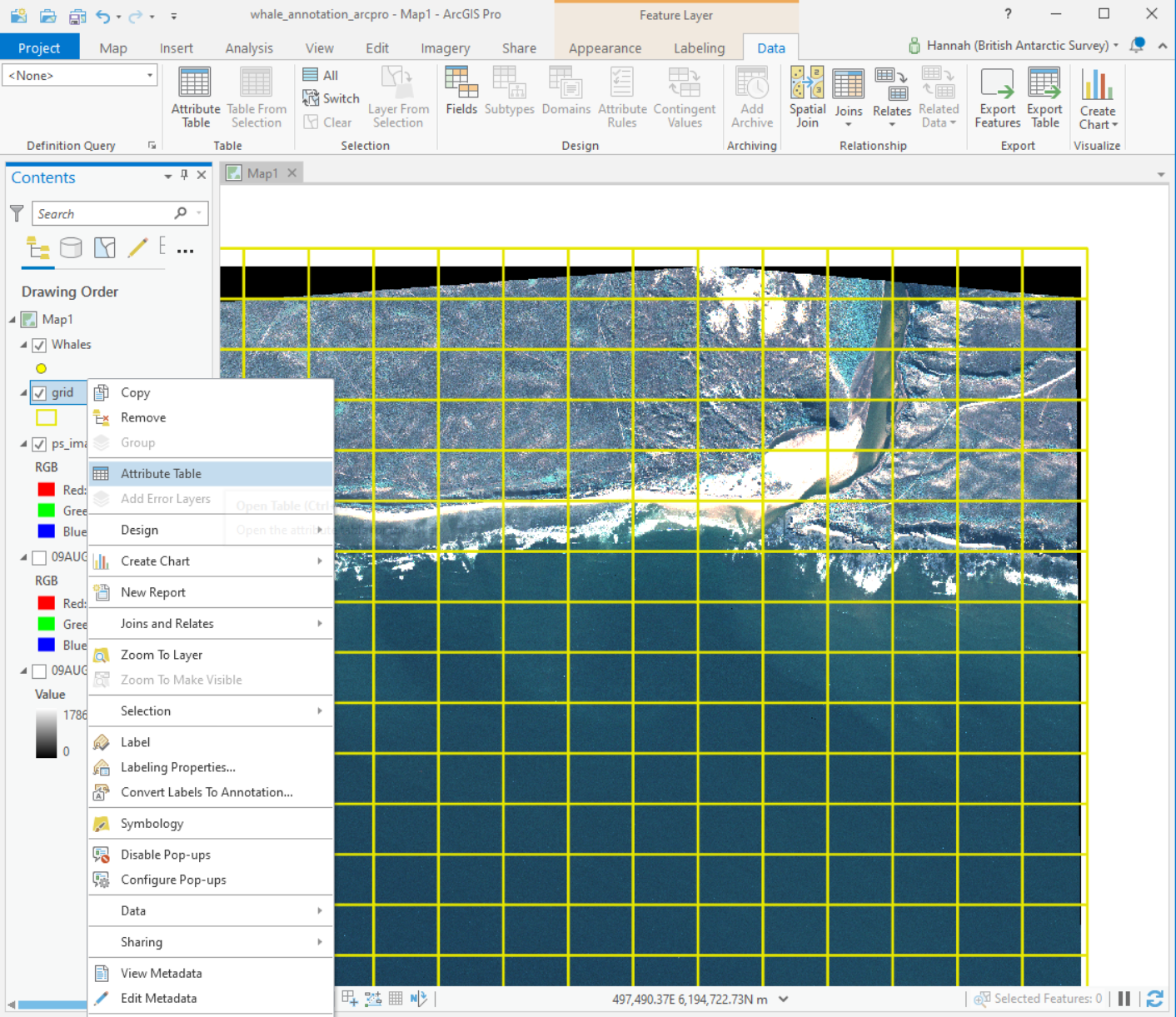

   1. You can now write in each cell of the attribute table. We recommend typing “Yes” for the cell of the grid that have been reviewed. If you fill the information after doing a whole row for example, you can copy/paste “Yes” to all the cells you have reviewed.


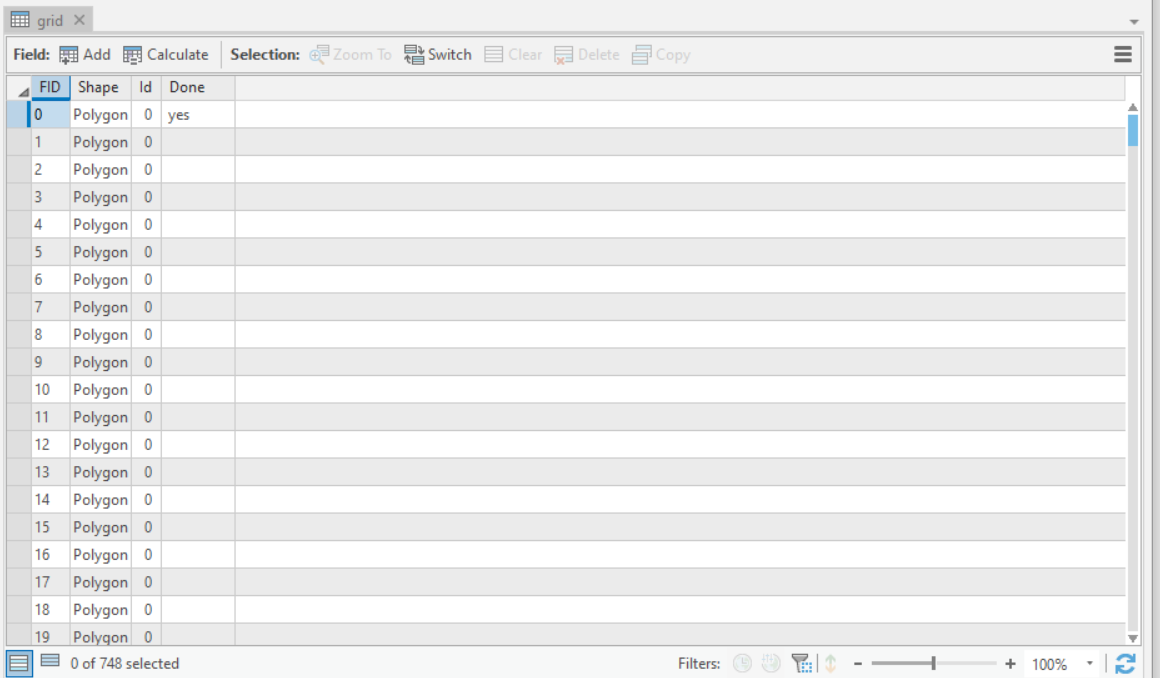


- 1. Make sure to save your edits. In the “Edit” tab, select “Save”.


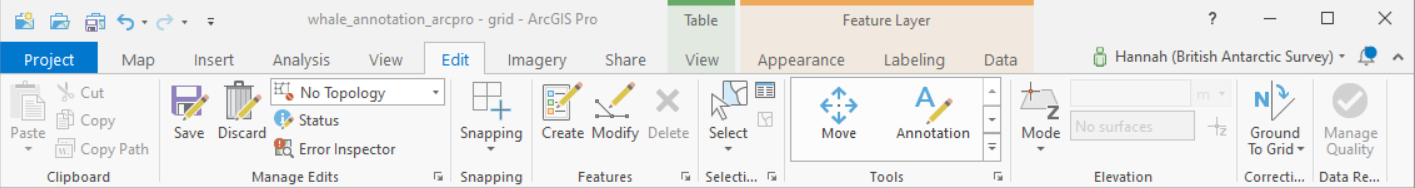


- 1. You can update the symbology of the grid shapefile to show the cells of the grid review in a different color than the cells that need reviewing.
     1. In the “Contents” window, right click on the grid shapefile and select “Symbology”. Under the “Symbology” window, update the following information:
- Primary Symbology: Unique Values
- Field 1: Done
- Classes: update the symbology of the “Yes” and empty values by double clicking on the polygon, we recommend to use a no fill and colored outline for the empty value, and a transparency fill and colored outline for the “yes”.


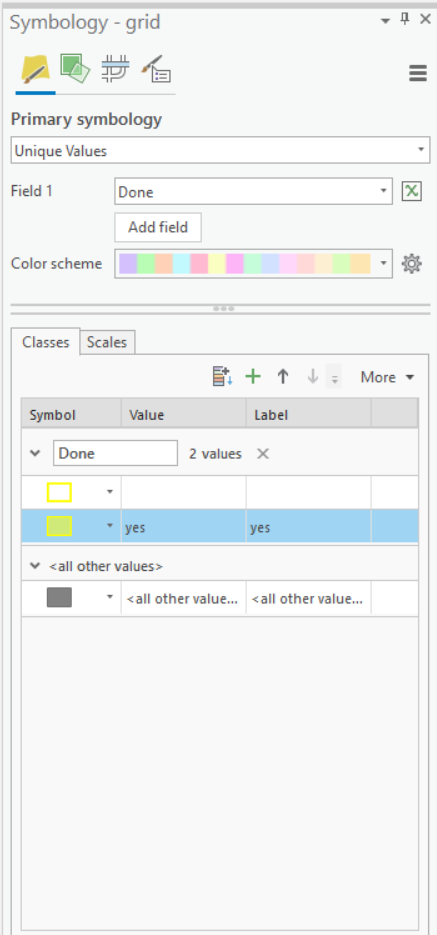


# Annotating

You are now all set up to start scanning the image and annotating the image when you find whales (or your feature of interest, such as confounding features).

## Placing points on top of whales

1. To be able to a place point on top of the whales you have found, select “Create” under the “Edit” tab.


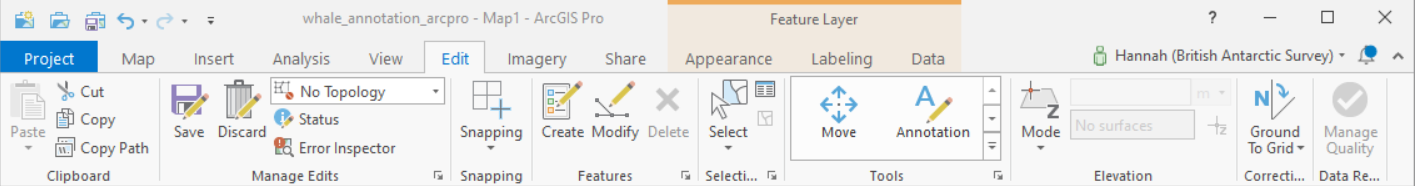


1. Under the “Create Features” window, select the whale shapefile created in step 11.4, then select the “Point” icon.


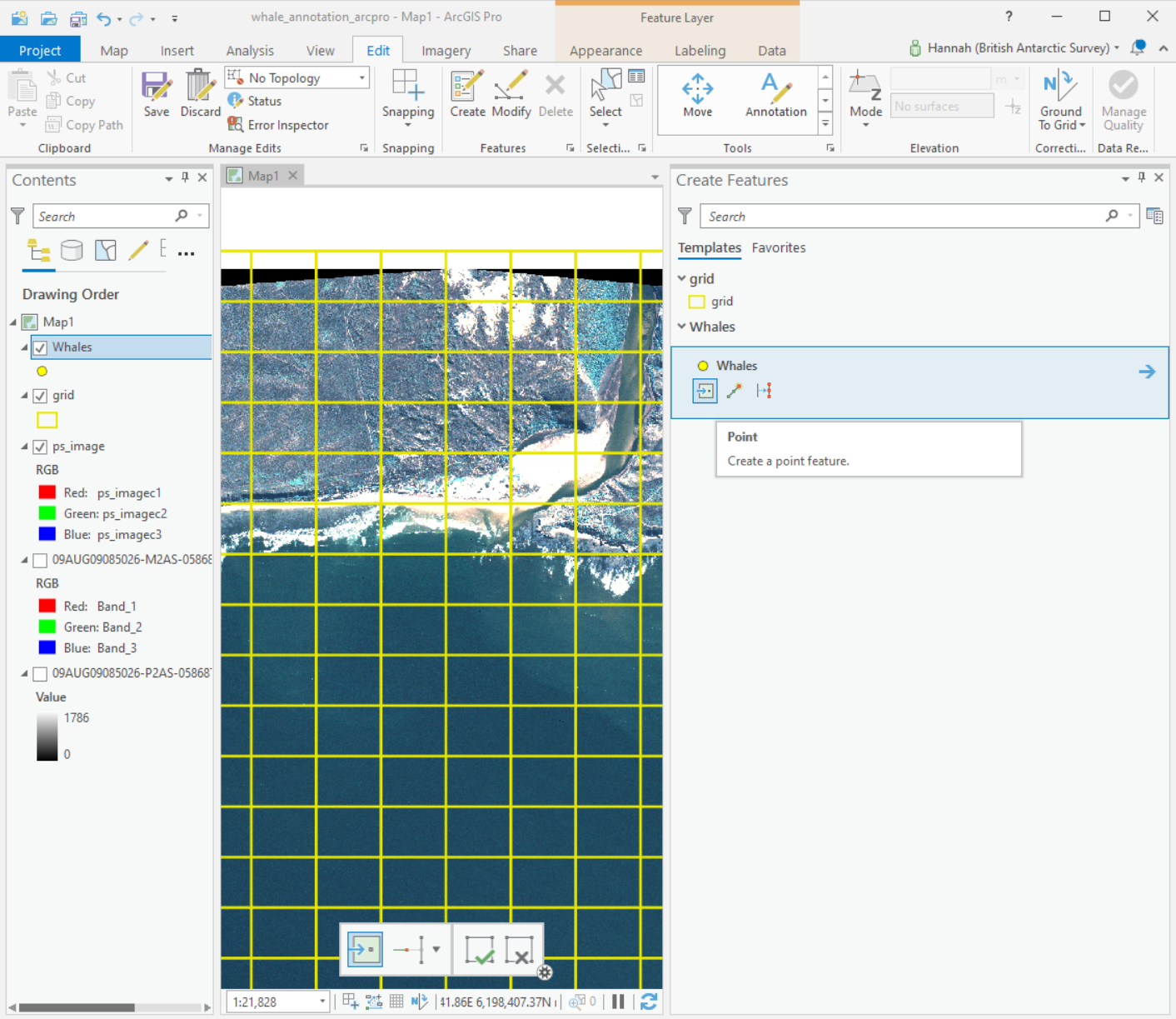


1. You are now ready to place a point on the image, by clicking in the middle of the whale.

**IMPORTANT:** it is crucial that the point is in the center of the visible part of the whale to facilitate joining the annotations of different observers.


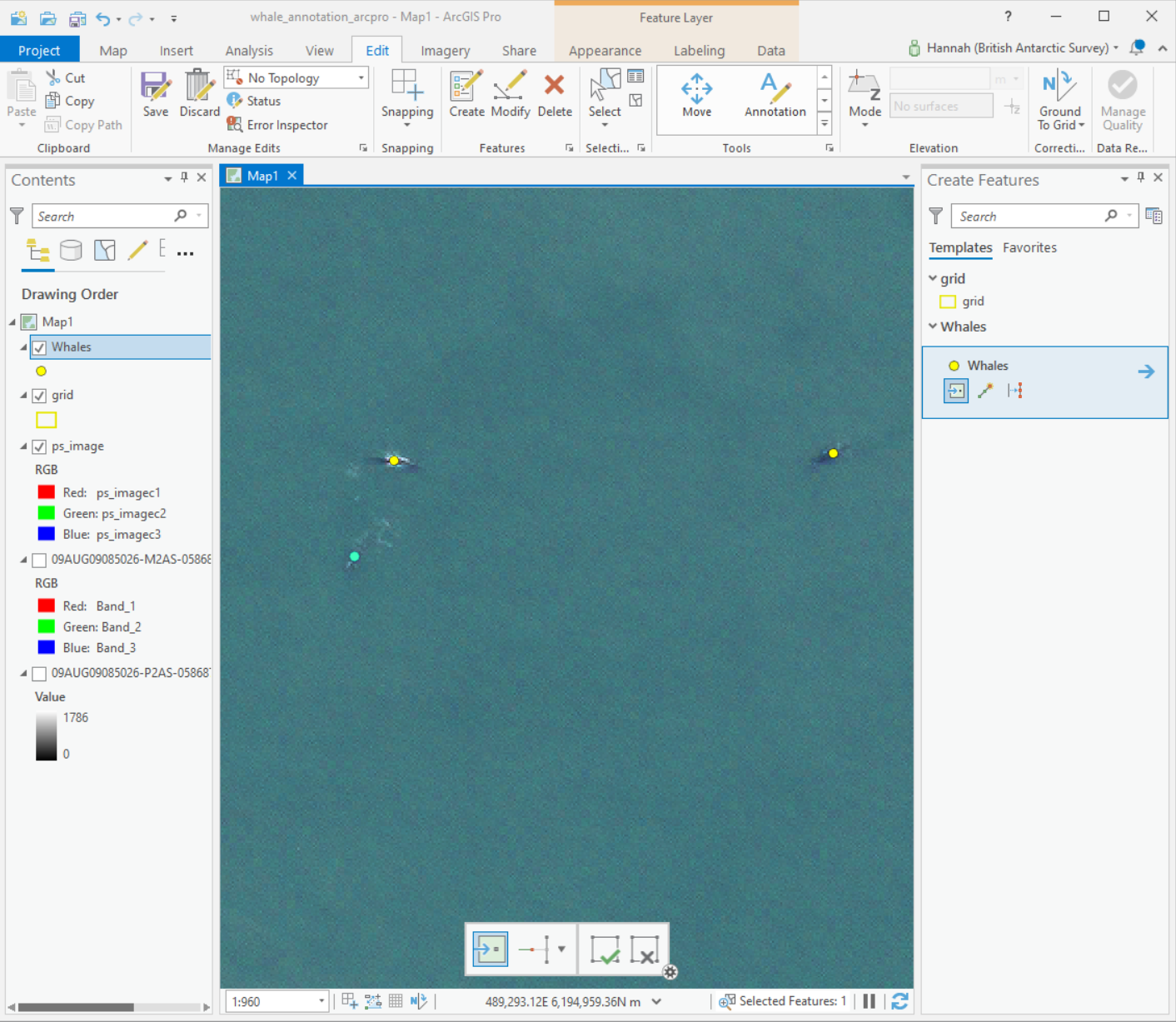


1. Make sure to save your edits, frequently, by selecting “Save” under the “Edit” tab.


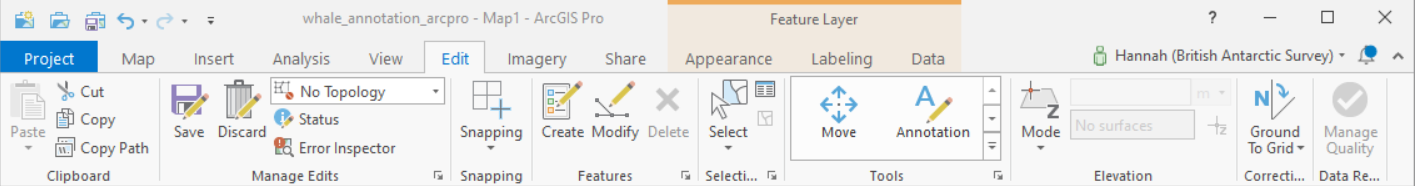


## Filling in the attribute table

Once you have fully scanned the image and placed points in the center of the detected whales, you can fill in the attribute table. Refer to Table 3 to know what information should go under each Field.

1. Open the attribute table, right click on the whale shapefile and select “Attribute Table”.


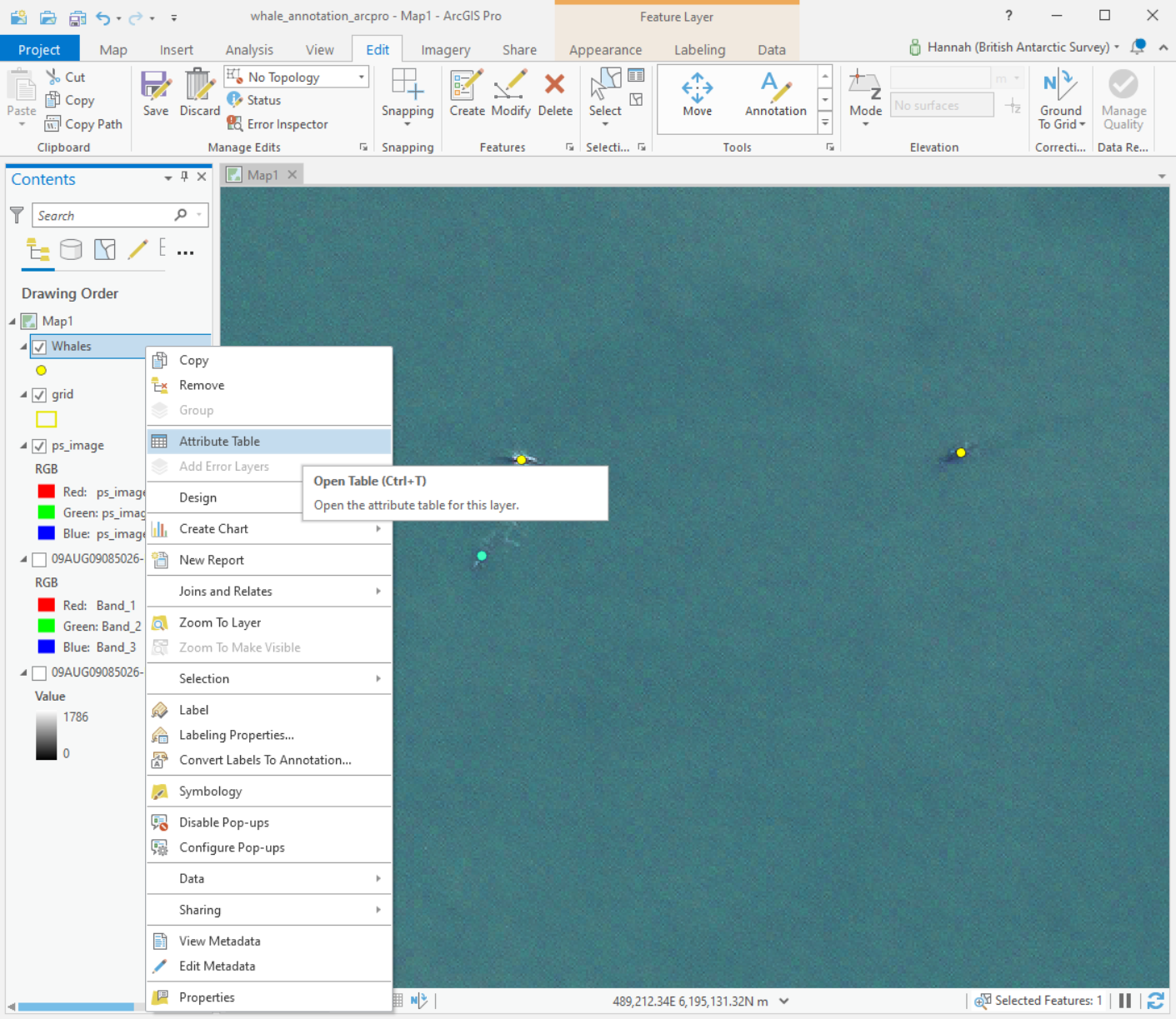


1. Fill in the different fields for each whale points.


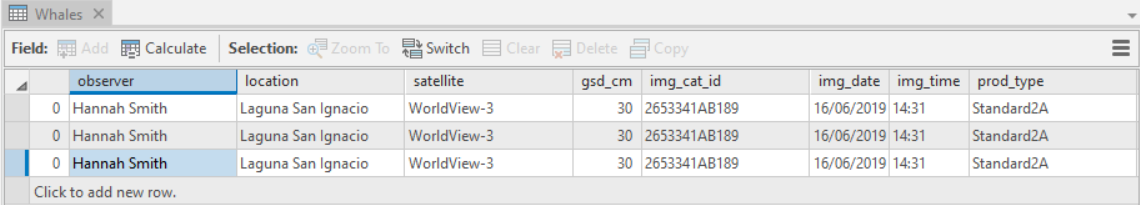


**TIPS:** to fill in the information quicker:

- you can use the “Calculate Field” tool for the following fields as they will contain the same information for all whale points: observer, location, satellite, gsd_cm, img_cat_id_img_date, prod_type, sea_state, cloud_cov, cloud_th, glare, turbidity, otr_env, gcs, projection. Right click on the name of the field, e.g. satellite as shown below and select “Calculate Field”.


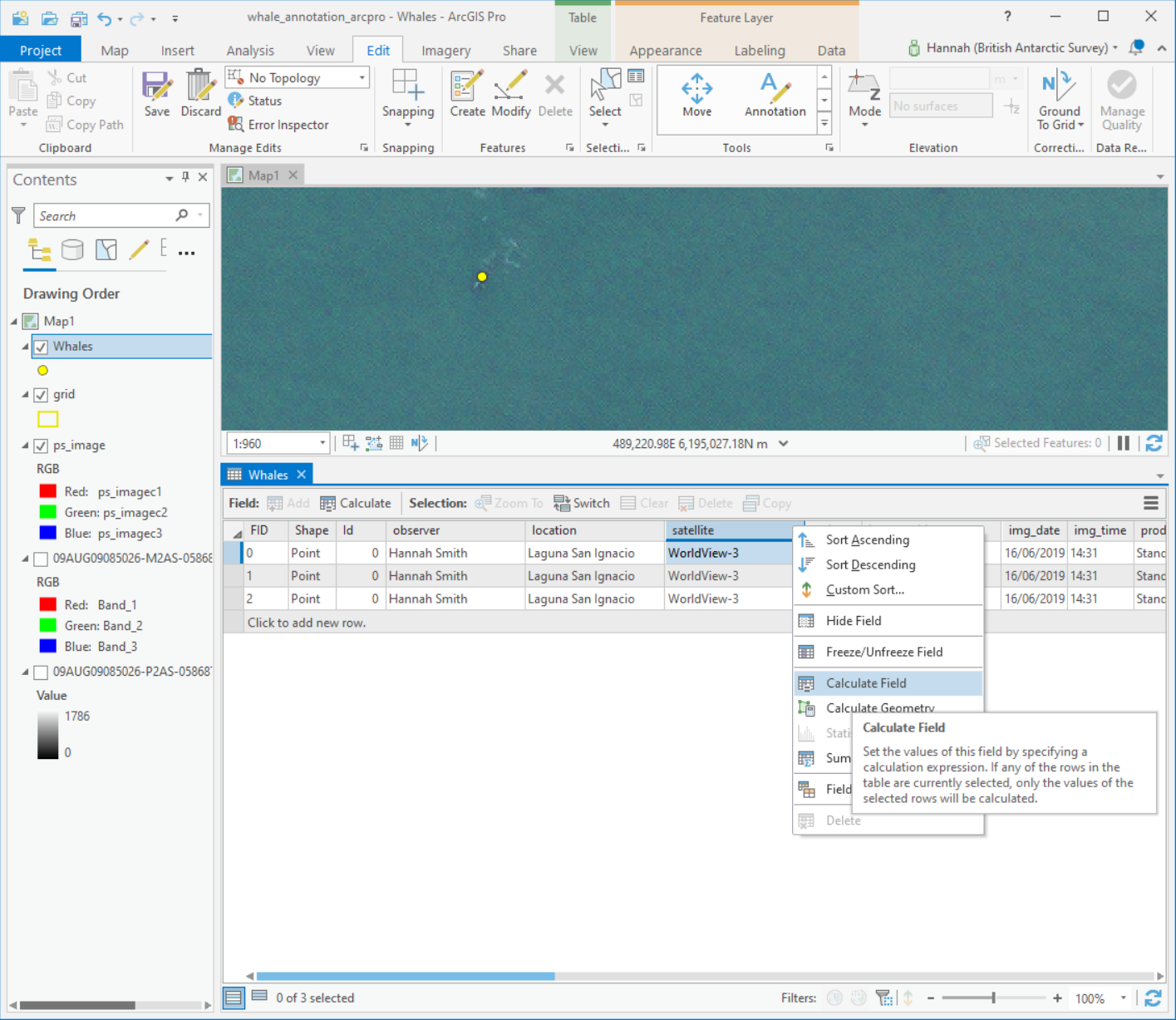


Under the “Calculate Field” window, you can write the value that will be the same through the whole field, e.g. for the Field “satellite”, you can type “WorldView-3” under the “Code Block” and select “Run”, as shown below and it will automatically populate every row in that column with the value “WorldView-3”.


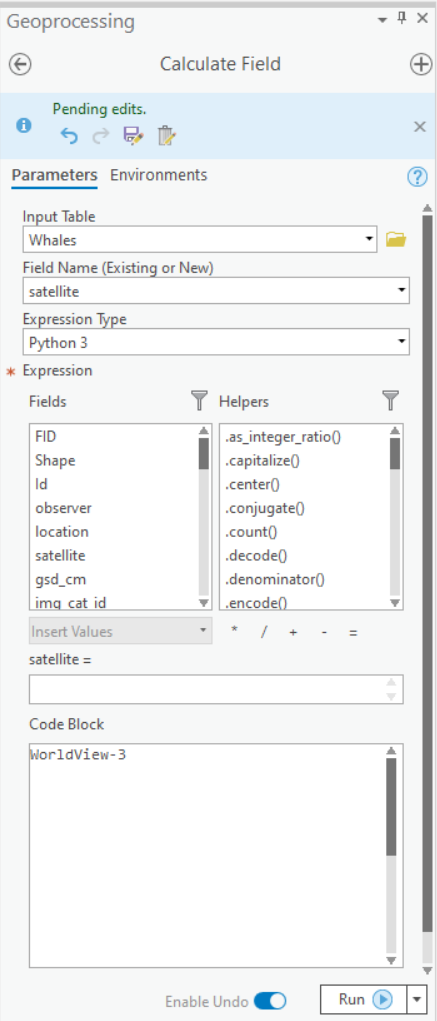


- you can use the “Calculate Geometry” tool, for the fields: latitude, longitude. Right click on the name of the field, e.g. latitude as shown below and select “Calculate Geometry”.


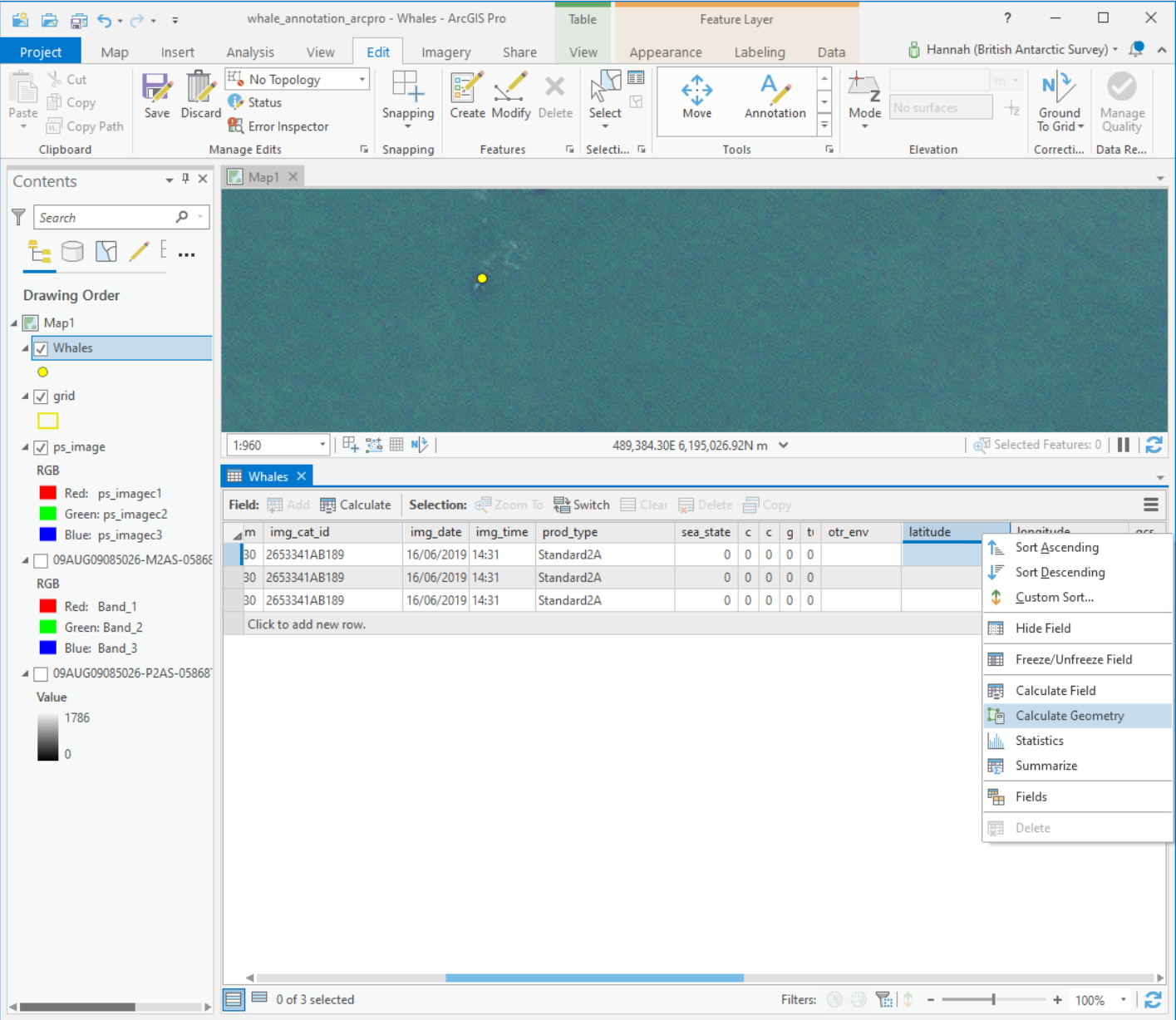


Under the “Calculate Geometry” window, enter the following information for latitude:

- - Property: Y Coordinate of Point

And the following information for longitude:

- - Property: X Coordinate of Point

For both latitude and longitude, choose “Decimal Degrees” for the “Coordinate Format”. Then select “Run”.


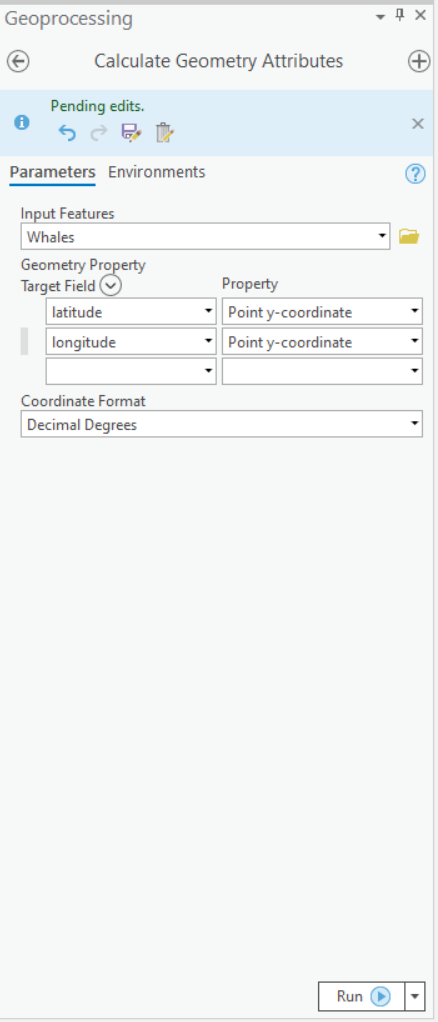


1. Make sure to save your edits, frequently, by selecting “Save” under the “Edit” tab.


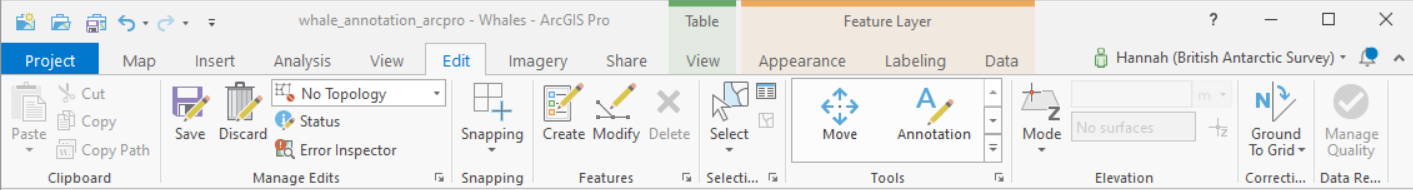


**Table 3.** Description of each Field contained in the attribute table with information on how to fill in the information for each Field.

| Field | Description | Unit | Value | Format |
| --- | --- | --- | --- | --- |
| observer | Name of person reviewing the image. | None | Letters | *e.g.* Hannah Smith |
| location | Name of the location where the satellite image was captured. | None | Letters | *e.g.* Laguna San Ignacio  *e.g.* Cape Cod |
| satellite | Name of the satellite that captured the image. | None | Letters and numbers | *e.g.* Worldview-3 |
| gsd_cm | The ground sampling distance (the distance between the center points of each pixel), which can be found in the metadata, by right clicking on the panchromatic file and selecting “Properties”, then “Source” and “Raster Information”. | Centimeter | Numbers | *e.g.* 3 |
| img_id | Unique identification that the satellite imagery provider assigns to each image. With Maxar, this corresponds to the catalog ID. | None | Letters and numbers | *e.g.* 10400ED2959 |
| img_date | Date the image was captured. | Day, month, year | Numbers | dd/mm/yyyy |
| Img_time | Time the image was captured. | Hours and minutes | Numbers | hh:mm |
| prod_type | The product type indicates the level of pre-processing an image has gone through when it was acquired from the satellite imagery provider, such as projection. See Table 1 for the various product type offered by the main VHR satellite imagery providers. | None | Letters and numbers | *e.g.* Standard 2A |
| sea_state | Sea state adapted from Figure 4 in Bamford et al (2020).  **1= Good** (minimal swell, no white caps, no wavelets)  **2 = Moderate** (minimal swell, sparse white caps, few wavelets)  **3 = Average** (slight swell, intermittent wavelets, no or very few white caps)  **4 = Sub-average** (medium swell, apparent waves, several white caps).  **5 = Poor** (significant swell, directional surface wind, large wave, several white caps) | None | 1 to 5 | *e.g.* 1 |
| cloud_cov | Cloud cover for the whole image, using the aviation system:  0 = SKC (sky clear)  1-2 = FEW (traces)  3-4 = SCT (scattered)  5-7 = BKN (Broken)  8 = OVC (Overcast) | Okta | 0 to 8 | *e.g.* 0 |
| cloud_th | Cloud thickness for the clouds present in the image  1 = Thin (can see fairly well through the cloud)  2 = Medium thin (can see through but no clear view of the sea)  3 = Thick (can’t see through)  4 = mix of thin, medium, thick clouds | Number | 1 to 4 | *e.g.* 1 |
| glare | Proportion of glare in the whole image:  0 = None  1 = Mild  2 = Moderate  3 = Severe | Number | 1 to 3 | *e.g.* 1 |
| turbidity | Qualitative estimations of the level of turbidity:  1 = Non-turbid  2 = Moderate  3 = Turbid  4 = mix of turbid and non-turbid waters | Number | 1 to 4 | *e.g.* 1 |
| otr_env | Other environmental conditions that the observer think might limit the visibility of whales (*e.g.* dark image for polar regions from autumn to spring) | None | Letters | *e.g.* dark image |
| latitude | Latitude of the whale detection | Decimal degree | Numbers | *e.g.* 67.50 |
| longitude | Longitude of the whale detection | Decimal degree | Numbers | *e.g.* 12.70 |
| gcs | Geographical coordinate system, it can be found in the metadata. | None | Letters and numbers | *e.g.* WGS 1984 |
| projection | Projection applied to the image to remove distortion | None | Letters and numbers | *e.g.* WGS 1984 UTM S12 |
| sp_code | Species code for the species or the next higher taxonomic level, see Appendix 1 (Supplementary material 3) to help you decide, and Appendix 2 (Supplementary material 4) for the code to use. | None | See Appendix 2 (or Supplementary material 4) | *e.g.* *Eubalaena glacialis* |
| certainty | Certainty of the assignment of the species or the next higher taxonomic level. See Appendix 3 (Supplementary material 5)to help you decide.  1 = **Definite**: you are confident in your species determination (90-100% confidence)  2 = **Probable**: you think that your species determination is likely but you are not sure (60-90% confidence)  3 = **Possible**: you think that your species determination is possible but it is hard to tell (10-60% confidence) | None | 1 to 3 | *e.g.* 1 |
| body_color | Body color of the whale when at the surface (dorsally when viewed in VHR satellite imagery). | None | Letters | *e.g.* brownish gray |
| body_shp | Overall shape of the body excluding fluke and flippers. | None | Letters | *e.g.* streamlined, sleek ellipsoid |
| body_l | Maximum visible length between the tip of the head and the fluke with values ranging from calf size to maximum adult length. | Meters | Numbers | *e.g.* 12.4 |
| body_w | Body width, it is measured at the widest part of the body and perpendicular the body length. | Meters | Numbers | *e.g.* 2.0 |
| flipper | Forelimb used to stabilise and turn.  1 = Yes  2 = No  3 = Maybe | None | 1 to 3 | *e.g.* 1 |
| lg_flipper | Species specific – Humpback whale have long flippers, which are one third of the body length.  1 = Yes  2 = No  3 = Maybe | None | 1 to 3 | *e.g.* 1 |
| fluke | Tail used to generate thrust.  1 = Yes  2 = No  3 = Maybe | None | 1 to 3 | *e.g.* 1 |
| head_callo | Species specific – white head callosities for the species of the genus Eubalaena. White patches on top of the head.  1 = Yes  2 = No  3 = Maybe | None | 1 to 3 | *e.g.* 1 |
| wh_lr_jaw | Species specific – white right lower jaw for fin whales.  1 = Yes  2 = No  3 = Maybe | None | 1 to 3 | *e.g.* 1 |
| aft_breach | After breach, large white area left after a whale breached, or lobtailed, flipper-slapped.  1 = Yes  2 = No  3 = Maybe | None | 1 to 3 | *e.g.* 1 |
| bubble_net | Species specific – bubble net for humpback whales. One white spiral formed of several white circular patches, or several white spirals nested together.  1 = Yes  2 = No  3 = Maybe | None | 1 to 3 | *e.g.* 1 |
| contour | White line separating the part of the whale body that is above and below the sea surface (*e.g.*, when a whale is rolling its back or surfacing to breathe).  1 = Yes  2 = No  3 = Maybe | None | 1 to 3 | *e.g.* 1 |
| flukeprint | White circle left after whale dove or while swimming (Levy *et al.*, 2011).  1 = Yes  2 = No  3 = Maybe | None | 1 to 3 | *e.g.* 1 |
| wake | V-shaped white trail behind the animal.  1 = Yes  2 = No  3 = Maybe | None | 1 to 3 | *e.g.* 1 |
| blow | Vaporous whitish patch next to a whale, like fog.  1 = Yes  2 = No  3 = Maybe | None | 1 to 3 | *e.g.* 1 |
| mudtrail | Plume/cloud of substrate behind a whale.  1 = Yes  2 = No  3 = Maybe | None | 1 to 3 | *e.g.* 1 |
| sur_act_gr | Two or more whales rolling and touching at the surface.  1 = Yes  2 = No  3 = Maybe | None | 1 to 3 | *e.g.* 1 |
| travel_gr | Two or more cetaceans traveling together in the same direction and less than a few meters apart.  1 = Yes  2 = No  3 = Maybe | None | 1 to 3 | *e.g.* 1 |
| mc_pair | Mother-calf pair, observed when the calf next to the mother.  1 = Yes  2 = No  3 = Maybe | None | 1 to 3 | *e.g.* 1 |
| otr_gr | Other type of group, if not socialising or traveling.  1 = Yes  2 = No  3 = Maybe | None | 1 to 3 | *e.g.* 1 |
| defecation | 1 = Yes  2 = No  3 = Maybe | None | 1 to 3 | *e.g.* 1 |
| comment | Any other comment the observer would like to make about the specific detection | None | Letters | *e.g.* mother of the mother-calf pair |

# Joining annotations from multiple observers

We recommend that at least two observers should review the same satellite image(s) independently. Once each observer has annotated the images, the point shapefiles can be used to join the annotations.

# Creating bounding box

Here we are following the method from Cubaynes and Fretwell, 2022 adapted for ArcGIS Pro.

## Centre the points in a middle of a pixel

1. Use “Extract by Mask” tool to select the pixel the point is on. You can access this tool by selecting “Tools”, under the “Analysis” tab.
   1. Under the “Geoprocessing” window, type “extract by mask” and select the top option “Extract by Mask (Spatial Analyst Tools)”.


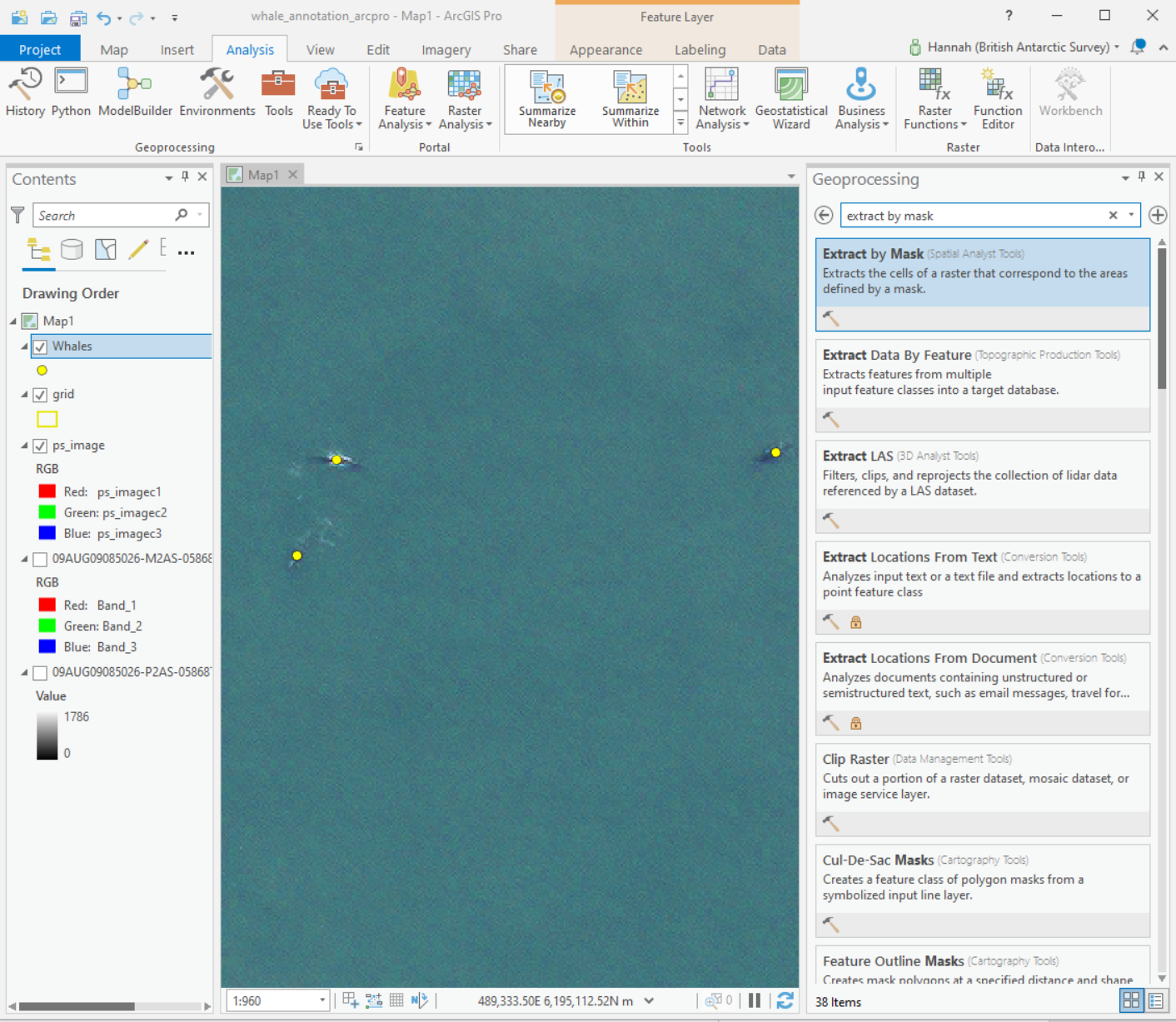


- 1. In the “Extract by Mask” window, fill in the following information, then select “Run”:

Input raster: the panchromatic file

Input raster or feature mask data: the point shapefiles with the joined whale annotations

Output raster: leave as it is, *i.e.* going to your default ArcGIS folder


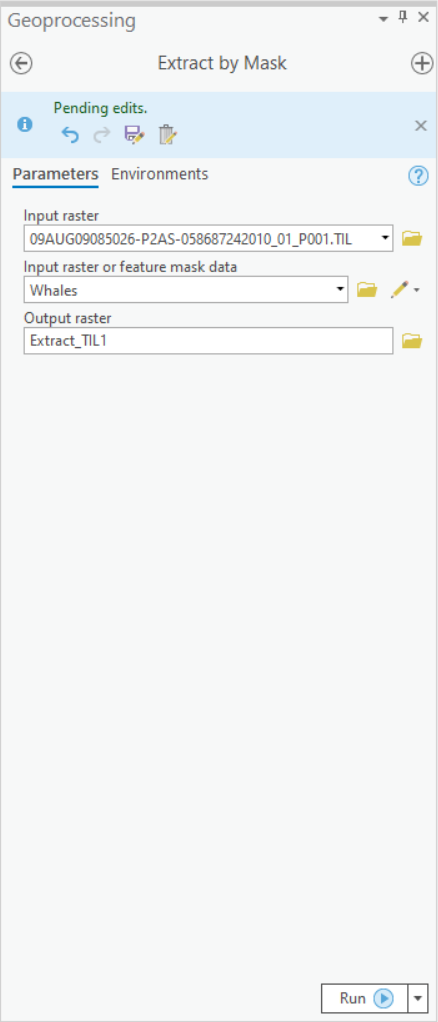


- 1. A new file will appear under the “Contents” window.


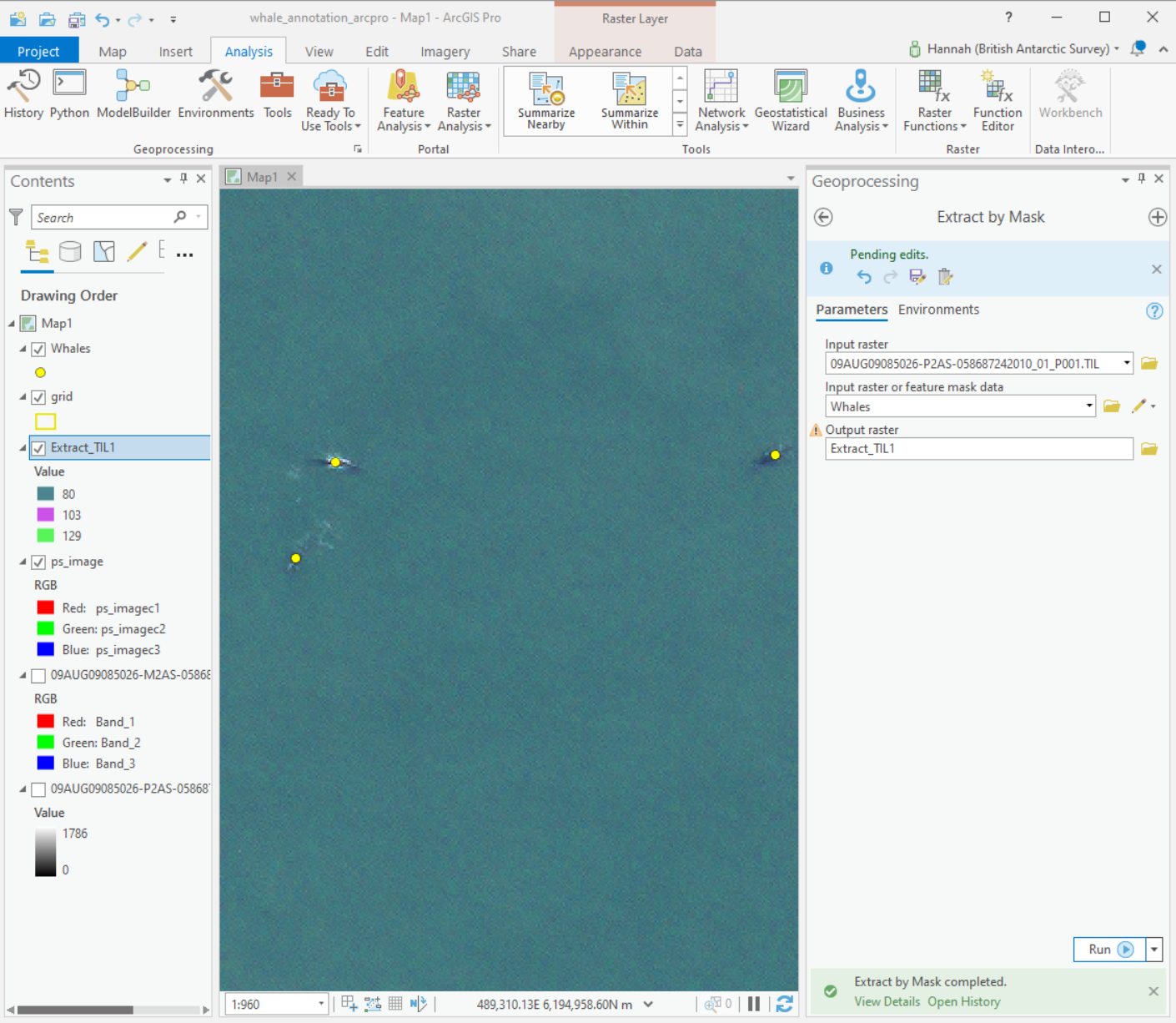


1. Use the “Raster to Point” tool to create a point centered on the pixel we have just extracted. You can access this tool by selecting “Tools”, under the “Analysis” tab, similar to step 22.
   1. Under the “Geoprocessing” window, type “raster to point” and select the top option “Raster to Point (Conversion Tools)”.


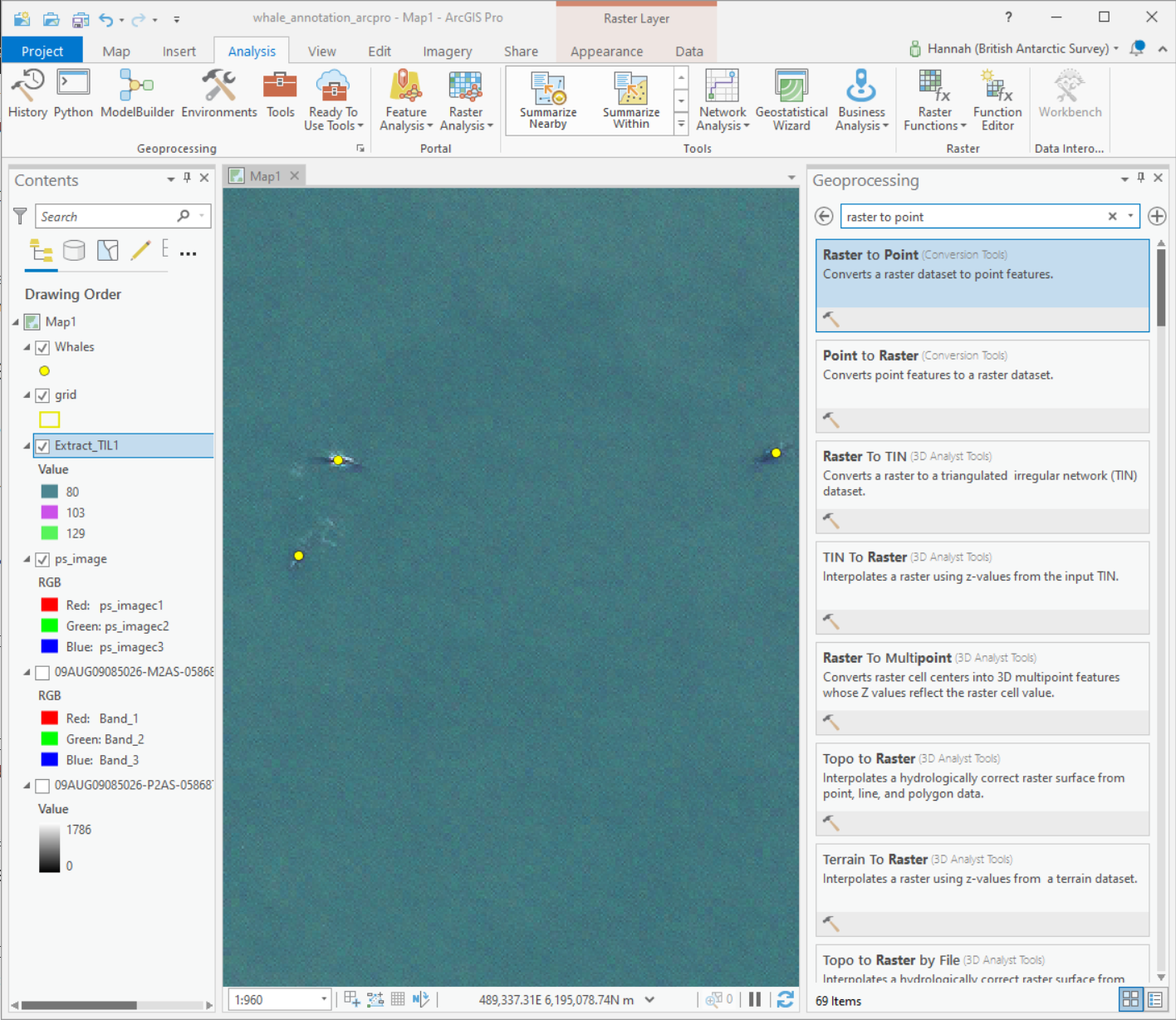


- 1. Under the “Raster to Point” window, fill in the following information and then select “Run”:

Input raster: the file created in step 22.2.

Field: Value

Output: leave as it is


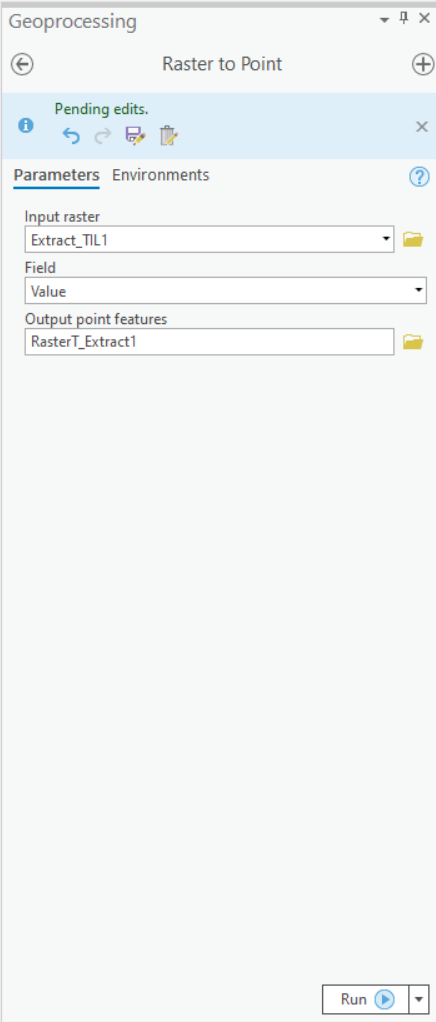


- 1. The newly created file will appear under the “Contents” window.


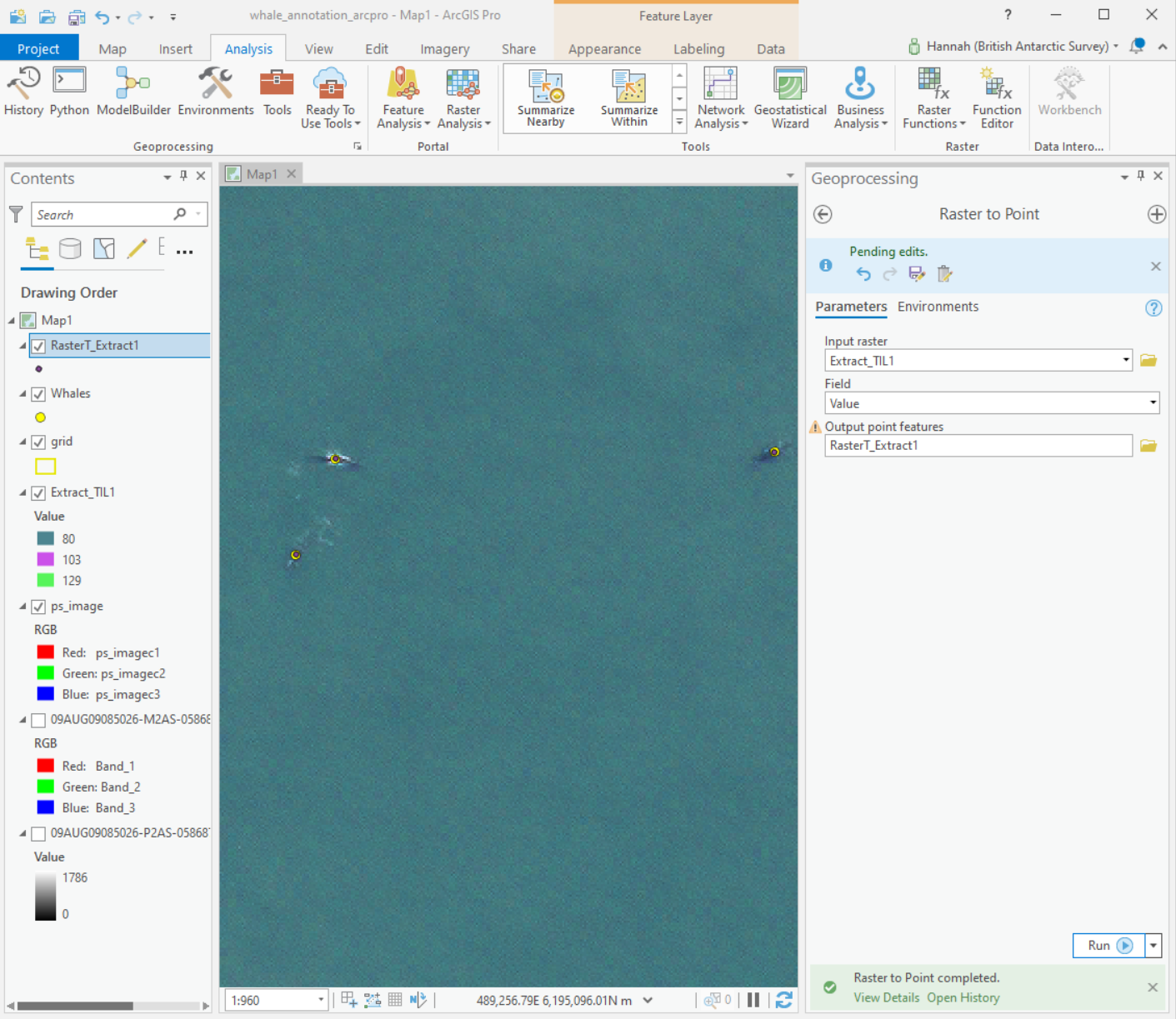


1. Use the “Spatial Join” tool, to attach the attribute table of the original point shapefile with the whale annotation to the newly created shapefile with centered points. You can access this tool by selecting “Tools”, under the “Analysis” tab, similar to step 22.
   1. Under the “Geoprocessing” window, type “spatial join” and select the “Spatial Join (Analysis)”.


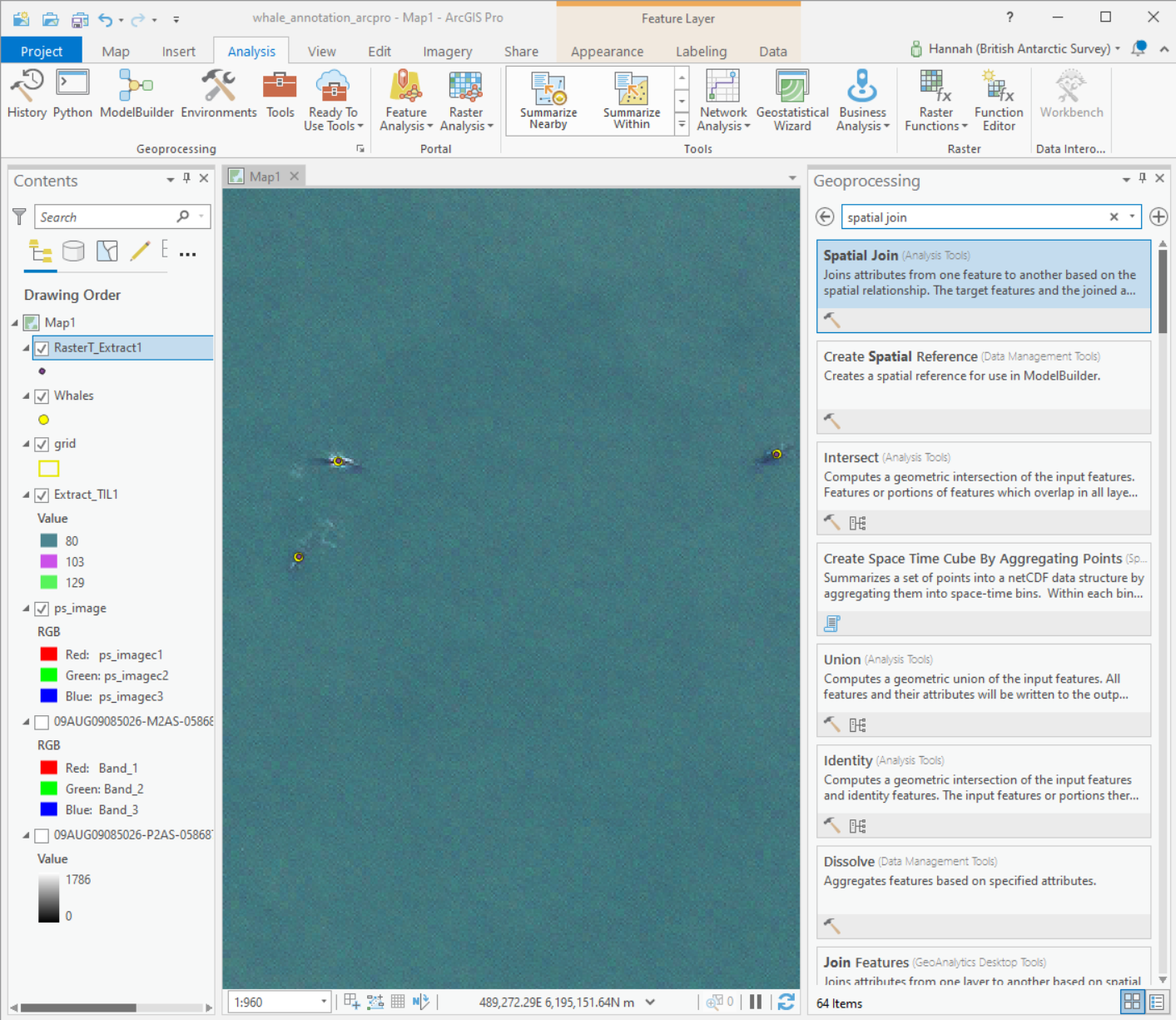


- 1. Under the “Spatial Join” window, fill in the following information, then select “Run”:

Target Features: the centered point shapefile created step 23.2.

Join Features: the point shapefile with the original whale annotation (uncentered).

Output Features Class: choose where to save it and name the file to be created.

Join Operation: Join one to many

Keep All Target Features: tick

Match Option: Closest

Search Radius: 0.5 Meters (here we are using a GeoEye-1 image which has a spatial resolution of 0.5m.


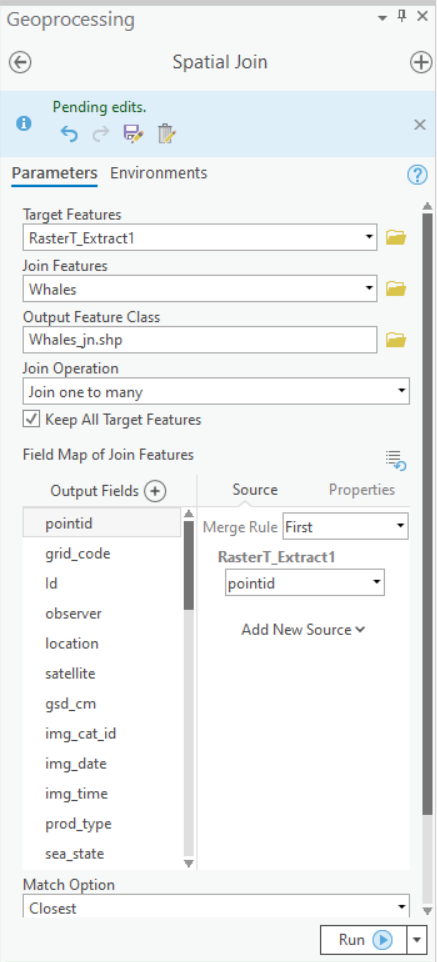

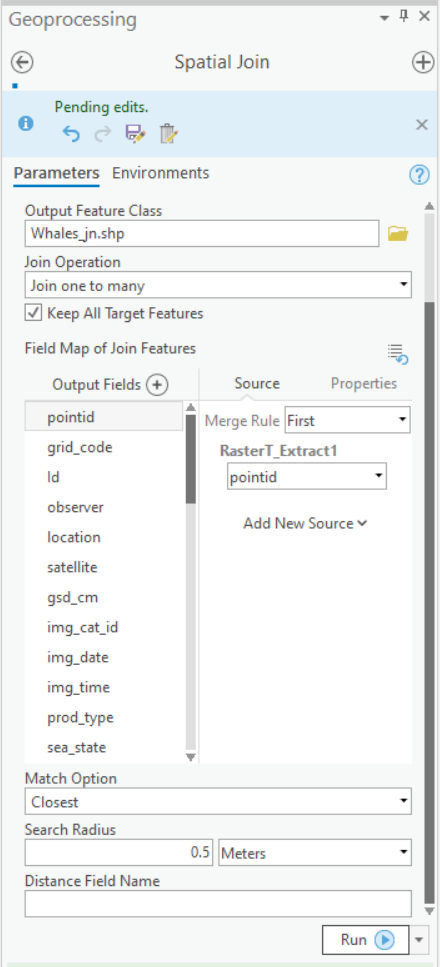


- 1. The newly created shapefile will appear in the “Contents” window.


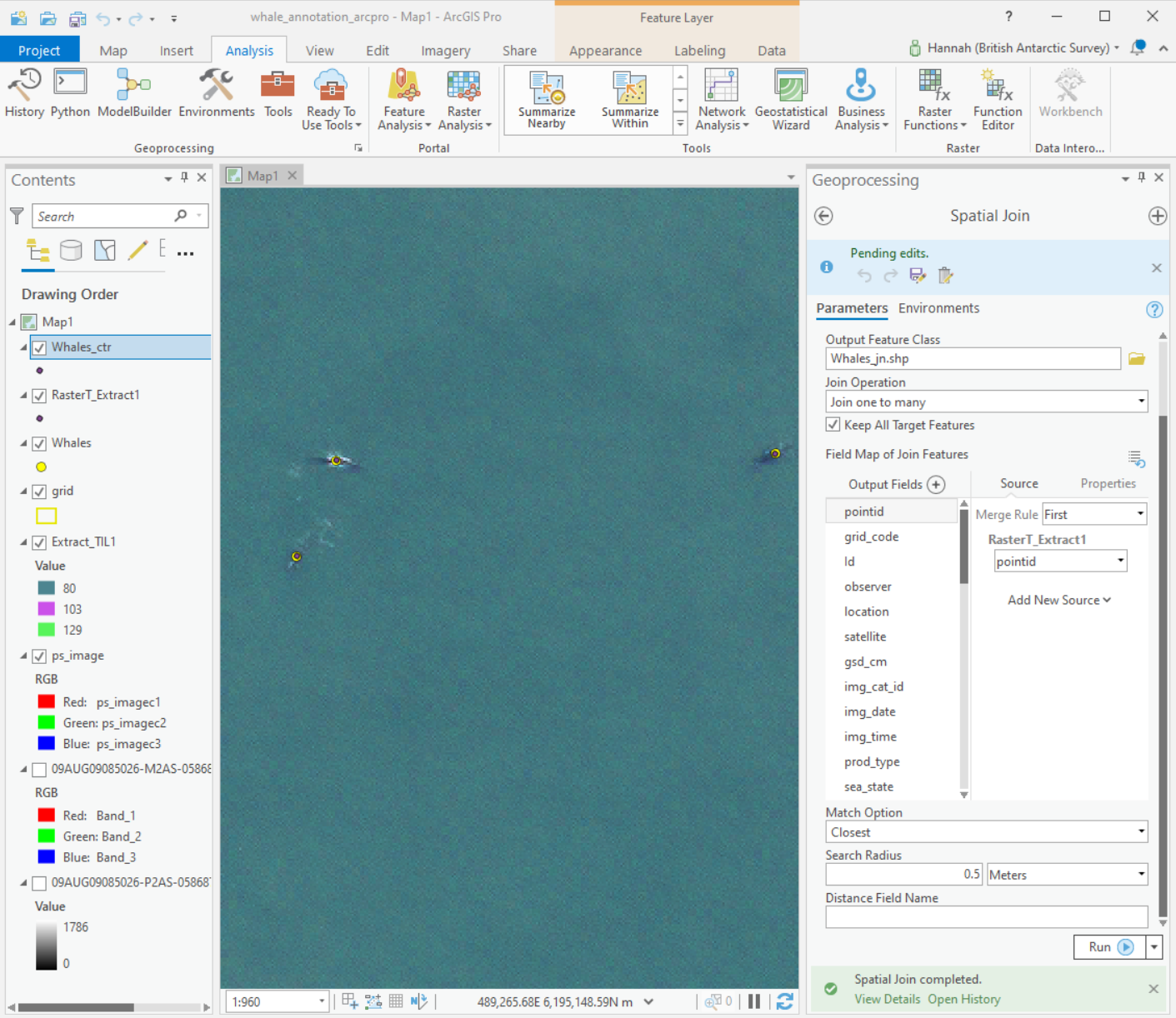


## Create a buffer around the centered points

1. Create a buffer around the centered points, by using the “Buffer” tool. You can access this tool by selecting “Tools”, under the “Analysis” tab, similar to step 22.
   1. Under the “Geoprocessing” window, type “buffer” and select the “Buffer (Analysis)”.


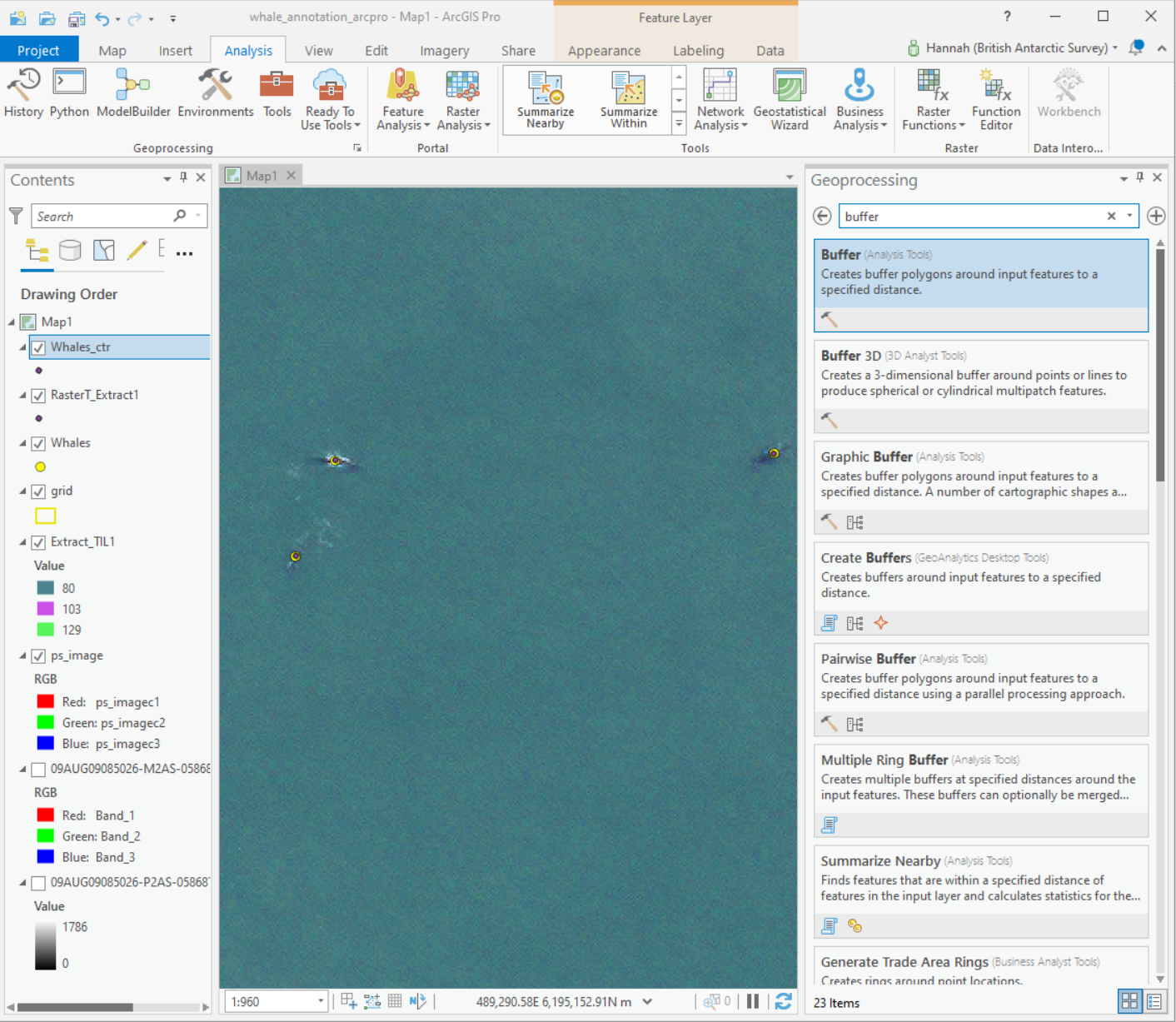


- 1. Under the “Buffer” window, fill the following information, then select “Run”:

Input Features: the point shapefile with the centered points and adjoining attribute table created in step 24.2.

Output feature class: choose the location to save, and rename the file that is being created at this step.

Distance: keep linear unit and inform “32 Meters” for a GeoEye-1 image, you can reduce or increase this size. A 32 buffer means that the whale point should be fully included within the buffer. For the bounding boxes to be useful to train machine learning systems, it is necessary that these boxes cover an even number of pixels and preferably a power of two, so we chose for our boxes to be 128 by 128 pixels. The width (or height of the bounding boxes, here 128 pixels) divided by the spatial resolution (50cm here), gives a diameter for the buffer of 64 m and a radius of 32 m.

Leave the rest as default.


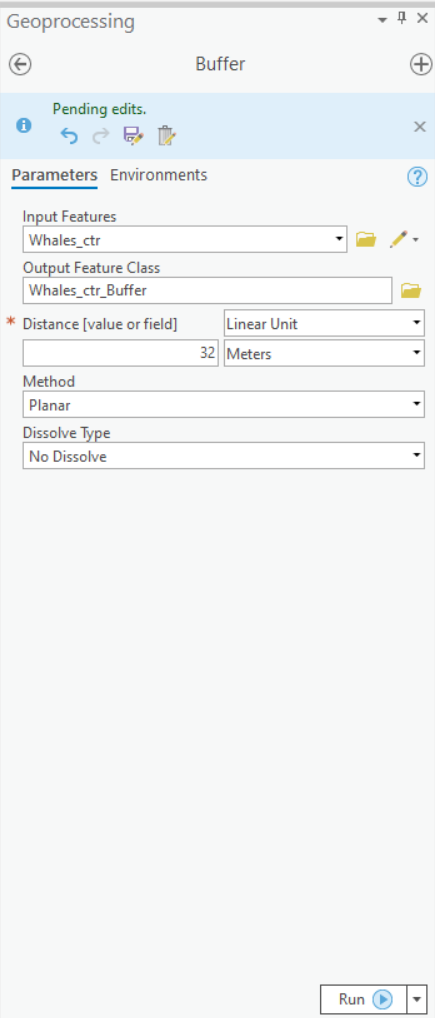


- 1. The buffer shapefile will appear in the “Contents” window, and you can visualize the buffers in the “Map” window.


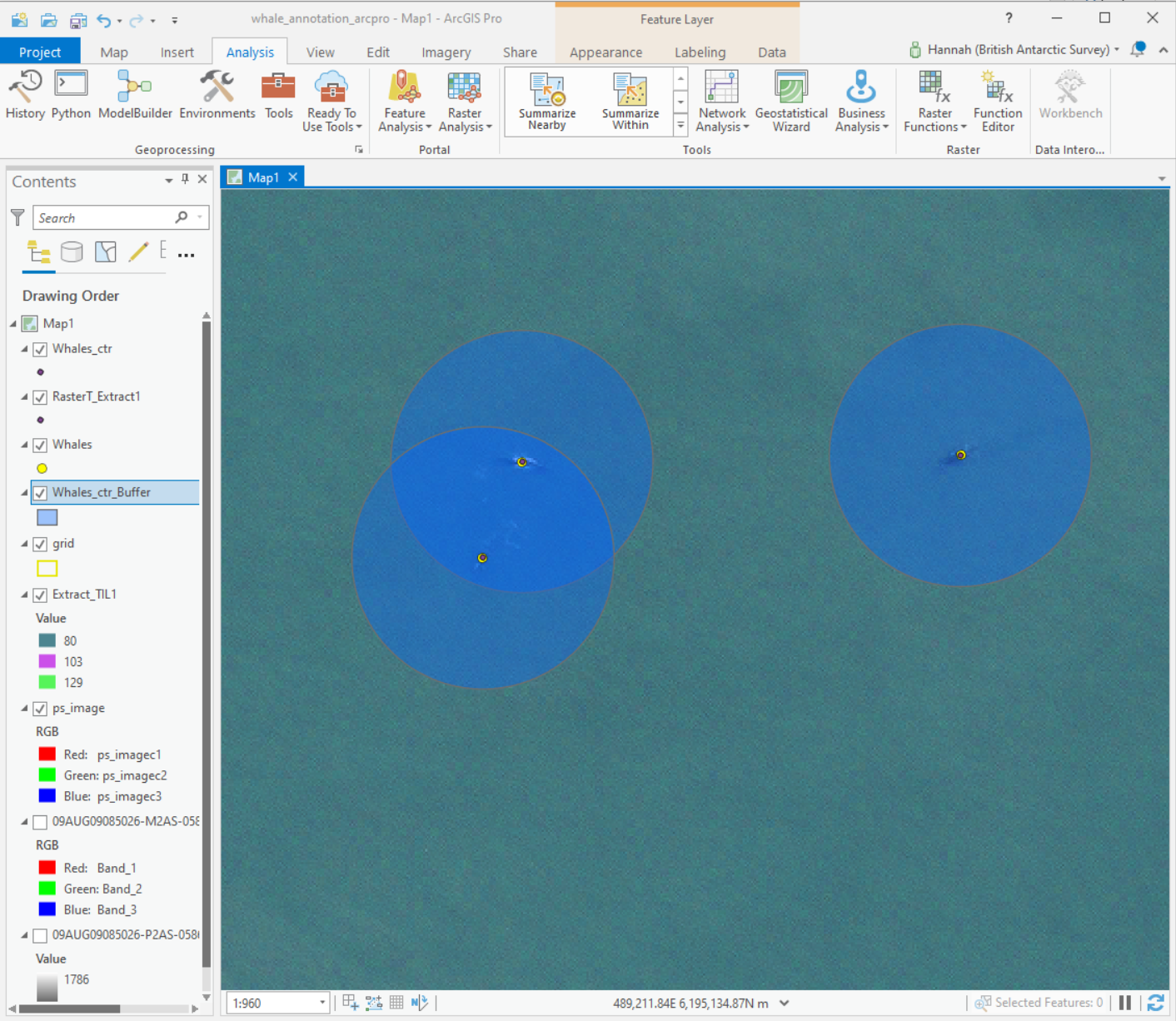


1. The buffers need to be shifted so they align with the border of the pixels at the top, bottom, left and right.
   1. Select all the buffers.


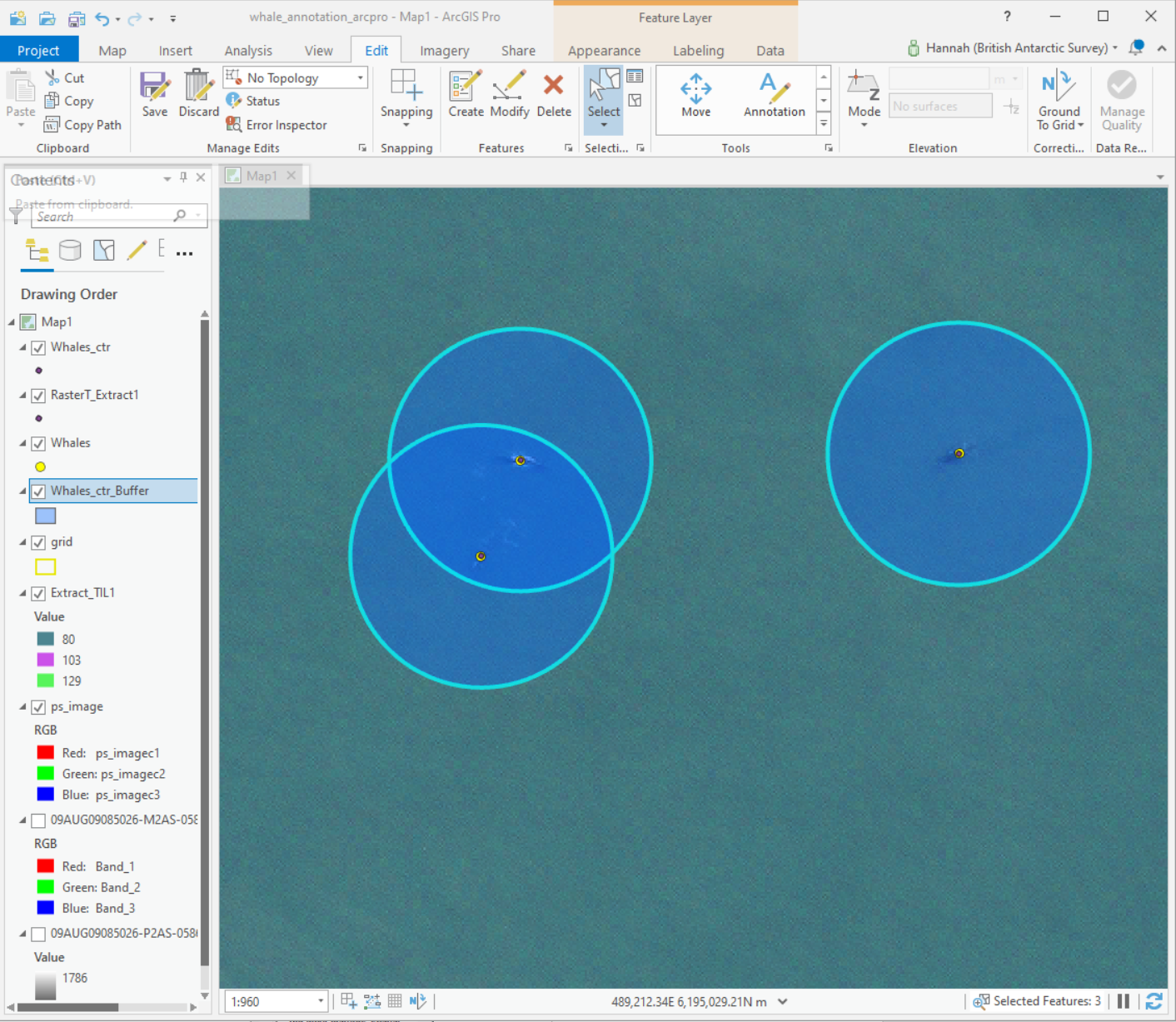


- 1. Under the “Edit” tab, select the “Move to” icon. You may have to scroll to reach this tool using the arrows on the right.


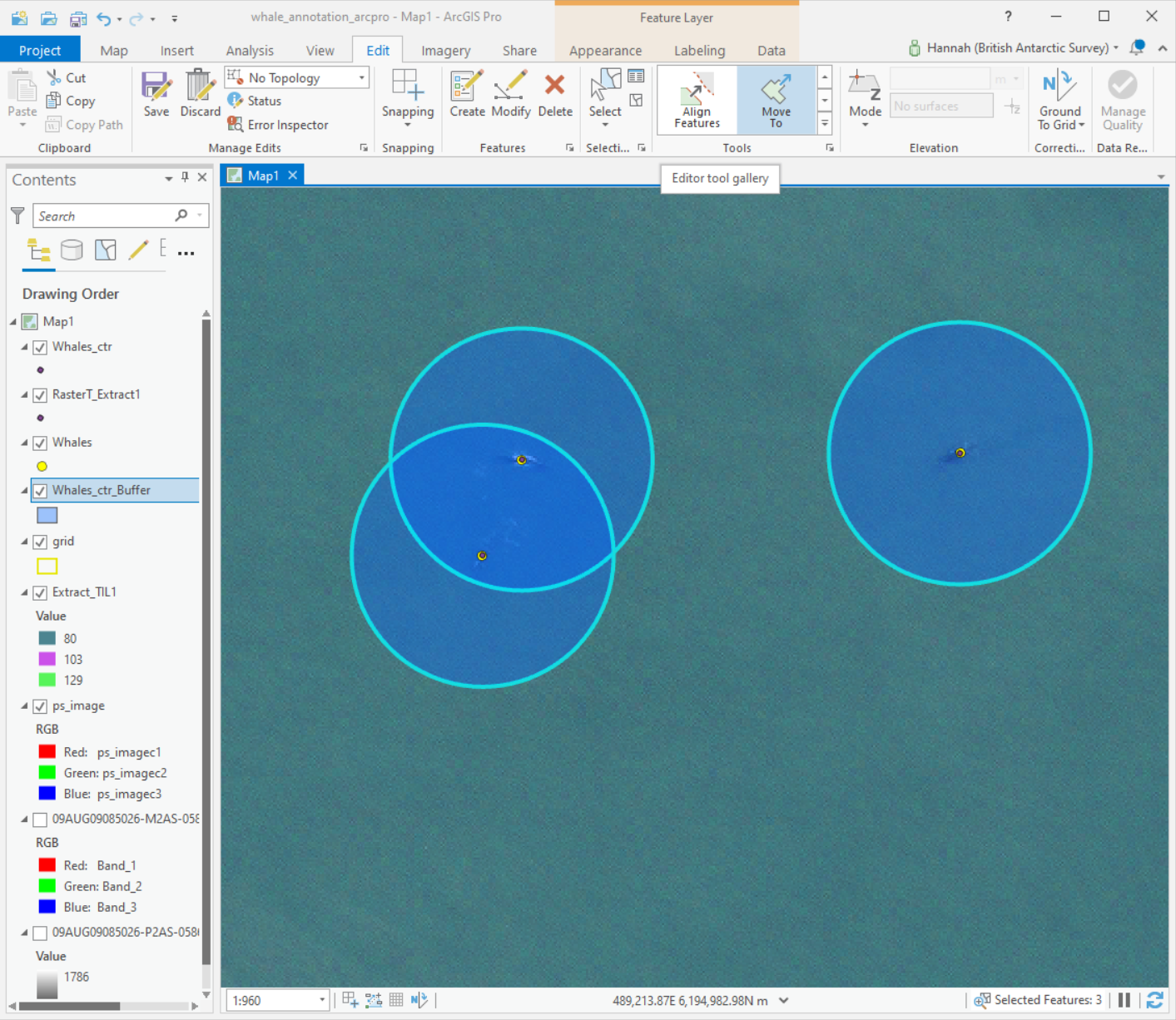


- 1. Under the “Move to” window, fill in the following value for a GeoEye-1 image if centering on the pansharpened image: 0.25 (i.e. the spatial resolution divided by 2). Then select “Move to”.


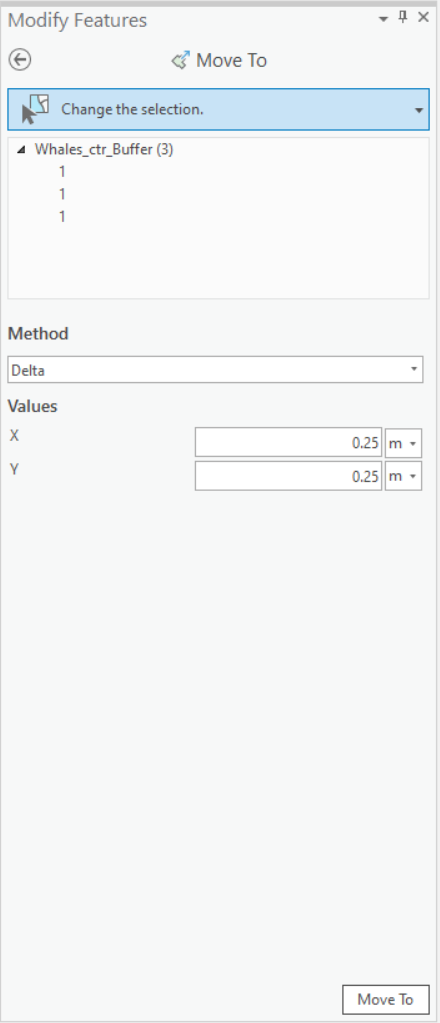


- 1. Remember to save your edits by selecting “Save” under the “Edit” tab.


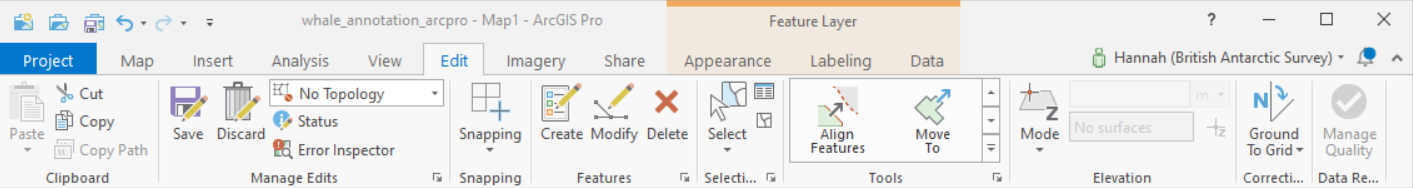


## Create a bounding box around the buffer

1. To create a box encompassing the buffer (bounding box), use the “Feature Envelope to Polygon” tool. You can access this tool by selecting “Tools”, under the “Analysis” tab, similar to step 22.
   1. Under the “Geoprocessing” window, type “envelope” and select the “Feature Envelope to Polygon (Data Management)”.


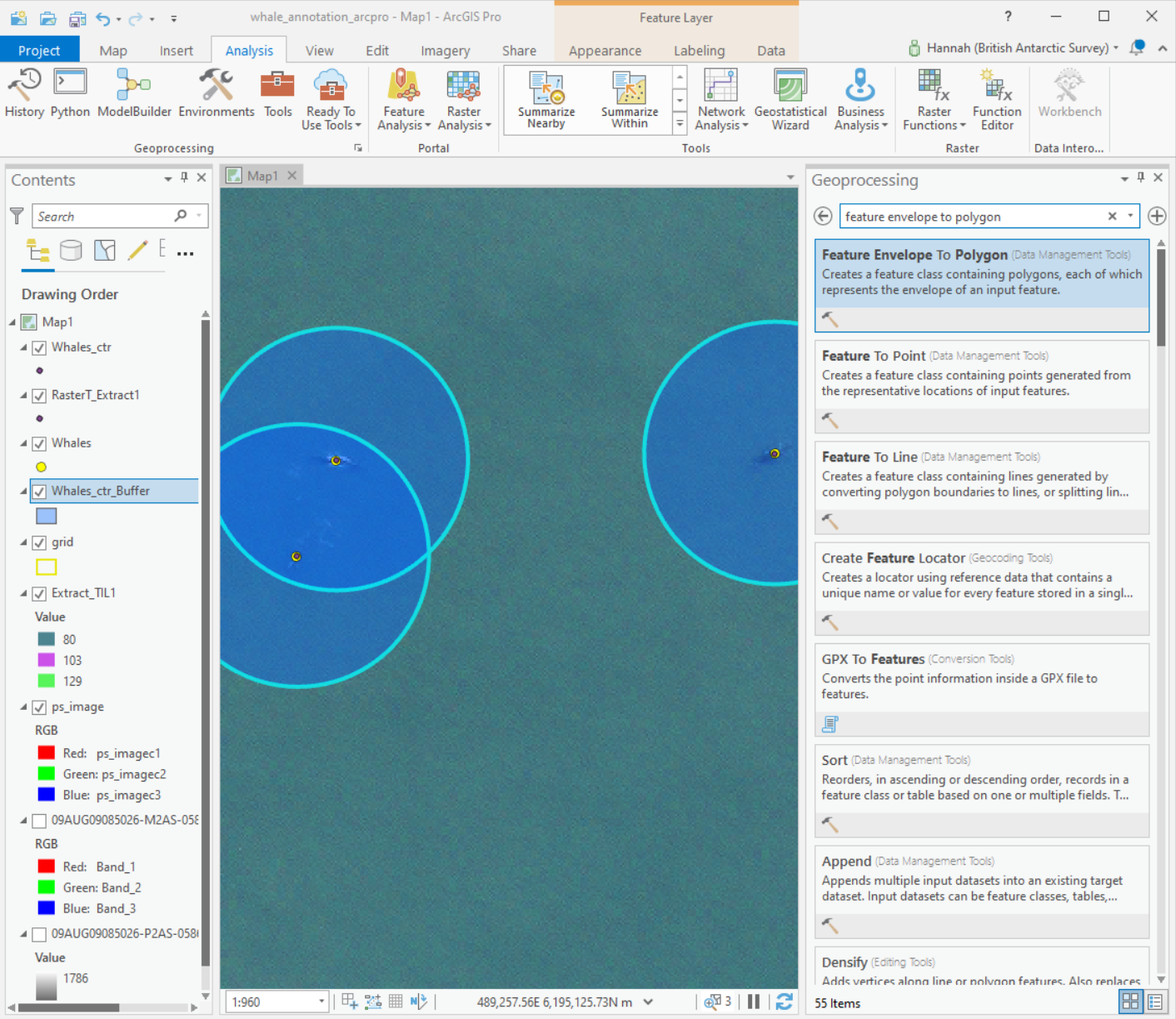


- 1. Under the “Feature to Envelope” window, fill in the following information, then select “Run”:

Input Features: the buffer file created at step 26.3

Output Feature Class: choose where to save the file to be created and name the file

Create multipart features: tick


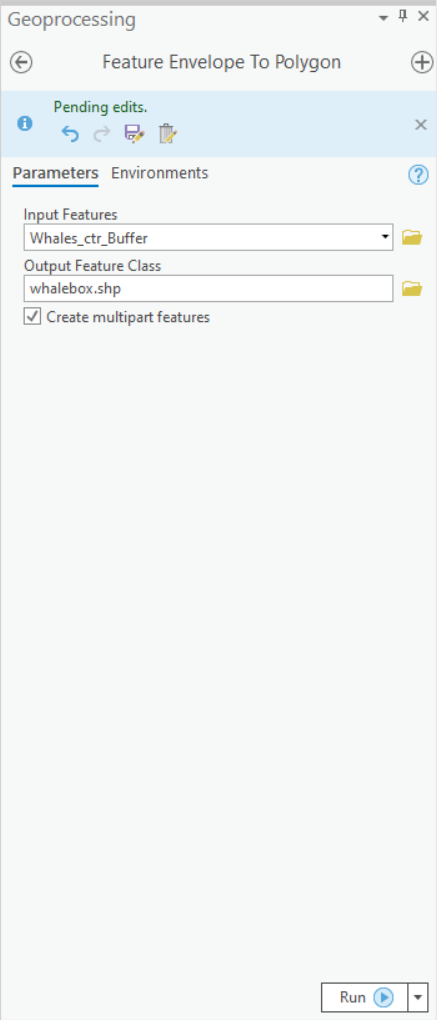


- 1. The bounding box will now appear in the “Contents” window and be visible in the “Map” window.


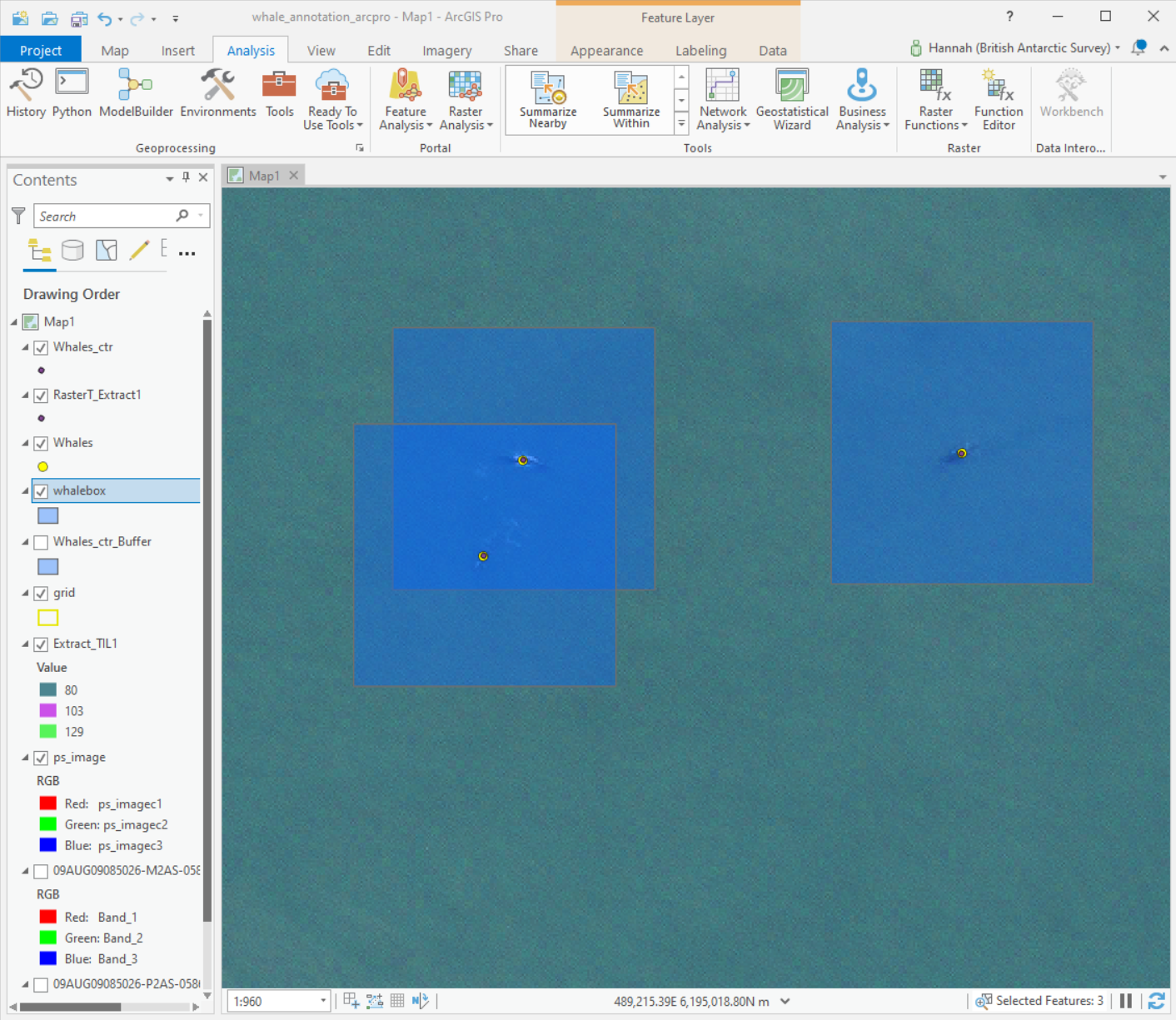


- 1. We recommend adding the following fields to the attribute table of the bounding box shapefile. Follow steps 13.1 to 13.4 to create new fields and add the fields listed in Table 4.

**Table 4.** Information necessary to fill the “Fields” window, each row represents a different Field.

| **Name** | **Type** | **Field Properties** | **Description** |
| --- | --- | --- | --- |
| num_whale | Short integer | Precision: 0 | The number of whales within the bounding box. Sometimes two whales swimming nearby will be in each other’s bounding box. Even if only a portion of the whale is inside the box, it counts as one whale. |
| box_id | Text | Length: 50 | A unique ID for each bounding box. |
| box_size | Text | Length: 15 | The size of the bounding box, e.g. 128x128pixels |

- 1. Fill in the attribute table following the steps from steps 19 to 21. To fill in some information quicker, remember you can use the “Calculate Field” tool (see step 20) using the information below.

TIP: to fill in the information of box_id quicker, especially if you have more than 10 boxes, you can enter the following in the “Field Calculator”: To get SRW_GoeEye-1_20170221_B1: write [sp_code]&"_"& [satellite]&"_"&"_"& [img_date]&"_"&"B"& [FID]

WARNING: ensure there is no space, otherwise it will not work.

# Creating image chips (PNG)

Due to licensing restrictions, the image chips cannot currently be shared in a .tif format; therefore, below we show how to export them in a .png format. If you wish to share your image chips, similar to Cubaynes and Fretwell, 2022, check the conditions with the satellite imagery provider, and the end user license agreement.

## Clipping the satellite image to the outline of the bounding boxes

1. Select all the bounding boxes by zooming out to see the whole image and using the “Select” tool, that can be found under the “Map” tab. Then click on the top left corner of the satellite image and drag to encompass all boxes.


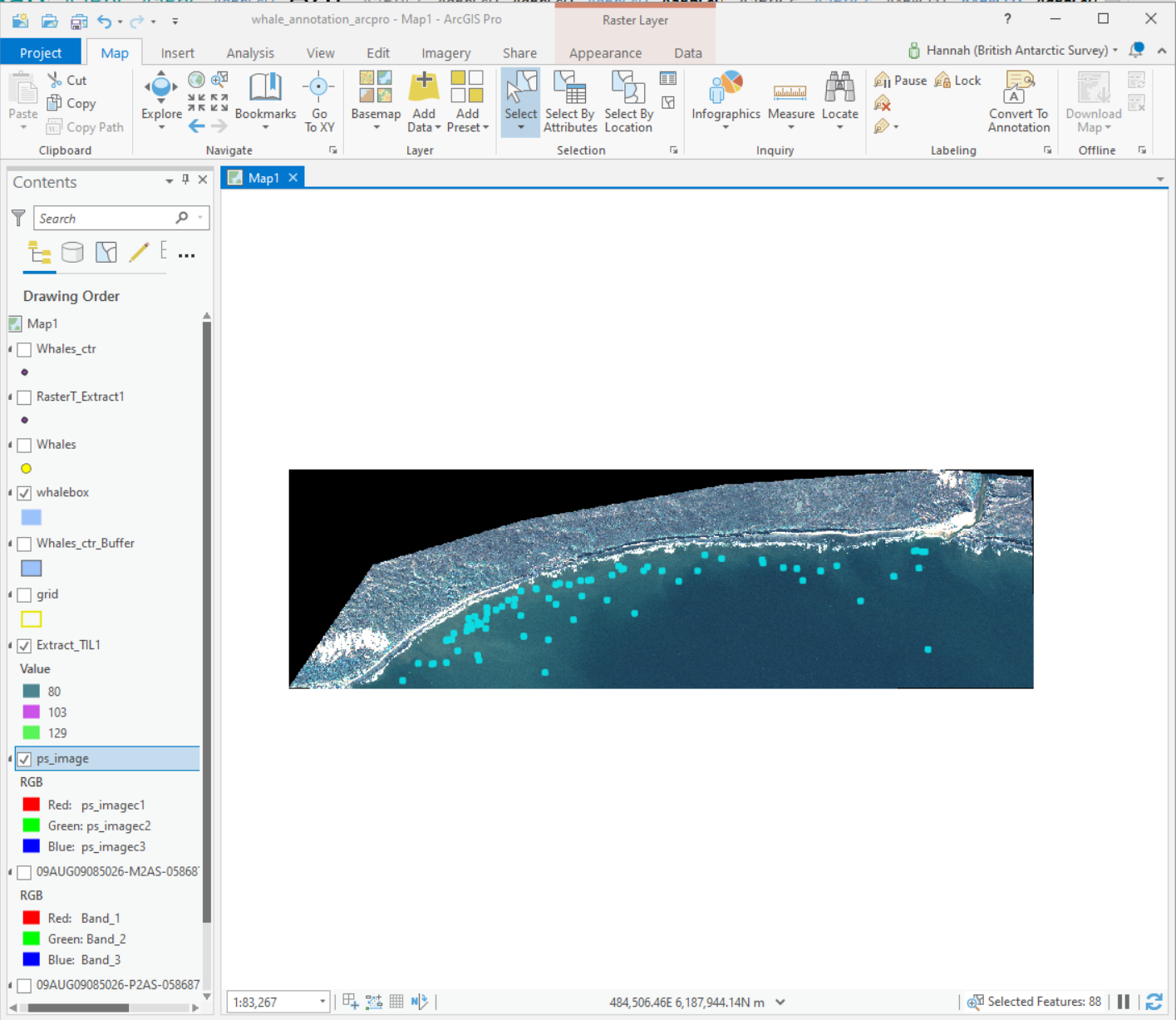


1. Under the “Analysis” tab, select “Raster Functions”.


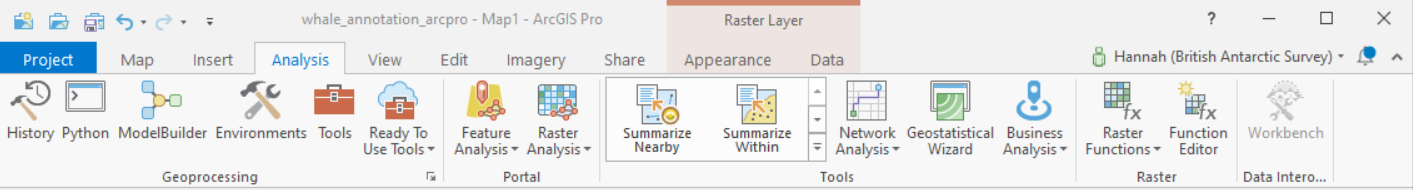


- 1. In the “Raster Function” window, search for “clip” and select the “Clip” tool that can also be found under “Data Management”.

WARNING: make sure that the full extent of the satellite image is visible, as the clip tool only applies to what is visible in the “Map” window.


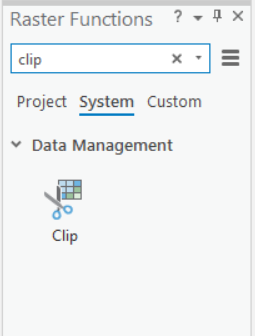


- 1. In the “Clip Properties” window, fill in the following information, then select “Create new layer”:

Raster: the pansharpened file

Clipping Type: Outside

Clipping Geometry / Raster: the shapefile with the whale bounding boxes

Use input features for clipping geometry: tick


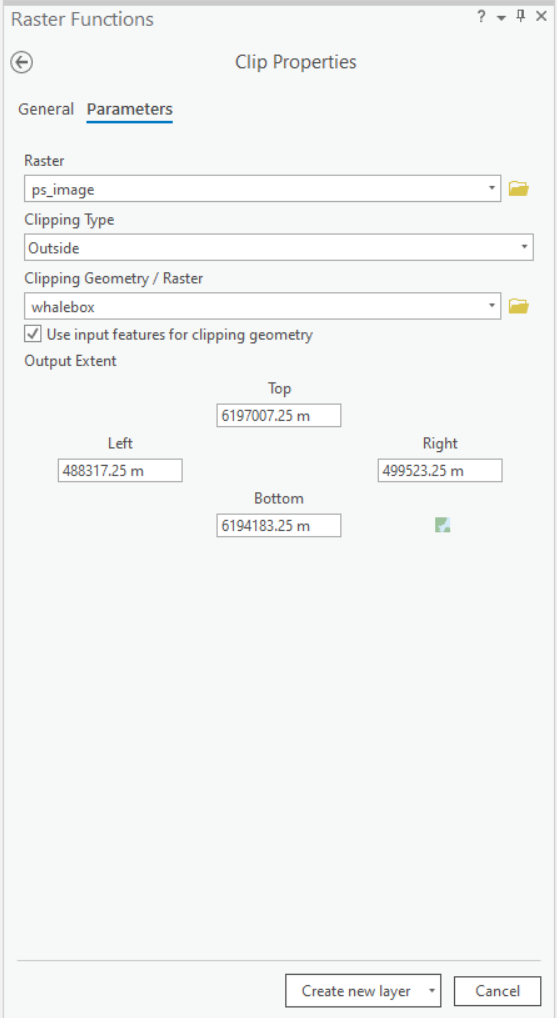


## Exporting the clipped tif file as a png file

1. Right click on the clipped raster file, select “Data”, then select “Export Raster”.


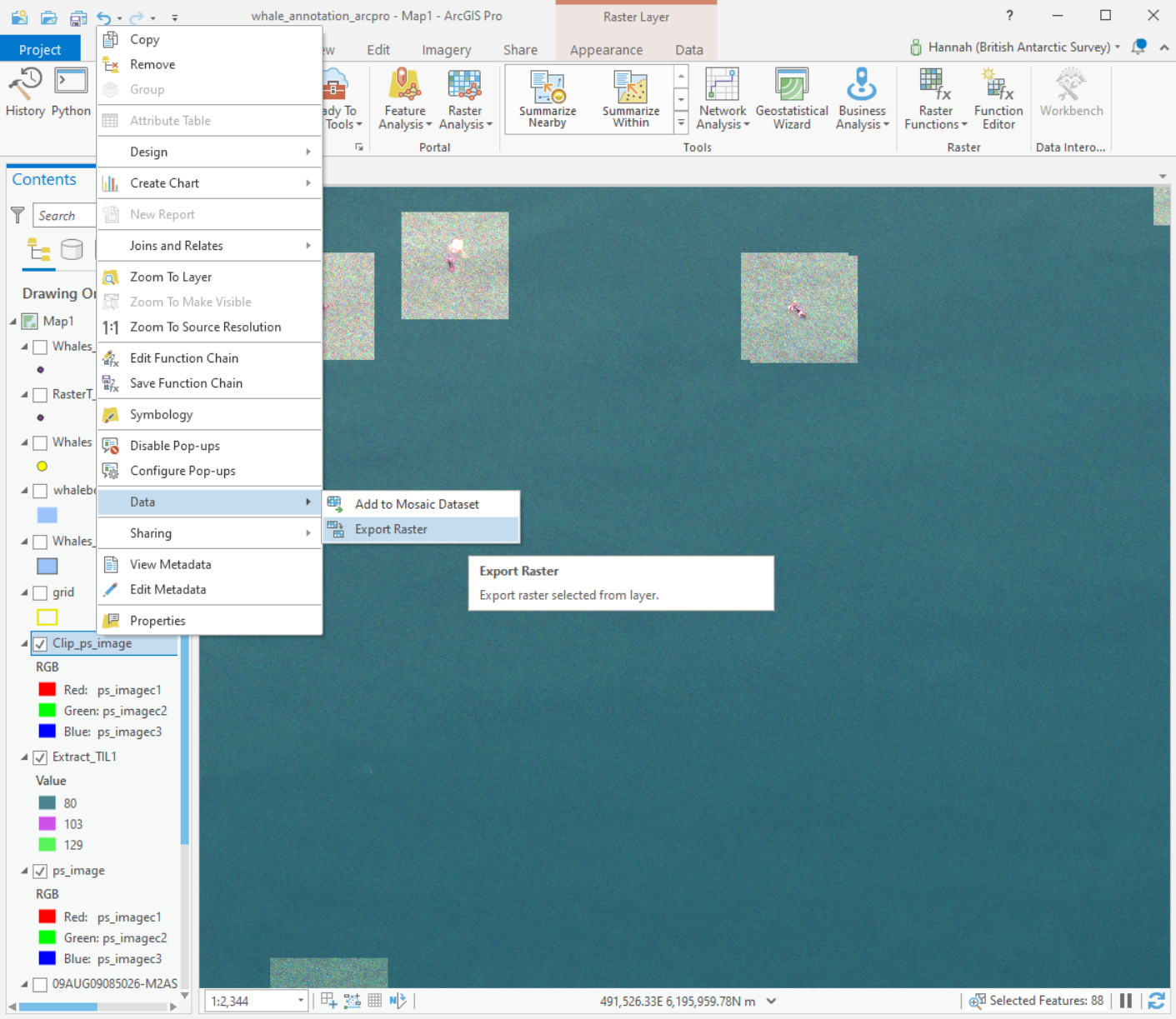


1. Fill in the newly opened “Export Raster” window as follow, then select “Export”:

Output Raster Dataset: the clipped raster file

Leave the “Extent” and “Spatial Reference” as they are

Select “Use Renderer, and ensure “Force RGB” is selected too

Pixel Type: 16 bit unsigned (check the properties of the clipped raster created in steps 28 to 29)

Output Format: PNG

Name: choose the name for the png file to be created

No Data: 0 (as otherwise the output of the next step, using the split tool will be black image chips)


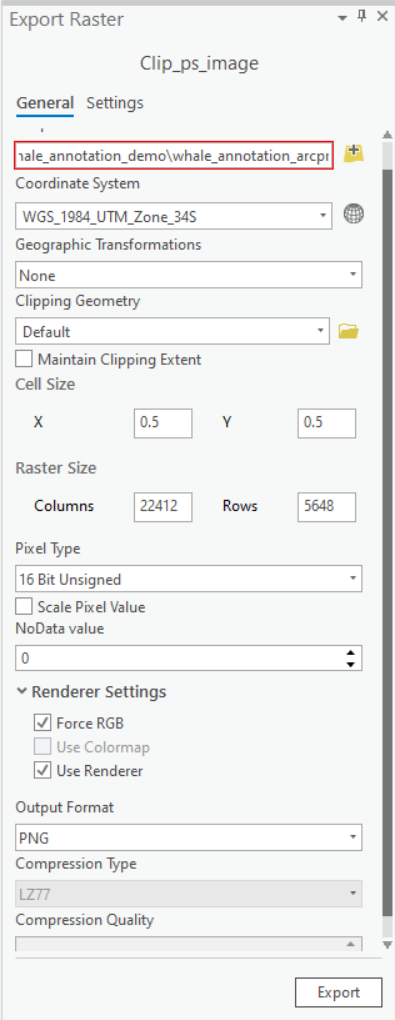


## Create multiple image chips

1. Use the “Split Raster” tool to split the png image into several image chips. You can access this tool by selecting “Tools”, under the “Analysis” tab, similar to step 22.
   1. Under the “Geoprocessing” window, type “split raster”, then select “Split Raster (Data Management Tools)”.


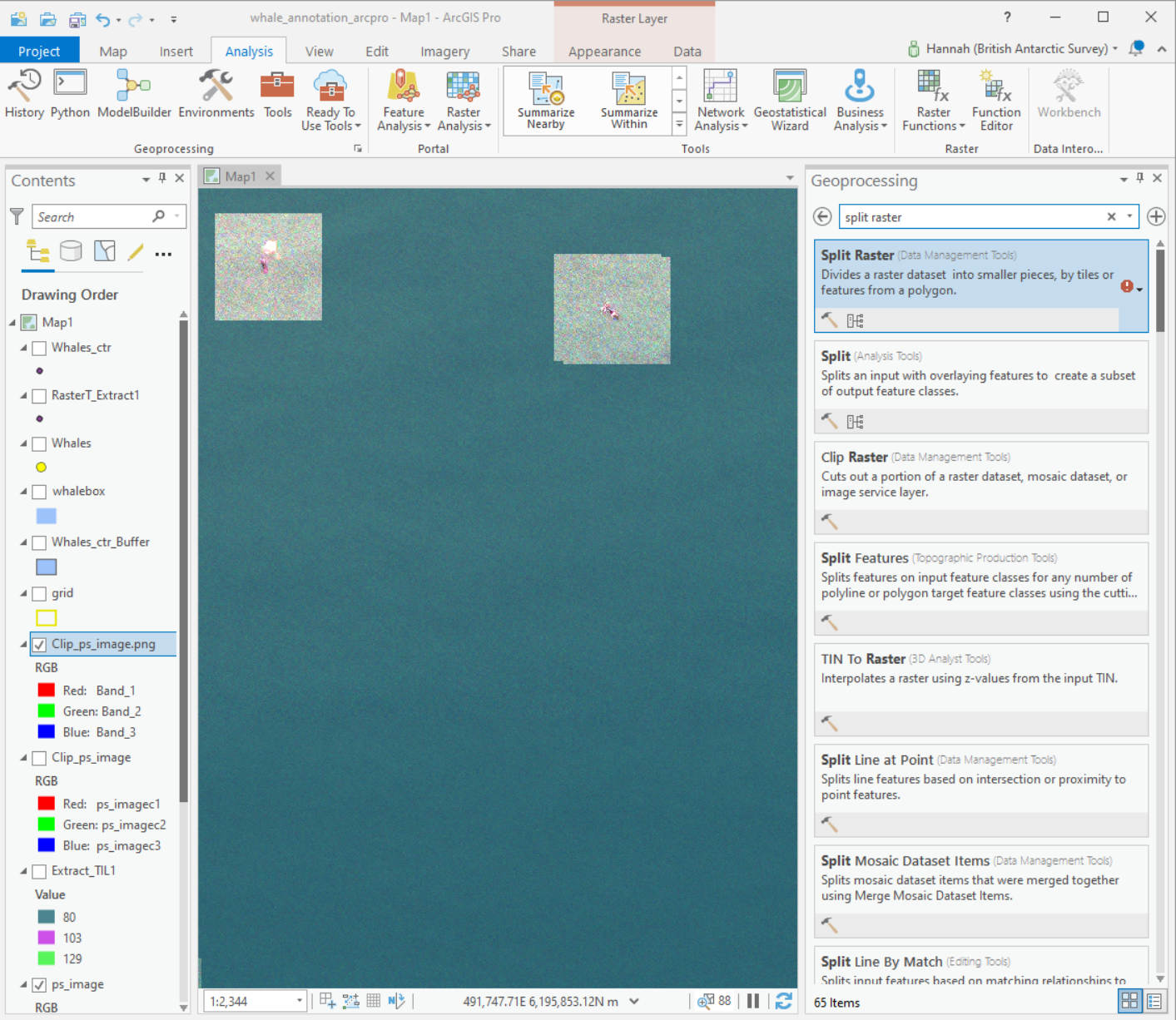


- 1. Under the “Split Raster” window, fill in the following information, then select “Run”:

Input Raster: the png image chips created at step 31.

Output folder: choose the location where you wish to save the several image chips.

Output Basename: we recommend using the same as you entered in the box_id (minus the FID number).

Split Method: Polygon features

Output Format: PNG

Resampling method: Nearest

Split Polygon Feature Class: select the bounding box shapefile you created in step 27.2.

Other Options> Overlap: 1000

Other Options> Units of Output Raster Size and Overlap: PIXELS


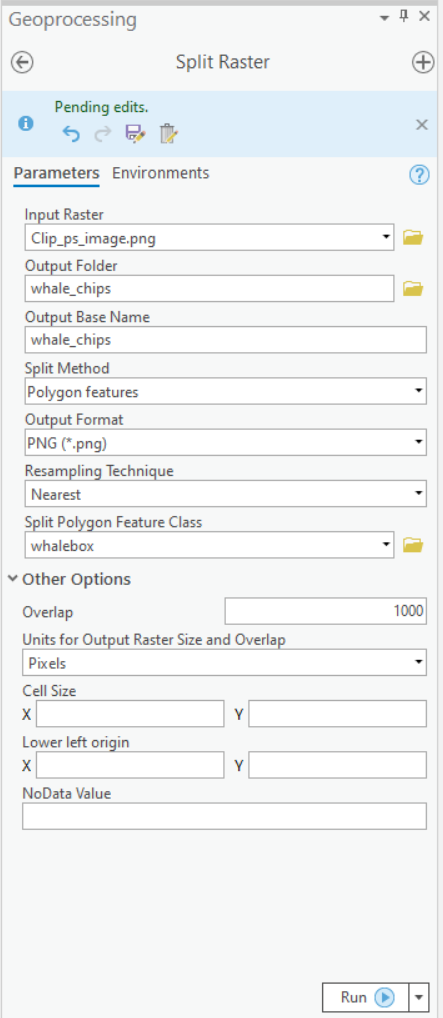


# Acknowledgments

This research was possible thanks to the Marine Mammal Commission for their financial support of the project MMC21-043. This study represents a contribution of the Ecosystems component of the British Antarctic Survey, funded by the Natural Environment Research council (NERC). The aerial images of cetaceans were collecting under the following permits: MMPA 17355, MMPA 21371, MMPA 775-1875, MMPA 775-1600.

# References

Airbus (2022) ‘Pleiades imagery user guide’, p. 114. Available at: https://www.intelligence-airbusds.com/en/8718-user-guides.

Cubaynes, H. C. *et al.* (2019) ‘Whales from space: Four mysticete species described using new VHR satellite imagery’, *Marine Mammal Science*, 35(2), pp. 466–491. doi: 10.1111/mms.12544.

Cubaynes, H. C. and Fretwell, P. T. (2022) ‘Whales from space dataset, an annotated satellite image dataset of whales for training machine learning models’, *Scientific Data*, 9, p. 245. doi: 10.1038/s41597-022-01377-4.

Jefferson, T. A., Webber, M. A. and Pitman, R. L. (2008) ‘Marine Mammals of the World: A Comprehensive Guide to their Identification’, *Aquatic Mammals*, 35(3), pp. 414–415. doi: 10.1578/AM.35.3.2009.414.

Larrat, S. and Lair, S. (2022) ‘Body condition index in beluga whale (Delphinapterus leucas) carcasses derived from morphometric measurements’, *Marine Mammal Science*, 38, pp. 274–287. doi: 10.1111/mms.12855.

Levy, R. *et al.* (2011) ‘A theory for the hydrodynamic origin of whale flukeprints’, *International Journal of Non-Linear Mechanics*, 46(4), pp. 616–626. doi: 10.1016/j.ijnonlinmec.2010.12.009.

Maxar Technologies (2022) *Core imagery product information*. Available at: https://securewatchdocs.maxar.com/en-us/Orders/Orders_ProductInfo.htm#CoreImageryProductInformation.

Planet (2022) ‘Planet imagery product specifications’, p. 101. Available at: https://assets.planet.com/docs/Planet_Combined_Imagery_Product_Specs_letter_screen.pdf.

Williams, T. M., Noren, S. R. and Glenn, M. (2011) ‘Extreme physiological adaptations as predictors of climate-change sensitivity in the narwhal, Monodon monoceros’, *Marine Mammal Science*, 27(2), pp. 334–349. doi: 10.1111/j.1748-7692.2010.00408.x.

Woodward, B. L., Winn, J. P. and Fish, F. E. (2006) ‘Morphological specializations of baleen whales associated with hydrodynamic performance and ecological niche’, *Journal of Morphology*, 267, pp. 1284–1294. doi: 10.1002/jmor.10474.

# Appendix 1: Species decision tree for cetaceans observed in VHR satellite imagery

**Important considerations when using the “Species decision tree for cetaceans observed in VHR satellite imagery”:**

- Figure S1.1 only includes the species that have confidently been observed in satellite imagery, which on 16^th^ June 2022 includes: narwhal (*Monodon monoceros*), beluga (*Delphinapterus leucas*), Eubalaena spp., fin whale (*Balaenoptera physalus*), humpback whale (*Megaptera novaeangliae*), and gray whale (*Eschrichtius robustus*).
- Biogeography: the location of the image will play an important role in determining the species, particularly for Eubalaena spp. Therefore, the “Species decision tree for cetaceans previously observed in VHR satellite imagery” needs to be used alongside known distribution map. For example narwhals are only found in the Arctic.
  - Suggested references: IUCN Red List (www.iucnredlist.org) and the Encyclopedia of Marine Mammals (https://www.sciencedirect.com/book/9780128043271/encyclopedia-of-marine-mammals)
- Full body here refers to seeing the head to at least the peduncle of the potential whale-object

**
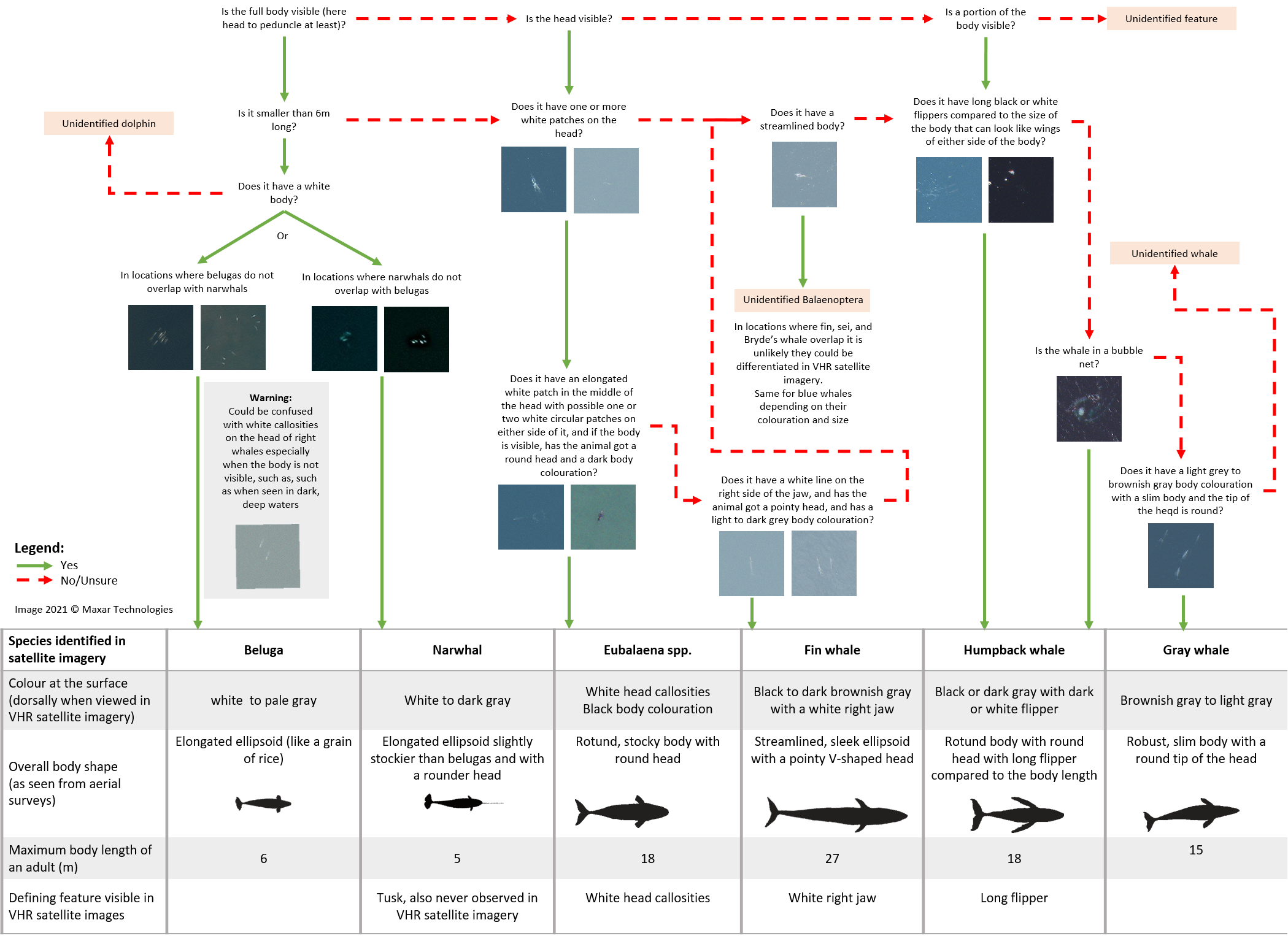
**

**Figure S1.1.** Species decision tree for cetaceans observed in VHR satellite imagery.

# Appendix 2: Species code

Table S2.1. List of species codes for cetaceans that can be identified in very high-resolution satellite images.

| **sp_code** | **Scientific Name** | **Common Name** |
| --- | --- | --- |
| BELU | *Delphinapterus leucas* | Beluga |
| NAR | *Monoceros monoceros* | Narwhal |
| RIWH | *Eubalaena spp.* | Right whale |
| FIWH | *Balaenoptera physalus* | Fin whale |
| HUWH | *Megaptera novaeangliae* | Humpback whale |
| GRWH | *Eschrichtius robustus* | Gray whale |
| UNBA | NA | Unidentified Balaenoptera |
| UNWH | NA | Unidentified whale |
| UNDO | NA | Unidentified dolphin |
| UNFE | NA | Unidentified feature |

# Appendix 3: Assigning a certainty level

**Steps to Classify Whale Species in VHR Satellite Imagery and Assign Certainty Levels:**

1. **Background Research**

Before reviewing a very high-resolution (VHR) satellite image for the presence of whales, examine the images in Tables S3.1-4 to familiarize yourself with what the different species look like. Where VHR satellite imagery is not available for a particular species, we have provided examples from higher resolution aerial imagery captured with DSLR camera, but bear in mind that satellite images will appear more blurry or grainy. The species list is not exhaustive, so if there are other large animals in your study area, familiarise yourself with examples of these species in aerial images.

1. **Species Determination**

Once you have reviewed the available material and detected a potential whale in a satellite image, use the Species Decision Tree (Appendix 1 or Supplementary material 3) to assign a species or the next higher taxonomic level.

1. **Certainty**

Assign a certainty level based on the list of cues in Tables S3.1, S3.2, and S3.3, and examples of various species at various spatial resolution (Table S3.4):

- - **Definite**: you are confident in your species determination (90-100%)
  - **Probable**: you think that your species determination is likely but you are not sure (60-90%)
  - **Possible**: you think that your species determination is possible but it is hard to tell (10-60%)

**Table S3.1.** Individual characteristics to help confirm species identification based on (Woodward, Winn and Fish, 2006; Jefferson, Webber and Pitman, 2008; Williams, Noren and Glenn, 2011; Larrat and Lair, 2022). Examples in very high-resolution satellite imagery and aerial images are given. Grayed-out cells indicate no imagery was available.

| **Cue** | **Description** | **Narwhal** | **Beluga** | **Gray whale** | **Fin whale** | **Humpback whale** | **Eubalaena spp.** |
| --- | --- | --- | --- | --- | --- | --- | --- |
| Body coloration | Color at the surface (dorsally when viewed in VHR satellite imagery) | White to dark gray | White to dark gray | Brownish gray to light gray | Black to dark brownish gray with a white right jaw | Black or dark gray with dark or white flipper | Black body with white head callosities |
| Body shape | Overall shape of the body excluding fluke and flippers | Elongated ellipsoid slightly stockier than belugas and with a rounder head | Elongated ellipsoid (like a grain of rice) | If full body visible: Robust, slim ellipsoid with a round tip of the head; if full body not visible: ellipsoid; if only head: circular (*e.g.*, when spy-hopping) or triangular with rounded angle | If full body visible: streamlined, sleek ellipsoid with a pointy V-shaped head; if full body not visible: ellipsoid; if only head: triangular with rounded angle | If full body visible: Rotund ellipsoid with round head; if full body not visible: ellipsoid; if only head: circular (*e.g.*, when spy-hopping) or triangular with rounded angle | If full body visible: Rotund, stocky ellipsoid with round head; if full body not visible: ellipsoid; if only head: circular (*e.g.*, when spy-hopping) or triangular with rounded angle |
| Body length | Maximum visible length between the tip of the head and the fluke with values ranging from calf size to maximum adult length | 1.6 – 4.2 m  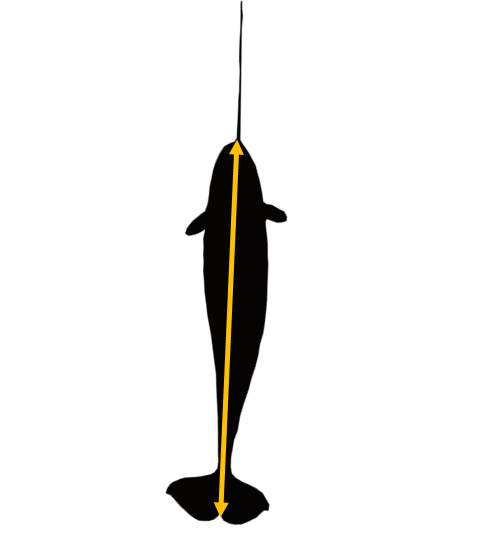 | 1.6 – 5.5 m  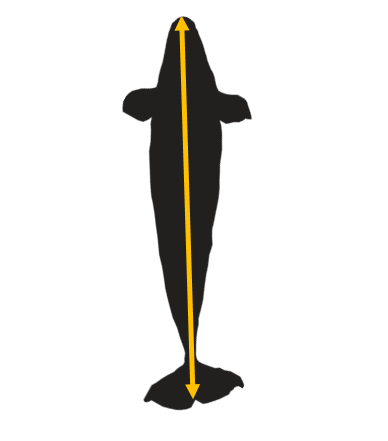 | 4.6 m - 15 m  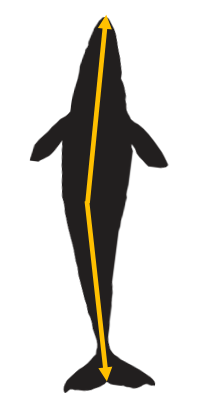 | 6 m - 27 m  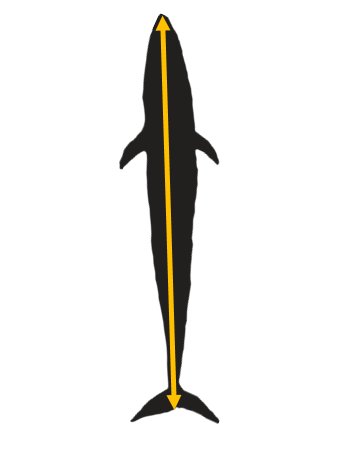 | 4 m - 18 m  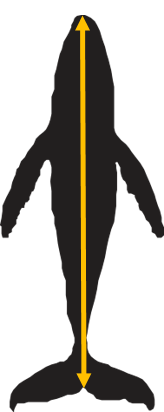 | 4 m - 18 m  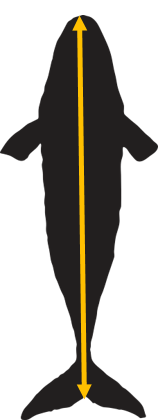 |
| Body width | It is measured at the widest part of the body and perpendicular the body length. | ≤0.82 m  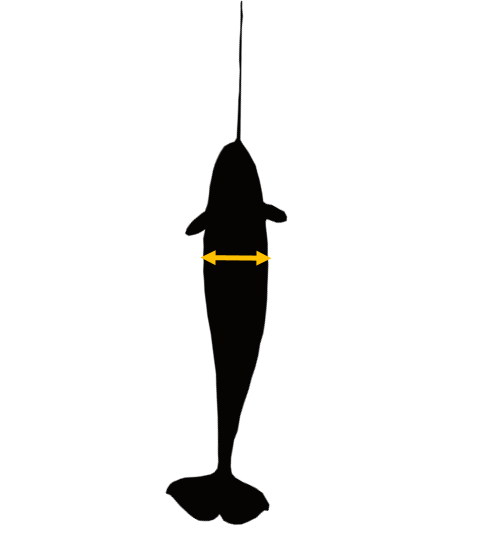 | ≤0.75 m  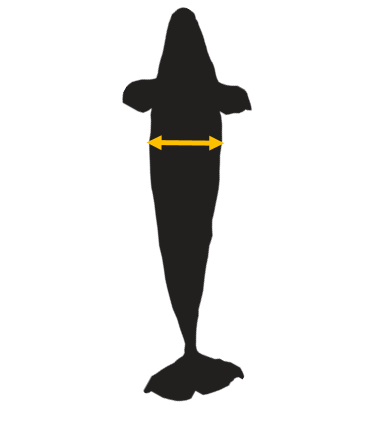 | ≤ 2.2 m | ≤ 3.9 m | ≤ 3.2 m | ≤ 3.3 m |
| Flipper – VHR | Forelimb used to stabilise and turn. |  |  |  |  | *See below “Long flipper”* |  |
| Flipper - Aerial |  |  |  |  |  |  |  |
| Long Flipper - VHR | Species specific – Humpback whale flippers are one third of the body length. | *NA* | *NA* | *NA* | *NA* |  | *NA* |
| Long flipper - Aerial |  |  |  |  |  |  |  |
| Fluke - VHR | Tail used to generate thrust. |  |  |  |  |  |  |
| Fluke - Aerial |  |  |  |  |  |  |  |
| White head callosities - VHR | Species specific – only reported for the species of the genus Eubalaena.  White patches on top of the head. | *NA* | *NA* | *NA* | *NA* | *NA* |  |
| White head callosities - Aerial |  |  |  |  |  |  |  |
| White right lower jaw - VHR | Species specific – only reported for fin whales.  White coloration of the lower right jaw. | *NA* | *NA* | *NA* |  | *NA* | *NA* |
| White right lower jaw - Aerial |  |  |  |  |  |  |  |

**Table S3.2.** Behavioural cues indicating the presence of whales related to sea surface disturbance. Examples in very high-resolution satellite imagery and aerial images are given. Grayed-out cells indicate no imagery was available

| **Cue** | **Description** | **Narwhal** | **Beluga** | **Gray whale** | **Fin whale** | **Humpback whale** | **Eubalaena spp.** |
| --- | --- | --- | --- | --- | --- | --- | --- |
| After-breach - VHR | Large white area left after a whale breached, or lobtailed, flipper-slapped. |  |  |  |  |  |  |
| After-breach - Aerial |  |  |  |  |  |  |  |
| Bubble net – VHR | Species specific - only reported for Humpback whales  One white spiral formed of several white circular patches, or several white spirals nested together. | *NA* | *NA* | *NA* | *NA* |  | *NA* |
| Bubble net – Aerial |  |  |  |  |  |  |  |
| Contour – VHR | White line separating the part of the whale body that is above and below the sea surface (*e.g.*, when a whale is rolling its back or surfacing to breathe). |  |  |  |  |  |  |
| Contour - Aerial |  |  |  |  |  |  |  |
| Flukeprint – VHR | White circle left after whale dove or while swimming (Levy *et al.*, 2011). |  |  |  |  |  |  |
| Flukeprint – Aerial |  |  |  |  |  |  |  |
| Wake – VHR | V-shaped white trail behind the animal. |  |  |  |  |  |  |
| Wake - Aerial |  |  |  |  |  |  |  |

**Table S3.3.** Other cues indicating the presence of whales (not related to sea surface disturbance or cetaceans’ body). Examples in very high-resolution satellite imagery and aerial images are given. Grayed-out cells indicate no imagery was available

| **Cue** | **Description** | **Narwhal** | **Beluga** | **Gray whale** | **Fin whale** | **Humpback whale** | **Eubalaena spp.** |
| --- | --- | --- | --- | --- | --- | --- | --- |
| Blow – VHR | Vaporous whitish patch next to a whale, similar looking to fog. |  |  |  |  |  |  |
| Blow – Aerial |  |  |  |  |  |  |  |
| Mudtrail – VHR | Plume/cloud of substrate behind a whale. |  |  |  |  |  |  |
| Mudtrail - Aerial |  |  |  |  |  |  |  |
| Surface active group - VHR | Two or more whales rolling and touching at the surface. |  |  |  |  |  |  |
| Surface active group – Aerial |  |  |  |  |  |  |  |
| Travelling group - VHR | Two or more cetaceans travelling together in the same direction and less than a few meters apart. |  |  |  |  |  |  |
| Travelling group - Aerial |  |  |  |  |  |  |  |
| Mother-calf pair - Aerial | When a calf is in the close proximity of an adult whale. |  |  |  |  |  |  |
| Mother-calf pair - VHR |  |  |  |  |  |  |  |
| Defecation – VHR | Trail of colored clouds behind animal. |  |  |  |  |  |  |

**Table S3.4.** Examples of narwhal, beluga, gray whale, fin whale, humpback whale and Eubalaena spp. in 30 cm and 50 cm very high-resolution (VHR) satellite imagery, and in aerial images. Grayed-out cells indicate no imagery was available.

|  | **Narwhal** | **Beluga** | **Gray whale** | **Fin whale** | **Humpback whale** | **Eubalaena spp.** |
| --- | --- | --- | --- | --- | --- | --- |
| 30cm VHR satellite imagery |  |  |  |  |  |  |
| 50 cm VHR satellite imagery |  |  |  |  |  |  |
| Aerial images |  |  |  |  |  |  |
