# Supplementary material for: Annotating very high-resolution satellite imagery: A whale case study
Source: MethodsX. 2023 Jan 25;10:102040. doi: 10.1016/j.mex.2023.102040 (PMC9923222; doi:10.1016/j.mex.2023.102040)
Supplement: Supplementary materiel 3 — Species decision tree for cetaceans observed in VHR satellite imagery. [file mmc3.zip › Supplementary3_Species-tree.docx]

# Supplementa material 3: Species decision tree for cetaceans observed in VHR satellite imagery

**Important considerations when using the “Species decision tree for cetaceans observed in VHR satellite imagery”:**

- Figure S1.1 only includes the species that have confidently been observed in satellite imagery, which on 16^th^ June 2022 includes: narwhal (*Monodon monoceros*), beluga (*Delphinapterus leucas*), Eubalaena spp., fin whale (*Balaenoptera physalus*), humpback whale (*Megaptera novaeangliae*), and gray whale (*Eschrichtius robustus*).
- Biogeography: the location of the image will play an important role in determining the species, particularly for Eubalaena spp. Therefore, the “Species decision tree for cetaceans previously observed in VHR satellite imagery” needs to be used alongside known distribution map. For example narwhals are only found in the Arctic.
  - Suggested references: IUCN Red List (www.iucnredlist.org) and the Encyclopedia of Marine Mammals (https://www.sciencedirect.com/boOK/9780128043271/encyclopedia-of-marine-mammals)
- Full body here refers to seeing the head to at least the peduncle of the potential whale-object

**
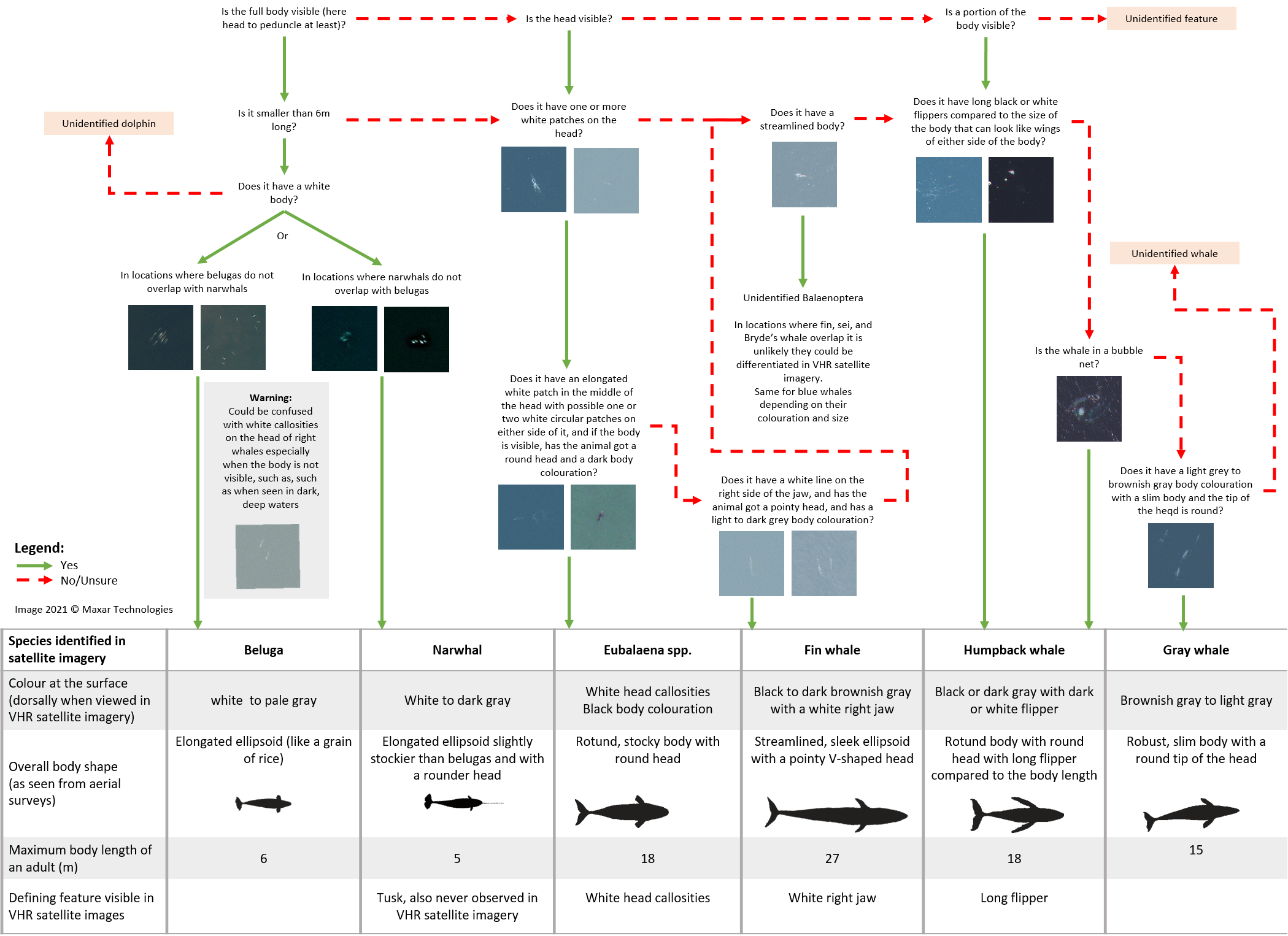
**

**Figure S1.1.** Species decision tree for cetaceans observed in VHR satellite imagery.
