# Supplementary material for: Annotating very high-resolution satellite imagery: A whale case study
Source: MethodsX. 2023 Jan 25;10:102040. doi: 10.1016/j.mex.2023.102040 (PMC9923222; doi:10.1016/j.mex.2023.102040)
Supplement: Supplementary materiel 4 — Species code. [file mmc4.zip › Supplementary4_Species-code.docx]

# Supplementary material 4: Species code

Table S2.1. List of species codes for cetaceans that can be identified in very high-resolution satellite images.

| **sp_code** | **Scientific Name** | **Common Name** |
| --- | --- | --- |
| BELU | *Delphinapterus leucas* | Beluga |
| NAR | *Monoceros monoceros* | Narwhal |
| RIWH | *Eubalaena spp.* | Right whale |
| FIWH | *Balaenoptera physalus* | Fin whale |
| HUWH | *Megaptera novaeangliae* | Humpback whale |
| GRWH | *Eschrichtius robustus* | Gray whale |
| UNBA | NA | Unidentified Balaenoptera |
| UNWH | NA | Unidentified whale |
| UNDO | NA | Unidentified dolphin |
| UNFE | NA | Unidentified feature |
