# Supplementary material for: Annotating very high-resolution satellite imagery: A whale case study
Source: MethodsX. 2023 Jan 25;10:102040. doi: 10.1016/j.mex.2023.102040 (PMC9923222; doi:10.1016/j.mex.2023.102040)
Supplement: Supplementary materiel 5 — Assigning a certainty level to whale observed in VHR satellite imagery. [file mmc5.zip › Supplementary5_Certainty_level.docx]

# Supplementary material 5: Assigning a certainty level

**Steps to Classify Whale Species in VHR Satellite Imagery and Assign Certainty Levels:**

1. **Background Research**

Before reviewing a very high-resolution (VHR) satellite image for the presence of whales, examine the images in Tables S3.1-4 to familiarize yourself with what the different species look like. Where VHR satellite imagery is not available for a particular species, we have provided examples from higher resolution aerial imagery captured with DSLR camera, but bear in mind that satellite images will appear more blurry or grainy. The species list is not exhaustive, so if there are other large animals in your study area, familiarise yourself with examples of these species in aerial images.

1. **Species Determination**

Once you have reviewed the available material and detected a potential whale in a satellite image, use the Species Decision Tree (Supplementary material 3) to assign a species or the next higher taxonomic level.

1. **Certainty**

Assign a certainty level based on the list of cues in Tables S3.1, S3.2, and S3.3, and examples of various species at various spatial resolution (Table S3.4):

- - **Definite**: you are confident in your species determination (90-100%)
  - **Probable**: you think that your species determination is likely but you are not sure (60-90%)
  - **Possible**: you think that your species determination is possible but it is hard to tell (10-60%)

**Table S3.1.** Individual characteristics to help confirm species identification based on (Woodward, Winn and Fish, 2006; Jefferson, Webber and Pitman, 2008; Williams, Noren and Glenn, 2011; Larrat and Lair, 2022). Examples in very high-resolution satellite imagery and aerial images are given. Grayed-out cells indicate no imagery was available

| **Cue** | **Description** | **Narwhal** | **Beluga** | **Gray whale** | **Fin whale** | **Humpback whale** | **Eubalaena spp.** |
| --- | --- | --- | --- | --- | --- | --- | --- |
| Body coloration | Color at the surface (dorsally when viewed in VHR satellite imagery) | White to dark gray | White to dark gray | Brownish gray to light gray | Black to dark brownish gray with a white right jaw | Black or dark gray with dark or white flipper | Black body with white head callosities |
| Body shape | Overall shape of the body excluding fluke and flippers | Elongated ellipsoid slightly stockier than belugas and with a rounder head | Elongated ellipsoid (like a grain of rice) | If full body visible: Robust, slim ellipsoid with a round tip of the head; if full body not visible: ellipsoid; if only head: circular (*e.g.*, when spy-hopping) or triangular with rounded angle | If full body visible: streamlined, sleek ellipsoid with a pointy V-shaped head; if full body not visible: ellipsoid; if only head: triangular with rounded angle | If full body visible: Rotund ellipsoid with round head; if full body not visible: ellipsoid; if only head: circular (*e.g.*, when spy-hopping) or triangular with rounded angle | If full body visible: Rotund, stocky ellipsoid with round head; if full body not visible: ellipsoid; if only head: circular (*e.g.*, when spy-hopping) or triangular with rounded angle |
| Body length | Maximum visible length between the tip of the head and the fluke with values ranging from calf size to maximum adult length | 1.6 – 4.2 m  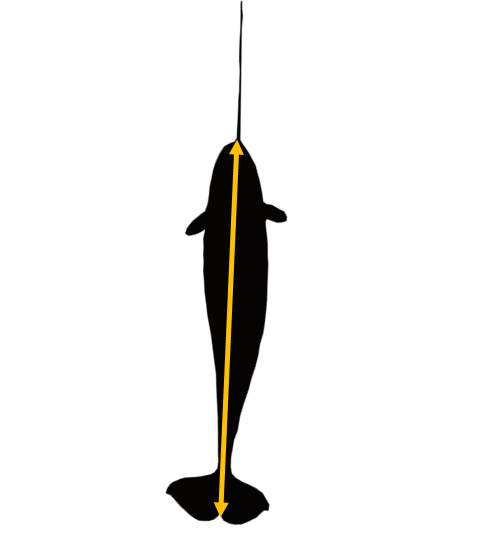 | 1.6 – 5.5 m  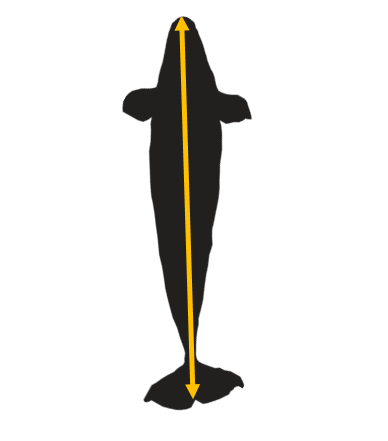 | 4.6 m - 15 m  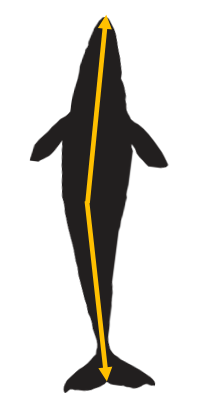 | 6 m - 27 m  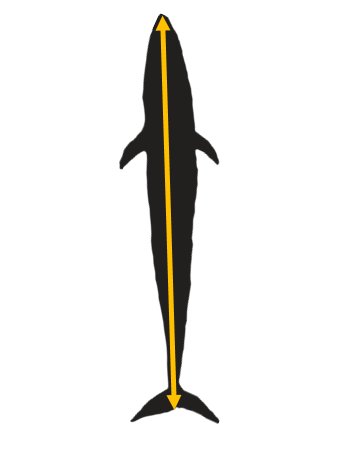 | 4 m - 18 m  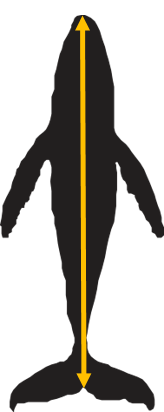 | 4 m - 18 m  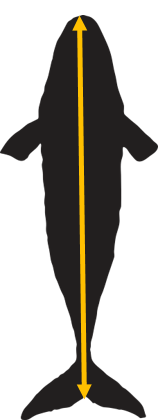 |
| Body width | It is measured at the widest part of the body and perpendicular the body length. | ≤0.82 m  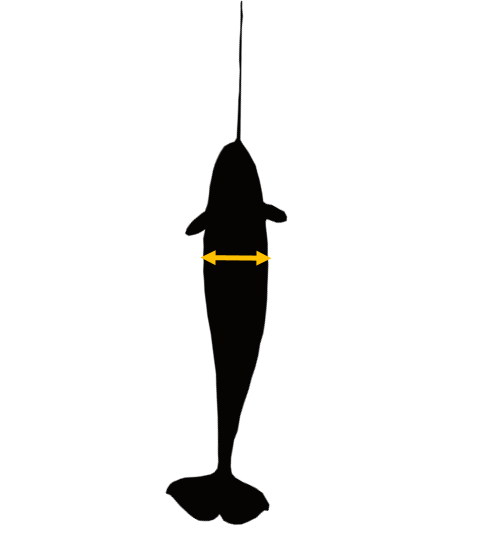 | ≤0.75 m  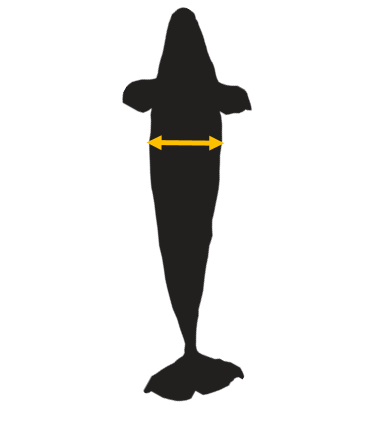 | ≤ 2.2 m  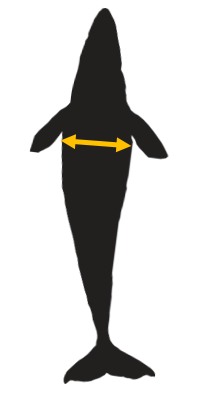 | ≤ 3.9 m  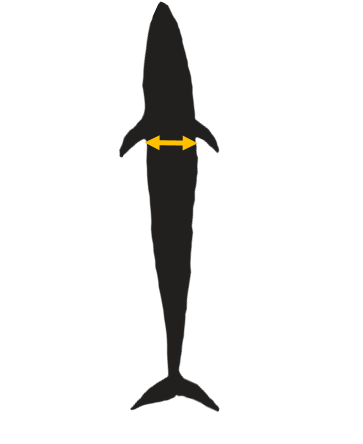 | ≤ 3.2 m  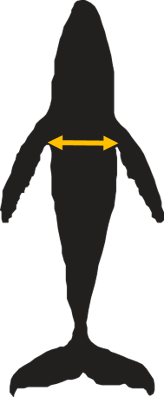 | ≤ 3.3 m  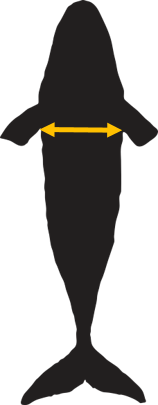 |
| Flipper – VHR | Forelimb used to stabilise and turn. |  |  |  | 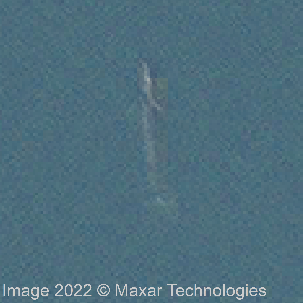 | *See below “Long flipper”* |  |
| Flipper - Aerial |  |  | 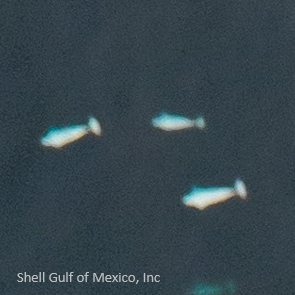 | 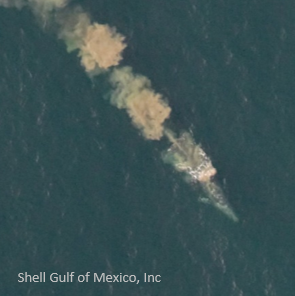 | **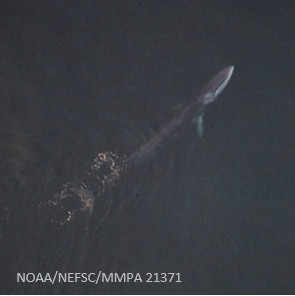** |  | 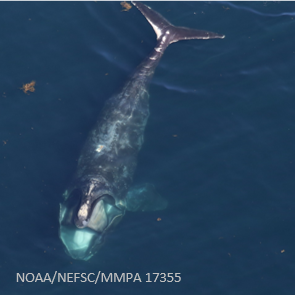 |
| Long Flipper - VHR | Species specific – Humpback whale flippers are one third of the body length. | *NA* | *NA* | *NA* | *NA* | 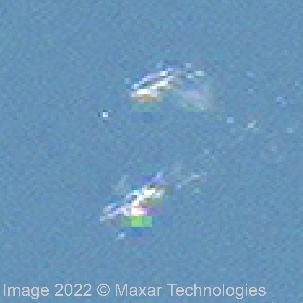 | *NA* |
| Long flipper - Aerial |  |  |  |  |  | **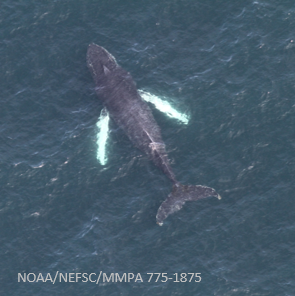** |  |
| Fluke - VHR | Tail used to generate thrust. |  | 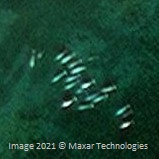 | 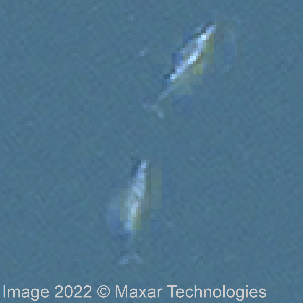 | 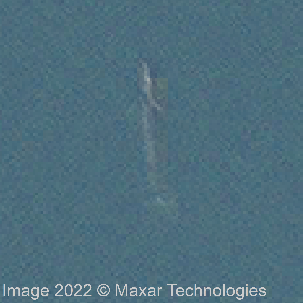 | 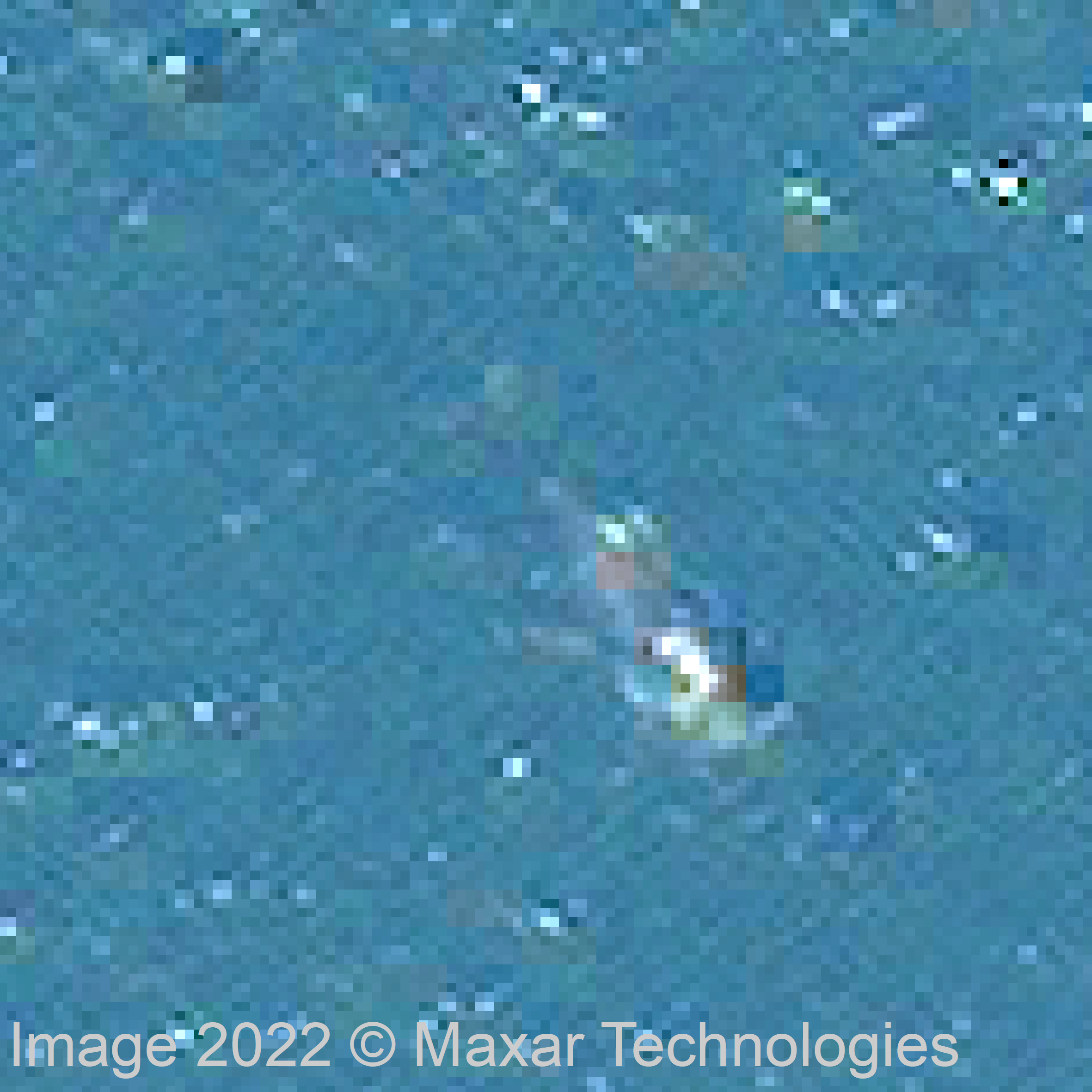 | 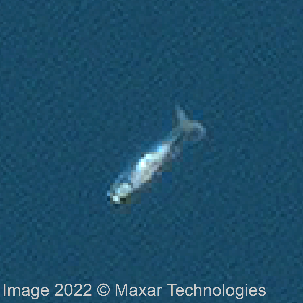 |
| Fluke - Aerial |  | 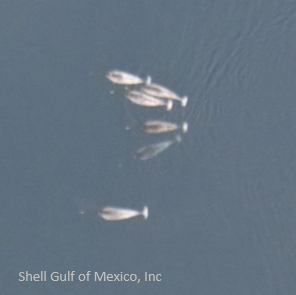 | **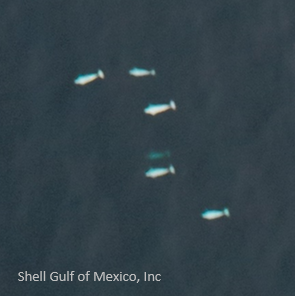** | **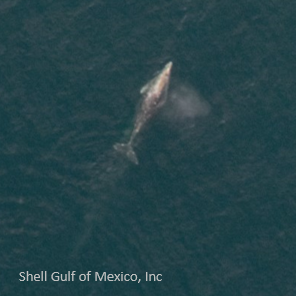** | **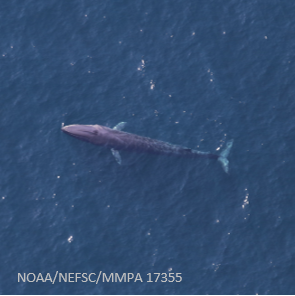** | **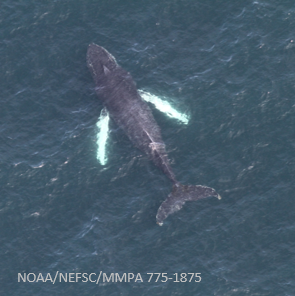** | 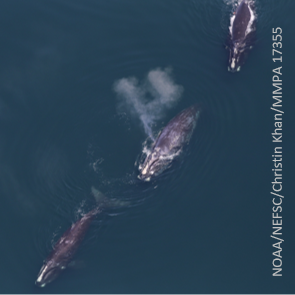 |
| White head callosities - VHR | Species specific – only reported for the species of the genus Eubalaena.  White patches on top of the head. | *NA* | *NA* | *NA* | *NA* | *NA* | 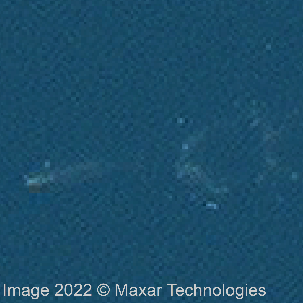 |
| White head callosities - Aerial |  |  |  |  |  |  | 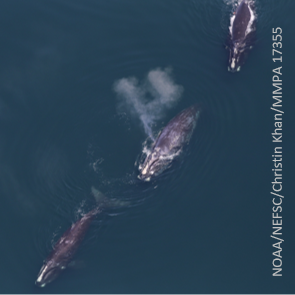 |
| White right lower jaw - VHR | Species specific – only reported for fin whales.  White coloration of the lower right jaw. | *NA* | *NA* | *NA* | 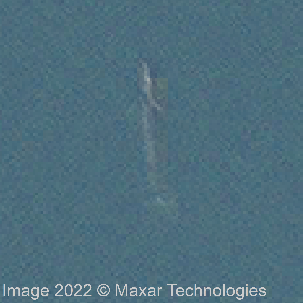 | *NA* | *NA* |
| White right lower jaw - Aerial |  |  |  |  | **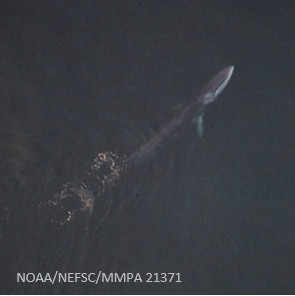** |  |  |

**Table S3.2.** Behavioural cues indicating the presence of whales related to sea surface disturbance. Examples in very high-resolution satellite imagery and aerial images are given. Grayed-out cells indicate no imagery was available

| **Cue** | **Description** | **Narwhal** | **Beluga** | **Gray whale** | **Fin whale** | **Humpback whale** | **Eubalaena spp.** |
| --- | --- | --- | --- | --- | --- | --- | --- |
| After-breach - VHR | Large white area left after a whale breached, or lobtailed, flipper-slapped. |  |  |  |  | 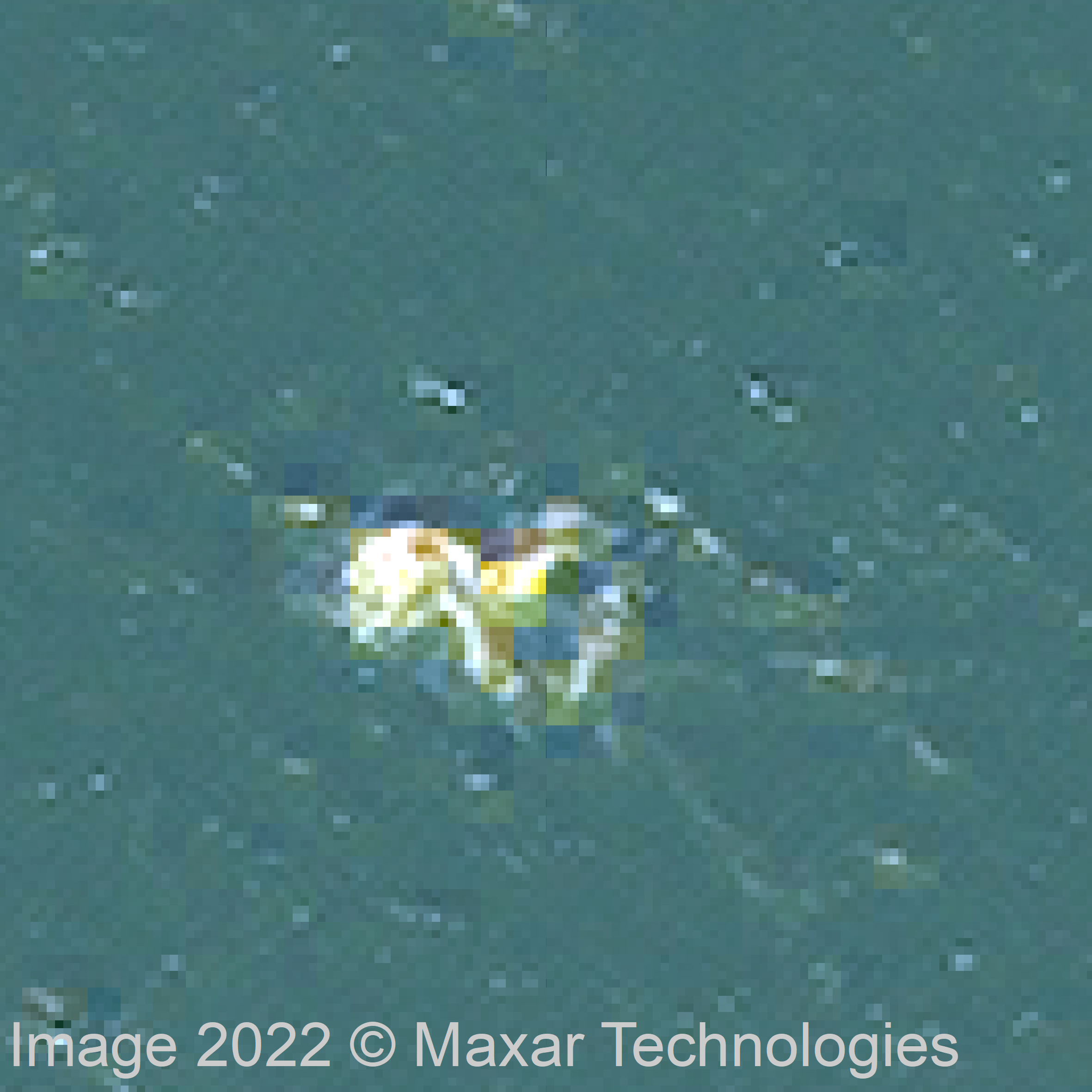 |  |
| After-breach - Aerial |  |  |  |  |  |  | 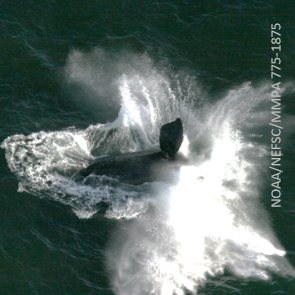 |
| Bubble net – VHR | Species specific - only reported for Humpback whales  One white spiral formed of several white circular patches, or several white spirals nested together. | *NA* | *NA* | *NA* | *NA* | 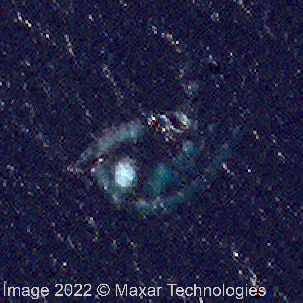 | *NA* |
| Bubble net – Aerial |  |  |  |  |  | 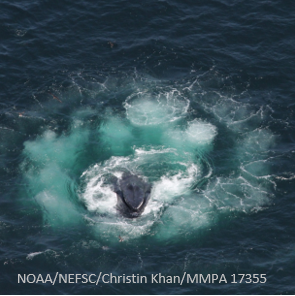 |  |
| Contour – VHR | White line separating the part of the whale body that is above and below the sea surface (*e.g.*, when a whale is rolling its back or surfacing to breathe). |  |  | 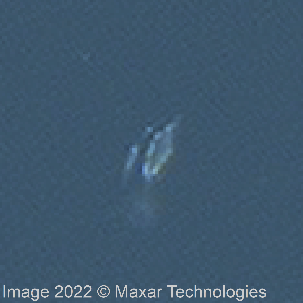 | 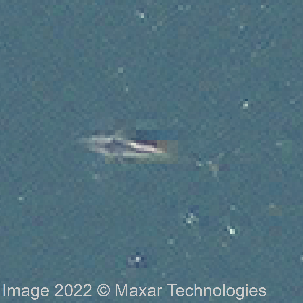 | 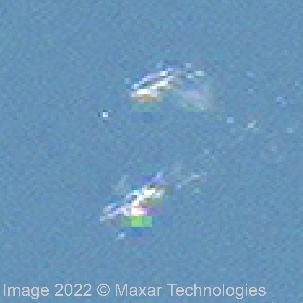 | 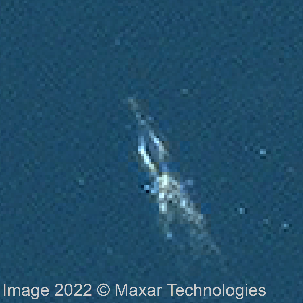 |
| Contour - Aerial |  |  |  |  | 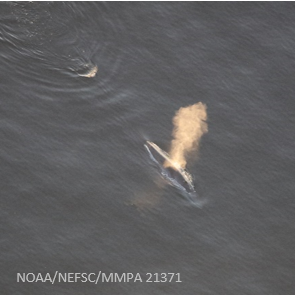 | 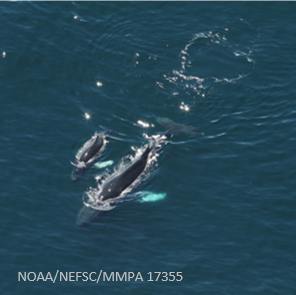 | 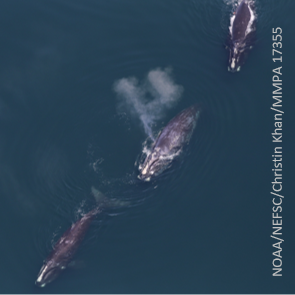 |
| Flukeprint – VHR | White circle left after whale dove or while swimming (Levy *et al.*, 2011). |  |  | 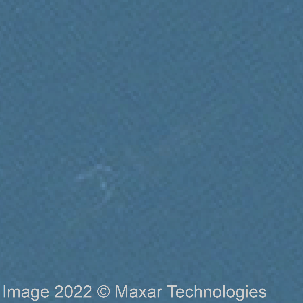 | 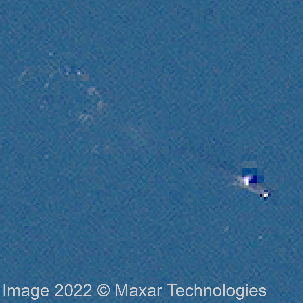 | 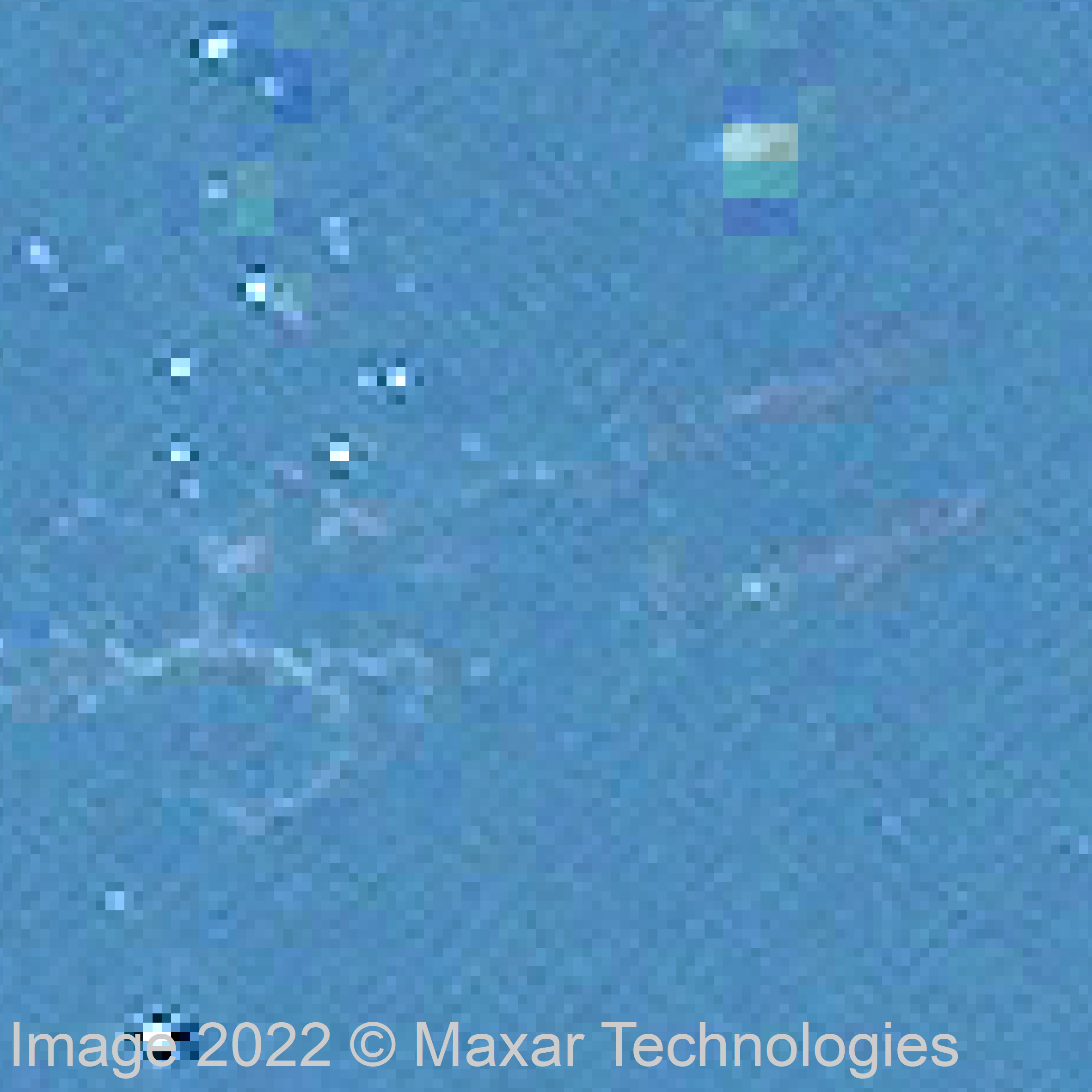 | 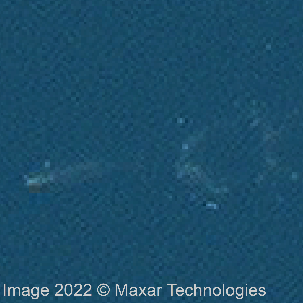 |
| Flukeprint – Aerial |  |  |  |  |  | 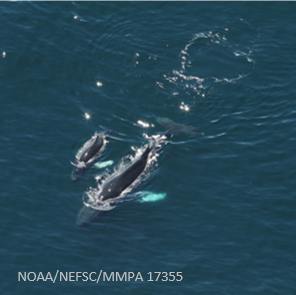 |  |
| Wake – VHR | V-shaped white trail behind the animal. |  |  |  | 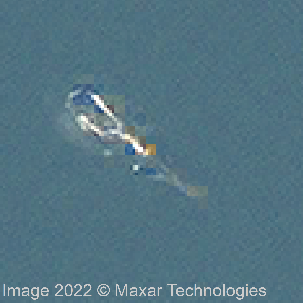 | 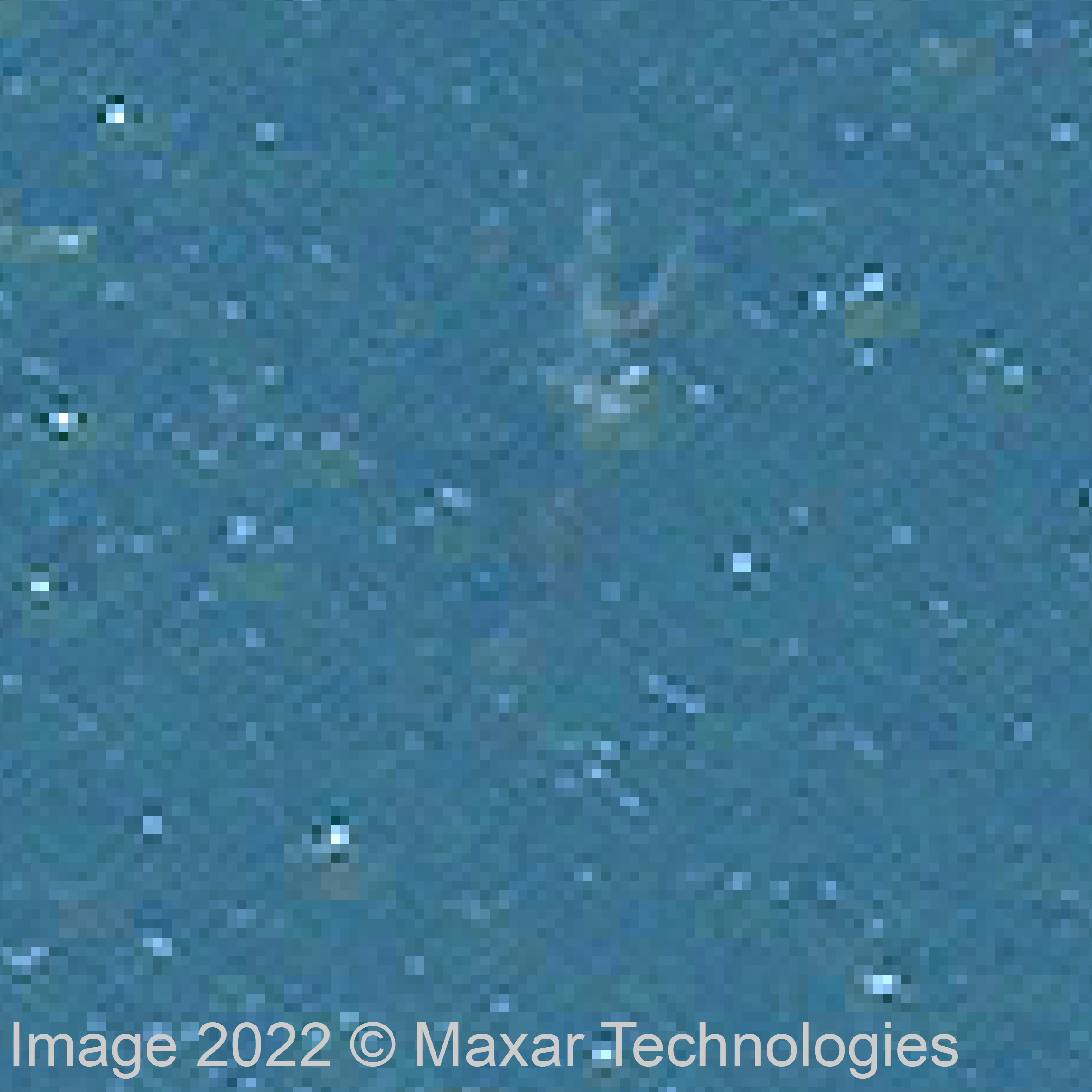 | 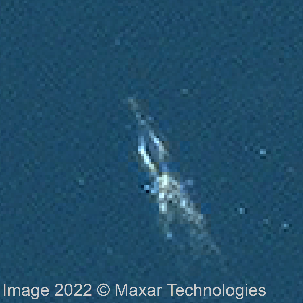 |
| Wake - Aerial |  |  |  |  |  | 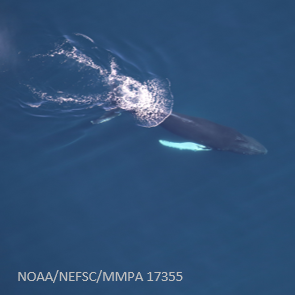 |  |

**Table S3.3.** Other cues indicating the presence of whales (not related to sea surface disturbance or cetaceans’ body). Examples in very high-resolution satellite imagery and aerial images are given. Grayed-out cells indicate no imagery was available

| **Cue** | **Description** | **Narwhal** | **Beluga** | **Gray whale** | **Fin whale** | **Humpback whale** | **Eubalaena spp.** |
| --- | --- | --- | --- | --- | --- | --- | --- |
| Blow – VHR | Vaporous whitish patch next to a whale, similar looking to fog. |  |  | 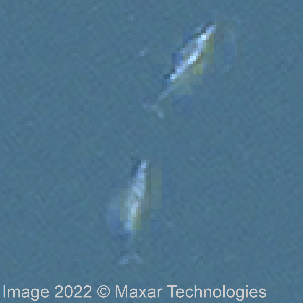 |  | 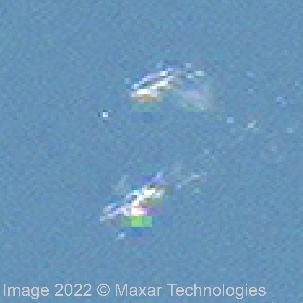 | 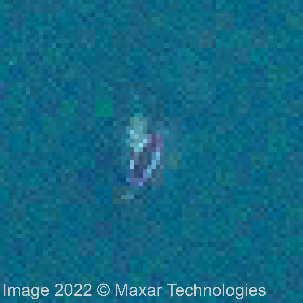 |
| Blow – Aerial |  |  |  | 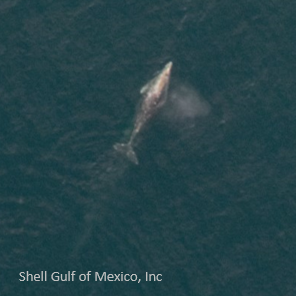 | 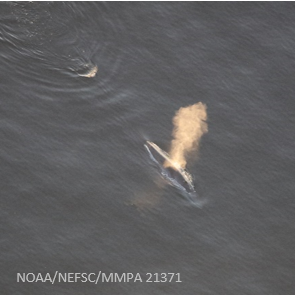 | 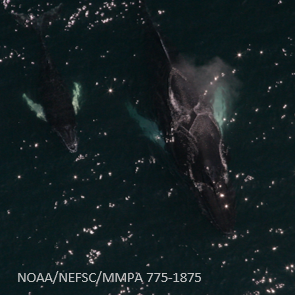 | 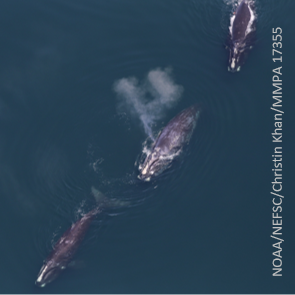 |
| Mudtrail – VHR | Plume/cloud of substrate behind a whale. |  | 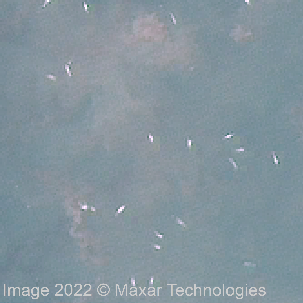 |  |  |  | 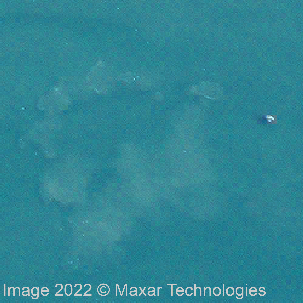 |
| Mudtrail - Aerial |  |  |  | 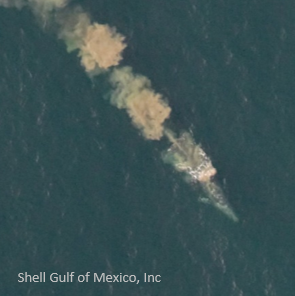 |  |  |  |
| Surface active group - VHR | Two or more whales rolling and touching at the surface. | 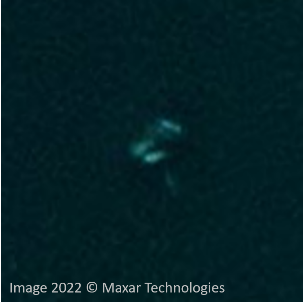 | 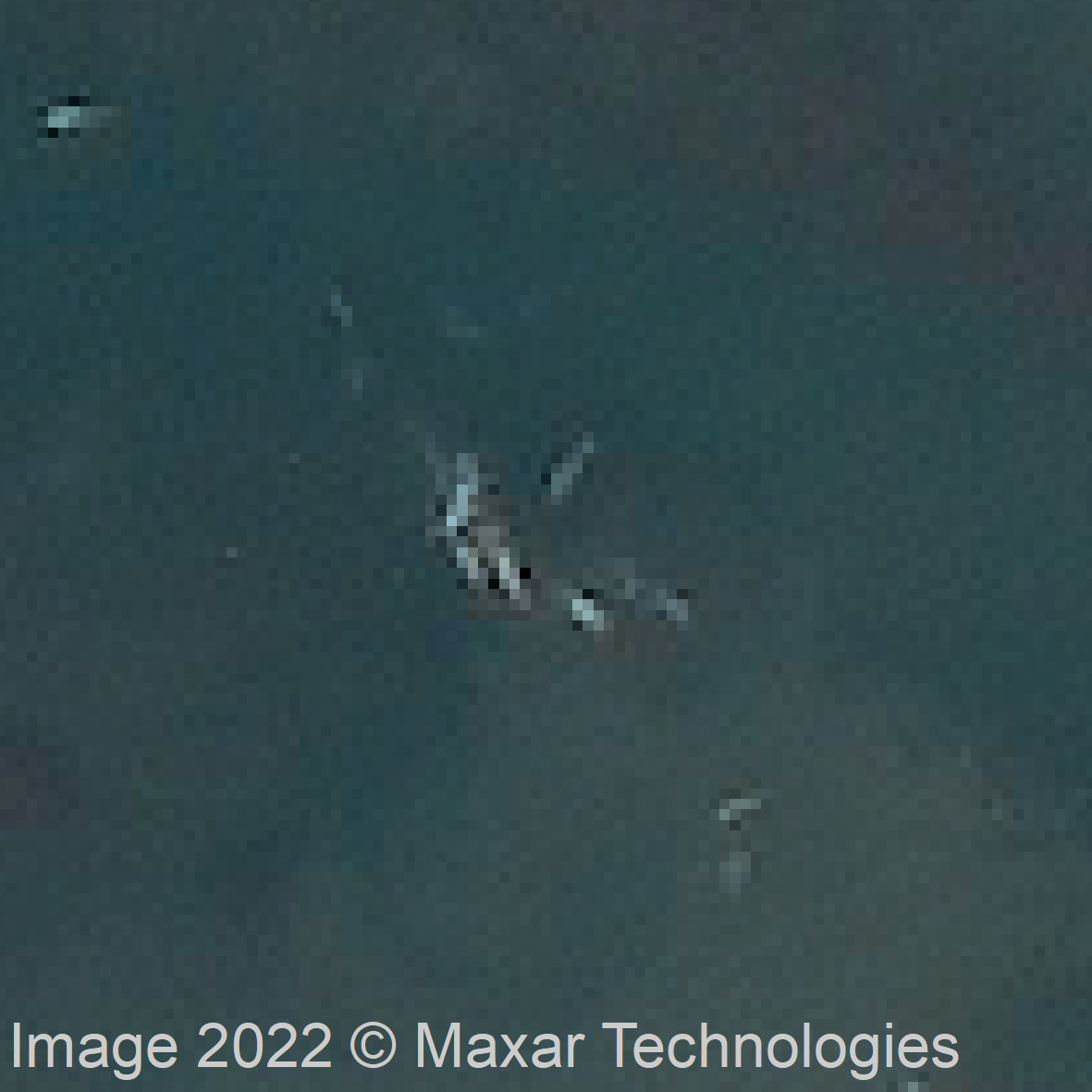 |  |  |  | 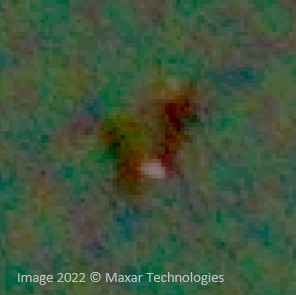 |
| Surface active group – Aerial |  |  |  |  |  |  | 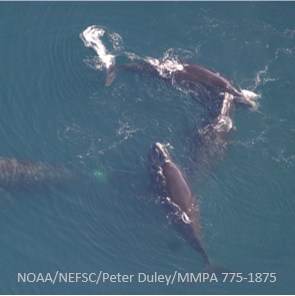 |
| Travelling group - VHR | Two or more cetaceans travelling together in the same direction and less than a few meters apart. | 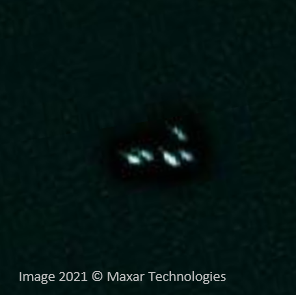 | 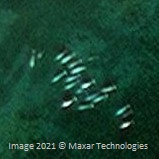 | 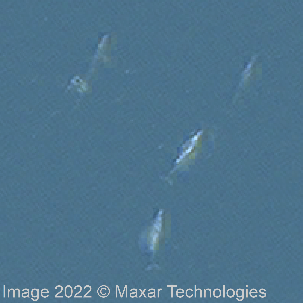 | 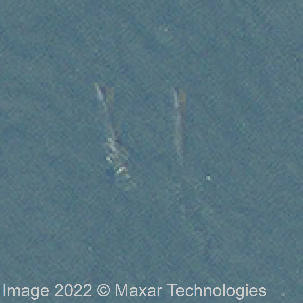 | 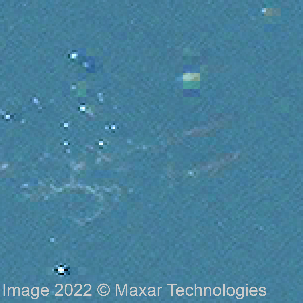 | 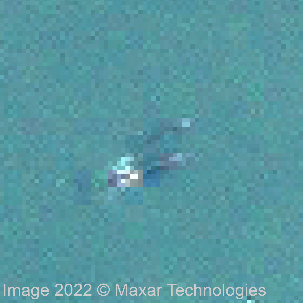 |
| Travelling group - Aerial |  | 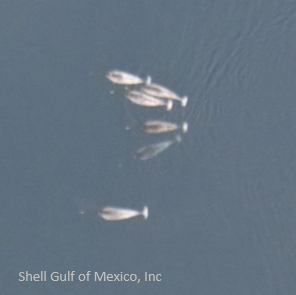 | 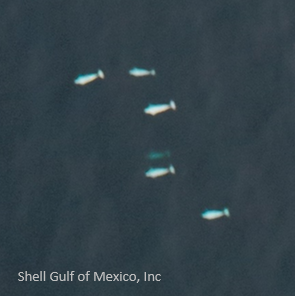 |  | 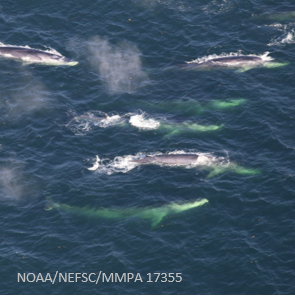 | 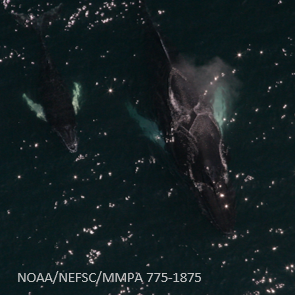 | 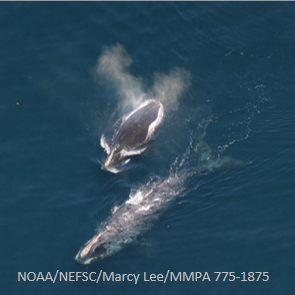 |
| Mother-calf pair - Aerial | When a calf is in the close proximity of an adult whale. |  | 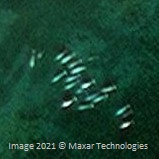 | *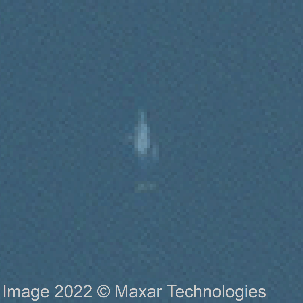* |  |  | 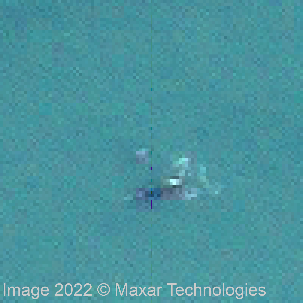 |
| Mother-calf pair - VHR |  |  |  |  |  |  |  |
| Defecation – VHR | Trail of colored clouds behind animal. |  |  |  |  |  | 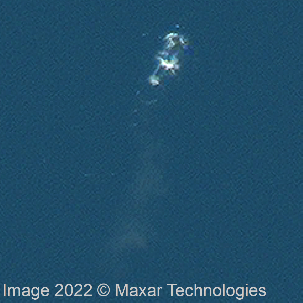 |

**Table S3.4.** Examples of narwhal, beluga, gray whale, fin whale, humpback whale and Eubalaena spp. in 30 cm and 50 cm very high-resolution (VHR) satellite imagery, and in aerial images. Grayed-out cells indicate no imagery was available.

|  | **Narwhal** | **Beluga** | **Gray whale** | **Fin whale** | **Humpback whale** | **Eubalaena spp.** |
| --- | --- | --- | --- | --- | --- | --- |
| 30cm VHR satellite imagery | **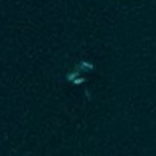** | 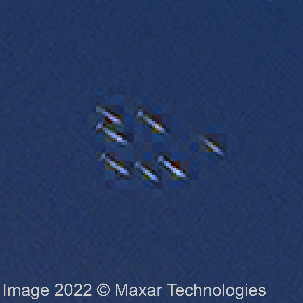 | 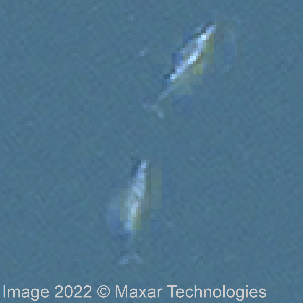 | 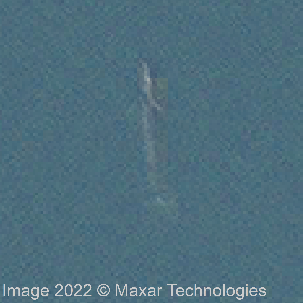 | 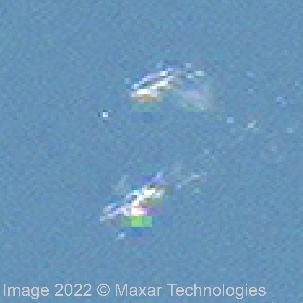 | 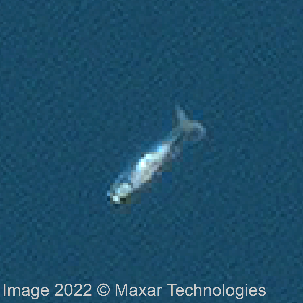 |
| 50 cm VHR satellite imagery |  | 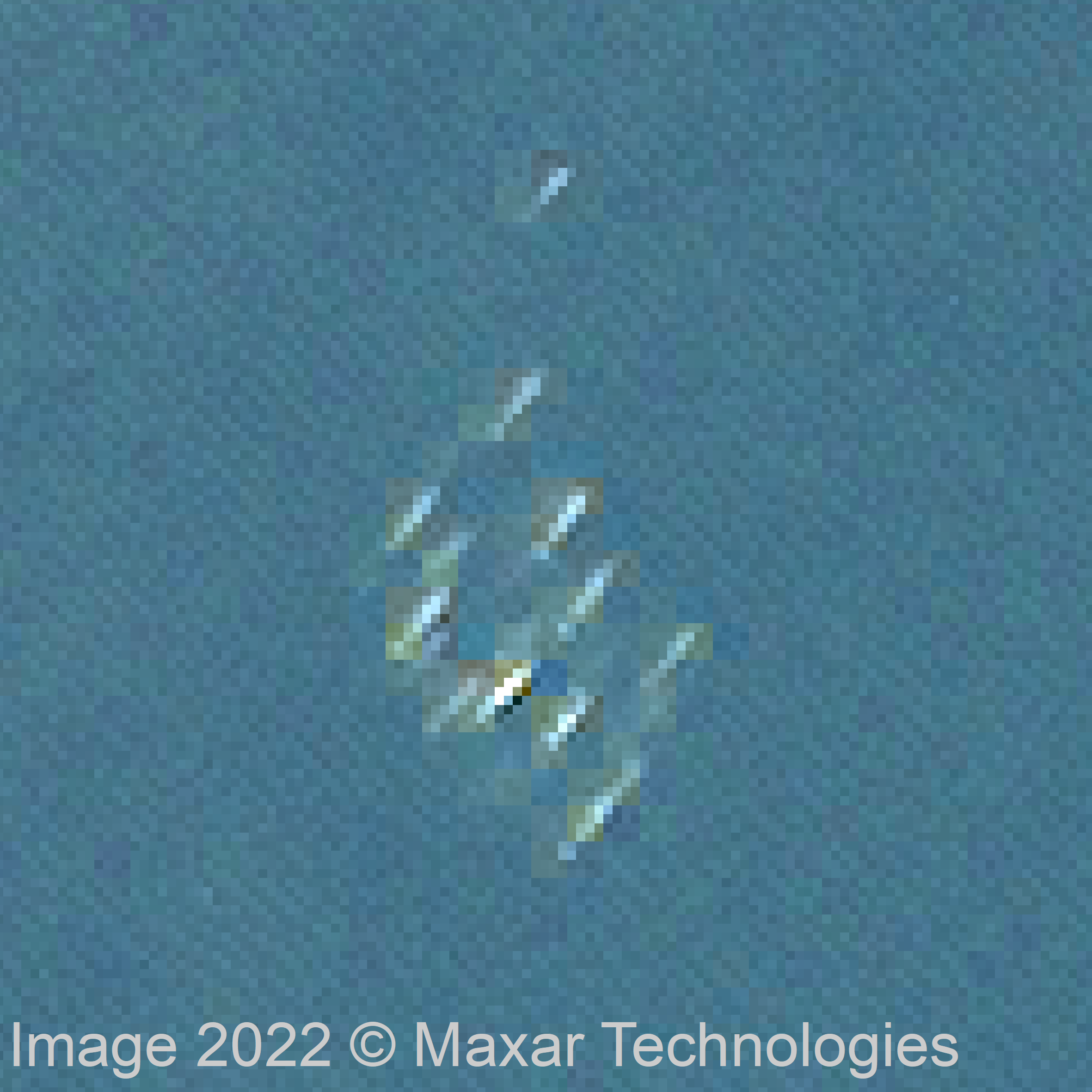 | 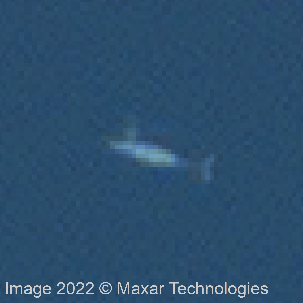 |  |  | 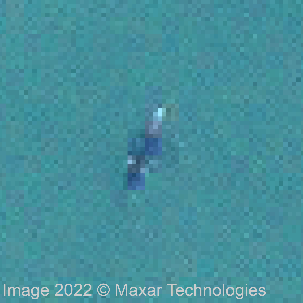 |
| Aerial images | 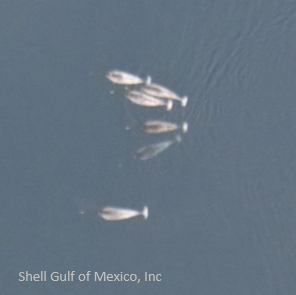 | 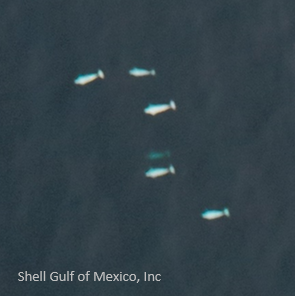 | 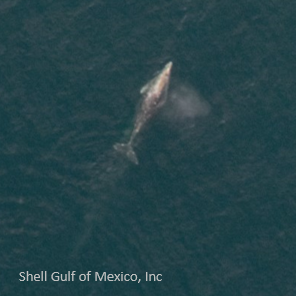 | 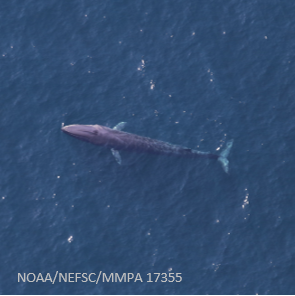 | 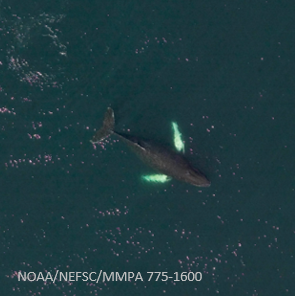 | 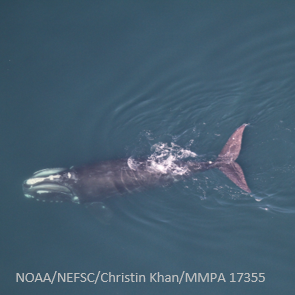 |
